# Extracellular Vesicles and Their Mimetics: A Comparative Study of Their Pharmacological Activities and Immunogenicity Profiles

**DOI:** 10.3390/pharmaceutics15041290

**Published:** 2023-04-20

**Authors:** Wei Heng Chng, Ram Pravin Kumar Muthuramalingam, Charles Kang Liang Lou, Silas New, Yub Raj Neupane, Choon Keong Lee, Ayca Altay Benetti, Chenyuan Huang, Praveen Thoniyot, Wei Seong Toh, Jiong-Wei Wang, Giorgia Pastorin

**Affiliations:** 1 Integrative Sciences and Engineering Programme, NUS Graduate School, National University of Singapore, Singapore 119077, Singapore; 2 Department of Pharmacy, National University of Singapore, Singapore 117543, Singapore; 3 Department of Surgery, Yong Loo Lin School of Medicine, National University of Singapore, Singapore 119228, Singapore; 4 Nanomedicine Translational Research Programme, Centre for Nanomedicine, Yong Loo Lin School of Medicine, National University of Singapore, Singapore 119609, Singapore; 5 Department of Pharmaceutical Sciences and Experimental Therapeutics, College of Pharmacy, The University of Iowa, Iowa City, IA 52242, USA; 6 Cardiovascular Research Institute, National University Heart Centre, Singapore 119599, Singapore; 7 Institute of Sustainability for Chemicals, Energy and Environment (ICES), A*STAR, Singapore 627833, Singapore; 8 Department of Orthopaedic Surgery, Yong Loo Lin School of Medicine, National University of Singapore, Singapore 119288, Singapore; 9 Tissue Engineering Program, Life Sciences Institute, National University of Singapore, Singapore 117510, Singapore; 10 Department of Physiology, Yong Loo Lin School of Medicine, National University of Singapore, Singapore 119593, Singapore

**Keywords:** extracellular vesicles, small extracellular vesicles, cell-derived nanovesicles, exosome mimetics, immunogenicity

## Abstract

Extracellular vesicles (EVs), which are miniaturised carriers loaded with functional proteins, lipids, and nucleic acid material, are naturally secreted by cells and show intrinsic pharmacological effects in several conditions. As such, they have the potential to be used for the treatment of various human diseases. However, the low isolation yield and laborious purification process are obstacles to their translation for clinical use. To overcome this problem, our lab developed cell-derived nanovesicles (CDNs), which are EV mimetics produced by shearing cells through membrane-fitted spin cups. To evaluate the similarities between EVs and CDNs, we compare the physical properties and biochemical composition of monocytic U937 EVs and U937 CDNs. Besides having similar hydrodynamic diameters, the produced CDNs had proteomic, lipidomic, and miRNA profiles with key communalities compared to those of natural EVs. Further characterisation was conducted to examine if CDNs could exhibit similar pharmacological activities and immunogenicity when administered in vivo. Consistently, CDNs and EVs modulated inflammation and displayed antioxidant activities. EVs and CDNs both did not exert immunogenicity when administered in vivo. Overall, CDNs could serve as a scalable and efficient alternative to EVs for further translation into clinical use.

## 1. Introduction

Extracellular vesicles (EVs) are lipid membrane-bound vesicles that are naturally secreted by cells and are unable to replicate [1]. EVs can be isolated from cell culture media and various bodily fluids such as urine and blood [2,3]. According to their biogenesis and size, there are three main categories of EVs, namely, exosomes/small EVs, microvesicles, and apoptotic bodies [3,4], even though the scientific community has reclassified them simply as EVs. Recent developments revealed that EVs are involved in intercellular communication [4]. These EVs carry and facilitate the transport of various bioactive molecules such as proteins, lipids, and nucleic acids [4,5]. They are also involved in normal physiology and in pathological conditions [6,7,8]. In addition, EVs have therapeutic potential in various disease conditions such as myocardial infarction and wound healing [9,10,11].

Despite the potential applications of EVs as therapeutics, translation into the clinical setting is limited due to the low isolation yield (a few nanograms from 1 million cells in terms of protein amount) and long isolation process [12,13]. To overcome these limitations, novel approaches to produce EV mimetics have been developed. One of these alternative methods includes the use of cell-derived nanovesicles (CDNs), which are produced by shearing cells through membrane filters via extrusion, membrane-fitted spin cups, microchannels in microfluidic devices, or by slicing cell membranes using microfabricated blades [14,15,16,17,18]. By artificially producing nanovesicles from cells, the yield (in terms of protein content) of CDNs is at least 15 times higher than that EVs, and the duration of the production process (from growing to producing the CDNs) is reduced by about twofold [18].

Previous reports showed that CDNs had similar physical properties to those of EVs, including hydrodynamic size (below 200 nm), zeta potential, and morphology [18]. Moreover, key EV protein markers such as tetraspanins and multivesicular body markers are detected on the surface membrane of CDNs [18]. Former lipidomic analyses also revealed that the major lipid classes are similar [18]. Although the physical properties and most of the biochemical composition of CDNs have remarkable communalities with EVs, research falls short in comparing the pharmacological activities of EVs and CDNs, with only a few exceptions reporting neck-to-neck comparisons [19]. It is worthwhile to investigate whether CDNs exert similar pharmacological activities as EVs derived from the same cell source. For that purpose, two main types of pharmacological activity of EVs and CDNs, namely, potential anti-inflammatory and antioxidant activities, are investigated in this work. We hypothesise that CDNs as EV mimetics exhibit similar pharmacological activities to those of their EV counterparts.

Inflammation is a biological response triggered by various factors such as infection and tissue injury. The inflammatory response helps in clearing cellular debris and initiating tissue repair [20]. However, inflammation is a double-edged sword, and excessive inflammation may further damage the tissue. EVs could modulate inflammation in various disease models [21]. For instance, mesenchymal-stem-cell-derived EVs could reduce the infiltration of inflammatory cells and attenuate the inflammatory response after an episode of myocardial infarction [22]. The current literature also indicates that EVs could induce both pro- and anti-inflammatory activities depending on the parental cell type [23].

Additionally, EVs have antioxidant properties that would be beneficial for the treatment of diseases with the dysregulation of oxidative stress. This includes cardiovascular diseases such as myocardial infarction and ischemic stroke, and neurodegenerative conditions such as Parkinson’s disease and Alzheimer’s disease. For instance, mesenchymal-stem-cell-derived EVs can decrease oxidative stress after myocardial ischemia/reperfusion injury [24].

Besides looking into the pharmacological activities of EVs and CDNs, it is important to evaluate the immunological responses and safety profiles of these nanovesicles when they are intravenously administered. As these nanovesicles may be considered foreign to the body, there might be an activation of the immune response. Immune cell profiling offers preliminary data on whether these nanovesicles are safe for administration.

Taking these aspects into account, we investigated and compared the pharmacological activities of EVs and CDNs derived from U937 monocytes, which were chosen as the parental cell type because monocytes are recruited to the disease sites in many pathological conditions such as cancer and myocardial infarction [25,26]. As immune cells, monocytes are involved in the initiation and resolution of inflammation, and the repair of damaged tissues [27,28]. U937 EVs contain antioxidant enzymes such as SOD-1, SOD-2, catalase, GSTK1, and PRDX6 [29]. Moreover, U937 monocytes were used in our previous work on the production of CDNs with the use of spin cups [18]. The monocytes had a short doubling time, and our production protocol was optimised for this cell type.

Overall, in this study, we compared U937 EVs and U937 CDNs in terms of physical characterisation, biochemical composition, and pharmacological activity. The size and zeta potential of the nanovesicles were measured. Proteomic, lipidomic, and miRNA profiling were also conducted to compare the biochemical compositions of the nanovesicles. To test whether the nanovesicles modulated inflammation, nitric oxide and inflammatory cytokine concentrations were analysed after having subjected the macrophages to EVs or CDNs. Antioxidant activities such as the catalase and glutathione S-transferase activities of the nanovesicles were also quantified. Lastly, the immunological responses of the nanovesicles upon intravenous administration were also analysed by evaluating the changes in the immune cell populations in the blood and spleen. Overall, U937 CDNs demonstrated similar properties to those of U937 EVs, rendering them a more scalable alternative for therapeutic purposes.

## 2. Materials and Methods

### 2.1. Materials

Spin cups were purchased from ThermoFisher Scientific, and were supplied with 10 µm filters attached; hydrophilic 8 µm membrane filters were purchased from Merck Millipore and used as supplied. Protease inhibitor cocktail ab201111 was purchased from Abcam. Sephadex G-50 was purchased from Sigma Aldrich, and equilibrated in phosphate-buffered saline (PBS). The 0.22 µm filters were purchased from Merck Millipore.

Lipopolysaccharide (LPS) from *Escherichia coli* O111:B4 was purchased from Sigma Aldrich. Measure-iT^TM^ High-Sensitivity Nitrite Assay Kit (M36051) was purchased from ThermoFisher Scientific. Biolegend Mouse ELISA Max^TM^ Deluxe (TNF-α, IL-1β, IL-6 and IL-10) were purchased from BioLegend. Total Antioxidant Capacity (TAC) assay kit (ab65329), Catalase Activity assay kit (ab83464) and GST assay kit (ab65326) were purchased from Abcam.

### 2.2. Cell Culture

U937 monocytes were a kind gift from A/P Gigi Chiu (NUS). U937 monocytes were grown in RPMI-1640 cell culture medium supplemented with 10% fetal bovine serum (FBS), HyClone, and RAW264.7 macrophages were grown in DMEM cell culture medium supplemented with 10% FBS. All cells were maintained in a 5% CO_2_ incubator at 37 °C.

### 2.3. Animal Work

Female 4-week-old BALB/cAnNTac mice were purchased from InVivos, Singapore. All experimental procedures involving the use of animals were approved by the Office of Institutional Animal Care and Use Committee (IACUC), National University of Singapore (protocol number: R2021-0034), and were in accordance with the Guidelines on the Care and Use of Animals for Scientific Purposes by the National Advisory Committee for Laboratory Animal Research (NACLAR), and the Guide for the Care and Use of Laboratory Animals by the US National Institutes of Health. Animals were acclimatised for 3 days and kept in room temperature (24 ± 2 °C), a 12 h light/dark cycle, and relative humidity of 30–70% throughout the study, and they were provided ready access to food and water.

### 2.4. Isolation of U937 Extracellular Vesicles (EVs)

**Figure 1:**
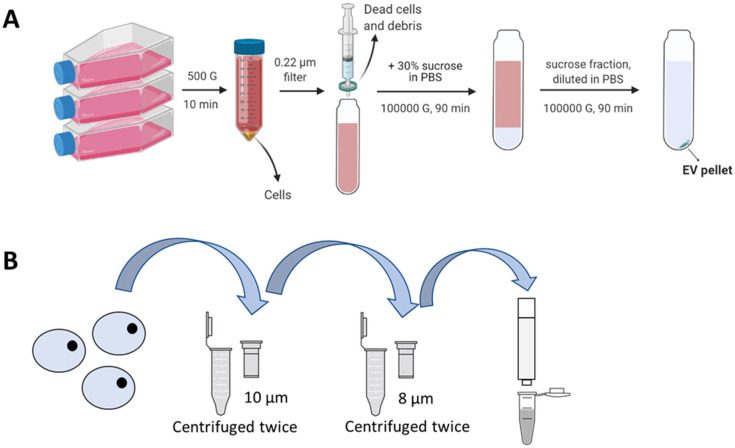
(**A**) Isolation of U937 EVs with sucrose cushion differential ultracentrifugation. The isolated EV pellet was resuspended in PBS. (**B**) Production of U937 CDNs by shearing cells through spin cups fitted with membrane filters. Spin cups were centrifuged at 14,000× *g* for 10 min at 4 °C. The resultant product was purified using a Sephadex G-50 size exclusion column. Image created using BioRender.

U937 monocytes were transferred into a 10% exosome-depleted FBS supplemented RPMI-1640 cell culture medium for 2 days. EVs were isolated with sucrose cushion differential ultracentrifugation described by Théry et al. with slight modifications [13]. Briefly, the conditioned medium was first centrifuged at 500× *g* for 10 min at 4 °C to remove the cells. The supernatant was then passed through a 0.22 µm membrane filter to remove dead cells and cellular debris. Then, 30% sucrose in PBS solution was added to the resultant supernatant and centrifuged at 100,000× *g* for 90 min at 4 °C. The sucrose fraction was collected and diluted with PBS before a final centrifugation at 100,000× *g* for 90 min at 4 °C. The obtained EV pellet was resuspended in PBS. Figure 1A shows a graphical description of the EV isolation process. EVs were filtered with a 0.22 µm filter and stored at −80 °C until used.

### 2.5. Production of U937 Cell-Derived Nanovesicles (CDNs)

CDNs were produced with the method described by Goh et al. [18]. Briefly, U937 monocytes were cultured in 10% FBS supplemented RPMI-1640 medium till 70% confluence. The cells were centrifuged at 500× *g* for 10 min, and washed twice using PBS to remove cellular debris and culture medium. The cells were then resuspended in PBS supplemented with a protease inhibitor cocktail (EDTA-free) (ab201111) (Abcam) to a concentration of 2 × 10^7^ cells/mL. The resultant cell suspension was placed in a spin cup fitted with a 10 µm membrane filter and centrifuged at 14,000× *g* for 10 min at 4 °C. The flowthrough was reintroduced into the spin cup, and the process was repeated. The flowthrough was then added to another spin cup fitted with two 8 µm membrane filters and centrifuged twice at 14,000× *g* for 10 min at 4 °C. The resultant product was then purified using a Sephadex G-50 size exclusion column equilibrated in PBS. The fraction corresponding to the highest protein concentration and the appropriate hydrodynamic diameters (i.e., the second fraction) was collected. Figure 1B shows a graphical description of the CDN production process. CDNs were filtered with a 0.22 µm filter and stored at −80 °C until use.

### 2.6. Physical Characterisation of EVs and CDNs

The protein concentrations were determined using Pierce^TM^ BCA Protein Assay Kit (ThermoFisher Scientific, Waltham, MA, USA). Absorbance readings at 562 nm were determined with a Hidex Sense Microplate Reader (Hidex, Turku, Finland). The hydrodynamic diameters and particle concentrations were determined via dynamic light scattering using the Zetasizer Ultra (Malvern Instruments, Malvern, UK). The zeta potentials were also determined with the Zetasizer Ultra (Malvern Instruments, Malvern, UK).

### 2.7. Transmission Electron Microscopy Imaging

U937 EVs or U937 CDNs were fixed using glutaraldehyde (final concentration: 2.5%) and stored at 4 °C until imaged. The samples had been dropped onto a glow-discharged formvar-coated 200-mesh copper grid (Electron Microscopy Sciences, PA, USA) for 5 min before the excess sample fluid was removed with a filter paper. Samples were then contrasted using 1% phosphotungstic acid (PTA) for 1 min. The negatively stained vesicles were imaged using a JEM-1400 Flash Electron Microscope (Jeol) with an accelerating voltage of 100 kV. Transmission electron microscopy images were recorded using the built-in Matataki Flash camera.

### 2.8. Proteomic Analysis

U937 EVs or CDNs were subjected to three freeze–thaw cycles in liquid nitrogen for 10 min, and centrifuged at 17,000× *g* for 10 min at 25 °C. Samples were processed using an S-Trap mini column (Protifi) according to the manufacturer’s instructions. Eluted peptides were dried using a vacuum evaporator and reconstituted in 2% acetonitrile with 0.1% formic acid for liquid chromatography/mass spectrometry (LC/MS) analysis. The peptides were separated using an Eksigent ChromXP C18-CL trap column (3 µm, 120 Å, 200 µm × 0.5 mm) and Eksigent ChromXP C18-CL analytical column (3 µm, 120 Å, 75 µm × 150 mm) in an Eksigent NanoLC-Ultra with a cHiPLC–Nanoflex system. The used solvents as Mobile Phase A were 2% acetonitrile with 0.1% formic acid, and 98% acetonitrile with 0.1% formic acid as Mobile Phase B. The eluted peptides were then analysed using an SCIEX TripleTOF 5600. The data were subjected to an MSMS spectral search using ProteinPilot 5.0 (SCIEX) and Mascot Server 2.7 (Matrix Science) with the Human SwissProt Reference Proteome (2019 Dec release) spiked with common contaminant proteins (cRAP). Proteomic analysis was conducted in the Protein and Proteomics Centre, National University of Singapore.

### 2.9. Lipidomic Analysis

U937 EVs or CDNs were diluted using 150 mM ammonium bicarbonate (NH_4_HCO_3_) solution and mixed with methyl tert-butyl ether/methanol (7:2, *v*/*v*) solution containing internal standards. The samples were then vortexed for 2 min before sonication for 30 min at 4 °C. The resultant sample were centrifuged at 3000× *g* for 5 min at 4 °C to facilitate the phase separation. The organic layer was dried using a SpeedVac vacuum concentrator (ThermoFisher Scientific), and reconstituted in butanol/methanol (1:1, *v*/*v*) for LC/MS analysis.

The samples were separated and analysed using an Agilent 1290 Infinity II LC coupled to 6495 C Triple Quadrupole LC/MS system (Agilent Technologies, Santa Clara, CA, USA). A Zorbax Eclipse Plus C18 column (2.1 × 50 mm, 1.8 µm) was used for the separation. The used solvents were 40% acetonitrile in water with 10 mM ammonium formate as Mobile Phase A, and 10% acetonitrile in isopropanol with 10 mM ammonium formate as Mobile Phase B. The internal standards used for lipidomic analysis were: acylcarnitine 16:0 d3, cardiolipin 56:0, CE 18:0 d6, Cer d18:0/08:0, Cer d18:1/12:0, Cer m18:1/12:0, DG 30:0, GM3 d18:1/18:0 d3, Hex1Cer d18:1/12:0, Hex2Cer d18:1/12:0, Hex3Cer d18:1/18:0 d3, LPC 13:0, LPE 14:0, PC 26:0, PC-P 36:1 d9, PE 34:0, PE-P 18:0/18:1, PG 34:0, PI 25:0, PS 34:0, SM d18:1/12:0, and TG 36:0. The quantification data were extracted using Aligent MassHunter Quantitative Analysis software. Lipidomic analysis was conducted at the Singapore Lipidomics Incubator, National University of Singapore.

### 2.10. miRNA Profiling

U937 EVs were resuspended in tris-buffered saline (TBS) after the final ultracentrifugation step. A buffer exchange to TBS was conducted on U937 CDNs during the size exclusion step. U937 cells had been washed twice with PBS before the cell pellet was frozen using liquid N_2_. miRNAs were extracted using a Maxwell RSC miRNA tissue kit (Promega, Madison, WI, USA) according to manufacturer’s instructions with RNA spike-in controls added for workflow quality control. miRNAs were profiled using an ID3EAL miRNA Knowledge Panel kit (MiRXES, Singapore) according to the manufacturer’s instructions. Real-time polymerase chain reaction was performed using a QuantStudio Real-Time PCR System (Applied BioSystems, Waltham, MA, USA). Threshold cycle values were determined using QuantStudio Design and Analysis software, and normalised to the global miRNA expression. miRNAs were profiled at the NUSMed noncoding RNA Core Facility, National University of Singapore.

### 2.11. Nitric Oxide Assay

We plated 5 × 10^4^ RAW264.7 macrophages on a 96-well plate and incubated them overnight at 37 °C for adhesion. Then, 10 ng/mL LPS was added to simulate inflammation. At 2 h after the stimulation with LPS, EVs or CDNs were added (final concentration: 5 and 25 µg/mL of proteins) and incubated for 24 h. The cell culture supernatant was then collected and analysed for the nitrite concentration using Measure-iT^TM^ High-Sensitivity Nitrite Assay Kit (M36051) (ThermoFisher Scientific). Fluorescence intensity at 355/444 nm was measured using a Hidex-Sense Microplate Reader (Hidex).

### 2.12. Inflammatory Cytokines Production

We plated 1 × 10^6^ RAW264.7 macrophages on a 6-well plate and incubated them overnight at 37 °C for adhesion. We then added 10 ng/mL LPS to simulate inflammation. At 2 h after simulation with LPS, EVs or CDNs were added (final concentration: 5 and 25 µg/mL of proteins) and incubated for 24 h. The cell culture supernatant was then collected and centrifuged at 3000× *g* for 10 min at 4 °C to remove the cells. The resultant supernatant was stored at −80 °C until further analysis. The concentrations of inflammatory cytokines TNF-α, IL-1β, IL-6 and IL-10 were quantified with ELISA using Biolegend Mouse TNF-α ELISA Max^TM^ Deluxe, Biolegend Mouse IL-1β ELISA Max^TM^ Deluxe, Biolegend Mouse IL-6 ELISA Max^TM^ Deluxe and Biolegend Mouse IL-10 ELISA Max^TM^ Deluxe (Biolegend, CA, USA), respectively, according to the manufacturer’s instructions.

### 2.13. Antioxidant Levels

The total antioxidant capacity (TAC) of U937 EVs and CDNs was quantified using a Total Antioxidant Capacity Assay Kit (ab65329) (Abcam, Cambridge, UK) according to the manufacturer’s instructions, with Trolox as the reference standard. The activity of antioxidant enzymes catalase and glutathione-S-transferase was quantified using a Catalase Activity Assay Kit (ab83464) and GST Assay Kit (ab65326) (Abcam), respectively, according to the manufacturer’s instructions.

### 2.14. In Vivo Immunological Profiling

We administered 40 µg proteins of U937 EVs or 40 µg proteins of U937 CDNs or 100 µL saline intravenously to Balb/cAnNTac mice (InVivos, Singapore) via the tail vein (protocol number: R2021-0034). After 24 h of drug administration, blood was collected via cardiac puncture, and the spleen was harvested. Cells in the blood and spleen were isolated and stained for surface markers with primary antibodies for 30 min on ice, followed by washing with staining buffer twice. The cells were then fixed using a BD Cytofix/Cytoperm^TM^ Fixation/Permeabilisation solution for 20 min on ice, followed by staining for intracellular markers (iNOS and CD206) with primary antibodies for 30 min on ice. The stained cells were then washed twice using a staining buffer and analysed with flow cytometry using BD LSRFortessa (BD BioSciences, East Rutherford, NJ, USA).

### 2.15. Statistical Analysis

The data are presented as the mean with the standard error of the mean (SEM) as the error bar. Statistical analysis was performed using GraphPad Prism 8 (GraphPad Software). When comparing two groups, Student’s *t*-test and two-way ANOVA, followed by Sidak’s multiple-comparisons post hoc test, were used for the parametric data, and the Mann–Whitney U-test for nonparametric data. A *p* value < 0.05 was considered significant.

## 3. Results

### 3.1. Physical Characterisation of U937 EVs and CDNs

**Figure 2:**
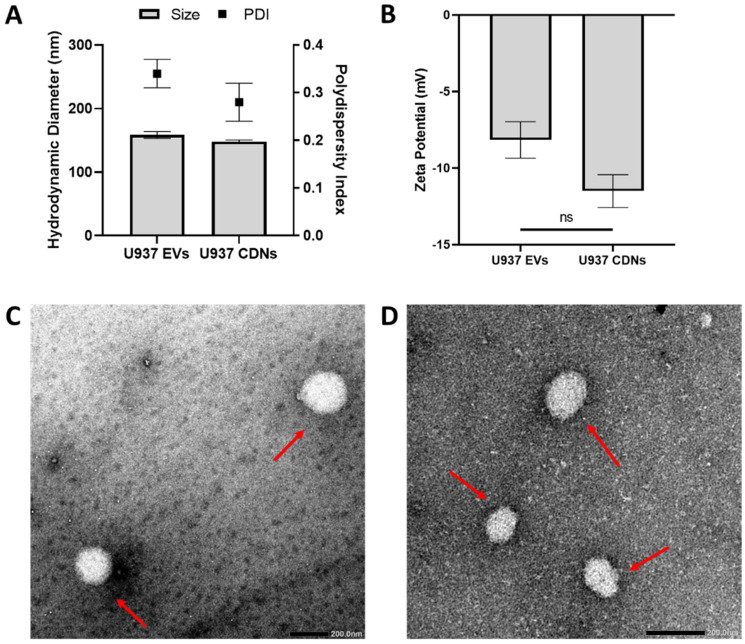
Physical properties of U937 EVs and U937 CDNs. (**A**) Hydrodynamic diameters and polydispersity index of U937 EVs and U937 CDNs; *n* = 3. Data presented as mean ± SEM. (**B**) Zeta potentials of U937 EVs and U937 CDNs; *n* = 3. Data presented as mean ± SEM. Transmission electron microscopy images of (**C**) U937 EVs and (**D**) U937 CDNs. The red arrow indicates nanovesicles. The scale bar is 200 nm.

The hydrodynamic diameters of U937 EVs and CDNs were analysed with dynamic light scattering. U937 EVs had an average diameter of 158.5 ± 5.4 nm, while U937 CDNs showed a size of 148.0 ± 2.5 nm (Figure 2A). The polydispersity index, which measured the homogeneity of the U937 EVs and U937 CDNs, was around 0.3 (Figure 2A). This was expected, as biological samples are more heterogeneous. The zeta potentials of U937 EVs and CDNs were −8.16 ± 1.19 and −11.80 ± 0.70 mV, respectively (Figure 2B). This confirmed that the CDNs produced via spin cups had similar physical properties to those of EVs.

In terms of yields, we quantified the protein content of the isolated EVs and produced CDNs. We obtained an isolation yield of 15.4 ± 1.3 µg protein of EVs from 2 × 10^7^ cells. The isolation yield for the EVs was lower than what was previously reported [18], possibly due to the addition of a 30% sucrose cushion purification step to remove contaminants. We produced 586.8 ± 33.2 µg protein of CDNs from 2 × 10^7^ cells, which was approximately 40 times more than the isolation yield of EVs. Moreover, the time required to produce CDNs (3 to 4 days) was shorter than the duration required to isolate EVs (6 to 7 days). This confirmed that the production of CDNs is more efficient than the isolation of EVs.

To evaluate the morphology of the nanovesicles, transmission electron microscopy (TEM) imaging was conducted. The nanovesicles were negatively stained using 1% PTA before imaging. Figure 2C,D show a representative TEM image of U937 EVs and CDNs.

The morphology of U937 EVs and CDNs was similar, and both nanovesicle types were spherical. Overall, U937 CDNs had similar physical properties to those of U937 EVs.

### 3.2. Biochemical Composition of U937 EVs and CDNs

**Figure 3:**
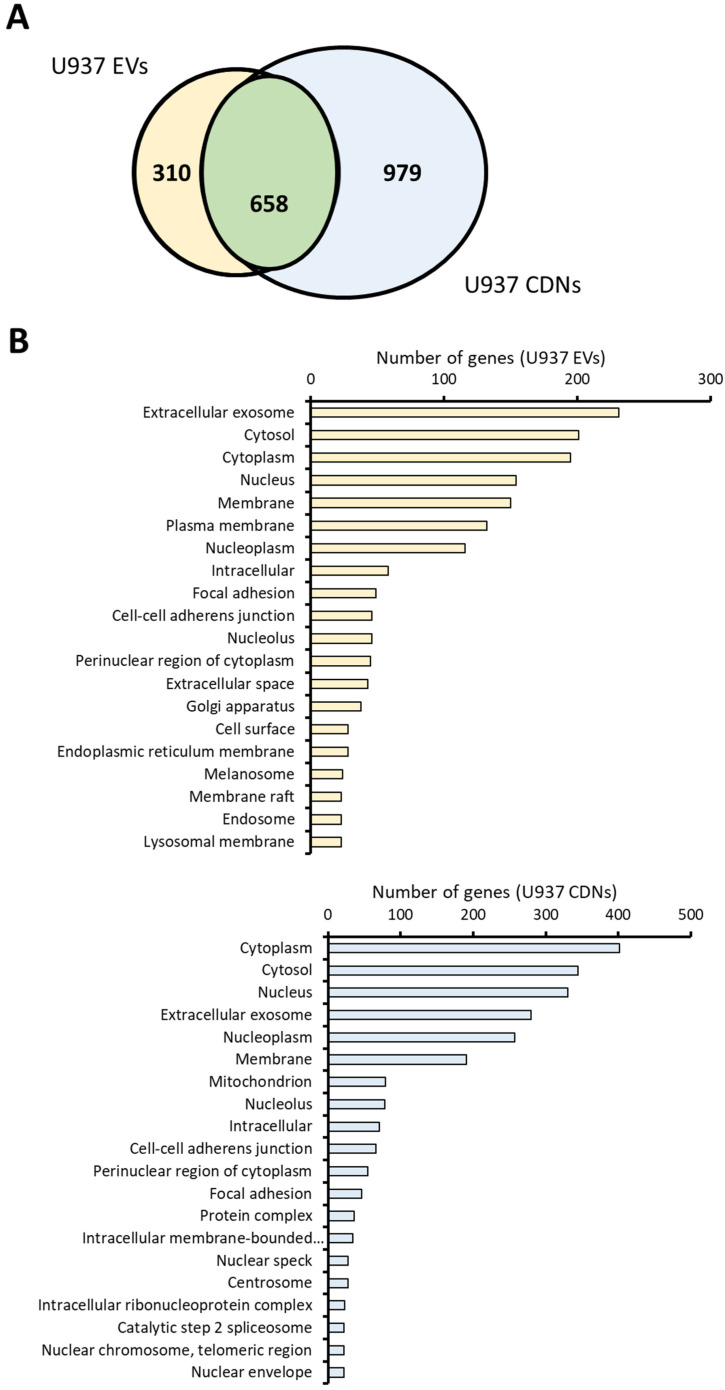

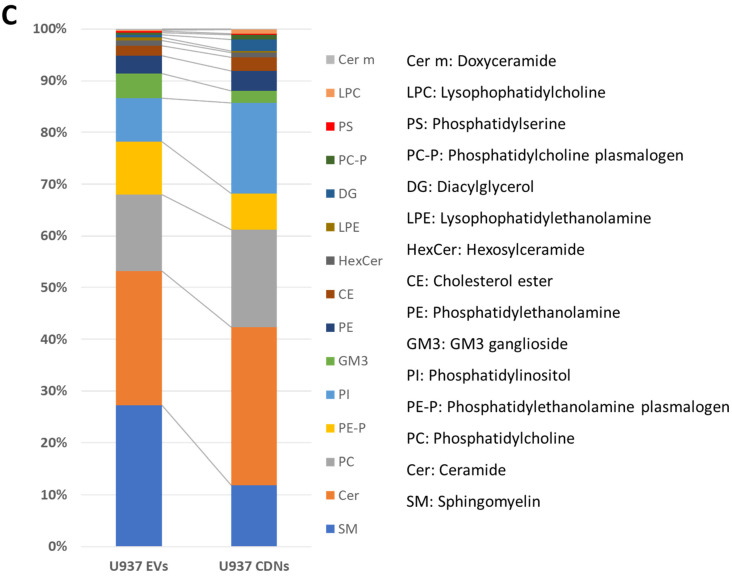

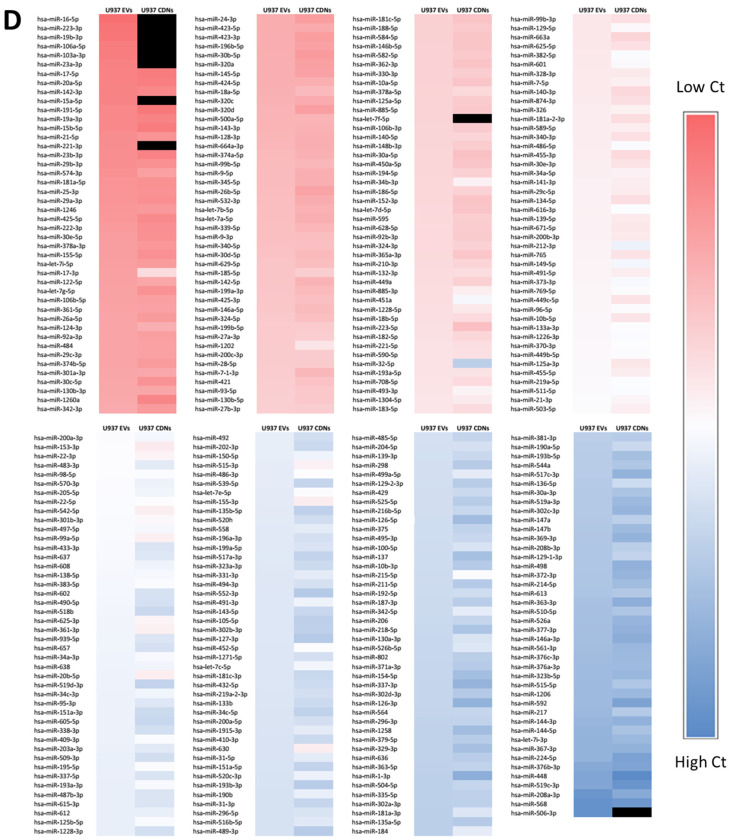
Comparison of the biochemical composition of U937 EVs and U937 CDNs. (**A**) Venn diagram comparing the protein profiles of U937 EVs and U937 CDNs. (**B**) Gene ontology of U937 EVs and U937 CDNs with respect to the cellular component. (**C**) Comparison of the lipid profiles of U937 EVs and U937 CDNs; *n* = 3. (**D**) Heat map comparing the miRNA profiles of (left) U937 EVs and (right) U937 CDNs; *n* = 2. Red indicates high abundance (low Ct values), blue indicates low abundance (high Ct values), and black indicates a lack of detection in at least one of the two replicates.

After comparing the physical properties of U937 EVs and U937 CDNs, their biochemical composition was assessed. Proteomic, lipidomic, and miRNA profiling for U937 EVs and U937 CDNs was performed in triplicate and compared as shown in Figure 3.

From the proteomic analysis, 968 proteins were detected in U937 EVs compared to 1637 proteins in U937 CDNs. The list of identified proteins in the U937 EVs and U937 CDNs is provided in the Supplementary Table S1. Among these proteins, 658 (i.e., 67.9% of the proteins found in U937 EVs) were common between U937 EVs and U937 CDNs (Figure 3A). Moreover, 89 of the proteins detected in U937 EVs and 73 of the proteins detected in U937 CDNs were among the top 100 EV marker proteins in the ExoCarta database [30]. As the biogenesis of EVs is highly regulated, the proteins found in EVs were expected to be fewer than those in CDNs. Unlike EVs, the production of CDNs involves the whole cell, and other cellular proteins may be encapsulated during the shearing process, thus justifying the greater protein number in CDNs.

To predict and compare the subcellular localisation of identified proteins in U937 EVs and U937 CDNs, we performed gene ontology (GO) analysis with respect to the cellular components. GO is a knowledge base that is used to perform enrichment analysis on gene sets [31]. On the basis of the number of associated genes, the top 20 highly enriched GO (cellular component) terms were selected and are presented in Figure 3B. Interestingly, the top cellular components for U937 EVs and U937 CDNs were in common, including proteins usually present in extracellular exosomes, the cytosol, cytoplasm, and nucleus. This GO analysis demonstrates that U937 CDNs mimicked U937 EVs in the localisation of these proteins.

We also conducted lipidomic analysis on U937 EVs and CDNs. The lipid profiles of these nanovesicles affect the fluidity, deformability, stability, and potential leakiness of the vesicles [32]. The top 5 most abundant lipid classes found in U937 EVs and CDNs were sphingomyelin (SM), ceramide (Cer), phosphatidylcholine (PC), phosphatidylethanolamine plasmalogen (PE-P), and phosphatidylinositol (PI) (Figure 3C). These classes of lipids correspond to around 85% of the total lipid composition in U937 EVs and U937 CDNs. Cer (*p* = 0.0584) and PI (*p* < 0.001) were enriched in U937 CDNs, while SM (*p* < 0.001) was enriched in U937 EVs (Supplementary Figure S1). SM had high affinity for membrane cholesterol, which could reduce membrane fluidity, while Cer, with its inverted cone shape, could induce a negative curvature of the membrane [33]. These lipids affect the overall membrane structure, and similarities in the proportion of these lipids imply that EVs and CDNs would have similar membrane structure and properties (such as membrane permeability and fluidity). Moreover, both nanovesicles contained low levels of phosphatidylserine (PS) (0.32% in U937 EVs and 0.22% in U937 CDNs) as compared to the parent U937 cells [18]. A low proportion of PS could possibly reduce clearance with the mononuclear phagocyte system, thereby increasing circulation time in vivo [34,35].

Besides proteins and lipids, we also investigated the miRNA profiles in U937 EVs and U937 CDNs, which were similar (Figure 3D). Most miRNAs (97.1% of miRNAs found in U937 EVs) were also present in the U937 CDNs. The abundance of the miRNAs was comparable, as indicated by the analogous colour shade in the heat map. Interestingly, U937 CDNs contained fewer miRNAs than U937 EVs did, and most of the missing miRNAs were highly abundant in U937 EVs. Some of the undetected miRNA in U937 CDNs, which were highly abundant in U937 EVs, are hsa-miR-16-5p, hsa-miR-223-3p, hsa-miR-19-3p, hsa-miR-106a-5p, and hsa-miR-103-3p. Among these, hsa-miR-16-5p and hsa-miR-223-3p were not detected in all U937 CDNs replicates as compared to U937 EVs. This implies that there were some differences between EVs and CDNs. We also profiled the miRNAs of U937 cells and compared the threshold cycle (Ct) values between U937 cell pellets (CPs) and U937 CDNs. miRNA expression in CDNs is highly correlated to the miRNA expression in parental cells (r^2^ = 0.837) (Supplementary Figure S2). This implies that miRNA expression in CDNs could be altered by modifying the miRNA expression in parental cells.

### 3.3. Pharmacological Activity of U937 EVs and U937 CDNs on Macrophages

Nitric oxide (NO) is an intracellular messenger produced by cells that is involved in many physiological processes such as neuronal signalling and cardiovascular homeostasis [36,37]. NO can also be generated by phagocytes (including macrophages) during inflammation [38,39]. NO has a short half-life, in the order of milliseconds, and is broken down into nitrite. As such, we detected the nitrite concentration in the cell culture supernatant as a proxy to the NO concentration [40].

**Figure 4:**
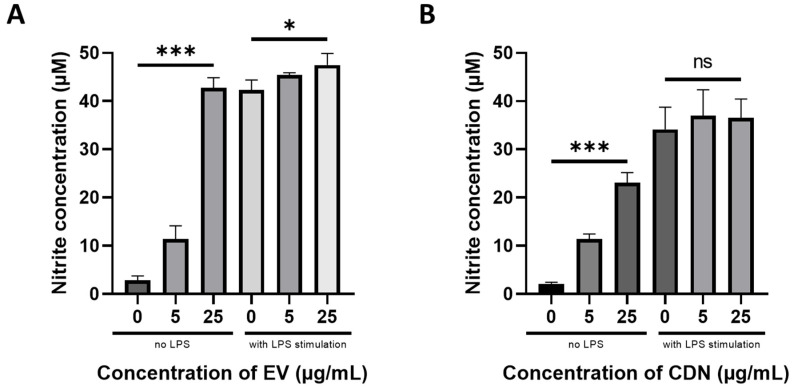

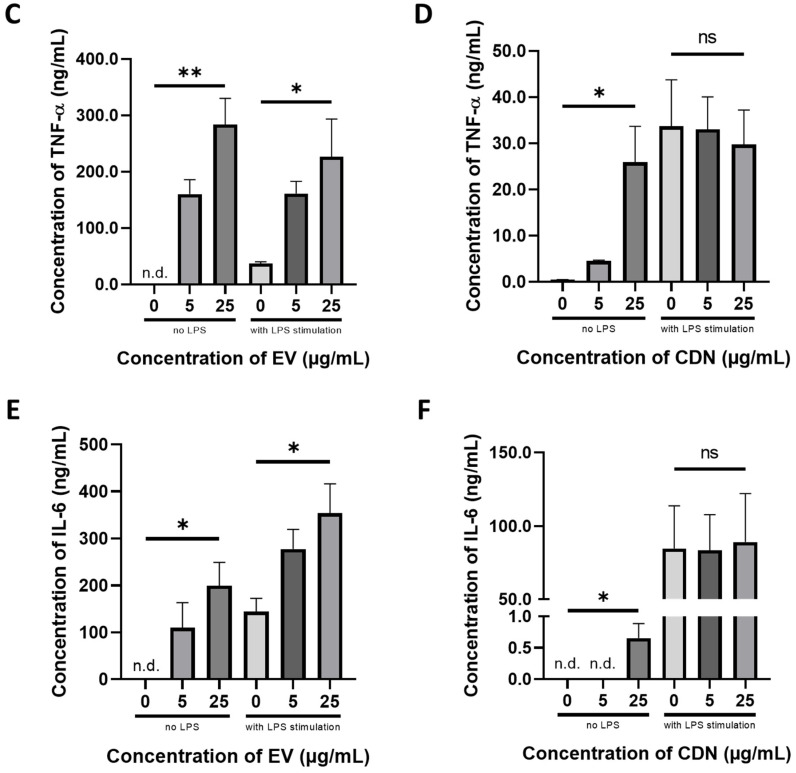
Nitrite concentrations in cell culture supernatant after RAW264.7 macrophages had been treated with increasing concentrations of (**A**) U937 EVs and (**B**) CDNs for 24 h. TNF-α concentrations in cell culture supernatant after RAW264.7 macrophages were treated with increasing concentrations of (**C**) U937 EVs and (**D**) CDNs for 24 h. IL-6 concentrations in the cell culture supernatant after RAW264.7 macrophages were treated with increasing concentrations of (**E**) U937 EVs and (**F**) CDNs for 24 h. IL-1β and IL-10 concentrations were below the detection limit of the ELISA kits (<31.3 pg/mL). The LPS concentration was 10 ng/mL. Data are presented as mean ± SEM (*n* = 3). n.s., no statistical difference; *, *p* < 0.05, **, *p* < 0.01; ***, *p* < 0.001.

We tested the EVs and CDNs on two different types of macrophages: resting and activated. The activated macrophages were stimulated with 10 ng/mL LPS prior to treatment with EVs or CDNs. Figure 4A,B show the nitrite concentrations in the cell culture supernatant after treatment with U937 EVs and U937 CDNs, respectively. U937 EVs and U937 CDNs stimulated the production of NO in the resting macrophages within 24 h. This shows that the U937 EVs and CDNs may be proinflammatory. Interestingly, unlike U937 EVs, U937 CDNs did not further stimulate the production of NO in activated macrophages. This suggests that U937 CDNs do not further aggravate inflammation. The doses (5 versus 25 µg/mL) did not influence this profile, indicating that the effect was not dose-dependent.

We then investigated the inflammatory cytokines secreted by RAW264.7 macrophages after the treatment of U937 EVs and U937 CDNs. We conducted ELISA assays for key proinflammatory cytokines (TNF-α, IL-6 and IL-1β) and anti-inflammatory cytokines (e.g., IL-10). Figure 4C–F show the concentrations of TNF-α and IL-6 in the cell culture supernatant after treating the RAW264.7 macrophages with U937 EVs or U937 CDNs. The concentrations of IL-1β and IL-10 were below the detection limit (<31.3 pg/mL) of the used ELISA kits. Both U937 EVs and U937 CDNs stimulated the production of proinflammatory cytokines TNF-α and IL-6 in the resting macrophages. This supports reports from the literature that monocyte-derived EVs could exhibit proinflammatory effects [23,41]. Similar to the observed trend for NO production, U937 CDNs did not further stimulate the production of proinflammatory cytokines in the activated macrophages. As anti-inflammatory cytokine IL-10 was not detected in all samples, U937 EVs and U937 CDNs might not exhibit anti-inflammatory activity.

### 3.4. Antioxidant Levels and Enzyme Activities

**Figure 5:**
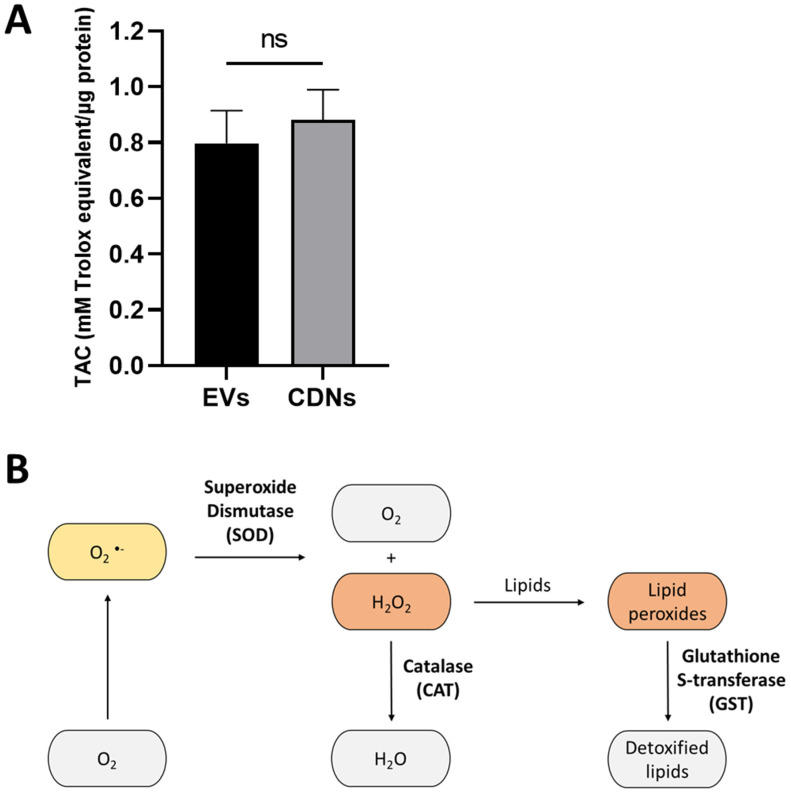

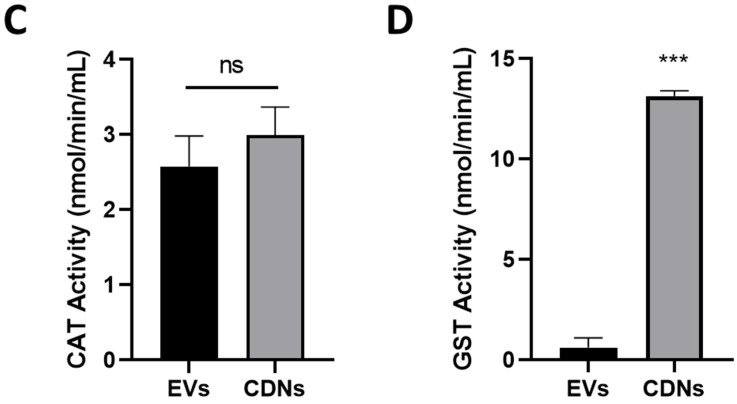
(**A**) Total antioxidant capacity of U937 EVs and U937 CDNs. Data presented as mean ± SEM (*n* = 5). (**B**) Detoxification of ROS by cellular antioxidant enzymes. In the first line of defence, antioxidant enzymes such as SOD and CAT can convert ROS into oxygen and water. In the third line of defence, antioxidant enzymes such as GST could detoxify oxidised biomolecules and repair damage caused by free radicals. (**C**) Catalase activity of U937 EVs and U937 CDNs. Data presented as mean ± SEM (*n* = 4). (**D**) Glutathione S-transferase activity of U937 EVs and U937 CDNs. Data presented as mean ± SEM (*n* = 3). ns, no statistical difference; ***, *p* < 0.001.

Besides investigating the inflammatory activities of U937 EVs and U937 CDNs, we also examined the antioxidant activities of the nanovesicles. First, we measured the total antioxidant capacity (TAC) of the nanovesicles. Figure 5A shows the TAC level of U937 EVs and CDNs. U937 EVs and U937 CDNs had a similar TAC at around 0.8 mM Trolox equivalent/µg protein.

After demonstrating that U937 EVs and CDNs had similar antioxidant capacities, we analysed whether these nanovesicles also had antioxidant enzymatic activities. Antioxidant enzymes contribute to overall antioxidant activity through two main roles: as preventive antioxidants, and as repair and de novo antioxidants [42,43].

As preventive antioxidants, antioxidant enzymes could detoxify reactive oxygen species (ROS) such as superoxide (O_2_^−^) and hydrogen peroxide (H_2_O_2_) (Figure 5B). Superoxide has a short half-life and could spontaneously undergo disproportionation to form hydrogen peroxide and oxygen. This reaction could also be catalysed by superoxide-scavenging enzyme superoxide dismutase (SOD). Subsequently, the detoxification of hydrogen peroxide into water can be catalysed by catalase (CAT) or glutathione peroxidase (GPx).

Moreover, repair and de novo enzymes could repair the oxidative damage caused by free radicals on cellular proteins, lipids, and DNA. A form of such oxidative damage is lipid peroxidation, which affects the structural and functional integrity of the cell membrane [44]. To reverse the damage, enzymes such as glutathione S-transferase (GST) could catalyse the detoxification of lipid peroxides into less-toxic products (Figure 5B).

For the comparative study, we investigated the activity of catalase and glutathione S-transferase as representative enzymes of different antioxidant mechanisms. Figure 5C,D show the CAT and GST activities in U937 EVs and CDNs. Although CAT activity was relatively similar in U937 EVs and CDNs, U937 CDNs exhibited higher GST activity (*p* < 0.001). This implies that U937 CDNs may be able to repair oxidative damage on biomolecules. CDNs may be a potential treatment for diseases caused by high oxidative stress.

### 3.5. In Vivo Immune Cell Profiling

**Figure 6:**
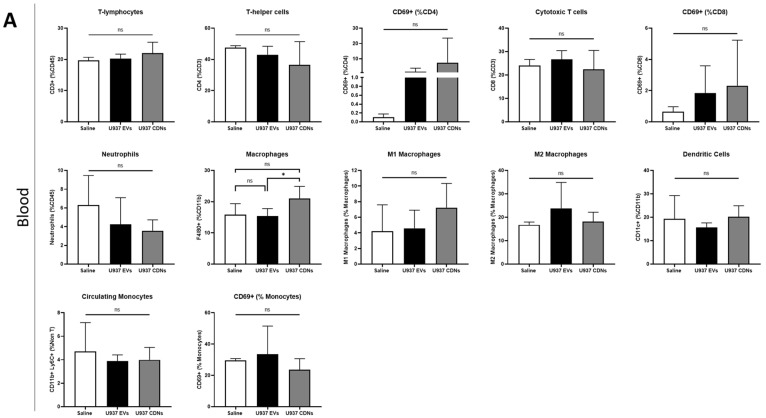

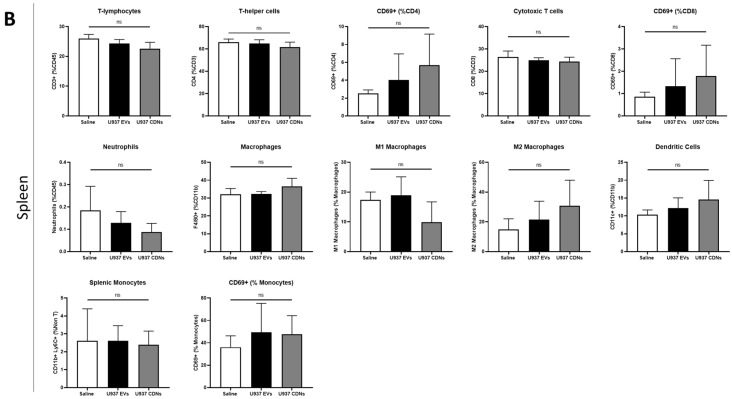
Immune cell profiles of (**A**) blood and (**B**) spleen after intravenous administration of saline, U937 EVs or U937 CDNs. Data presented as mean ± SEM (*n* = 4 for saline, *n* = 5 for U937 EVs and CDNs). ns indicates no statistical difference, * indicates *p* < 0.05.

On top of comparing the pharmacological activities between U937 EVs and U937 CDNs, we also evaluated the immunogenicity of these nanovesicles in comparison to the normal saline. U937 EVs or U937 CDNs or saline were administered to Balb/cAnNTac mice intravenously via tail vein. The blood was collected, and the spleen was harvested 24 h after the injection. The spleen is involved in immunity regulation and hosts a pool of immune cells [45,46,47]. Immunophenotyping of the blood and spleen was performed and presented in Figure 6A and Figure 6B, respectively.

There were no significant differences in the immune cell profiles of the blood and spleen after the administration of U937 EVs or U937 CDNs in comparison to the saline control. There were no significant changes observed in the polarisation of macrophages to either the proinflammatory M1 or anti-inflammatory M2 phenotype. Likewise, there was no significant increase in CD69+ expression levels in T-helper cells, cytotoxic T cells, and monocytes, implying that there was no significant activation of immune cells [48,49]. The immune cell profiles signify that these nanovesicles do not exert significant immune responses when injected intravenously in vivo.

Comparing the immunological profiles of the blood and spleen of U937 EVs and U937 CDNs, there were no significant differences, with the sole exception of the percentage of macrophages found in the blood (*p* = 0.027). Despite an increase in blood macrophages when U937 CDNs were administered, there was no significant change in the polarisation of blood macrophages. The immune cell profiles of both blood and spleen show that U937 CDNs exerted similar immunological responses to those of U937 EVs when administered in vivo.

## 4. Conclusions

We compared the physical, biochemical, and pharmacological activities of U937 EVs and U937 CDNs. As EV mimetics, CDNs have a similar hydrodynamic diameter, zeta potential, and morphology. Proteomic, lipidomic, and miRNA profiling also showed that CDNs had some similarity to EVs in terms of biochemical composition. More than 60% of the proteins found in U937 EVs were detected in U937 CDNs, and these proteins were localised in similar cellular components. U937 CDNs contained more proteins than U937 EVs did. While the top 5 most abundant lipid classes were the same for U937 EVs and U937 CDNs, there were statistical differences in the ceramide and phosphatidylinositol levels. Likewise, although the relative abundance of the miRNAs found in U937 EVs and U937 CDNs were similar, there were miRNAs found only in U937 EVs. While CDNs are mimetics of EVs, the pharmacological activities of CDNs may differ due to the slight differences in physical and biochemical properties.

We investigated the inflammatory properties of U937 EVs and U937 CDNs. The inflammatory properties of U937 EVs and CDNs are summarised in Table 1. U937 EVs and U937 CDNs exhibited proinflammatory activities, stimulating the production of NO, and proinflammatory cytokines TNF-α and IL-6 when added to resting macrophages. Interestingly, U937 CDNs did not further stimulate the production of NO and proinflammatory cytokines in activated macrophages. This implies that U937 CDNs do not further aggravate inflammation, which may be beneficial when they are administered as therapeutics to treat diseases. Further studies could investigate the time-dependent stimulation of NO and inflammatory cytokines. For instance, cytokine concentrations could be analysed after incubation for 72 h.

Moreover, U937 EVs and U937 CDNs had similar antioxidant activities. Nonetheless, the contributing factors seemed to differ: while CAT activity was comparable, GST activity differed. This suggests that U937 CDNs may be able to repair the damage caused by oxidative stress. To fully characterise the antioxidant activities of EVs and CDNs, the activities of other antioxidant enzymes should also be analysed. The antioxidant enzymes could include superoxide dismutase and glutathione peroxidase. Moreover, EVs and CDNs could also be tested in in vitro models, such as on hypoxic cells, regarding their potential to reduce intracellular oxidative stress and repair oxidative damage.

Future work could also investigate the mechanism of action and the biomolecule or class of biomolecules responsible for pharmacological activities. This study demonstrated that, while CDNs could potentially be a replacement for EVs for therapeutic purposes, some in vitro and in vivo work is still necessary to validate the activity. It is also important to identify the key components within the EVs that contribute to the therapeutic activity as this allows for the better translation and quality control of production.

The in vivo immune cell profiling of the blood and spleen confirmed that U937 EVs and U937 CDNs do not exert significant immune responses when administered intravenously. U937 EVs and U937 CDNs did not activate the blood and spleen immune cells. Although the administration of U937 CDNs resulted in an increase in blood macrophages as compared to U937 EVs, there was no significant difference in the polarisation of the blood macrophages. To further verify the immunological response of the nanovesicles, cytokine and chemokine levels in the blood plasma could be analysed and evaluated in the future.

Overall, U937 CDNs as EV mimetics had similar pharmacological activities to those of U937 EVs, although the extent of the effect may differ. Likewise, U937 CDNs had similar in vivo immunological profiles to those of U937 EVs. However, there were some differences, probably due to the slight differences in the biochemical composition of the nanovesicles. Future work should further evaluate whether these slight changes have an effect, for example, on the long-term usage of CDNs as therapeutic options for myocardial infarction.

## Figures and Tables

**Table 1:** Summary of the inflammatory properties of U937 EVs and U937 CDNs on resting and LPS-stimulated activated macrophages.

	Resting Macrophages		Activated Macrophages	
	U937 EVs	U937 CDNs	U937 EVs	U937 CDNs
Nitrite (proxy for NO)	↑	↑	↑	n.s.
TNF-α	↑	↑	↑	n.s.
IL-6	↑	↑	↑	n.s.

n.s., statistical difference. ↑, increase in expression.

## Data Availability

Data is contained within the article or Supplementary Materials.

## Supplementary Materials

The following are available online at https://www.mdpi.com/article/10.3390/pharmaceutics15041290/s1. Supplementary Table S1: Proteomics Analysis of U937 EVs and U937 CDNs (95% confidence intervals and 1% false discovery rate). Supplementary Figure S1: Lipidomic Analysis of U937 EVs and U937 CDNs. Lipids are quantified as a percentage of the total lipids. Data presented as mean ± SEM (*n* = 3). *** indicates *p* < 0.001. Supplementary Figure S2: Comparison of mean Ct values between U937 cells (indicated as CP on the *x* axis) and U937 CDNs (indicated as CDNs on the *y* axis). *n* = 2. Correlation coefficient (r^2^) = 0.837. Figure prepared by NUSMed noncoding RNA Core Facility, National University of Singapore.

## References

[1] Théry C., Witwer K.W., Aikawa E., Alcaraz M.J., Anderson J.D., Andriantsitohaina R., Antoniou A., Arab T., Archer F., Atkin-Smith G.K. (2018). Minimal information for studies of extracellular vesicles 2018 (MISEV2018): A position statement of the International Society for Extracellular Vesicles and update of the MISEV2014 guidelines. J. Extracell. Vesicles.

[2] Yáñez-Mó M., Siljander P.R.M., Andreu Z., Zavec A.B., Borràs F.E., Buzas E.I., Buzas K., Casal E., Cappello F., Carvalho J. (2015). Biological properties of extracellular vesicles and their physiological functions. J. Extracell. Vesicles.

[3] Doyle L.M., Wang M.Z. (2019). Overview of Extracellular Vesicles, Their Origin, Composition, Purpose, and Methods for Exosome Isolation and Analysis. Cells.

[4] Zaborowski M.P., Balaj L., Breakefield X.O., Lai C.P. (2015). Extracellular Vesicles: Composition, Biological Relevance, and Methods of Study. BioScience.

[5] van Niel G., D’Angelo G., Raposo G. (2018). Shedding light on the cell biology of extracellular vesicles. Nat. Rev. Mol. Cell Biol..

[6] De Toro J., Herschlik L., Waldner C., Mongini C. (2015). Emerging Roles of Exosomes in Normal and Pathological Conditions: New Insights for Diagnosis and Therapeutic Applications. Front. Immunol..

[7] McConnell M.J. (2018). Extracellular vesicles and immune modulation. Immunol. Cell Biol..

[8] Rak J., Guha A. (2012). Extracellular vesicles–vehicles that spread cancer genes. BioEssays.

[9] Lai R.C., Arslan F., Lee M.M., Sze N.S.K., Choo A., Chen T.S., Salto-Tellez M., Timmers L., Lee C.N., El Oakley R.M. (2010). Exosome secreted by MSC reduces myocardial ischemia/reperfusion injury. Stem Cell Res..

[10] Lai R.C., Chen T.S., Lim S.K. (2011). Mesenchymal stem cell exosome: A novel stem cell-based therapy for cardiovascular disease. Regen. Med..

[11] Vu N.B., Nguyen H.T., Palumbo R., Pellicano R., Fagoonee S., Pham P.V. (2021). Stem cell-derived exosomes for wound healing: Current status and promising directions. Minerva Med..

[12] Ahn S.-H., Ryu S.-W., Choi H., You S., Park J., Choi C. (2022). Manufacturing Therapeutic Exosomes: From Bench to Industry. Mol. Cells.

[13] Théry C., Amigorena S., Raposo G., Clayton A. (2006). Isolation and Characterization of Exosomes from Cell Culture Supernatants and Biological Fluids. Curr. Protoc. Cell Biol..

[14] Jang S.C., Kim O.Y., Yoon C.M., Choi D.-S., Roh T.-Y., Park J., Nilsson J., Lötvall J., Kim Y.-K., Gho Y.S. (2013). Bioinspired Exosome-Mimetic Nanovesicles for Targeted Delivery of Chemotherapeutics to Malignant Tumors. ACS Nano.

[15] Jo W., Jeong D., Kim J., Cho S., Jang S.C., Han C., Kang J.Y., Gho Y.S., Park J. (2014). Microfluidic fabrication of cell-derived nanovesicles as endogenous RNA carriers. Lab A Chip.

[16] Jo W., Kim J., Yoon J., Jeong D., Cho S., Jeong H., Yoon Y.J., Kim S.C., Gho Y.S., Park J. (2014). Large-scale generation of cell-derived nanovesicles. Nanoscale.

[17] Yoon J., Jo W., Jeong D., Kim J., Jeong H., Park J. (2015). Generation of nanovesicles with sliced cellular membrane fragments for exogenous material delivery. Biomaterials.

[18] Goh W.J., Zou S., Ong W.Y., Torta F., Alexandra A.F., Schiffelers R.M., Storm G., Wang J.-W., Czarny B., Pastorin G. (2017). Bioinspired Cell-Derived Nanovesicles versus Exosomes as Drug Delivery Systems: A Cost-Effective Alternative. Sci. Rep..

[19] Neupane Y.R., Handral H.K., Alkaff S.A., Chng W.H., Venkatesan G., Huang C., Lee C.K., Wang J.-W., Sriram G., Dienzo R.A. (2022). Cell-derived nanovesicles from mesenchymal stem cells as extracellular vesicle-mimetics in wound healing. Acta Pharm. Sin. B.

[20] Rock K.L., Kono H. (2008). The inflammatory response to cell death. Annu. Rev. Pathol..

[21] Hwang H.S., Kim H., Han G., Lee J.W., Kim K., Kwon I.C., Yang Y., Kim S.H. (2021). Extracellular Vesicles as Potential Therapeutics for Inflammatory Diseases. Int. J. Mol. Sci..

[22] Teng X., Chen L., Chen W., Yang J., Yang Z., Shen Z. (2015). Mesenchymal Stem Cell-Derived Exosomes Improve the Microenvironment of Infarcted Myocardium Contributing to Angiogenesis and Anti-Inflammation. Cell. Physiol. Biochem..

[23] Hezel M.E.V., Nieuwland R., Bruggen R.V., Juffermans N.P. (2017). The Ability of Extracellular Vesicles to Induce a Pro-Inflammatory Host Response. Int. J. Mol. Sci..

[24] Arslan F., Lai R.C., Smeets M.B., Akeroyd L., Choo A., Aguor E.N.E., Timmers L., van Rijen H.V., Doevendans P.A., Pasterkamp G. (2013). Mesenchymal stem cell-derived exosomes increase ATP levels, decrease oxidative stress and activate PI3K/Akt pathway to enhance myocardial viability and prevent adverse remodeling after myocardial ischemia/reperfusion injury. Stem Cell Res..

[25] Ugel S., Canè S., De Sanctis F., Bronte V. (2021). Monocytes in the Tumor Microenvironment. Annu. Rev. Pathol. Mech. Dis..

[26] Mentkowski K.I., Euscher L.M., Patel A., Alevriadou B.R., Lang J.K. (2020). Monocyte recruitment and fate specification after myocardial infarction. Am. J. Physiol. Cell Physiol..

[27] Parihar A., Eubank T.D., Doseff A.I. (2010). Monocytes and macrophages regulate immunity through dynamic networks of survival and cell death. J. Innate Immun..

[28] Ogle M.E., Segar C.E., Sridhar S., Botchwey E.A. (2016). Monocytes and macrophages in tissue repair: Implications for immunoregenerative biomaterial design. Exp. Biol. Med..

[29] Haque S., Sinha N., Ranjit S., Midde N.M., Kashanchi F., Kumar S. (2017). Monocyte-derived exosomes upon exposure to cigarette smoke condensate alter their characteristics and show protective effect against cytotoxicity and HIV-1 replication. Sci. Rep..

[30] Keerthikumar S., Chisanga D., Ariyaratne D., Al Saffar H., Anand S., Zhao K., Samuel M., Pathan M., Jois M., Chilamkurti N. (2016). ExoCarta: A Web-Based Compendium of Exosomal Cargo. J. Mol. Biol..

[31] Haraszti R.A., Didiot M.-C., Sapp E., Leszyk J., Shaffer S.A., Rockwell H.E., Gao F., Narain N.R., DiFiglia M., Kiebish M.A. (2016). High-resolution proteomic and lipidomic analysis of exosomes and microvesicles from different cell sources. J. Extracell. Vesicles.

[32] Kooijmans S.A.A., Vader P., van Dommelen S.M., van Solinge W.W., Schiffelers R.M. (2012). Exosome mimetics: A novel class of drug delivery systems. Int. J. Nanomed..

[33] Verderio C., Gabrielli M., Giussani P. (2018). Role of sphingolipids in the biogenesis and biological activity of extracellular vesicles. J. Lipid Res..

[34] Fadok V.A., Voelker D.R., Campbell P.A., Cohen J.J., Bratton D.L., Henson P.M. (1992). Exposure of phosphatidylserine on the surface of apoptotic lymphocytes triggers specific recognition and removal by macrophages. J. Immunol..

[35] Fadok V.A., Bratton D.L., Frasch S.C., Warner M.L., Henson P.M. (1998). The role of phosphatidylserine in recognition of apoptotic cells by phagocytes. Cell Death Differ..

[36] Tuteja N., Chandra M., Tuteja R., Misra M.K. (2004). Nitric Oxide as a Unique Bioactive Signaling Messenger in Physiology and Pathophysiology. J. Biomed. Biotechnol..

[37] Farah C., Michel L.Y.M., Balligand J.-L. (2018). Nitric oxide signalling in cardiovascular health and disease. Nat. Rev. Cardiol..

[38] Green S.J., Mellouk S., Hoffman S.L., Meltzer M.S., Nacy C.A. (1990). Cellular mechanisms of nonspecific immunity to intracellular infection: Cytokine-induced synthesis of toxic nitrogen oxides from l-arginine by macrophages and hepatocytes. Immunol. Lett..

[39] Bogdan C. (2001). Nitric oxide and the immune response. Nat. Immunol..

[40] Bryan N.S., Grisham M.B. (2007). Methods to detect nitric oxide and its metabolites in biological samples. Free. Radic. Biol. Med..

[41] Halim A.T.A., Ariffin N.A.F.M., Azlan M. (2016). Review: The Multiple Roles of Monocytic Microparticles. Inflammation.

[42] Niki E., Poli G., Albano E., Dianzani M.U. (1993). Antioxidant Defenses in Eukariotic Cells: An Overview. Free Radicals: From Basic Science to Medicine.

[43] Ighodaro O.M., Akinloye O.A. (2018). First line defence antioxidants-superoxide dismutase (SOD), catalase (CAT) and glutathione peroxidase (GPX): Their fundamental role in the entire antioxidant defence grid. Alex. J. Med..

[44] Ayala A., Muñoz M.F., Argüelles S. (2014). Lipid peroxidation: Production, metabolism, and signaling mechanisms of malondialdehyde and 4-hydroxy-2-nonenal. Oxidative Med. Cell. Longev..

[45] Lewis S.M., Williams A., Eisenbarth S.C. (2019). Structure and function of the immune system in the spleen. Sci. Immunol..

[46] Kauffman K.J., Mir F.F., Jhunjhunwala S., Kaczmarek J.C., Hurtado J.E., Yang J.H., Webber M.J., Kowalski P.S., Heartlein M.W., DeRosa F. (2016). Efficacy and immunogenicity of unmodified and pseudouridine-modified mRNA delivered systemically with lipid nanoparticles in vivo. Biomaterials.

[47] Ou Y.-H., Liang J., Chng W.H., Muthuramalingam R.P.K., Ng Z.X., Lee C.K., Neupane Y.R., Yau J.N.N., Zhang S., Lou C.K.L. (2022). Investigations on Cellular Uptake Mechanisms and Immunogenicity Profile of Novel Bio-Hybrid Nanovesicles. Pharmaceutics.

[48] Marzio R., Jirillo E., Ransijn A., Mauël J., Corradin S.B. (1997). Expression and function of the early activation antigen CD69 in murine macrophages. J. Leukoc. Biol..

[49] Cibrián D., Sánchez-Madrid F. (2017). CD69: From activation marker to metabolic gatekeeper. Eur. J. Immunol..

