# Supplementary material for: Extracellular Vesicles and Their Mimetics: A Comparative Study of Their Pharmacological Activities and Immunogenicity Profiles
Source: Pharmaceutics. 2023 Apr 20;15(4):1290. doi: 10.3390/pharmaceutics15041290 (PMC10142599; doi:10.3390/pharmaceutics15041290)

Supplementary Material

Extracellular Vesicles and their mimetics: A Comparative Study of their Pharmacological Activities and Immunogenicity Profiles

Wei Heng Chng [1,2,†], Ram Pravin Kumar Muthuramalingam [2,†], Charles Kang Liang Lou [3,4], Silas New [2], Yub Raj Neupane [2,5], Choon Keong Lee [2], Ayca Altay Benetti [2], Chenyuan Huang [3,6], Praveen Thoniyot [7], Wei Seong Toh [1,8,9], Jiong-Wei Wang [3,4,6,10] and Giorgia Pastorin [1,2,*]

Supplementary Table S1: Proteomics Analysis of A) U937 EVs and B) U937 CDNs (95% confidence intervals and 1% false discovery rate).

A) Proteomics Analysis of U937 EVs

Accession #	Species	Name
sp|P49327|FAS_HUMAN	HUMAN	Fatty acid synthase OS=Homo sapiens OX=9606 GN=FASN PE=1 SV=3
sp|Q14204|DYHC1_HUMAN	HUMAN	Cytoplasmic dynein 1 heavy chain 1 OS=Homo sapiens OX=9606 GN=DYNC1H1 PE=1 SV=5
sp|Q00610|CLH1_HUMAN	HUMAN	Clathrin heavy chain 1 OS=Homo sapiens OX=9606 GN=CLTC PE=1 SV=5
sp|P78527|PRKDC_HUMAN	HUMAN	DNA-dependent protein kinase catalytic subunit OS=Homo sapiens OX=9606 GN=PRKDC PE=1 SV=3
sp|P29144|TPP2_HUMAN	HUMAN	Tripeptidyl-peptidase 2 OS=Homo sapiens OX=9606 GN=TPP2 PE=1 SV=4
sp|P35579|MYH9_HUMAN	HUMAN	Myosin-9 OS=Homo sapiens OX=9606 GN=MYH9 PE=1 SV=4
sp|Q9Y490|TLN1_HUMAN	HUMAN	Talin-1 OS=Homo sapiens OX=9606 GN=TLN1 PE=1 SV=3
sp|P14618|KPYM_HUMAN	HUMAN	Pyruvate kinase PKM OS=Homo sapiens OX=9606 GN=PKM PE=1 SV=4
sp|P08238|HS90B_HUMAN	HUMAN	Heat shock protein HSP 90-beta OS=Homo sapiens OX=9606 GN=HSP90AB1 PE=1 SV=4
sp|P53396|ACLY_HUMAN	HUMAN	ATP-citrate synthase OS=Homo sapiens OX=9606 GN=ACLY PE=1 SV=3
contam_sp|ALBU_BOVIN|		contam_sp|ALBU_BOVIN|
sp|P04264|K2C1_HUMAN	HUMAN	Keratin, type II cytoskeletal 1 OS=Homo sapiens OX=9606 GN=KRT1 PE=1 SV=6
sp|P22314|UBA1_HUMAN	HUMAN	Ubiquitin-like modifier-activating enzyme 1 OS=Homo sapiens OX=9606 GN=UBA1 PE=1 SV=3
sp|P53621|COPA_HUMAN	HUMAN	Coatomer subunit alpha OS=Homo sapiens OX=9606 GN=COPA PE=1 SV=2
sp|O43707|ACTN4_HUMAN	HUMAN	Alpha-actinin-4 OS=Homo sapiens OX=9606 GN=ACTN4 PE=1 SV=2
sp|P13639|EF2_HUMAN	HUMAN	Elongation factor 2 OS=Homo sapiens OX=9606 GN=EEF2 PE=1 SV=4
sp|P41252|SYIC_HUMAN	HUMAN	Isoleucine--tRNA ligase, cytoplasmic OS=Homo sapiens OX=9606 GN=IARS1 PE=1 SV=2
sp|P78371|TCPB_HUMAN	HUMAN	T-complex protein 1 subunit beta OS=Homo sapiens OX=9606 GN=CCT2 PE=1 SV=4
sp|P26038|MOES_HUMAN	HUMAN	Moesin OS=Homo sapiens OX=9606 GN=MSN PE=1 SV=3
sp|P07814|SYEP_HUMAN	HUMAN	Bifunctional glutamate/proline--tRNA ligase OS=Homo sapiens OX=9606 GN=EPRS1 PE=1 SV=5
sp|P11142|HSP7C_HUMAN	HUMAN	Heat shock cognate 71 kDa protein OS=Homo sapiens OX=9606 GN=HSPA8 PE=1 SV=1
sp|Q08211|DHX9_HUMAN	HUMAN	ATP-dependent RNA helicase A OS=Homo sapiens OX=9606 GN=DHX9 PE=1 SV=4
sp|P35527|K1C9_HUMAN	HUMAN	Keratin, type I cytoskeletal 9 OS=Homo sapiens OX=9606 GN=KRT9 PE=1 SV=3
sp|P17987|TCPA_HUMAN	HUMAN	T-complex protein 1 subunit alpha OS=Homo sapiens OX=9606 GN=TCP1 PE=1 SV=1
sp|P48643|TCPE_HUMAN	HUMAN	T-complex protein 1 subunit epsilon OS=Homo sapiens OX=9606 GN=CCT5 PE=1 SV=1
sp|P46940|IQGA1_HUMAN	HUMAN	Ras GTPase-activating-like protein IQGAP1 OS=Homo sapiens OX=9606 GN=IQGAP1 PE=1 SV=1
sp|P08575|PTPRC_HUMAN	HUMAN	Receptor-type tyrosine-protein phosphatase C OS=Homo sapiens OX=9606 GN=PTPRC PE=1 SV=3

sp|P26640|SYVC_HUMAN	HUMAN	Valine--tRNA ligase OS=Homo sapiens OX=9606 GN=VARS1 PE=1 SV=4
sp|P07437|TBB5_HUMAN	HUMAN	Tubulin beta chain OS=Homo sapiens OX=9606 GN=TUBB PE=1 SV=2
sp|P13796|PLSL_HUMAN	HUMAN	Plastin-2 OS=Homo sapiens OX=9606 GN=LCP1 PE=1 SV=6
sp|P15144|AMPN_HUMAN	HUMAN	Aminopeptidase N OS=Homo sapiens OX=9606 GN=ANPEP PE=1 SV=4
sp|P27708|PYR1_HUMAN	HUMAN	CAD protein OS=Homo sapiens OX=9606 GN=CAD PE=1 SV=3
sp|O14980|XPO1_HUMAN	HUMAN	Exportin-1 OS=Homo sapiens OX=9606 GN=XPO1 PE=1 SV=1
sp|P49368|TCPG_HUMAN	HUMAN	T-complex protein 1 subunit gamma OS=Homo sapiens OX=9606 GN=CCT3 PE=1 SV=4
sp|Q99832|TCPH_HUMAN	HUMAN	T-complex protein 1 subunit eta OS=Homo sapiens OX=9606 GN=CCT7 PE=1 SV=2
sp|P50990|TCPQ_HUMAN	HUMAN	T-complex protein 1 subunit theta OS=Homo sapiens OX=9606 GN=CCT8 PE=1 SV=4
sp|P06737|PYGL_HUMAN	HUMAN	Glycogen phosphorylase, liver form OS=Homo sapiens OX=9606 GN=PYGL PE=1 SV=4
sp|P12956|XRCC6_HUMAN	HUMAN	X-ray repair cross-complementing protein 6 OS=Homo sapiens OX=9606 GN=XRCC6 PE=1 SV=2
sp|P02786|TFR1_HUMAN	HUMAN	Transferrin receptor protein 1 OS=Homo sapiens OX=9606 GN=TFRC PE=1 SV=2
sp|P00558|PGK1_HUMAN	HUMAN	Phosphoglycerate kinase 1 OS=Homo sapiens OX=9606 GN=PGK1 PE=1 SV=3
sp|Q9P2J5|SYLC_HUMAN	HUMAN	Leucine--tRNA ligase, cytoplasmic OS=Homo sapiens OX=9606 GN=LARS1 PE=1 SV=2
sp|P49588|SYAC_HUMAN	HUMAN	Alanine--tRNA ligase, cytoplasmic OS=Homo sapiens OX=9606 GN=AARS1 PE=1 SV=2
sp|P55072|TERA_HUMAN	HUMAN	Transitional endoplasmic reticulum ATPase OS=Homo sapiens OX=9606 GN=VCP PE=1 SV=4
sp|P13010|XRCC5_HUMAN	HUMAN	X-ray repair cross-complementing protein 5 OS=Homo sapiens OX=9606 GN=XRCC5 PE=1 SV=3
sp|Q71U36|TBA1A_HUMAN	HUMAN	Tubulin alpha-1A chain OS=Homo sapiens OX=9606 GN=TUBA1A PE=1 SV=1
sp|P08133|ANXA6_HUMAN	HUMAN	Annexin A6 OS=Homo sapiens OX=9606 GN=ANXA6 PE=1 SV=3
sp|P08195|4F2_HUMAN	HUMAN	4F2 cell-surface antigen heavy chain OS=Homo sapiens OX=9606 GN=SLC3A2 PE=1 SV=3
sp|P05023|AT1A1_HUMAN	HUMAN	Sodium/potassium-transporting ATPase subunit alpha-1 OS=Homo sapiens OX=9606 GN=ATP1A1 PE=1 SV=1
sp|B0I1T2|MYO1G_HUMAN	HUMAN	Unconventional myosin-Ig OS=Homo sapiens OX=9606 GN=MYO1G PE=1 SV=2
sp|P63261|ACTG_HUMAN	HUMAN	Actin, cytoplasmic 2 OS=Homo sapiens OX=9606 GN=ACTG1 PE=1 SV=1
sp|Q14152|EIF3A_HUMAN	HUMAN	Eukaryotic translation initiation factor 3 subunit A OS=Homo sapiens OX=9606 GN=EIF3A PE=1 SV=1
sp|P13645|K1C10_HUMAN	HUMAN	Keratin, type I cytoskeletal 10 OS=Homo sapiens OX=9606 GN=KRT10 PE=1 SV=6
sp|P50991|TCPD_HUMAN	HUMAN	T-complex protein 1 subunit delta OS=Homo sapiens OX=9606 GN=CCT4 PE=1 SV=4
sp|P04439|HLAA_HUMAN	HUMAN	HLA class I histocompatibility antigen, A alpha chain OS=Homo sapiens OX=9606 GN=HLA-A PE=1 SV=2
sp|P14868|SYDC_HUMAN	HUMAN	Aspartate--tRNA ligase, cytoplasmic OS=Homo sapiens OX=9606 GN=DARS1 PE=1 SV=2
sp|Q16555|DPYL2_HUMAN	HUMAN	Dihydropyrimidinase-related protein 2 OS=Homo sapiens OX=9606 GN=DPYSL2 PE=1 SV=1
sp|Q15393|SF3B3_HUMAN	HUMAN	Splicing factor 3B subunit 3 OS=Homo sapiens OX=9606 GN=SF3B3 PE=1 SV=4

sp|Q9Y262|EIF3L_HUMAN	HUMAN	Eukaryotic translation initiation factor 3 subunit L OS=Homo sapiens OX=9606 GN=EIF3L PE=1 SV=1
sp|P40227|TCPZ_HUMAN	HUMAN	T-complex protein 1 subunit zeta OS=Homo sapiens OX=9606 GN=CCT6A PE=1 SV=3
sp|P35908|K22E_HUMAN	HUMAN	Keratin, type II cytoskeletal 2 epidermal OS=Homo sapiens OX=9606 GN=KRT2 PE=1 SV=2
sp|P08779|K1C16_HUMAN	HUMAN	Keratin, type I cytoskeletal 16 OS=Homo sapiens OX=9606 GN=KRT16 PE=1 SV=4
sp|O75533|SF3B1_HUMAN	HUMAN	Splicing factor 3B subunit 1 OS=Homo sapiens OX=9606 GN=SF3B1 PE=1 SV=3
sp|P04406|G3P_HUMAN	HUMAN	Glyceraldehyde-3-phosphate dehydrogenase OS=Homo sapiens OX=9606 GN=GAPDH PE=1 SV=3
sp|P33993|MCM7_HUMAN	HUMAN	DNA replication licensing factor MCM7 OS=Homo sapiens OX=9606 GN=MCM7 PE=1 SV=4
sp|Q86UX7|URP2_HUMAN	HUMAN	Fermitin family homolog 3 OS=Homo sapiens OX=9606 GN=FERMT3 PE=1 SV=1
sp|Q02880|TOP2B_HUMAN	HUMAN	DNA topoisomerase 2-beta OS=Homo sapiens OX=9606 GN=TOP2B PE=1 SV=3
sp|P23526|SAHH_HUMAN	HUMAN	Adenosylhomocysteinase OS=Homo sapiens OX=9606 GN=AHCY PE=1 SV=4
sp|P07900|HS90A_HUMAN	HUMAN	Heat shock protein HSP 90-alpha OS=Homo sapiens OX=9606 GN=HSP90AA1 PE=1 SV=5
sp|Q99460|PSMD1_HUMAN	HUMAN	26S proteasome non-ATPase regulatory subunit 1 OS=Homo sapiens OX=9606 GN=PSMD1 PE=1 SV=2
sp|P49736|MCM2_HUMAN	HUMAN	DNA replication licensing factor MCM2 OS=Homo sapiens OX=9606 GN=MCM2 PE=1 SV=4
sp|P23396|RS3_HUMAN	HUMAN	40S ribosomal protein S3 OS=Homo sapiens OX=9606 GN=RPS3 PE=1 SV=2
sp|P61158|ARP3_HUMAN	HUMAN	Actin-related protein 3 OS=Homo sapiens OX=9606 GN=ACTR3 PE=1 SV=3
sp|P06733|ENOA_HUMAN	HUMAN	Alpha-enolase OS=Homo sapiens OX=9606 GN=ENO1 PE=1 SV=2
sp|P41250|GARS_HUMAN	HUMAN	Glycine--tRNA ligase OS=Homo sapiens OX=9606 GN=GARS1 PE=1 SV=3
sp|Q8WUM4|PDC6I_HUMAN	HUMAN	Programmed cell death 6-interacting protein OS=Homo sapiens OX=9606 GN=PDCD6IP PE=1 SV=1
sp|P63244|RACK1_HUMAN	HUMAN	Receptor of activated protein C kinase 1 OS=Homo sapiens OX=9606 GN=RACK1 PE=1 SV=3
sp|P11717|MPRI_HUMAN	HUMAN	Cation-independent mannose-6-phosphate receptor OS=Homo sapiens OX=9606 GN=IGF2R PE=1 SV=3
sp|P05556|ITB1_HUMAN	HUMAN	Integrin beta-1 OS=Homo sapiens OX=9606 GN=ITGB1 PE=1 SV=2
sp|P31939|PUR9_HUMAN	HUMAN	Bifunctional purine biosynthesis protein PURH OS=Homo sapiens OX=9606 GN=ATIC PE=1 SV=3
sp|Q9H4A4|AMPB_HUMAN	HUMAN	Aminopeptidase B OS=Homo sapiens OX=9606 GN=RNPEP PE=1 SV=2
sp|P60842|IF4A1_HUMAN	HUMAN	Eukaryotic initiation factor 4A-I OS=Homo sapiens OX=9606 GN=EIF4A1 PE=1 SV=1
sp|P33992|MCM5_HUMAN	HUMAN	DNA replication licensing factor MCM5 OS=Homo sapiens OX=9606 GN=MCM5 PE=1 SV=5
sp|P26641|EF1G_HUMAN	HUMAN	Elongation factor 1-gamma OS=Homo sapiens OX=9606 GN=EEF1G PE=1 SV=3
sp|P47897|SYQ_HUMAN	HUMAN	Glutamine--tRNA ligase OS=Homo sapiens OX=9606 GN=QARS1 PE=1 SV=1
sp|O75955|FLOT1_HUMAN	HUMAN	Flotillin-1 OS=Homo sapiens OX=9606 GN=FLOT1 PE=1 SV=3
sp|Q13200|PSMD2_HUMAN	HUMAN	26S proteasome non-ATPase regulatory subunit 2 OS=Homo sapiens OX=9606 GN=PSMD2 PE=1 SV=3
sp|P53618|COPB_HUMAN	HUMAN	Coatomer subunit beta OS=Homo sapiens OX=9606 GN=COPB1 PE=1 SV=3
sp|Q14697|GANAB_HUMAN	HUMAN	Neutral alpha-glucosidase AB OS=Homo sapiens OX=9606 GN=GANAB PE=1 SV=3

sp|P49591|SYSC_HUMAN	HUMAN	Serine--tRNA ligase, cytoplasmic OS=Homo sapiens OX=9606 GN=SARS1 PE=1 SV=3
sp|O75643|U520_HUMAN	HUMAN	U5 small nuclear ribonucleoprotein 200 kDa helicase OS=Homo sapiens OX=9606 GN=SNRNP200 PE=1 SV=2
sp|Q14764|MVP_HUMAN	HUMAN	Major vault protein OS=Homo sapiens OX=9606 GN=MVP PE=1 SV=4
sp|P0DMV9|HS71B_HUMAN	HUMAN	Heat shock 70 kDa protein 1B OS=Homo sapiens OX=9606 GN=HSPA1B PE=1 SV=1
sp|O43175|SERA_HUMAN	HUMAN	D-3-phosphoglycerate dehydrogenase OS=Homo sapiens OX=9606 GN=PHGDH PE=1 SV=4
sp|P08311|CATG_HUMAN	HUMAN	Cathepsin G OS=Homo sapiens OX=9606 GN=CTSG PE=1 SV=2
sp|P11586|C1TC_HUMAN	HUMAN	C-1-tetrahydrofolate synthase, cytoplasmic OS=Homo sapiens OX=9606 GN=MTHFD1 PE=1 SV=3
sp|P50995|ANX11_HUMAN	HUMAN	Annexin A11 OS=Homo sapiens OX=9606 GN=ANXA11 PE=1 SV=1
sp|P05107|ITB2_HUMAN	HUMAN	Integrin beta-2 OS=Homo sapiens OX=9606 GN=ITGB2 PE=1 SV=2
sp|P21399|ACOC_HUMAN	HUMAN	Cytoplasmic aconitate hydratase OS=Homo sapiens OX=9606 GN=ACO1 PE=1 SV=3
sp|P68104|EF1A1_HUMAN	HUMAN	Elongation factor 1-alpha 1 OS=Homo sapiens OX=9606 GN=EEF1A1 PE=1 SV=1
sp|P08648|ITA5_HUMAN	HUMAN	Integrin alpha-5 OS=Homo sapiens OX=9606 GN=ITGA5 PE=1 SV=2
sp|P04899|GNAI2_HUMAN	HUMAN	Guanine nucleotide-binding protein G(i) subunit alpha-2 OS=Homo sapiens OX=9606 GN=GNAI2 PE=1 SV=3
sp|P21333|FLNA_HUMAN	HUMAN	Filamin-A OS=Homo sapiens OX=9606 GN=FLNA PE=1 SV=4
sp|P07355|ANXA2_HUMAN	HUMAN	Annexin A2 OS=Homo sapiens OX=9606 GN=ANXA2 PE=1 SV=2
sp|O00299|CLIC1_HUMAN	HUMAN	Chloride intracellular channel protein 1 OS=Homo sapiens OX=9606 GN=CLIC1 PE=1 SV=4
sp|P08865|RSSA_HUMAN	HUMAN	40S ribosomal protein SA OS=Homo sapiens OX=9606 GN=RPSA PE=1 SV=4
sp|P23634|AT2B4_HUMAN	HUMAN	Plasma membrane calcium-transporting ATPase 4 OS=Homo sapiens OX=9606 GN=ATP2B4 PE=1 SV=2
sp|O75083|WDR1_HUMAN	HUMAN	WD repeat-containing protein 1 OS=Homo sapiens OX=9606 GN=WDR1 PE=1 SV=4
sp|O75044|SRGP2_HUMAN	HUMAN	SLIT-ROBO Rho GTPase-activating protein 2 OS=Homo sapiens OX=9606 GN=SRGAP2 PE=1 SV=3
sp|Q15758|AAAT_HUMAN	HUMAN	Neutral amino acid transporter B(0) OS=Homo sapiens OX=9606 GN=SLC1A5 PE=1 SV=2
sp|Q14974|IMB1_HUMAN	HUMAN	Importin subunit beta-1 OS=Homo sapiens OX=9606 GN=KPNB1 PE=1 SV=2
sp|P17858|PFKAL_HUMAN	HUMAN	ATP-dependent 6-phosphofructokinase, liver type OS=Homo sapiens OX=9606 GN=PFKL PE=1 SV=6
sp|P31146|COR1A_HUMAN	HUMAN	Coronin-1A OS=Homo sapiens OX=9606 GN=CORO1A PE=1 SV=4
sp|P11216|PYGB_HUMAN	HUMAN	Glycogen phosphorylase, brain form OS=Homo sapiens OX=9606 GN=PYGB PE=1 SV=5
sp|Q7KZF4|SND1_HUMAN	HUMAN	Staphylococcal nuclease domain-containing protein 1 OS=Homo sapiens OX=9606 GN=SND1 PE=1 SV=1
sp|Q9Y230|RUVB2_HUMAN	HUMAN	RuvB-like 2 OS=Homo sapiens OX=9606 GN=RUVBL2 PE=1 SV=3
contam_sp|TRYP_PIG|		contam_sp|TRYP_PIG|
sp|P07948|LYN_HUMAN	HUMAN	Tyrosine-protein kinase Lyn OS=Homo sapiens OX=9606 GN=LYN PE=1 SV=3
sp|O43143|DHX15_HUMAN	HUMAN	Pre-mRNA-splicing factor ATP-dependent RNA helicase DHX15 OS=Homo sapiens OX=9606 GN=DHX15 PE=1 SV=2

sp|P55884|EIF3B_HUMAN	HUMAN	Eukaryotic translation initiation factor 3 subunit B OS=Homo sapiens OX=9606 GN=EIF3B PE=1 SV=3
sp|Q01581|HMCS1_HUMAN	HUMAN	Hydroxymethylglutaryl-CoA synthase, cytoplasmic OS=Homo sapiens OX=9606 GN=HMGCS1 PE=1 SV=2
sp|Q6WKZ4|RFIP1_HUMAN	HUMAN	Rab11 family-interacting protein 1 OS=Homo sapiens OX=9606 GN=RAB11FIP1 PE=1 SV=3
sp|P06744|G6PI_HUMAN	HUMAN	Glucose-6-phosphate isomerase OS=Homo sapiens OX=9606 GN=GPI PE=1 SV=4
sp|O43242|PSMD3_HUMAN	HUMAN	26S proteasome non-ATPase regulatory subunit 3 OS=Homo sapiens OX=9606 GN=PSMD3 PE=1 SV=2
sp|O14745|NHRF1_HUMAN	HUMAN	Na(+)/H(+) exchange regulatory cofactor NHE-RF1 OS=Homo sapiens OX=9606 GN=SLC9A3R1 PE=1 SV=4
sp|Q01518|CAP1_HUMAN	HUMAN	Adenylyl cyclase-associated protein 1 OS=Homo sapiens OX=9606 GN=CAP1 PE=1 SV=5
sp|P61160|ARP2_HUMAN	HUMAN	Actin-related protein 2 OS=Homo sapiens OX=9606 GN=ACTR2 PE=1 SV=1
sp|Q9Y265|RUVB1_HUMAN	HUMAN	RuvB-like 1 OS=Homo sapiens OX=9606 GN=RUVBL1 PE=1 SV=1
sp|P23528|COF1_HUMAN	HUMAN	Cofilin-1 OS=Homo sapiens OX=9606 GN=CFL1 PE=1 SV=3
sp|Q96QK1|VPS35_HUMAN	HUMAN	Vacuolar protein sorting-associated protein 35 OS=Homo sapiens OX=9606 GN=VPS35 PE=1 SV=2
sp|Q6P2Q9|PRP8_HUMAN	HUMAN	Pre-mRNA-processing-splicing factor 8 OS=Homo sapiens OX=9606 GN=PRPF8 PE=1 SV=2
sp|Q9P265|DIP2B_HUMAN	HUMAN	Disco-interacting protein 2 homolog B OS=Homo sapiens OX=9606 GN=DIP2B PE=1 SV=3
sp|O00232|PSD12_HUMAN	HUMAN	26S proteasome non-ATPase regulatory subunit 12 OS=Homo sapiens OX=9606 GN=PSMD12 PE=1 SV=3
sp|P38919|IF4A3_HUMAN	HUMAN	Eukaryotic initiation factor 4A-III OS=Homo sapiens OX=9606 GN=EIF4A3 PE=1 SV=4
sp|Q9Y3I0|RTCB_HUMAN	HUMAN	RNA-splicing ligase RtcB homolog OS=Homo sapiens OX=9606 GN=RTCB PE=1 SV=1
sp|P15311|EZRI_HUMAN	HUMAN	Ezrin OS=Homo sapiens OX=9606 GN=EZR PE=1 SV=4
sp|P26639|SYTC_HUMAN	HUMAN	Threonine--tRNA ligase 1, cytoplasmic OS=Homo sapiens OX=9606 GN=TARS1 PE=1 SV=3
sp|Q15046|SYK_HUMAN	HUMAN	Lysine--tRNA ligase OS=Homo sapiens OX=9606 GN=KARS1 PE=1 SV=3
sp|Q9P258|RCC2_HUMAN	HUMAN	Protein RCC2 OS=Homo sapiens OX=9606 GN=RCC2 PE=1 SV=2
sp|P27348|1433T_HUMAN	HUMAN	14-3-3 protein theta OS=Homo sapiens OX=9606 GN=YWHAQ PE=1 SV=1
sp|P13612|ITA4_HUMAN	HUMAN	Integrin alpha-4 OS=Homo sapiens OX=9606 GN=ITGA4 PE=1 SV=3
sp|P62805|H4_HUMAN	HUMAN	Histone H4 OS=Homo sapiens OX=9606 GN=H4C1 PE=1 SV=2
sp|P62873|GBB1_HUMAN	HUMAN	Guanine nucleotide-binding protein G(I)/G(S)/G(T) subunit beta-1 OS=Homo sapiens OX=9606 GN=GNB1 PE=1 SV=3
sp|P15880|RS2_HUMAN	HUMAN	40S ribosomal protein S2 OS=Homo sapiens OX=9606 GN=RPS2 PE=1 SV=2
sp|P05388|RLA0_HUMAN	HUMAN	60S acidic ribosomal protein P0 OS=Homo sapiens OX=9606 GN=RPLP0 PE=1 SV=1
sp|Q14254|FLOT2_HUMAN	HUMAN	Flotillin-2 OS=Homo sapiens OX=9606 GN=FLOT2 PE=1 SV=2
sp|P11387|TOP1_HUMAN	HUMAN	DNA topoisomerase 1 OS=Homo sapiens OX=9606 GN=TOP1 PE=1 SV=2
sp|P61026|RAB10_HUMAN	HUMAN	Ras-related protein Rab-10 OS=Homo sapiens OX=9606 GN=RAB10 PE=1 SV=1
sp|P60228|EIF3E_HUMAN	HUMAN	Eukaryotic translation initiation factor 3 subunit E OS=Homo sapiens OX=9606 GN=EIF3E PE=1 SV=1

sp|P30153|2AAA_HUMAN	HUMAN	Serine/threonine-protein phosphatase 2A 65 kDa regulatory subunit A alpha isoform OS=Homo sapiens OX=9606 GN=PPP2R1A PE=1 SV=4
sp|Q92841|DDX17_HUMAN	HUMAN	Probable ATP-dependent RNA helicase DDX17 OS=Homo sapiens OX=9606 GN=DDX17 PE=1 SV=2
sp|Q13263|TIF1B_HUMAN	HUMAN	Transcription intermediary factor 1-beta OS=Homo sapiens OX=9606 GN=TRIM28 PE=1 SV=5
sp|Q15008|PSMD6_HUMAN	HUMAN	26S proteasome non-ATPase regulatory subunit 6 OS=Homo sapiens OX=9606 GN=PSMD6 PE=1 SV=1
sp|P15153|RAC2_HUMAN	HUMAN	Ras-related C3 botulinum toxin substrate 2 OS=Homo sapiens OX=9606 GN=RAC2 PE=1 SV=1
sp|P18669|PGAM1_HUMAN	HUMAN	Phosphoglycerate mutase 1 OS=Homo sapiens OX=9606 GN=PGAM1 PE=1 SV=2
sp|Q15907|RB11B_HUMAN	HUMAN	Ras-related protein Rab-11B OS=Homo sapiens OX=9606 GN=RAB11B PE=1 SV=4
sp|P54920|SNAA_HUMAN	HUMAN	Alpha-soluble NSF attachment protein OS=Homo sapiens OX=9606 GN=NAPA PE=1 SV=3
sp|P01130|LDLR_HUMAN	HUMAN	Low-density lipoprotein receptor OS=Homo sapiens OX=9606 GN=LDLR PE=1 SV=1
sp|P08567|PLEK_HUMAN	HUMAN	Pleckstrin OS=Homo sapiens OX=9606 GN=PLEK PE=1 SV=3
sp|P01024|CO3_HUMAN	HUMAN	Complement C3 OS=Homo sapiens OX=9606 GN=C3 PE=1 SV=2
sp|Q16576|RBBP7_HUMAN	HUMAN	Histone-binding protein RBBP7 OS=Homo sapiens OX=9606 GN=RBBP7 PE=1 SV=1
sp|P48668|K2C6C_HUMAN	HUMAN	Keratin, type II cytoskeletal 6C OS=Homo sapiens OX=9606 GN=KRT6C PE=1 SV=3
sp|Q99873|ANM1_HUMAN	HUMAN	Protein arginine N-methyltransferase 1 OS=Homo sapiens OX=9606 GN=PRMT1 PE=1 SV=3
sp|P62937|PPIA_HUMAN	HUMAN	Peptidyl-prolyl cis-trans isomerase A OS=Homo sapiens OX=9606 GN=PPIA PE=1 SV=2
sp|P60900|PSA6_HUMAN	HUMAN	Proteasome subunit alpha type-6 OS=Homo sapiens OX=9606 GN=PSMA6 PE=1 SV=1
sp|P35606|COPB2_HUMAN	HUMAN	Coatomer subunit beta' OS=Homo sapiens OX=9606 GN=COPB2 PE=1 SV=2
sp|P62195|PRS8_HUMAN	HUMAN	26S proteasome regulatory subunit 8 OS=Homo sapiens OX=9606 GN=PSMC5 PE=1 SV=1
sp|P62136|PP1A_HUMAN	HUMAN	Serine/threonine-protein phosphatase PP1-alpha catalytic subunit OS=Homo sapiens OX=9606 GN=PPP1CA PE=1 SV=1
sp|P19367|HXK1_HUMAN	HUMAN	Hexokinase-1 OS=Homo sapiens OX=9606 GN=HK1 PE=1 SV=3
sp|Q9Y696|CLIC4_HUMAN	HUMAN	Chloride intracellular channel protein 4 OS=Homo sapiens OX=9606 GN=CLIC4 PE=1 SV=4
sp|Q9H4M9|EHD1_HUMAN	HUMAN	EH domain-containing protein 1 OS=Homo sapiens OX=9606 GN=EHD1 PE=1 SV=2
sp|O95782|AP2A1_HUMAN	HUMAN	AP-2 complex subunit alpha-1 OS=Homo sapiens OX=9606 GN=AP2A1 PE=1 SV=3
sp|Q9UIA9|XPO7_HUMAN	HUMAN	Exportin-7 OS=Homo sapiens OX=9606 GN=XPO7 PE=1 SV=3
sp|P51149|RAB7A_HUMAN	HUMAN	Ras-related protein Rab-7a OS=Homo sapiens OX=9606 GN=RAB7A PE=1 SV=1
sp|Q7L576|CYFP1_HUMAN	HUMAN	Cytoplasmic FMR1-interacting protein 1 OS=Homo sapiens OX=9606 GN=CYFIP1 PE=1 SV=1
sp|Q99536|VAT1_HUMAN	HUMAN	Synaptic vesicle membrane protein VAT-1 homolog OS=Homo sapiens OX=9606 GN=VAT1 PE=1 SV=2
sp|P09211|GSTP1_HUMAN	HUMAN	Glutathione S-transferase P OS=Homo sapiens OX=9606 GN=GSTP1 PE=1 SV=2
sp|P50395|GDIB_HUMAN	HUMAN	Rab GDP dissociation inhibitor beta OS=Homo sapiens OX=9606 GN=GDI2 PE=1 SV=2
sp|P48960|CD97_HUMAN	HUMAN	CD97 antigen OS=Homo sapiens OX=9606 GN=CD97 PE=1 SV=4

sp|O43776|SYNC_HUMAN	HUMAN	Asparagine--tRNA ligase, cytoplasmic OS=Homo sapiens OX=9606 GN=NARS1 PE=1 SV=1
sp|Q13838|DX39B_HUMAN	HUMAN	Spliceosome RNA helicase DDX39B OS=Homo sapiens OX=9606 GN=DDX39B PE=1 SV=1
sp|P41091|IF2G_HUMAN	HUMAN	Eukaryotic translation initiation factor 2 subunit 3 OS=Homo sapiens OX=9606 GN=EIF2S3 PE=1 SV=3
sp|P62826|RAN_HUMAN	HUMAN	GTP-binding nuclear protein Ran OS=Homo sapiens OX=9606 GN=RAN PE=1 SV=3
sp|P55209|NP1L1_HUMAN	HUMAN	Nucleosome assembly protein 1-like 1 OS=Homo sapiens OX=9606 GN=NAP1L1 PE=1 SV=1
sp|P46781|RS9_HUMAN	HUMAN	40S ribosomal protein S9 OS=Homo sapiens OX=9606 GN=RPS9 PE=1 SV=3
sp|P18124|RL7_HUMAN	HUMAN	60S ribosomal protein L7 OS=Homo sapiens OX=9606 GN=RPL7 PE=1 SV=1
sp|P36871|PGM1_HUMAN	HUMAN	Phosphoglucomutase-1 OS=Homo sapiens OX=9606 GN=PGM1 PE=1 SV=3
sp|P16284|PECA1_HUMAN	HUMAN	Platelet endothelial cell adhesion molecule OS=Homo sapiens OX=9606 GN=PECAM1 PE=1 SV=2
sp|Q6FI13|H2A2A_HUMAN	HUMAN	Histone H2A type 2-A OS=Homo sapiens OX=9606 GN=HIST2H2AA3 PE=1 SV=3
sp|P14625|ENPL_HUMAN	HUMAN	Endoplasmin OS=Homo sapiens OX=9606 GN=HSP90B1 PE=1 SV=1
sp|O96019|ACL6A_HUMAN	HUMAN	Actin-like protein 6A OS=Homo sapiens OX=9606 GN=ACTL6A PE=1 SV=1
sp|Q9UPT5|EXOC7_HUMAN	HUMAN	Exocyst complex component 7 OS=Homo sapiens OX=9606 GN=EXOC7 PE=1 SV=3
sp|P06576|ATPB_HUMAN	HUMAN	ATP synthase subunit beta, mitochondrial OS=Homo sapiens OX=9606 GN=ATP5F1B PE=1 SV=3
sp|Q9BXJ9|NAA15_HUMAN	HUMAN	N-alpha-acetyltransferase 15, NatA auxiliary subunit OS=Homo sapiens OX=9606 GN=NAA15 PE=1 SV=1
sp|P07237|PDIA1_HUMAN	HUMAN	Protein disulfide-isomerase OS=Homo sapiens OX=9606 GN=P4HB PE=1 SV=3
sp|Q9NSD9|SYFB_HUMAN	HUMAN	Phenylalanine--tRNA ligase beta subunit OS=Homo sapiens OX=9606 GN=FARSB PE=1 SV=3
sp|O00303|EIF3F_HUMAN	HUMAN	Eukaryotic translation initiation factor 3 subunit F OS=Homo sapiens OX=9606 GN=EIF3F PE=1 SV=1
sp|P50570|DYN2_HUMAN	HUMAN	Dynamin-2 OS=Homo sapiens OX=9606 GN=DNM2 PE=1 SV=2
sp|P00338|LDHA_HUMAN	HUMAN	L-lactate dehydrogenase A chain OS=Homo sapiens OX=9606 GN=LDHA PE=1 SV=2
sp|P54136|SYRC_HUMAN	HUMAN	Arginine--tRNA ligase, cytoplasmic OS=Homo sapiens OX=9606 GN=RARS1 PE=1 SV=2
sp|Q15029|U5S1_HUMAN	HUMAN	116 kDa U5 small nuclear ribonucleoprotein component OS=Homo sapiens OX=9606 GN=EFTUD2 PE=1 SV=1
sp|P62241|RS8_HUMAN	HUMAN	40S ribosomal protein S8 OS=Homo sapiens OX=9606 GN=RPS8 PE=1 SV=2
sp|P52209|6PGD_HUMAN	HUMAN	6-phosphogluconate dehydrogenase, decarboxylating OS=Homo sapiens OX=9606 GN=PGD PE=1 SV=3
sp|P34896|GLYC_HUMAN	HUMAN	Serine hydroxymethyltransferase, cytosolic OS=Homo sapiens OX=9606 GN=SHMT1 PE=1 SV=1
sp|P55786|PSA_HUMAN	HUMAN	Puromycin-sensitive aminopeptidase OS=Homo sapiens OX=9606 GN=NPEPPS PE=1 SV=2
sp|P43686|PRS6B_HUMAN	HUMAN	26S proteasome regulatory subunit 6B OS=Homo sapiens OX=9606 GN=PSMC4 PE=1 SV=2
sp|O14672|ADA10_HUMAN	HUMAN	Disintegrin and metalloproteinase domain-containing protein 10 OS=Homo sapiens OX=9606 GN=ADAM10 PE=1 SV=1
sp|P55060|XPO2_HUMAN	HUMAN	Exportin-2 OS=Homo sapiens OX=9606 GN=CSE1L PE=1 SV=3
sp|Q9UMS4|PRP19_HUMAN	HUMAN	Pre-mRNA-processing factor 19 OS=Homo sapiens OX=9606 GN=PRPF19 PE=1 SV=1

sp|P67775|PP2AA_HUMAN	HUMAN	Serine/threonine-protein phosphatase 2A catalytic subunit alpha isoform OS=Homo sapiens OX=9606 GN=PPP2CA PE=1 SV=1
sp|P62701|RS4X_HUMAN	HUMAN	40S ribosomal protein S4, X isoform OS=Homo sapiens OX=9606 GN=RPS4X PE=1 SV=2
sp|P27105|STOM_HUMAN	HUMAN	Erythrocyte band 7 integral membrane protein OS=Homo sapiens OX=9606 GN=STOM PE=1 SV=3
sp|P17980|PRS6A_HUMAN	HUMAN	26S proteasome regulatory subunit 6A OS=Homo sapiens OX=9606 GN=PSMC3 PE=1 SV=3
sp|P35613|BASI_HUMAN	HUMAN	Basigin OS=Homo sapiens OX=9606 GN=BSG PE=1 SV=2
sp|P61224|RAP1B_HUMAN	HUMAN	Ras-related protein Rap-1b OS=Homo sapiens OX=9606 GN=RAP1B PE=1 SV=1
sp|Q99613|EIF3C_HUMAN	HUMAN	Eukaryotic translation initiation factor 3 subunit C OS=Homo sapiens OX=9606 GN=EIF3C PE=1 SV=1
sp|P46777|RL5_HUMAN	HUMAN	60S ribosomal protein L5 OS=Homo sapiens OX=9606 GN=RPL5 PE=1 SV=3
sp|O43488|ARK72_HUMAN	HUMAN	Aflatoxin B1 aldehyde reductase member 2 OS=Homo sapiens OX=9606 GN=AKR7A2 PE=1 SV=3
sp|P38606|VATA_HUMAN	HUMAN	V-type proton ATPase catalytic subunit A OS=Homo sapiens OX=9606 GN=ATP6V1A PE=1 SV=2
sp|Q14566|MCM6_HUMAN	HUMAN	DNA replication licensing factor MCM6 OS=Homo sapiens OX=9606 GN=MCM6 PE=1 SV=1
sp|Q99880|H2B1L_HUMAN	HUMAN	Histone H2B type 1-L OS=Homo sapiens OX=9606 GN=H2BC13 PE=1 SV=3
sp|Q9UNM6|PSD13_HUMAN	HUMAN	26S proteasome non-ATPase regulatory subunit 13 OS=Homo sapiens OX=9606 GN=PSMD13 PE=1 SV=2
sp|O14818|PSA7_HUMAN	HUMAN	Proteasome subunit alpha type-7 OS=Homo sapiens OX=9606 GN=PSMA7 PE=1 SV=1
sp|Q9UQ80|PA2G4_HUMAN	HUMAN	Proliferation-associated protein 2G4 OS=Homo sapiens OX=9606 GN=PA2G4 PE=1 SV=3
sp|P39023|RL3_HUMAN	HUMAN	60S ribosomal protein L3 OS=Homo sapiens OX=9606 GN=RPL3 PE=1 SV=2
sp|P04075|ALDOA_HUMAN	HUMAN	Fructose-bisphosphate aldolase A OS=Homo sapiens OX=9606 GN=ALDOA PE=1 SV=2
sp|P63104|1433Z_HUMAN	HUMAN	14-3-3 protein zeta/delta OS=Homo sapiens OX=9606 GN=YWHAZ PE=1 SV=1
sp|Q9Y285|SYFA_HUMAN	HUMAN	Phenylalanine--tRNA ligase alpha subunit OS=Homo sapiens OX=9606 GN=FARSA PE=1 SV=3
sp|P00966|ASSY_HUMAN	HUMAN	Argininosuccinate synthase OS=Homo sapiens OX=9606 GN=ASS1 PE=1 SV=2
sp|P20702|ITAX_HUMAN	HUMAN	Integrin alpha-X OS=Homo sapiens OX=9606 GN=ITGAX PE=1 SV=3
sp|P61586|RHOA_HUMAN	HUMAN	Transforming protein RhoA OS=Homo sapiens OX=9606 GN=RHOA PE=1 SV=1
sp|P25098|ARBK1_HUMAN	HUMAN	Beta-adrenergic receptor kinase 1 OS=Homo sapiens OX=9606 GN=GRK2 PE=1 SV=2
sp|Q9Y3F4|STRAP_HUMAN	HUMAN	Serine-threonine kinase receptor-associated protein OS=Homo sapiens OX=9606 GN=STRAP PE=1 SV=1
sp|P84077|ARF1_HUMAN	HUMAN	ADP-ribosylation factor 1 OS=Homo sapiens OX=9606 GN=ARF1 PE=1 SV=2
sp|P62424|RL7A_HUMAN	HUMAN	60S ribosomal protein L7a OS=Homo sapiens OX=9606 GN=RPL7A PE=1 SV=2
sp|P54577|SYYC_HUMAN	HUMAN	Tyrosine--tRNA ligase, cytoplasmic OS=Homo sapiens OX=9606 GN=YARS1 PE=1 SV=4
sp|P56192|SYMC_HUMAN	HUMAN	Methionine--tRNA ligase, cytoplasmic OS=Homo sapiens OX=9606 GN=MARS1 PE=1 SV=2
sp|Q9NPY3|C1QR1_HUMAN	HUMAN	Complement component C1q receptor OS=Homo sapiens OX=9606 GN=CD93 PE=1 SV=3
sp|Q14166|TTL12_HUMAN	HUMAN	Tubulin--tyrosine ligase-like protein 12 OS=Homo sapiens OX=9606 GN=TTLL12 PE=1 SV=2

sp|P62249|RS16_HUMAN	HUMAN	40S ribosomal protein S16 OS=Homo sapiens OX=9606 GN=RPS16 PE=1 SV=2
sp|Q01813|PFKAP_HUMAN	HUMAN	ATP-dependent 6-phosphofructokinase, platelet type OS=Homo sapiens OX=9606 GN=PFKP PE=1 SV=2
sp|P01023|A2MG_HUMAN	HUMAN	Alpha-2-macroglobulin OS=Homo sapiens OX=9606 GN=A2M PE=1 SV=3
sp|P61221|ABCE1_HUMAN	HUMAN	ATP-binding cassette sub-family E member 1 OS=Homo sapiens OX=9606 GN=ABCE1 PE=1 SV=1
sp|P63151|2ABA_HUMAN	HUMAN	Serine/threonine-protein phosphatase 2A 55 kDa regulatory subunit B alpha isoform OS=Homo sapiens OX=9606 GN=PPP2R2A PE=1 SV=1
sp|P60660|MYL6_HUMAN	HUMAN	Myosin light polypeptide 6 OS=Homo sapiens OX=9606 GN=MYL6 PE=1 SV=2
sp|P62333|PRS10_HUMAN	HUMAN	26S proteasome regulatory subunit 10B OS=Homo sapiens OX=9606 GN=PSMC6 PE=1 SV=1
sp|P43007|SATT_HUMAN	HUMAN	Neutral amino acid transporter A OS=Homo sapiens OX=9606 GN=SLC1A4 PE=1 SV=1
sp|P61019|RAB2A_HUMAN	HUMAN	Ras-related protein Rab-2A OS=Homo sapiens OX=9606 GN=RAB2A PE=1 SV=1
sp|Q93008|USP9X_HUMAN	HUMAN	Probable ubiquitin carboxyl-terminal hydrolase FAF-X OS=Homo sapiens OX=9606 GN=USP9X PE=1 SV=3
sp|P07737|PROF1_HUMAN	HUMAN	Profilin-1 OS=Homo sapiens OX=9606 GN=PFN1 PE=1 SV=2
sp|P52907|CAZA1_HUMAN	HUMAN	F-actin-capping protein subunit alpha-1 OS=Homo sapiens OX=9606 GN=CAPZA1 PE=1 SV=3
sp|P10321|HLAC_HUMAN	HUMAN	HLA class I histocompatibility antigen, C alpha chain OS=Homo sapiens OX=9606 GN=HLA-C PE=1 SV=3
sp|P51148|RAB5C_HUMAN	HUMAN	Ras-related protein Rab-5C OS=Homo sapiens OX=9606 GN=RAB5C PE=1 SV=2
sp|P69905|HBA_HUMAN	HUMAN	Hemoglobin subunit alpha OS=Homo sapiens OX=9606 GN=HBA1 PE=1 SV=2
sp|P27701|CD82_HUMAN	HUMAN	CD82 antigen OS=Homo sapiens OX=9606 GN=CD82 PE=1 SV=1
sp|P11234|RALB_HUMAN	HUMAN	Ras-related protein Ral-B OS=Homo sapiens OX=9606 GN=RALB PE=1 SV=1
sp|Q86VP6|CAND1_HUMAN	HUMAN	Cullin-associated NEDD8-dissociated protein 1 OS=Homo sapiens OX=9606 GN=CAND1 PE=1 SV=2
sp|Q9BUF5|TBB6_HUMAN	HUMAN	Tubulin beta-6 chain OS=Homo sapiens OX=9606 GN=TUBB6 PE=1 SV=1
sp|O43592|XPOT_HUMAN	HUMAN	Exportin-T OS=Homo sapiens OX=9606 GN=XPOT PE=1 SV=2
sp|P12259|FA5_HUMAN	HUMAN	Coagulation factor V OS=Homo sapiens OX=9606 GN=F5 PE=1 SV=4
sp|P30419|NMT1_HUMAN	HUMAN	Glycylpeptide N-tetradecanoyltransferase 1 OS=Homo sapiens OX=9606 GN=NMT1 PE=1 SV=2
sp|Q06830|PRDX1_HUMAN	HUMAN	Peroxiredoxin-1 OS=Homo sapiens OX=9606 GN=PRDX1 PE=1 SV=1
sp|P20618|PSB1_HUMAN	HUMAN	Proteasome subunit beta type-1 OS=Homo sapiens OX=9606 GN=PSMB1 PE=1 SV=2
sp|P60174|TPIS_HUMAN	HUMAN	Triosephosphate isomerase OS=Homo sapiens OX=9606 GN=TPI1 PE=1 SV=3
sp|Q13347|EIF3I_HUMAN	HUMAN	Eukaryotic translation initiation factor 3 subunit I OS=Homo sapiens OX=9606 GN=EIF3I PE=1 SV=1
sp|P13647|K2C5_HUMAN	HUMAN	Keratin, type II cytoskeletal 5 OS=Homo sapiens OX=9606 GN=KRT5 PE=1 SV=3
sp|O00410|IPO5_HUMAN	HUMAN	Importin-5 OS=Homo sapiens OX=9606 GN=IPO5 PE=1 SV=4
sp|Q5JWF2|GNAS1_HUMAN	HUMAN	Guanine nucleotide-binding protein G(s) subunit alpha isoforms XLas OS=Homo sapiens OX=9606 GN=GNAS PE=1 SV=2
sp|P37802|TAGL2_HUMAN	HUMAN	Transgelin-2 OS=Homo sapiens OX=9606 GN=TAGLN2 PE=1 SV=3

sp|P25788|PSA3_HUMAN	HUMAN	Proteasome subunit alpha type-3 OS=Homo sapiens OX=9606 GN=PSMA3 PE=1 SV=2
sp|P63010|AP2B1_HUMAN	HUMAN	AP-2 complex subunit beta OS=Homo sapiens OX=9606 GN=AP2B1 PE=1 SV=1
sp|P25205|MCM3_HUMAN	HUMAN	DNA replication licensing factor MCM3 OS=Homo sapiens OX=9606 GN=MCM3 PE=1 SV=3
sp|O43684|BUB3_HUMAN	HUMAN	Mitotic checkpoint protein BUB3 OS=Homo sapiens OX=9606 GN=BUB3 PE=1 SV=1
sp|P62979|RS27A_HUMAN	HUMAN	Ubiquitin-40S ribosomal protein S27a OS=Homo sapiens OX=9606 GN=RPS27A PE=1 SV=2
sp|P16402|H13_HUMAN	HUMAN	Histone H1.3 OS=Homo sapiens OX=9606 GN=H1-3 PE=1 SV=2
sp|P12814|ACTN1_HUMAN	HUMAN	Alpha-actinin-1 OS=Homo sapiens OX=9606 GN=ACTN1 PE=1 SV=2
sp|P19105|ML12A_HUMAN	HUMAN	Myosin regulatory light chain 12A OS=Homo sapiens OX=9606 GN=MYL12A PE=1 SV=2
sp|P05362|ICAM1_HUMAN	HUMAN	Intercellular adhesion molecule 1 OS=Homo sapiens OX=9606 GN=ICAM1 PE=1 SV=2
sp|P11413|G6PD_HUMAN	HUMAN	Glucose-6-phosphate 1-dehydrogenase OS=Homo sapiens OX=9606 GN=G6PD PE=1 SV=4
sp|Q96KP1|EXOC2_HUMAN	HUMAN	Exocyst complex component 2 OS=Homo sapiens OX=9606 GN=EXOC2 PE=1 SV=1
sp|Q8NG11|TSN14_HUMAN	HUMAN	Tetraspanin-14 OS=Homo sapiens OX=9606 GN=TSPAN14 PE=1 SV=1
sp|P49189|AL9A1_HUMAN	HUMAN	4-trimethylaminobutyraldehyde dehydrogenase OS=Homo sapiens OX=9606 GN=ALDH9A1 PE=1 SV=3
sp|Q8IZ83|A16A1_HUMAN	HUMAN	Aldehyde dehydrogenase family 16 member A1 OS=Homo sapiens OX=9606 GN=ALDH16A1 PE=1 SV=2
sp|O75340|PDCD6_HUMAN	HUMAN	Programmed cell death protein 6 OS=Homo sapiens OX=9606 GN=PDCD6 PE=1 SV=1
sp|P19623|SPEE_HUMAN	HUMAN	Spermidine synthase OS=Homo sapiens OX=9606 GN=SRM PE=1 SV=1
sp|P00734|THRB_HUMAN	HUMAN	Prothrombin OS=Homo sapiens OX=9606 GN=F2 PE=1 SV=2
sp|O00429|DNM1L_HUMAN	HUMAN	Dynamin-1-like protein OS=Homo sapiens OX=9606 GN=DNM1L PE=1 SV=2
sp|P36578|RL4_HUMAN	HUMAN	60S ribosomal protein L4 OS=Homo sapiens OX=9606 GN=RPL4 PE=1 SV=5
sp|P22392|NDKB_HUMAN	HUMAN	Nucleoside diphosphate kinase B OS=Homo sapiens OX=9606 GN=NME2 PE=1 SV=1
sp|P11388|TOP2A_HUMAN	HUMAN	DNA topoisomerase 2-alpha OS=Homo sapiens OX=9606 GN=TOP2A PE=1 SV=3
sp|P61163|ACTZ_HUMAN	HUMAN	Alpha-centractin OS=Homo sapiens OX=9606 GN=ACTR1A PE=1 SV=1
sp|Q9P2B2|FPRP_HUMAN	HUMAN	Prostaglandin F2 receptor negative regulator OS=Homo sapiens OX=9606 GN=PTGFRN PE=1 SV=2
sp|Q02790|FKBP4_HUMAN	HUMAN	Peptidyl-prolyl cis-trans isomerase FKBP4 OS=Homo sapiens OX=9606 GN=FKBP4 PE=1 SV=3
sp|P00491|PNPH_HUMAN	HUMAN	Purine nucleoside phosphorylase OS=Homo sapiens OX=9606 GN=PNP PE=1 SV=2
sp|P05198|IF2A_HUMAN	HUMAN	Eukaryotic translation initiation factor 2 subunit 1 OS=Homo sapiens OX=9606 GN=EIF2S1 PE=1 SV=3
sp|Q15435|PP1R7_HUMAN	HUMAN	Protein phosphatase 1 regulatory subunit 7 OS=Homo sapiens OX=9606 GN=PPP1R7 PE=1 SV=1
sp|P46459|NSF_HUMAN	HUMAN	Vesicle-fusing ATPase OS=Homo sapiens OX=9606 GN=NSF PE=1 SV=3
sp|P30050|RL12_HUMAN	HUMAN	60S ribosomal protein L12 OS=Homo sapiens OX=9606 GN=RPL12 PE=1 SV=1
sp|P21281|VATB2_HUMAN	HUMAN	V-type proton ATPase subunit B, brain isoform OS=Homo sapiens OX=9606 GN=ATP6V1B2 PE=1 SV=3
sp|P52566|GDIR2_HUMAN	HUMAN	Rho GDP-dissociation inhibitor 2 OS=Homo sapiens OX=9606 GN=ARHGDIB PE=1 SV=3

sp|Q8IYI6|EXOC8_HUMAN	HUMAN	Exocyst complex component 8 OS=Homo sapiens OX=9606 GN=EXOC8 PE=1 SV=2
sp|P28066|PSA5_HUMAN	HUMAN	Proteasome subunit alpha type-5 OS=Homo sapiens OX=9606 GN=PSMA5 PE=1 SV=3
sp|P02533|K1C14_HUMAN	HUMAN	Keratin, type I cytoskeletal 14 OS=Homo sapiens OX=9606 GN=KRT14 PE=1 SV=4
sp|P10909|CLUS_HUMAN	HUMAN	Clusterin OS=Homo sapiens OX=9606 GN=CLU PE=1 SV=1
sp|Q14956|GPNMB_HUMAN	HUMAN	Transmembrane glycoprotein NMB OS=Homo sapiens OX=9606 GN=GPNMB PE=1 SV=2
sp|P53999|TCP4_HUMAN	HUMAN	Activated RNA polymerase II transcriptional coactivator p15 OS=Homo sapiens OX=9606 GN=SUB1 PE=1 SV=3
sp|P34932|HSP74_HUMAN	HUMAN	Heat shock 70 kDa protein 4 OS=Homo sapiens OX=9606 GN=HSPA4 PE=1 SV=4
sp|P62888|RL30_HUMAN	HUMAN	60S ribosomal protein L30 OS=Homo sapiens OX=9606 GN=RPL30 PE=1 SV=2
sp|O00487|PSDE_HUMAN	HUMAN	26S proteasome non-ATPase regulatory subunit 14 OS=Homo sapiens OX=9606 GN=PSMD14 PE=1 SV=1
sp|O00231|PSD11_HUMAN	HUMAN	26S proteasome non-ATPase regulatory subunit 11 OS=Homo sapiens OX=9606 GN=PSMD11 PE=1 SV=3
sp|O60645|EXOC3_HUMAN	HUMAN	Exocyst complex component 3 OS=Homo sapiens OX=9606 GN=EXOC3 PE=1 SV=3
sp|P04792|HSPB1_HUMAN	HUMAN	Heat shock protein beta-1 OS=Homo sapiens OX=9606 GN=HSPB1 PE=1 SV=2
sp|P62277|RS13_HUMAN	HUMAN	40S ribosomal protein S13 OS=Homo sapiens OX=9606 GN=RPS13 PE=1 SV=2
sp|P10768|ESTD_HUMAN	HUMAN	S-formylglutathione hydrolase OS=Homo sapiens OX=9606 GN=ESD PE=1 SV=2
sp|Q14344|GNA13_HUMAN	HUMAN	Guanine nucleotide-binding protein subunit alpha-13 OS=Homo sapiens OX=9606 GN=GNA13 PE=1 SV=2
sp|P25786|PSA1_HUMAN	HUMAN	Proteasome subunit alpha type-1 OS=Homo sapiens OX=9606 GN=PSMA1 PE=1 SV=1
sp|P55263|ADK_HUMAN	HUMAN	Adenosine kinase OS=Homo sapiens OX=9606 GN=ADK PE=1 SV=2
sp|Q15233|NONO_HUMAN	HUMAN	Non-POU domain-containing octamer-binding protein OS=Homo sapiens OX=9606 GN=NONO PE=1 SV=4
sp|P62316|SMD2_HUMAN	HUMAN	Small nuclear ribonucleoprotein Sm D2 OS=Homo sapiens OX=9606 GN=SNRPD2 PE=1 SV=1
sp|P68366|TBA4A_HUMAN	HUMAN	Tubulin alpha-4A chain OS=Homo sapiens OX=9606 GN=TUBA4A PE=1 SV=1
sp|P20701|ITAL_HUMAN	HUMAN	Integrin alpha-L OS=Homo sapiens OX=9606 GN=ITGAL PE=1 SV=3
sp|P04083|ANXA1_HUMAN	HUMAN	Annexin A1 OS=Homo sapiens OX=9606 GN=ANXA1 PE=1 SV=2
sp|P26373|RL13_HUMAN	HUMAN	60S ribosomal protein L13 OS=Homo sapiens OX=9606 GN=RPL13 PE=1 SV=4
sp|P20073|ANXA7_HUMAN	HUMAN	Annexin A7 OS=Homo sapiens OX=9606 GN=ANXA7 PE=1 SV=3
sp|O14828|SCAM3_HUMAN	HUMAN	Secretory carrier-associated membrane protein 3 OS=Homo sapiens OX=9606 GN=SCAMP3 PE=1 SV=3
sp|O15144|ARPC2_HUMAN	HUMAN	Actin-related protein 2/3 complex subunit 2 OS=Homo sapiens OX=9606 GN=ARPC2 PE=1 SV=1
sp|P09525|ANXA4_HUMAN	HUMAN	Annexin A4 OS=Homo sapiens OX=9606 GN=ANXA4 PE=1 SV=4
sp|P33991|MCM4_HUMAN	HUMAN	DNA replication licensing factor MCM4 OS=Homo sapiens OX=9606 GN=MCM4 PE=1 SV=5
sp|P16070|CD44_HUMAN	HUMAN	CD44 antigen OS=Homo sapiens OX=9606 GN=CD44 PE=1 SV=3
sp|P61978|HNRPK_HUMAN	HUMAN	Heterogeneous nuclear ribonucleoprotein K OS=Homo sapiens OX=9606 GN=HNRNPK PE=1 SV=1

sp|Q99829|CPNE1_HUMAN	HUMAN	Copine-1 OS=Homo sapiens OX=9606 GN=CPNE1 PE=1 SV=1
sp|P01111|RASN_HUMAN	HUMAN	GTPase NRas OS=Homo sapiens OX=9606 GN=NRAS PE=1 SV=1
sp|P36955|PEDF_HUMAN	HUMAN	Pigment epithelium-derived factor OS=Homo sapiens OX=9606 GN=SERPINF1 PE=1 SV=4
sp|P35998|PRS7_HUMAN	HUMAN	26S proteasome regulatory subunit 7 OS=Homo sapiens OX=9606 GN=PSMC2 PE=1 SV=3
sp|Q9ULV4|COR1C_HUMAN	HUMAN	Coronin-1C OS=Homo sapiens OX=9606 GN=CORO1C PE=1 SV=1
sp|Q9BWD1|THIC_HUMAN	HUMAN	Acetyl-CoA acetyltransferase, cytosolic OS=Homo sapiens OX=9606 GN=ACAT2 PE=1 SV=2
sp|P67809|YBOX1_HUMAN	HUMAN	Y-box-binding protein 1 OS=Homo sapiens OX=9606 GN=YBX1 PE=1 SV=3
sp|P61106|RAB14_HUMAN	HUMAN	Ras-related protein Rab-14 OS=Homo sapiens OX=9606 GN=RAB14 PE=1 SV=4
sp|Q09666|AHNK_HUMAN	HUMAN	Neuroblast differentiation-associated protein AHNAK OS=Homo sapiens OX=9606 GN=AHNAK PE=1 SV=2
sp|O00154|BACH_HUMAN	HUMAN	Cytosolic acyl coenzyme A thioester hydrolase OS=Homo sapiens OX=9606 GN=ACOT7 PE=1 SV=3
sp|Q92769|HDAC2_HUMAN	HUMAN	Histone deacetylase 2 OS=Homo sapiens OX=9606 GN=HDAC2 PE=1 SV=2
sp|P20340|RAB6A_HUMAN	HUMAN	Ras-related protein Rab-6A OS=Homo sapiens OX=9606 GN=RAB6A PE=1 SV=3
sp|P59998|ARPC4_HUMAN	HUMAN	Actin-related protein 2/3 complex subunit 4 OS=Homo sapiens OX=9606 GN=ARPC4 PE=1 SV=3
sp|P62314|SMD1_HUMAN	HUMAN	Small nuclear ribonucleoprotein Sm D1 OS=Homo sapiens OX=9606 GN=SNRPD1 PE=1 SV=1
sp|Q13418|ILK_HUMAN	HUMAN	Integrin-linked protein kinase OS=Homo sapiens OX=9606 GN=ILK PE=1 SV=2
sp|P22102|PUR2_HUMAN	HUMAN	Trifunctional purine biosynthetic protein adenosine-3 OS=Homo sapiens OX=9606 GN=GART PE=1 SV=1
sp|P17655|CAN2_HUMAN	HUMAN	Calpain-2 catalytic subunit OS=Homo sapiens OX=9606 GN=CAPN2 PE=1 SV=6
sp|P28482|MK01_HUMAN	HUMAN	Mitogen-activated protein kinase 1 OS=Homo sapiens OX=9606 GN=MAPK1 PE=1 SV=3
sp|O00471|EXOC5_HUMAN	HUMAN	Exocyst complex component 5 OS=Homo sapiens OX=9606 GN=EXOC5 PE=1 SV=1
sp|Q5VW32|BROX_HUMAN	HUMAN	BRO1 domain-containing protein BROX OS=Homo sapiens OX=9606 GN=BROX PE=1 SV=1
sp|Q96SB4|SRPK1_HUMAN	HUMAN	SRSF protein kinase 1 OS=Homo sapiens OX=9606 GN=SRPK1 PE=1 SV=2
sp|Q6UWD8|CP054_HUMAN	HUMAN	Transmembrane protein C16orf54 OS=Homo sapiens OX=9606 GN=C16orf54 PE=1 SV=1
sp|P61006|RAB8A_HUMAN	HUMAN	Ras-related protein Rab-8A OS=Homo sapiens OX=9606 GN=RAB8A PE=1 SV=1
sp|P17812|PYRG1_HUMAN	HUMAN	CTP synthase 1 OS=Homo sapiens OX=9606 GN=CTPS1 PE=1 SV=2
sp|Q9NTK5|OLA1_HUMAN	HUMAN	Obg-like ATPase 1 OS=Homo sapiens OX=9606 GN=OLA1 PE=1 SV=2
sp|P61225|RAP2B_HUMAN	HUMAN	Ras-related protein Rap-2b OS=Homo sapiens OX=9606 GN=RAP2B PE=1 SV=1
sp|Q12913|PTPRJ_HUMAN	HUMAN	Receptor-type tyrosine-protein phosphatase eta OS=Homo sapiens OX=9606 GN=PTPRJ PE=1 SV=3
sp|O43390|HNRPR_HUMAN	HUMAN	Heterogeneous nuclear ribonucleoprotein R OS=Homo sapiens OX=9606 GN=HNRNPR PE=1 SV=1
sp|P06730|IF4E_HUMAN	HUMAN	Eukaryotic translation initiation factor 4E OS=Homo sapiens OX=9606 GN=EIF4E PE=1 SV=2
sp|P13489|RINI_HUMAN	HUMAN	Ribonuclease inhibitor OS=Homo sapiens OX=9606 GN=RNH1 PE=1 SV=2
sp|P08243|ASNS_HUMAN	HUMAN	Asparagine synthetase [glutamine-hydrolyzing] OS=Homo sapiens OX=9606 GN=ASNS PE=1 SV=4

sp|P60953|CDC42_HUMAN	HUMAN	Cell division control protein 42 homolog OS=Homo sapiens OX=9606 GN=CDC42 PE=1 SV=2
sp|P84095|RHOG_HUMAN	HUMAN	Rho-related GTP-binding protein RhoG OS=Homo sapiens OX=9606 GN=RHOG PE=1 SV=1
sp|Q96DI7|SNR40_HUMAN	HUMAN	U5 small nuclear ribonucleoprotein 40 kDa protein OS=Homo sapiens OX=9606 GN=SNRNP40 PE=1 SV=1
sp|Q8WWI5|CTL1_HUMAN	HUMAN	Choline transporter-like protein 1 OS=Homo sapiens OX=9606 GN=SLC44A1 PE=1 SV=1
sp|Q8IV08|PLD3_HUMAN	HUMAN	5'-3' exonuclease PLD3 OS=Homo sapiens OX=9606 GN=PLD3 PE=1 SV=1
sp|P51884|LUM_HUMAN	HUMAN	Lumican OS=Homo sapiens OX=9606 GN=LUM PE=1 SV=2
sp|P01730|CD4_HUMAN	HUMAN	T-cell surface glycoprotein CD4 OS=Homo sapiens OX=9606 GN=CD4 PE=1 SV=1
sp|P48556|PSMD8_HUMAN	HUMAN	26S proteasome non-ATPase regulatory subunit 8 OS=Homo sapiens OX=9606 GN=PSMD8 PE=1 SV=2
sp|P61247|RS3A_HUMAN	HUMAN	40S ribosomal protein S3a OS=Homo sapiens OX=9606 GN=RPS3A PE=1 SV=2
sp|P50914|RL14_HUMAN	HUMAN	60S ribosomal protein L14 OS=Homo sapiens OX=9606 GN=RPL14 PE=1 SV=4
sp|P62280|RS11_HUMAN	HUMAN	40S ribosomal protein S11 OS=Homo sapiens OX=9606 GN=RPS11 PE=1 SV=3
sp|P08246|ELNE_HUMAN	HUMAN	Neutrophil elastase OS=Homo sapiens OX=9606 GN=ELANE PE=1 SV=1
sp|Q02543|RL18A_HUMAN	HUMAN	60S ribosomal protein L18a OS=Homo sapiens OX=9606 GN=RPL18A PE=1 SV=2
sp|P06493|CDK1_HUMAN	HUMAN	Cyclin-dependent kinase 1 OS=Homo sapiens OX=9606 GN=CDK1 PE=1 SV=3
sp|P08754|GNAI3_HUMAN	HUMAN	Guanine nucleotide-binding protein G(i) subunit alpha OS=Homo sapiens OX=9606 GN=GNAI3 PE=1 SV=3
sp|Q71DI3|H32_HUMAN	HUMAN	Histone H3.2 OS=Homo sapiens OX=9606 GN=HIST2H3A PE=1 SV=3
sp|P28062|PSB8_HUMAN	HUMAN	Proteasome subunit beta type-8 OS=Homo sapiens OX=9606 GN=PSMB8 PE=1 SV=3
sp|O60488|ACSL4_HUMAN	HUMAN	Long-chain-fatty-acid--CoA ligase 4 OS=Homo sapiens OX=9606 GN=ACSL4 PE=1 SV=2
sp|P62258|1433E_HUMAN	HUMAN	14-3-3 protein epsilon OS=Homo sapiens OX=9606 GN=YWHAE PE=1 SV=1
sp|Q02878|RL6_HUMAN	HUMAN	60S ribosomal protein L6 OS=Homo sapiens OX=9606 GN=RPL6 PE=1 SV=3
sp|P62851|RS25_HUMAN	HUMAN	40S ribosomal protein S25 OS=Homo sapiens OX=9606 GN=RPS25 PE=1 SV=1
sp|P10809|CH60_HUMAN	HUMAN	60 kDa heat shock protein, mitochondrial OS=Homo sapiens OX=9606 GN=HSPD1 PE=1 SV=2
sp|P14550|AK1A1_HUMAN	HUMAN	Aldo-keto reductase family 1 member A1 OS=Homo sapiens OX=9606 GN=AKR1A1 PE=1 SV=3
sp|Q07020|RL18_HUMAN	HUMAN	60S ribosomal protein L18 OS=Homo sapiens OX=9606 GN=RPL18 PE=1 SV=2
sp|P20645|MPRD_HUMAN	HUMAN	Cation-dependent mannose-6-phosphate receptor OS=Homo sapiens OX=9606 GN=M6PR PE=1 SV=1
sp|O75531|BAF_HUMAN	HUMAN	Barrier-to-autointegration factor OS=Homo sapiens OX=9606 GN=BANF1 PE=1 SV=1
sp|P61289|PSME3_HUMAN	HUMAN	Proteasome activator complex subunit 3 OS=Homo sapiens OX=9606 GN=PSME3 PE=1 SV=1
sp|P62244|RS15A_HUMAN	HUMAN	40S ribosomal protein S15a OS=Homo sapiens OX=9606 GN=RPS15A PE=1 SV=2
sp|P06132|DCUP_HUMAN	HUMAN	Uroporphyrinogen decarboxylase OS=Homo sapiens OX=9606 GN=UROD PE=1 SV=2
sp|Q9GZY6|NTAL_HUMAN	HUMAN	Linker for activation of T-cells family member 2 OS=Homo sapiens OX=9606 GN=LAT2 PE=1 SV=1
sp|P78417|GSTO1_HUMAN	HUMAN	Glutathione S-transferase omega-1 OS=Homo sapiens OX=9606 GN=GSTO1 PE=1 SV=2

sp|Q96A65|EXOC4_HUMAN	HUMAN	Exocyst complex component 4 OS=Homo sapiens OX=9606 GN=EXOC4 PE=1 SV=1
sp|P07996|TSP1_HUMAN	HUMAN	Thrombospondin-1 OS=Homo sapiens OX=9606 GN=THBS1 PE=1 SV=2
sp|P18621|RL17_HUMAN	HUMAN	60S ribosomal protein L17 OS=Homo sapiens OX=9606 GN=RPL17 PE=1 SV=3
sp|Q99805|TM9S2_HUMAN	HUMAN	Transmembrane 9 superfamily member 2 OS=Homo sapiens OX=9606 GN=TM9SF2 PE=1 SV=1
sp|P11166|GTR1_HUMAN	HUMAN	Solute carrier family 2, facilitated glucose transporter member 1 OS=Homo sapiens OX=9606 GN=SLC2A1 PE=1 SV=2
sp|P16401|H15_HUMAN	HUMAN	Histone H1.5 OS=Homo sapiens OX=9606 GN=H1-5 PE=1 SV=3
sp|Q13451|FKBP5_HUMAN	HUMAN	Peptidyl-prolyl cis-trans isomerase FKBP5 OS=Homo sapiens OX=9606 GN=FKBP5 PE=1 SV=2
sp|O43865|SAHH2_HUMAN	HUMAN	S-adenosylhomocysteine hydrolase-like protein 1 OS=Homo sapiens OX=9606 GN=AHCYL1 PE=1 SV=2
sp|O14744|ANM5_HUMAN	HUMAN	Protein arginine N-methyltransferase 5 OS=Homo sapiens OX=9606 GN=PRMT5 PE=1 SV=4
sp|P29350|PTN6_HUMAN	HUMAN	Tyrosine-protein phosphatase non-receptor type 6 OS=Homo sapiens OX=9606 GN=PTPN6 PE=1 SV=1
sp|P62191|PRS4_HUMAN	HUMAN	26S proteasome regulatory subunit 4 OS=Homo sapiens OX=9606 GN=PSMC1 PE=1 SV=1
sp|P19440|GGT1_HUMAN	HUMAN	Glutathione hydrolase 1 proenzyme OS=Homo sapiens OX=9606 GN=GGT1 PE=1 SV=2
sp|Q9H9T3|ELP3_HUMAN	HUMAN	Elongator complex protein 3 OS=Homo sapiens OX=9606 GN=ELP3 PE=1 SV=2
sp|P40429|RL13A_HUMAN	HUMAN	60S ribosomal protein L13a OS=Homo sapiens OX=9606 GN=RPL13A PE=1 SV=2
sp|Q9Y6G9|DC1L1_HUMAN	HUMAN	Cytoplasmic dynein 1 light intermediate chain 1 OS=Homo sapiens OX=9606 GN=DYNC1LI1 PE=1 SV=3
sp|P08631|HCK_HUMAN	HUMAN	Tyrosine-protein kinase HCK OS=Homo sapiens OX=9606 GN=HCK PE=1 SV=5
sp|O43324|MCA3_HUMAN	HUMAN	Eukaryotic translation elongation factor 1 epsilon-1 OS=Homo sapiens OX=9606 GN=EEF1E1 PE=1 SV=1
sp|P27694|RFA1_HUMAN	HUMAN	Replication protein A 70 kDa DNA-binding subunit OS=Homo sapiens OX=9606 GN=RPA1 PE=1 SV=2
sp|P11215|ITAM_HUMAN	HUMAN	Integrin alpha-M OS=Homo sapiens OX=9606 GN=ITGAM PE=1 SV=2
sp|Q16222|UAP1_HUMAN	HUMAN	UDP-N-acetylhexosamine pyrophosphorylase OS=Homo sapiens OX=9606 GN=UAP1 PE=1 SV=3
sp|Q8N5I2|ARRD1_HUMAN	HUMAN	Arrestin domain-containing protein 1 OS=Homo sapiens OX=9606 GN=ARRDC1 PE=1 SV=1
sp|Q9NV70|EXOC1_HUMAN	HUMAN	Exocyst complex component 1 OS=Homo sapiens OX=9606 GN=EXOC1 PE=1 SV=4
sp|Q9NQW7|XPP1_HUMAN	HUMAN	Xaa-Pro aminopeptidase 1 OS=Homo sapiens OX=9606 GN=XPNPEP1 PE=1 SV=3
sp|Q6IBS0|TWF2_HUMAN	HUMAN	Twinfilin-2 OS=Homo sapiens OX=9606 GN=TWF2 PE=1 SV=2
sp|P32969|RL9_HUMAN	HUMAN	60S ribosomal protein L9 OS=Homo sapiens OX=9606 GN=RPL9 PE=1 SV=1
sp|Q9Y4L1|HYOU1_HUMAN	HUMAN	Hypoxia up-regulated protein 1 OS=Homo sapiens OX=9606 GN=HYOU1 PE=1 SV=1
sp|P07741|APT_HUMAN	HUMAN	Adenine phosphoribosyltransferase OS=Homo sapiens OX=9606 GN=APRT PE=1 SV=2
sp|P09496|CLCA_HUMAN	HUMAN	Clathrin light chain A OS=Homo sapiens OX=9606 GN=CLTA PE=1 SV=1
sp|P06396|GELS_HUMAN	HUMAN	Gelsolin OS=Homo sapiens OX=9606 GN=GSN PE=1 SV=1
sp|P53985|MOT1_HUMAN	HUMAN	Monocarboxylate transporter 1 OS=Homo sapiens OX=9606 GN=SLC16A1 PE=1 SV=3

sp|Q12905|ILF2_HUMAN	HUMAN	Interleukin enhancer-binding factor 2 OS=Homo sapiens OX=9606 GN=ILF2 PE=1 SV=2
sp|O15143|ARC1B_HUMAN	HUMAN	Actin-related protein 2/3 complex subunit 1B OS=Homo sapiens OX=9606 GN=ARPC1B PE=1 SV=3
sp|P11908|PRPS2_HUMAN	HUMAN	Ribose-phosphate pyrophosphokinase 2 OS=Homo sapiens OX=9606 GN=PRPS2 PE=1 SV=2
sp|O60763|USO1_HUMAN	HUMAN	General vesicular transport factor p115 OS=Homo sapiens OX=9606 GN=USO1 PE=1 SV=2
sp|Q9UJ70|NAGK_HUMAN	HUMAN	N-acetyl-D-glucosamine kinase OS=Homo sapiens OX=9606 GN=NAGK PE=1 SV=4
sp|O95373|IPO7_HUMAN	HUMAN	Importin-7 OS=Homo sapiens OX=9606 GN=IPO7 PE=1 SV=1
sp|P07195|LDHB_HUMAN	HUMAN	L-lactate dehydrogenase B chain OS=Homo sapiens OX=9606 GN=LDHB PE=1 SV=2
sp|P49721|PSB2_HUMAN	HUMAN	Proteasome subunit beta type-2 OS=Homo sapiens OX=9606 GN=PSMB2 PE=1 SV=1
sp|P49915|GUAA_HUMAN	HUMAN	GMP synthase [glutamine-hydrolyzing] OS=Homo sapiens OX=9606 GN=GMPS PE=1 SV=1
sp|P15529|MCP_HUMAN	HUMAN	Membrane cofactor protein OS=Homo sapiens OX=9606 GN=CD46 PE=1 SV=3
sp|P50225|ST1A1_HUMAN	HUMAN	Sulfotransferase 1A1 OS=Homo sapiens OX=9606 GN=SULT1A1 PE=1 SV=3
sp|P68371|TBB4B_HUMAN	HUMAN	Tubulin beta-4B chain OS=Homo sapiens OX=9606 GN=TUBB4B PE=1 SV=1
sp|Q04760|LGUL_HUMAN	HUMAN	Lactoylglutathione lyase OS=Homo sapiens OX=9606 GN=GLO1 PE=1 SV=4
sp|P22234|PUR6_HUMAN	HUMAN	Multifunctional protein ADE2 OS=Homo sapiens OX=9606 GN=PAICS PE=1 SV=3
sp|Q9UBT2|SAE2_HUMAN	HUMAN	SUMO-activating enzyme subunit 2 OS=Homo sapiens OX=9606 GN=UBA2 PE=1 SV=2
sp|Q9Y6M7|S4A7_HUMAN	HUMAN	Sodium bicarbonate cotransporter 3 OS=Homo sapiens OX=9606 GN=SLC4A7 PE=1 SV=2
sp|P61353|RL27_HUMAN	HUMAN	60S ribosomal protein L27 OS=Homo sapiens OX=9606 GN=RPL27 PE=1 SV=2
sp|P08758|ANXA5_HUMAN	HUMAN	Annexin A5 OS=Homo sapiens OX=9606 GN=ANXA5 PE=1 SV=2
sp|Q9Y617|SERC_HUMAN	HUMAN	Phosphoserine aminotransferase OS=Homo sapiens OX=9606 GN=PSAT1 PE=1 SV=2
sp|P62847|RS24_HUMAN	HUMAN	40S ribosomal protein S24 OS=Homo sapiens OX=9606 GN=RPS24 PE=1 SV=1
sp|O94776|MTA2_HUMAN	HUMAN	Metastasis-associated protein MTA2 OS=Homo sapiens OX=9606 GN=MTA2 PE=1 SV=1
sp|P05455|LA_HUMAN	HUMAN	Lupus La protein OS=Homo sapiens OX=9606 GN=SSB PE=1 SV=2
sp|P14324|FPPS_HUMAN	HUMAN	Farnesyl pyrophosphate synthase OS=Homo sapiens OX=9606 GN=FDPS PE=1 SV=4
sp|P51665|PSMD7_HUMAN	HUMAN	26S proteasome non-ATPase regulatory subunit 7 OS=Homo sapiens OX=9606 GN=PSMD7 PE=1 SV=2
sp|P63000|RAC1_HUMAN	HUMAN	Ras-related C3 botulinum toxin substrate 1 OS=Homo sapiens OX=9606 GN=RAC1 PE=1 SV=1
sp|Q96T76|MMS19_HUMAN	HUMAN	MMS19 nucleotide excision repair protein homolog OS=Homo sapiens OX=9606 GN=MMS19 PE=1 SV=2
sp|Q06187|BTK_HUMAN	HUMAN	Tyrosine-protein kinase BTK OS=Homo sapiens OX=9606 GN=BTK PE=1 SV=3
sp|P25789|PSA4_HUMAN	HUMAN	Proteasome subunit alpha type-4 OS=Homo sapiens OX=9606 GN=PSMA4 PE=1 SV=1
sp|P13861|KAP2_HUMAN	HUMAN	cAMP-dependent protein kinase type II-alpha regulatory subunit OS=Homo sapiens OX=9606 GN=PRKAR2A PE=1 SV=2
sp|P04632|CPNS1_HUMAN	HUMAN	Calpain small subunit 1 OS=Homo sapiens OX=9606 GN=CAPNS1 PE=1 SV=1

sp|O43813|LANC1_HUMAN	HUMAN	Glutathione S-transferase LANCL1 OS=Homo sapiens OX=9606 GN=LANCL1 PE=1 SV=1
sp|Q71UI9|H2AV_HUMAN	HUMAN	Histone H2A.V OS=Homo sapiens OX=9606 GN=H2AFV PE=1 SV=3
sp|Q7L2H7|EIF3M_HUMAN	HUMAN	Eukaryotic translation initiation factor 3 subunit M OS=Homo sapiens OX=9606 GN=EIF3M PE=1 SV=1
sp|P56537|IF6_HUMAN	HUMAN	Eukaryotic translation initiation factor 6 OS=Homo sapiens OX=9606 GN=EIF6 PE=1 SV=1
sp|Q9UGN4|CLM8_HUMAN	HUMAN	CMRF35-like molecule 8 OS=Homo sapiens OX=9606 GN=CD300A PE=1 SV=2
sp|P02794|FRIH_HUMAN	HUMAN	Ferritin heavy chain OS=Homo sapiens OX=9606 GN=FTH1 PE=1 SV=2
sp|Q6GTX8|LAIR1_HUMAN	HUMAN	Leukocyte-associated immunoglobulin-like receptor 1 OS=Homo sapiens OX=9606 GN=LAIR1 PE=1 SV=1
sp|Q8WTV0|SCRB1_HUMAN	HUMAN	Scavenger receptor class B member 1 OS=Homo sapiens OX=9606 GN=SCARB1 PE=1 SV=1
sp|Q08431|MFGM_HUMAN	HUMAN	Lactadherin OS=Homo sapiens OX=9606 GN=MFGE8 PE=1 SV=3
sp|P48444|COPD_HUMAN	HUMAN	Coatomer subunit delta OS=Homo sapiens OX=9606 GN=ARCN1 PE=1 SV=1
sp|Q9Y624|JAM1_HUMAN	HUMAN	Junctional adhesion molecule A OS=Homo sapiens OX=9606 GN=F11R PE=1 SV=1
sp|Q9H813|PACC1_HUMAN	HUMAN	Proton-activated chloride channel OS=Homo sapiens OX=9606 GN=PACC1 PE=1 SV=1
sp|Q15833|STXB2_HUMAN	HUMAN	Syntaxin-binding protein 2 OS=Homo sapiens OX=9606 GN=STXBP2 PE=1 SV=2
sp|P62081|RS7_HUMAN	HUMAN	40S ribosomal protein S7 OS=Homo sapiens OX=9606 GN=RPS7 PE=1 SV=1
sp|Q9UNF0|PACN2_HUMAN	HUMAN	Protein kinase C and casein kinase substrate in neurons protein 2 OS=Homo sapiens OX=9606 GN=PACSIN2 PE=1 SV=2
sp|Q9NT62|ATG3_HUMAN	HUMAN	Ubiquitin-like-conjugating enzyme ATG3 OS=Homo sapiens OX=9606 GN=ATG3 PE=1 SV=1
sp|Q14558|KPRA_HUMAN	HUMAN	Phosphoribosyl pyrophosphate synthase-associated protein 1 OS=Homo sapiens OX=9606 GN=PRPSAP1 PE=1 SV=2
sp|P62318|SMD3_HUMAN	HUMAN	Small nuclear ribonucleoprotein Sm D3 OS=Homo sapiens OX=9606 GN=SNRPD3 PE=1 SV=1
sp|P62269|RS18_HUMAN	HUMAN	40S ribosomal protein S18 OS=Homo sapiens OX=9606 GN=RPS18 PE=1 SV=3
sp|P62263|RS14_HUMAN	HUMAN	40S ribosomal protein S14 OS=Homo sapiens OX=9606 GN=RPS14 PE=1 SV=3
sp|P29692|EF1D_HUMAN	HUMAN	Elongation factor 1-delta OS=Homo sapiens OX=9606 GN=EEF1D PE=1 SV=5
sp|P02792|FRIL_HUMAN	HUMAN	Ferritin light chain OS=Homo sapiens OX=9606 GN=FTL PE=1 SV=2
sp|P0DMR1|HNRC4_HUMAN	HUMAN	Heterogeneous nuclear ribonucleoprotein C-like 4 OS=Homo sapiens OX=9606 GN=HNRNPCL4 PE=3 SV=1
sp|P18085|ARF4_HUMAN	HUMAN	ADP-ribosylation factor 4 OS=Homo sapiens OX=9606 GN=ARF4 PE=1 SV=3
sp|P61981|1433G_HUMAN	HUMAN	14-3-3 protein gamma OS=Homo sapiens OX=9606 GN=YWHAG PE=1 SV=2
sp|O00571|DDX3X_HUMAN	HUMAN	ATP-dependent RNA helicase DDX3X OS=Homo sapiens OX=9606 GN=DDX3X PE=1 SV=3
sp|Q9UBQ5|EIF3K_HUMAN	HUMAN	Eukaryotic translation initiation factor 3 subunit K OS=Homo sapiens OX=9606 GN=EIF3K PE=1 SV=1
sp|P11766|ADHX_HUMAN	HUMAN	Alcohol dehydrogenase class-3 OS=Homo sapiens OX=9606 GN=ADH5 PE=1 SV=4
sp|Q7Z4W1|DCXR_HUMAN	HUMAN	L-xylulose reductase OS=Homo sapiens OX=9606 GN=DCXR PE=1 SV=2
sp|P43490|NAMPT_HUMAN	HUMAN	Nicotinamide phosphoribosyltransferase OS=Homo sapiens OX=9606 GN=NAMPT PE=1 SV=1

sp|Q14203|DCTN1_HUMAN	HUMAN	Dynactin subunit 1 OS=Homo sapiens OX=9606 GN=DCTN1 PE=1 SV=3
sp|P28070|PSB4_HUMAN	HUMAN	Proteasome subunit beta type-4 OS=Homo sapiens OX=9606 GN=PSMB4 PE=1 SV=4
sp|P06748|NPM_HUMAN	HUMAN	Nucleophosmin OS=Homo sapiens OX=9606 GN=NPM1 PE=1 SV=2
sp|P04040|CATA_HUMAN	HUMAN	Catalase OS=Homo sapiens OX=9606 GN=CAT PE=1 SV=3
sp|P51809|VAMP7_HUMAN	HUMAN	Vesicle-associated membrane protein 7 OS=Homo sapiens OX=9606 GN=VAMP7 PE=1 SV=3
sp|O75976|CBPD_HUMAN	HUMAN	Carboxypeptidase D OS=Homo sapiens OX=9606 GN=CPD PE=1 SV=2
sp|Q6UXN9|WDR82_HUMAN	HUMAN	WD repeat-containing protein 82 OS=Homo sapiens OX=9606 GN=WDR82 PE=1 SV=1
sp|P43034|LIS1_HUMAN	HUMAN	Platelet-activating factor acetylhydrolase IB subunit alpha OS=Homo sapiens OX=9606 GN=PAFAH1B1 PE=1 SV=2
sp|O75351|VPS4B_HUMAN	HUMAN	Vacuolar protein sorting-associated protein 4B OS=Homo sapiens OX=9606 GN=VPS4B PE=1 SV=2
sp|O14786|NRP1_HUMAN	HUMAN	Neuropilin-1 OS=Homo sapiens OX=9606 GN=NRP1 PE=1 SV=3
sp|P14866|HNRPL_HUMAN	HUMAN	Heterogeneous nuclear ribonucleoprotein L OS=Homo sapiens OX=9606 GN=HNRNPL PE=1 SV=2
sp|P13807|GYS1_HUMAN	HUMAN	Glycogen [starch] synthase, muscle OS=Homo sapiens OX=9606 GN=GYS1 PE=1 SV=2
sp|P62753|RS6_HUMAN	HUMAN	40S ribosomal protein S6 OS=Homo sapiens OX=9606 GN=RPS6 PE=1 SV=1
sp|P52565|GDIR1_HUMAN	HUMAN	Rho GDP-dissociation inhibitor 1 OS=Homo sapiens OX=9606 GN=ARHGDIA PE=1 SV=3
sp|Q13765|NACA_HUMAN	HUMAN	Nascent polypeptide-associated complex subunit alpha OS=Homo sapiens OX=9606 GN=NACA PE=1 SV=1
sp|P30101|PDIA3_HUMAN	HUMAN	Protein disulfide-isomerase A3 OS=Homo sapiens OX=9606 GN=PDIA3 PE=1 SV=4
sp|P17813|EGLN_HUMAN	HUMAN	Endoglin OS=Homo sapiens OX=9606 GN=ENG PE=1 SV=2
sp|P26006|ITA3_HUMAN	HUMAN	Integrin alpha-3 OS=Homo sapiens OX=9606 GN=ITGA3 PE=1 SV=5
sp|P52292|IMA1_HUMAN	HUMAN	Importin subunit alpha-1 OS=Homo sapiens OX=9606 GN=KPNA2 PE=1 SV=1
sp|Q96QD8|S38A2_HUMAN	HUMAN	Sodium-coupled neutral amino acid transporter 2 OS=Homo sapiens OX=9606 GN=SLC38A2 PE=1 SV=2
sp|P54709|AT1B3_HUMAN	HUMAN	Sodium/potassium-transporting ATPase subunit beta-3 OS=Homo sapiens OX=9606 GN=ATP1B3 PE=1 SV=1
sp|P05387|RLA2_HUMAN	HUMAN	60S acidic ribosomal protein P2 OS=Homo sapiens OX=9606 GN=RPLP2 PE=1 SV=1
sp|P62910|RL32_HUMAN	HUMAN	60S ribosomal protein L32 OS=Homo sapiens OX=9606 GN=RPL32 PE=1 SV=2
sp|Q92608|DOCK2_HUMAN	HUMAN	Dedicator of cytokinesis protein 2 OS=Homo sapiens OX=9606 GN=DOCK2 PE=1 SV=2
sp|P83731|RL24_HUMAN	HUMAN	60S ribosomal protein L24 OS=Homo sapiens OX=9606 GN=RPL24 PE=1 SV=1
sp|Q15181|IPYR_HUMAN	HUMAN	Inorganic pyrophosphatase OS=Homo sapiens OX=9606 GN=PPA1 PE=1 SV=2
sp|Q08945|SSRP1_HUMAN	HUMAN	FACT complex subunit SSRP1 OS=Homo sapiens OX=9606 GN=SSRP1 PE=1 SV=1
sp|O14929|HAT1_HUMAN	HUMAN	Histone acetyltransferase type B catalytic subunit OS=Homo sapiens OX=9606 GN=HAT1 PE=1 SV=1
sp|Q8TAG9|EXOC6_HUMAN	HUMAN	Exocyst complex component 6 OS=Homo sapiens OX=9606 GN=EXOC6 PE=1 SV=3
sp|O95819|M4K4_HUMAN	HUMAN	Mitogen-activated protein kinase kinase kinase kinase 4 OS=Homo sapiens OX=9606 GN=MAP4K4 PE=1 SV=2

sp|P12004|PCNA_HUMAN	HUMAN	Proliferating cell nuclear antigen OS=Homo sapiens OX=9606 GN=PCNA PE=1 SV=1
sp|Q9Y5B9|SP16H_HUMAN	HUMAN	FACT complex subunit SPT16 OS=Homo sapiens OX=9606 GN=SUPT16H PE=1 SV=1
sp|Q99447|PCY2_HUMAN	HUMAN	Ethanolamine-phosphate cytidylyltransferase OS=Homo sapiens OX=9606 GN=PCYT2 PE=1 SV=1
sp|O15371|EIF3D_HUMAN	HUMAN	Eukaryotic translation initiation factor 3 subunit D OS=Homo sapiens OX=9606 GN=EIF3D PE=1 SV=1
sp|P22352|GPX3_HUMAN	HUMAN	Glutathione peroxidase 3 OS=Homo sapiens OX=9606 GN=GPX3 PE=1 SV=2
sp|P32970|CD70_HUMAN	HUMAN	CD70 antigen OS=Homo sapiens OX=9606 GN=CD70 PE=1 SV=2
sp|Q96CW1|AP2M1_HUMAN	HUMAN	AP-2 complex subunit mu OS=Homo sapiens OX=9606 GN=AP2M1 PE=1 SV=2
sp|P19823|ITIH2_HUMAN	HUMAN	Inter-alpha-trypsin inhibitor heavy chain H2 OS=Homo sapiens OX=9606 GN=ITIH2 PE=1 SV=2
sp|P11169|GTR3_HUMAN	HUMAN	Solute carrier family 2, facilitated glucose transporter member 3 OS=Homo sapiens OX=9606 GN=SLC2A3 PE=1 SV=1
sp|P62879|GBB2_HUMAN	HUMAN	Guanine nucleotide-binding protein G(I)/G(S)/G(T) subunit beta-2 OS=Homo sapiens OX=9606 GN=GNB2 PE=1 SV=3
sp|P27707|DCK_HUMAN	HUMAN	Deoxycytidine kinase OS=Homo sapiens OX=9606 GN=DCK PE=1 SV=1
sp|Q9NXR7|BABA2_HUMAN	HUMAN	BRISC and BRCA1-A complex member 2 OS=Homo sapiens OX=9606 GN=BABAM2 PE=1 SV=2
sp|P51812|KS6A3_HUMAN	HUMAN	Ribosomal protein S6 kinase alpha-3 OS=Homo sapiens OX=9606 GN=RPS6KA3 PE=1 SV=1
sp|Q9Y315|DEOC_HUMAN	HUMAN	Deoxyribose-phosphate aldolase OS=Homo sapiens OX=9606 GN=DERA PE=1 SV=2
sp|Q15366|PCBP2_HUMAN	HUMAN	Poly(rC)-binding protein 2 OS=Homo sapiens OX=9606 GN=PCBP2 PE=1 SV=1
sp|Q8NC42|RN149_HUMAN	HUMAN	E3 ubiquitin-protein ligase RNF149 OS=Homo sapiens OX=9606 GN=RNF149 PE=2 SV=2
sp|Q9UBQ0|VPS29_HUMAN	HUMAN	Vacuolar protein sorting-associated protein 29 OS=Homo sapiens OX=9606 GN=VPS29 PE=1 SV=1
sp|Q15019|SEPT2_HUMAN	HUMAN	Septin-2 OS=Homo sapiens OX=9606 GN=SEPTIN2 PE=1 SV=1
sp|Q92597|NDRG1_HUMAN	HUMAN	Protein NDRG1 OS=Homo sapiens OX=9606 GN=NDRG1 PE=1 SV=1
sp|Q9Y3U8|RL36_HUMAN	HUMAN	60S ribosomal protein L36 OS=Homo sapiens OX=9606 GN=RPL36 PE=1 SV=3
sp|P46782|RS5_HUMAN	HUMAN	40S ribosomal protein S5 OS=Homo sapiens OX=9606 GN=RPS5 PE=1 SV=4
sp|Q16181|SEPT7_HUMAN	HUMAN	Septin-7 OS=Homo sapiens OX=9606 GN=SEPTIN7 PE=1 SV=2
sp|O75695|XRP2_HUMAN	HUMAN	Protein XRP2 OS=Homo sapiens OX=9606 GN=RP2 PE=1 SV=4
sp|P18433|PTPRA_HUMAN	HUMAN	Receptor-type tyrosine-protein phosphatase alpha OS=Homo sapiens OX=9606 GN=PTPRA PE=1 SV=3
sp|Q9BZQ8|NIBA1_HUMAN	HUMAN	Protein Niban 1 OS=Homo sapiens OX=9606 GN=NIBAN1 PE=1 SV=1
sp|Q9GZS3|WDR61_HUMAN	HUMAN	WD repeat-containing protein 61 OS=Homo sapiens OX=9606 GN=WDR61 PE=1 SV=1
sp|Q86Y82|STX12_HUMAN	HUMAN	Syntaxin-12 OS=Homo sapiens OX=9606 GN=STX12 PE=1 SV=1
sp|Q92499|DDX1_HUMAN	HUMAN	ATP-dependent RNA helicase DDX1 OS=Homo sapiens OX=9606 GN=DDX1 PE=1 SV=2
sp|Q06210|GFPT1_HUMAN	HUMAN	Glutamine--fructose-6-phosphate aminotransferase [isomerizing] 1 OS=Homo sapiens OX=9606 GN=GFPT1 PE=1 SV=3

sp|Q09028|RBBP4_HUMAN	HUMAN	Histone-binding protein RBBP4 OS=Homo sapiens OX=9606 GN=RBBP4 PE=1 SV=3
sp|Q9UI12|VATH_HUMAN	HUMAN	V-type proton ATPase subunit H OS=Homo sapiens OX=9606 GN=ATP6V1H PE=1 SV=1
sp|O75367|H2AY_HUMAN	HUMAN	Core histone macro-H2A.1 OS=Homo sapiens OX=9606 GN=MACROH2A1 PE=1 SV=4
sp|O00203|AP3B1_HUMAN	HUMAN	AP-3 complex subunit beta-1 OS=Homo sapiens OX=9606 GN=AP3B1 PE=1 SV=3
sp|P09429|HMGB1_HUMAN	HUMAN	High mobility group protein B1 OS=Homo sapiens OX=9606 GN=HMGB1 PE=1 SV=3
sp|O60341|KDM1A_HUMAN	HUMAN	Lysine-specific histone demethylase 1A OS=Homo sapiens OX=9606 GN=KDM1A PE=1 SV=2
sp|Q16531|DDB1_HUMAN	HUMAN	DNA damage-binding protein 1 OS=Homo sapiens OX=9606 GN=DDB1 PE=1 SV=1
sp|P14923|PLAK_HUMAN	HUMAN	Junction plakoglobin OS=Homo sapiens OX=9606 GN=JUP PE=1 SV=3
sp|O75874|IDHC_HUMAN	HUMAN	Isocitrate dehydrogenase [NADP] cytoplasmic OS=Homo sapiens OX=9606 GN=IDH1 PE=1 SV=2
sp|P63162|RSMN_HUMAN	HUMAN	Small nuclear ribonucleoprotein-associated protein N OS=Homo sapiens OX=9606 GN=SNRPN PE=1 SV=1
sp|P30520|PURA2_HUMAN	HUMAN	Adenylosuccinate synthetase isozyme 2 OS=Homo sapiens OX=9606 GN=ADSS2 PE=1 SV=3
sp|P06753|TPM3_HUMAN	HUMAN	Tropomyosin alpha-3 chain OS=Homo sapiens OX=9606 GN=TPM3 PE=1 SV=2
sp|Q9NR45|SIAS_HUMAN	HUMAN	Sialic acid synthase OS=Homo sapiens OX=9606 GN=NANS PE=1 SV=2
sp|O00160|MYO1F_HUMAN	HUMAN	Unconventional myosin-If OS=Homo sapiens OX=9606 GN=MYO1F PE=1 SV=3
sp|O43172|PRP4_HUMAN	HUMAN	U4/U6 small nuclear ribonucleoprotein Prp4 OS=Homo sapiens OX=9606 GN=PRPF4 PE=1 SV=2
sp|P51570|GALK1_HUMAN	HUMAN	Galactokinase OS=Homo sapiens OX=9606 GN=GALK1 PE=1 SV=1
sp|P62820|RAB1A_HUMAN	HUMAN	Ras-related protein Rab-1A OS=Homo sapiens OX=9606 GN=RAB1A PE=1 SV=3
sp|P25787|PSA2_HUMAN	HUMAN	Proteasome subunit alpha type-2 OS=Homo sapiens OX=9606 GN=PSMA2 PE=1 SV=2
sp|P50148|GNAQ_HUMAN	HUMAN	Guanine nucleotide-binding protein G(q) subunit alpha OS=Homo sapiens OX=9606 GN=GNAQ PE=1 SV=4
sp|O95297|MPZL1_HUMAN	HUMAN	Myelin protein zero-like protein 1 OS=Homo sapiens OX=9606 GN=MPZL1 PE=1 SV=1
sp|P55160|NCKPL_HUMAN	HUMAN	Nck-associated protein 1-like OS=Homo sapiens OX=9606 GN=NCKAP1L PE=1 SV=3
sp|P27635|RL10_HUMAN	HUMAN	60S ribosomal protein L10 OS=Homo sapiens OX=9606 GN=RPL10 PE=1 SV=4
sp|Q15459|SF3A1_HUMAN	HUMAN	Splicing factor 3A subunit 1 OS=Homo sapiens OX=9606 GN=SF3A1 PE=1 SV=1
sp|P16278|BGAL_HUMAN	HUMAN	Beta-galactosidase OS=Homo sapiens OX=9606 GN=GLB1 PE=1 SV=2
sp|Q13303|KCAB2_HUMAN	HUMAN	Voltage-gated potassium channel subunit beta-2 OS=Homo sapiens OX=9606 GN=KCNAB2 PE=1 SV=2
sp|Q9Y678|COPG1_HUMAN	HUMAN	Coatomer subunit gamma-1 OS=Homo sapiens OX=9606 GN=COPG1 PE=1 SV=1
sp|Q13571|LAPM5_HUMAN	HUMAN	Lysosomal-associated transmembrane protein 5 OS=Homo sapiens OX=9606 GN=LAPTM5 PE=1 SV=1
sp|Q15020|SART3_HUMAN	HUMAN	Squamous cell carcinoma antigen recognized by T-cells 3 OS=Homo sapiens OX=9606 GN=SART3 PE=1 SV=1
sp|P61764|STXB1_HUMAN	HUMAN	Syntaxin-binding protein 1 OS=Homo sapiens OX=9606 GN=STXBP1 PE=1 SV=1
sp|P10599|THIO_HUMAN	HUMAN	Thioredoxin OS=Homo sapiens OX=9606 GN=TXN PE=1 SV=3
sp|P51159|RB27A_HUMAN	HUMAN	Ras-related protein Rab-27A OS=Homo sapiens OX=9606 GN=RAB27A PE=1 SV=3

sp|Q9NTJ3|SMC4_HUMAN	HUMAN	Structural maintenance of chromosomes protein 4 OS=Homo sapiens OX=9606 GN=SMC4 PE=1 SV=2
sp|Q13683|ITA7_HUMAN	HUMAN	Integrin alpha-7 OS=Homo sapiens OX=9606 GN=ITGA7 PE=1 SV=3
sp|Q06033|ITIH3_HUMAN	HUMAN	Inter-alpha-trypsin inhibitor heavy chain H3 OS=Homo sapiens OX=9606 GN=ITIH3 PE=1 SV=2
sp|Q9Y6D6|BIG1_HUMAN	HUMAN	Brefeldin A-inhibited guanine nucleotide-exchange protein 1 OS=Homo sapiens OX=9606 GN=ARFGEF1 PE=1 SV=2
sp|P11171|41_HUMAN	HUMAN	Protein 4.1 OS=Homo sapiens OX=9606 GN=EPB41 PE=1 SV=4
sp|P19338|NUCL_HUMAN	HUMAN	Nucleolin OS=Homo sapiens OX=9606 GN=NCL PE=1 SV=3
sp|P42224|STAT1_HUMAN	HUMAN	Signal transducer and activator of transcription 1-alpha/beta OS=Homo sapiens OX=9606 GN=STAT1 PE=1 SV=2
sp|P55735|SEC13_HUMAN	HUMAN	Protein SEC13 homolog OS=Homo sapiens OX=9606 GN=SEC13 PE=1 SV=3
sp|Q8N163|CCAR2_HUMAN	HUMAN	Cell cycle and apoptosis regulator protein 2 OS=Homo sapiens OX=9606 GN=CCAR2 PE=1 SV=2
sp|Q9GZL7|WDR12_HUMAN	HUMAN	Ribosome biogenesis protein WDR12 OS=Homo sapiens OX=9606 GN=WDR12 PE=1 SV=2
sp|O00186|STXB3_HUMAN	HUMAN	Syntaxin-binding protein 3 OS=Homo sapiens OX=9606 GN=STXBP3 PE=1 SV=2
sp|Q13085|ACACA_HUMAN	HUMAN	Acetyl-CoA carboxylase 1 OS=Homo sapiens OX=9606 GN=ACACA PE=1 SV=2
sp|P61313|RL15_HUMAN	HUMAN	60S ribosomal protein L15 OS=Homo sapiens OX=9606 GN=RPL15 PE=1 SV=2
sp|P47756|CAPZB_HUMAN	HUMAN	F-actin-capping protein subunit beta OS=Homo sapiens OX=9606 GN=CAPZB PE=1 SV=4
sp|P43405|KSYK_HUMAN	HUMAN	Tyrosine-protein kinase SYK OS=Homo sapiens OX=9606 GN=SYK PE=1 SV=1
sp|P15924|DESP_HUMAN	HUMAN	Desmoplakin OS=Homo sapiens OX=9606 GN=DSP PE=1 SV=3
sp|Q8IX19|MCEM1_HUMAN	HUMAN	Mast cell-expressed membrane protein 1 OS=Homo sapiens OX=9606 GN=MCEMP1 PE=1 SV=1
sp|P37837|TALDO_HUMAN	HUMAN	Transaldolase OS=Homo sapiens OX=9606 GN=TALDO1 PE=1 SV=2
sp|P07339|CATD_HUMAN	HUMAN	Cathepsin D OS=Homo sapiens OX=9606 GN=CTSD PE=1 SV=1
sp|P30041|PRDX6_HUMAN	HUMAN	Peroxiredoxin-6 OS=Homo sapiens OX=9606 GN=PRDX6 PE=1 SV=3
sp|P08962|CD63_HUMAN	HUMAN	CD63 antigen OS=Homo sapiens OX=9606 GN=CD63 PE=1 SV=2
sp|P61201|CSN2_HUMAN	HUMAN	COP9 signalosome complex subunit 2 OS=Homo sapiens OX=9606 GN=COPS2 PE=1 SV=1
sp|P10155|RO60_HUMAN	HUMAN	60 kDa SS-A/Ro ribonucleoprotein OS=Homo sapiens OX=9606 GN=RO60 PE=1 SV=2
sp|Q13045|FLII_HUMAN	HUMAN	Protein flightless-1 homolog OS=Homo sapiens OX=9606 GN=FLII PE=1 SV=2
sp|P09543|CN37_HUMAN	HUMAN	2',3'-cyclic-nucleotide 3'-phosphodiesterase OS=Homo sapiens OX=9606 GN=CNP PE=1 SV=2
sp|P50552|VASP_HUMAN	HUMAN	Vasodilator-stimulated phosphoprotein OS=Homo sapiens OX=9606 GN=VASP PE=1 SV=3
sp|P62942|FKB1A_HUMAN	HUMAN	Peptidyl-prolyl cis-trans isomerase FKBP1A OS=Homo sapiens OX=9606 GN=FKBP1A PE=1 SV=2
sp|P62917|RL8_HUMAN	HUMAN	60S ribosomal protein L8 OS=Homo sapiens OX=9606 GN=RPL8 PE=1 SV=2
sp|P62913|RL11_HUMAN	HUMAN	60S ribosomal protein L11 OS=Homo sapiens OX=9606 GN=RPL11 PE=1 SV=2
sp|P27361|MK03_HUMAN	HUMAN	Mitogen-activated protein kinase 3 OS=Homo sapiens OX=9606 GN=MAPK3 PE=1 SV=4

sp|Q9BT78|CSN4_HUMAN	HUMAN	COP9 signalosome complex subunit 4 OS=Homo sapiens OX=9606 GN=COPS4 PE=1 SV=1
sp|P62854|RS26_HUMAN	HUMAN	40S ribosomal protein S26 OS=Homo sapiens OX=9606 GN=RPS26 PE=1 SV=3
sp|Q658P3|STEA3_HUMAN	HUMAN	Metalloreductase STEAP3 OS=Homo sapiens OX=9606 GN=STEAP3 PE=1 SV=2
sp|P01889|HLAB_HUMAN	HUMAN	HLA class I histocompatibility antigen, B alpha chain OS=Homo sapiens OX=9606 GN=HLA-B PE=1 SV=3
sp|Q92930|RAB8B_HUMAN	HUMAN	Ras-related protein Rab-8B OS=Homo sapiens OX=9606 GN=RAB8B PE=1 SV=2
sp|P62834|RAP1A_HUMAN	HUMAN	Ras-related protein Rap-1A OS=Homo sapiens OX=9606 GN=RAP1A PE=1 SV=1
sp|Q15836|VAMP3_HUMAN	HUMAN	Vesicle-associated membrane protein 3 OS=Homo sapiens OX=9606 GN=VAMP3 PE=1 SV=3
sp|P46783|RS10_HUMAN	HUMAN	40S ribosomal protein S10 OS=Homo sapiens OX=9606 GN=RPS10 PE=1 SV=1
sp|P31151|S10A7_HUMAN	HUMAN	Protein S100-A7 OS=Homo sapiens OX=9606 GN=S100A7 PE=1 SV=4
sp|Q9BUL8|PDC10_HUMAN	HUMAN	Programmed cell death protein 10 OS=Homo sapiens OX=9606 GN=PDCD10 PE=1 SV=1
sp|Q9BQA1|MEP50_HUMAN	HUMAN	Methylosome protein 50 OS=Homo sapiens OX=9606 GN=WDR77 PE=1 SV=1
sp|Q92522|H1X_HUMAN	HUMAN	Histone H1x OS=Homo sapiens OX=9606 GN=H1FX PE=1 SV=1
sp|Q13277|STX3_HUMAN	HUMAN	Syntaxin-3 OS=Homo sapiens OX=9606 GN=STX3 PE=1 SV=3
sp|Q07666|KHDR1_HUMAN	HUMAN	KH domain-containing, RNA-binding, signal transduction-associated protein 1 OS=Homo sapiens OX=9606 GN=KHDRBS1 PE=1 SV=1
sp|P84098|RL19_HUMAN	HUMAN	60S ribosomal protein L19 OS=Homo sapiens OX=9606 GN=RPL19 PE=1 SV=1
sp|P80723|BASP1_HUMAN	HUMAN	Brain acid soluble protein 1 OS=Homo sapiens OX=9606 GN=BASP1 PE=1 SV=2
sp|P61769|B2MG_HUMAN	HUMAN	Beta-2-microglobulin OS=Homo sapiens OX=9606 GN=B2M PE=1 SV=1
sp|P35268|RL22_HUMAN	HUMAN	60S ribosomal protein L22 OS=Homo sapiens OX=9606 GN=RPL22 PE=1 SV=2
sp|P30273|FCERG_HUMAN	HUMAN	High affinity immunoglobulin epsilon receptor subunit gamma OS=Homo sapiens OX=9606 GN=FCER1G PE=1 SV=1
sp|P28074|PSB5_HUMAN	HUMAN	Proteasome subunit beta type-5 OS=Homo sapiens OX=9606 GN=PSMB5 PE=1 SV=3
sp|P28072|PSB6_HUMAN	HUMAN	Proteasome subunit beta type-6 OS=Homo sapiens OX=9606 GN=PSMB6 PE=1 SV=4
sp|P14174|MIF_HUMAN	HUMAN	Macrophage migration inhibitory factor OS=Homo sapiens OX=9606 GN=MIF PE=1 SV=4
sp|P08670|VIME_HUMAN	HUMAN	Vimentin OS=Homo sapiens OX=9606 GN=VIM PE=1 SV=4
sp|O75608|LYPA1_HUMAN	HUMAN	Acyl-protein thioesterase 1 OS=Homo sapiens OX=9606 GN=LYPLA1 PE=1 SV=1
sp|O15511|ARPC5_HUMAN	HUMAN	Actin-related protein 2/3 complex subunit 5 OS=Homo sapiens OX=9606 GN=ARPC5 PE=1 SV=3
sp|Q16563|SYPL1_HUMAN	HUMAN	Synaptophysin-like protein 1 OS=Homo sapiens OX=9606 GN=SYPL1 PE=1 SV=1
sp|Q9H223|EHD4_HUMAN	HUMAN	EH domain-containing protein 4 OS=Homo sapiens OX=9606 GN=EHD4 PE=1 SV=1
sp|P40926|MDHM_HUMAN	HUMAN	Malate dehydrogenase, mitochondrial OS=Homo sapiens OX=9606 GN=MDH2 PE=1 SV=3
sp|Q9UBV8|PEF1_HUMAN	HUMAN	Peflin OS=Homo sapiens OX=9606 GN=PEF1 PE=1 SV=1
sp|P11021|BIP_HUMAN	HUMAN	Endoplasmic reticulum chaperone BiP OS=Homo sapiens OX=9606 GN=HSPA5 PE=1 SV=2

sp|Q15286|RAB35_HUMAN	HUMAN	Ras-related protein Rab-35 OS=Homo sapiens OX=9606 GN=RAB35 PE=1 SV=1
sp|Q9NQC3|RTN4_HUMAN	HUMAN	Reticulon-4 OS=Homo sapiens OX=9606 GN=RTN4 PE=1 SV=2
sp|P30086|PEBP1_HUMAN	HUMAN	Phosphatidylethanolamine-binding protein 1 OS=Homo sapiens OX=9606 GN=PEBP1 PE=1 SV=3
sp|Q14103|HNRPD_HUMAN	HUMAN	Heterogeneous nuclear ribonucleoprotein D0 OS=Homo sapiens OX=9606 GN=HNRNPD PE=1 SV=1
sp|Q71UM5|RS27L_HUMAN	HUMAN	40S ribosomal protein S27-like OS=Homo sapiens OX=9606 GN=RPS27L PE=1 SV=3
sp|O14579|COPE_HUMAN	HUMAN	Coatomer subunit epsilon OS=Homo sapiens OX=9606 GN=COPE PE=1 SV=3
sp|Q9H4E7|DEFI6_HUMAN	HUMAN	Differentially expressed in FDCP 6 homolog OS=Homo sapiens OX=9606 GN=DEF6 PE=1 SV=1
sp|Q12846|STX4_HUMAN	HUMAN	Syntaxin-4 OS=Homo sapiens OX=9606 GN=STX4 PE=1 SV=2
sp|P60866|RS20_HUMAN	HUMAN	40S ribosomal protein S20 OS=Homo sapiens OX=9606 GN=RPS20 PE=1 SV=1
sp|Q9NQR4|NIT2_HUMAN	HUMAN	Omega-amidase NIT2 OS=Homo sapiens OX=9606 GN=NIT2 PE=1 SV=1
sp|P11233|RALA_HUMAN	HUMAN	Ras-related protein Ral-A OS=Homo sapiens OX=9606 GN=RALA PE=1 SV=1
sp|P46776|RL27A_HUMAN	HUMAN	60S ribosomal protein L27a OS=Homo sapiens OX=9606 GN=RPL27A PE=1 SV=2
sp|P68133|ACTS_HUMAN	HUMAN	Actin, alpha skeletal muscle OS=Homo sapiens OX=9606 GN=ACTA1 PE=1 SV=1
sp|Q9BV40|VAMP8_HUMAN	HUMAN	Vesicle-associated membrane protein 8 OS=Homo sapiens OX=9606 GN=VAMP8 PE=1 SV=1
sp|P61020|RAB5B_HUMAN	HUMAN	Ras-related protein Rab-5B OS=Homo sapiens OX=9606 GN=RAB5B PE=1 SV=1
sp|O15127|SCAM2_HUMAN	HUMAN	Secretory carrier-associated membrane protein 2 OS=Homo sapiens OX=9606 GN=SCAMP2 PE=1 SV=2
sp|P23284|PPIB_HUMAN	HUMAN	Peptidyl-prolyl cis-trans isomerase B OS=Homo sapiens OX=9606 GN=PPIB PE=1 SV=2
sp|P30740|ILEU_HUMAN	HUMAN	Leukocyte elastase inhibitor OS=Homo sapiens OX=9606 GN=SERPINB1 PE=1 SV=1
sp|Q8NBI5|S43A3_HUMAN	HUMAN	Solute carrier family 43 member 3 OS=Homo sapiens OX=9606 GN=SLC43A3 PE=1 SV=2
sp|P00749|UROK_HUMAN	HUMAN	Urokinase-type plasminogen activator OS=Homo sapiens OX=9606 GN=PLAU PE=1 SV=2
sp|P54619|AAKG1_HUMAN	HUMAN	5'-AMP-activated protein kinase subunit gamma-1 OS=Homo sapiens OX=9606 GN=PRKAG1 PE=1 SV=1
sp|Q9HD45|TM9S3_HUMAN	HUMAN	Transmembrane 9 superfamily member 3 OS=Homo sapiens OX=9606 GN=TM9SF3 PE=1 SV=2
sp|O75390|CISY_HUMAN	HUMAN	Citrate synthase, mitochondrial OS=Homo sapiens OX=9606 GN=CS PE=1 SV=2
sp|Q9UL25|RAB21_HUMAN	HUMAN	Ras-related protein Rab-21 OS=Homo sapiens OX=9606 GN=RAB21 PE=1 SV=3
sp|P09382|LEG1_HUMAN	HUMAN	Galectin-1 OS=Homo sapiens OX=9606 GN=LGALS1 PE=1 SV=2
sp|O00764|PDXK_HUMAN	HUMAN	Pyridoxal kinase OS=Homo sapiens OX=9606 GN=PDXK PE=1 SV=1
sp|Q16778|H2B2E_HUMAN	HUMAN	Histone H2B type 2-E OS=Homo sapiens OX=9606 GN=HIST2H2BE PE=1 SV=3
sp|P28838|AMPL_HUMAN	HUMAN	Cytosol aminopeptidase OS=Homo sapiens OX=9606 GN=LAP3 PE=1 SV=3
sp|Q96S97|MYADM_HUMAN	HUMAN	Myeloid-associated differentiation marker OS=Homo sapiens OX=9606 GN=MYADM PE=1 SV=2
sp|P51991|ROA3_HUMAN	HUMAN	Heterogeneous nuclear ribonucleoprotein A3 OS=Homo sapiens OX=9606 GN=HNRNPA3 PE=1 SV=2
sp|Q6RW13|ATRAP_HUMAN	HUMAN	Type-1 angiotensin II receptor-associated protein OS=Homo sapiens OX=9606 GN=AGTRAP PE=1 SV=1

sp|P37268|FDFT_HUMAN	HUMAN	Squalene synthase OS=Homo sapiens OX=9606 GN=FDFT1 PE=1 SV=1
sp|P68871|HBB_HUMAN	HUMAN	Hemoglobin subunit beta OS=Homo sapiens OX=9606 GN=HBB PE=1 SV=2
sp|P02788|TRFL_HUMAN	HUMAN	Lactotransferrin OS=Homo sapiens OX=9606 GN=LTF PE=1 SV=6
sp|Q8IUE6|H2A2B_HUMAN	HUMAN	Histone H2A type 2-B OS=Homo sapiens OX=9606 GN=HIST2H2AB PE=1 SV=3
sp|Q14232|EI2BA_HUMAN	HUMAN	Translation initiation factor eIF-2B subunit alpha OS=Homo sapiens OX=9606 GN=EIF2B1 PE=1 SV=1
sp|Q13148|TADBP_HUMAN	HUMAN	TAR DNA-binding protein 43 OS=Homo sapiens OX=9606 GN=TARDBP PE=1 SV=1
sp|P52272|HNRPM_HUMAN	HUMAN	Heterogeneous nuclear ribonucleoprotein M OS=Homo sapiens OX=9606 GN=HNRNPM PE=1 SV=3
sp|Q00839|HNRPU_HUMAN	HUMAN	Heterogeneous nuclear ribonucleoprotein U OS=Homo sapiens OX=9606 GN=HNRNPU PE=1 SV=6
sp|P49888|ST1E1_HUMAN	HUMAN	Sulfotransferase 1E1 OS=Homo sapiens OX=9606 GN=SULT1E1 PE=1 SV=1
sp|P24158|PRTN3_HUMAN	HUMAN	Myeloblastin OS=Homo sapiens OX=9606 GN=PRTN3 PE=1 SV=3
sp|Q8TBC4|UBA3_HUMAN	HUMAN	NEDD8-activating enzyme E1 catalytic subunit OS=Homo sapiens OX=9606 GN=UBA3 PE=1 SV=2
sp|P08697|A2AP_HUMAN	HUMAN	Alpha-2-antiplasmin OS=Homo sapiens OX=9606 GN=SERPINF2 PE=1 SV=3
sp|P12109|CO6A1_HUMAN	HUMAN	Collagen alpha-1(VI) chain OS=Homo sapiens OX=9606 GN=COL6A1 PE=1 SV=3
sp|Q86X55|CARM1_HUMAN	HUMAN	Histone-arginine methyltransferase CARM1 OS=Homo sapiens OX=9606 GN=CARM1 PE=1 SV=3
sp|Q04695|K1C17_HUMAN	HUMAN	Keratin, type I cytoskeletal 17 OS=Homo sapiens OX=9606 GN=KRT17 PE=1 SV=2
sp|P53602|MVD1_HUMAN	HUMAN	Diphosphomevalonate decarboxylase OS=Homo sapiens OX=9606 GN=MVD PE=1 SV=1
sp|Q13619|CUL4A_HUMAN	HUMAN	Cullin-4A OS=Homo sapiens OX=9606 GN=CUL4A PE=1 SV=3
sp|P22694|KAPCB_HUMAN	HUMAN	cAMP-dependent protein kinase catalytic subunit beta OS=Homo sapiens OX=9606 GN=PRKACB PE=1 SV=2
sp|P15927|RFA2_HUMAN	HUMAN	Replication protein A 32 kDa subunit OS=Homo sapiens OX=9606 GN=RPA2 PE=1 SV=1
sp|Q16539|MK14_HUMAN	HUMAN	Mitogen-activated protein kinase 14 OS=Homo sapiens OX=9606 GN=MAPK14 PE=1 SV=3
sp|P16150|LEUK_HUMAN	HUMAN	Leukosialin OS=Homo sapiens OX=9606 GN=SPN PE=1 SV=1
sp|P09661|RU2A_HUMAN	HUMAN	U2 small nuclear ribonucleoprotein A' OS=Homo sapiens OX=9606 GN=SNRPA1 PE=1 SV=2
sp|Q9H4A6|GOLP3_HUMAN	HUMAN	Golgi phosphoprotein 3 OS=Homo sapiens OX=9606 GN=GOLPH3 PE=1 SV=1
sp|Q9Y639|NPTN_HUMAN	HUMAN	Neuroplastin OS=Homo sapiens OX=9606 GN=NPTN PE=1 SV=2
sp|Q13155|AIMP2_HUMAN	HUMAN	Aminoacyl tRNA synthase complex-interacting multifunctional protein 2 OS=Homo sapiens OX=9606 GN=AIMP2 PE=1 SV=2
sp|Q14240|IF4A2_HUMAN	HUMAN	Eukaryotic initiation factor 4A-II OS=Homo sapiens OX=9606 GN=EIF4A2 PE=1 SV=2
sp|P16949|STMN1_HUMAN	HUMAN	Stathmin OS=Homo sapiens OX=9606 GN=STMN1 PE=1 SV=3
sp|O00560|SDCB1_HUMAN	HUMAN	Syntenin-1 OS=Homo sapiens OX=9606 GN=SDCBP PE=1 SV=1
sp|Q9Y240|CLC11_HUMAN	HUMAN	C-type lectin domain family 11 member A OS=Homo sapiens OX=9606 GN=CLEC11A PE=1 SV=1
sp|P23258|TBG1_HUMAN	HUMAN	Tubulin gamma-1 chain OS=Homo sapiens OX=9606 GN=TUBG1 PE=1 SV=2

sp|P78324|SHPS1_HUMAN	HUMAN	Tyrosine-protein phosphatase non-receptor type substrate 1 OS=Homo sapiens OX=9606 GN=SIRPA PE=1 SV=2
sp|P09417|DHPR_HUMAN	HUMAN	Dihydropteridine reductase OS=Homo sapiens OX=9606 GN=QDPR PE=1 SV=2
sp|Q96FW1|OTUB1_HUMAN	HUMAN	Ubiquitin thioesterase OTUB1 OS=Homo sapiens OX=9606 GN=OTUB1 PE=1 SV=2
sp|O60506|HNRPQ_HUMAN	HUMAN	Heterogeneous nuclear ribonucleoprotein Q OS=Homo sapiens OX=9606 GN=SYNCRIP PE=1 SV=2
sp|Q04917|1433F_HUMAN	HUMAN	14-3-3 protein eta OS=Homo sapiens OX=9606 GN=YWHAH PE=1 SV=4
sp|Q8NFH3|NUP43_HUMAN	HUMAN	Nucleoporin Nup43 OS=Homo sapiens OX=9606 GN=NUP43 PE=1 SV=1
sp|O15431|COPT1_HUMAN	HUMAN	High affinity copper uptake protein 1 OS=Homo sapiens OX=9606 GN=SLC31A1 PE=1 SV=1
sp|Q99733|NP1L4_HUMAN	HUMAN	Nucleosome assembly protein 1-like 4 OS=Homo sapiens OX=9606 GN=NAP1L4 PE=1 SV=1
sp|Q15365|PCBP1_HUMAN	HUMAN	Poly(rC)-binding protein 1 OS=Homo sapiens OX=9606 GN=PCBP1 PE=1 SV=2
sp|Q04721|NOTC2_HUMAN	HUMAN	Neurogenic locus notch homolog protein 2 OS=Homo sapiens OX=9606 GN=NOTCH2 PE=1 SV=3
sp|Q4KMQ2|ANO6_HUMAN	HUMAN	Anoctamin-6 OS=Homo sapiens OX=9606 GN=ANO6 PE=1 SV=2
sp|P15104|GLNA_HUMAN	HUMAN	Glutamine synthetase OS=Homo sapiens OX=9606 GN=GLUL PE=1 SV=4
sp|P62330|ARF6_HUMAN	HUMAN	ADP-ribosylation factor 6 OS=Homo sapiens OX=9606 GN=ARF6 PE=1 SV=2
sp|P49720|PSB3_HUMAN	HUMAN	Proteasome subunit beta type-3 OS=Homo sapiens OX=9606 GN=PSMB3 PE=1 SV=2
sp|P61970|NTF2_HUMAN	HUMAN	Nuclear transport factor 2 OS=Homo sapiens OX=9606 GN=NUTF2 PE=1 SV=1
sp|Q9Y295|DRG1_HUMAN	HUMAN	Developmentally-regulated GTP-binding protein 1 OS=Homo sapiens OX=9606 GN=DRG1 PE=1 SV=1
sp|P25705|ATPA_HUMAN	HUMAN	ATP synthase subunit alpha, mitochondrial OS=Homo sapiens OX=9606 GN=ATP5F1A PE=1 SV=1
sp|Q9ULH0|KDIS_HUMAN	HUMAN	Kinase D-interacting substrate of 220 kDa OS=Homo sapiens OX=9606 GN=KIDINS220 PE=1 SV=3
sp|P25398|RS12_HUMAN	HUMAN	40S ribosomal protein S12 OS=Homo sapiens OX=9606 GN=RPS12 PE=1 SV=3
sp|P09455|RET1_HUMAN	HUMAN	Retinol-binding protein 1 OS=Homo sapiens OX=9606 GN=RBP1 PE=1 SV=2
sp|P18077|RL35A_HUMAN	HUMAN	60S ribosomal protein L35a OS=Homo sapiens OX=9606 GN=RPL35A PE=1 SV=2
sp|P46778|RL21_HUMAN	HUMAN	60S ribosomal protein L21 OS=Homo sapiens OX=9606 GN=RPL21 PE=1 SV=2
sp|P30626|SORCN_HUMAN	HUMAN	Sorcin OS=Homo sapiens OX=9606 GN=SRI PE=1 SV=1
sp|P03973|SLPI_HUMAN	HUMAN	Antileukoproteinase OS=Homo sapiens OX=9606 GN=SLPI PE=1 SV=2
sp|P22732|GTR5_HUMAN	HUMAN	Solute carrier family 2, facilitated glucose transporter member 5 OS=Homo sapiens OX=9606 GN=SLC2A5 PE=1 SV=1
sp|P04004|VTNC_HUMAN	HUMAN	Vitronectin OS=Homo sapiens OX=9606 GN=VTN PE=1 SV=1
sp|P27986|P85A_HUMAN	HUMAN	Phosphatidylinositol 3-kinase regulatory subunit alpha OS=Homo sapiens OX=9606 GN=PIK3R1 PE=1 SV=2
sp|O15031|PLXB2_HUMAN	HUMAN	Plexin-B2 OS=Homo sapiens OX=9606 GN=PLXNB2 PE=1 SV=3
sp|P24941|CDK2_HUMAN	HUMAN	Cyclin-dependent kinase 2 OS=Homo sapiens OX=9606 GN=CDK2 PE=1 SV=2
sp|Q03405|UPAR_HUMAN	HUMAN	Urokinase plasminogen activator surface receptor OS=Homo sapiens OX=9606 GN=PLAUR PE=1 SV=1

sp|P48509|CD151_HUMAN	HUMAN	CD151 antigen OS=Homo sapiens OX=9606 GN=CD151 PE=1 SV=3
sp|P20742|PZP_HUMAN	HUMAN	Pregnancy zone protein OS=Homo sapiens OX=9606 GN=PZP PE=1 SV=4
sp|P15559|NQO1_HUMAN	HUMAN	NAD(P)H dehydrogenase [quinone] 1 OS=Homo sapiens OX=9606 GN=NQO1 PE=1 SV=1
sp|P62906|RL10A_HUMAN	HUMAN	60S ribosomal protein L10a OS=Homo sapiens OX=9606 GN=RPL10A PE=1 SV=2
sp|P31641|SC6A6_HUMAN	HUMAN	Sodium- and chloride-dependent taurine transporter OS=Homo sapiens OX=9606 GN=SLC6A6 PE=1 SV=2
sp|Q15185|TEBP_HUMAN	HUMAN	Prostaglandin E synthase 3 OS=Homo sapiens OX=9606 GN=PTGES3 PE=1 SV=1
sp|P41240|CSK_HUMAN	HUMAN	Tyrosine-protein kinase CSK OS=Homo sapiens OX=9606 GN=CSK PE=1 SV=1
sp|Q10588|BST1_HUMAN	HUMAN	ADP-ribosyl cyclase/cyclic ADP-ribose hydrolase 2 OS=Homo sapiens OX=9606 GN=BST1 PE=1 SV=2
sp|O00567|NOP56_HUMAN	HUMAN	Nucleolar protein 56 OS=Homo sapiens OX=9606 GN=NOP56 PE=1 SV=4
sp|P41227|NAA10_HUMAN	HUMAN	N-alpha-acetyltransferase 10 OS=Homo sapiens OX=9606 GN=NAA10 PE=1 SV=1
sp|Q7L1Q6|BZW1_HUMAN	HUMAN	Basic leucine zipper and W2 domain-containing protein 1 OS=Homo sapiens OX=9606 GN=BZW1 PE=1 SV=1
sp|P08708|RS17_HUMAN	HUMAN	40S ribosomal protein S17 OS=Homo sapiens OX=9606 GN=RPS17 PE=1 SV=2
sp|Q5T4S7|UBR4_HUMAN	HUMAN	E3 ubiquitin-protein ligase UBR4 OS=Homo sapiens OX=9606 GN=UBR4 PE=1 SV=1
sp|Q969U7|PSMG2_HUMAN	HUMAN	Proteasome assembly chaperone 2 OS=Homo sapiens OX=9606 GN=PSMG2 PE=1 SV=1
sp|Q9C0C4|SEM4C_HUMAN	HUMAN	Semaphorin-4C OS=Homo sapiens OX=9606 GN=SEMA4C PE=1 SV=2
sp|P12268|IMDH2_HUMAN	HUMAN	Inosine-5'-monophosphate dehydrogenase 2 OS=Homo sapiens OX=9606 GN=IMPDH2 PE=1 SV=2
sp|Q09161|NCBP1_HUMAN	HUMAN	Nuclear cap-binding protein subunit 1 OS=Homo sapiens OX=9606 GN=NCBP1 PE=1 SV=1
sp|O00161|SNP23_HUMAN	HUMAN	Synaptosomal-associated protein 23 OS=Homo sapiens OX=9606 GN=SNAP23 PE=1 SV=1
sp|Q9Y287|ITM2B_HUMAN	HUMAN	Integral membrane protein 2B OS=Homo sapiens OX=9606 GN=ITM2B PE=1 SV=1
sp|P62491|RB11A_HUMAN	HUMAN	Ras-related protein Rab-11A OS=Homo sapiens OX=9606 GN=RAB11A PE=1 SV=3
sp|Q92616|GCN1_HUMAN	HUMAN	eIF-2-alpha kinase activator GCN1 OS=Homo sapiens OX=9606 GN=GCN1 PE=1 SV=6
sp|Q9H2K8|TAOK3_HUMAN	HUMAN	Serine/threonine-protein kinase TAO3 OS=Homo sapiens OX=9606 GN=TAOK3 PE=1 SV=2
sp|Q15493|RGN_HUMAN	HUMAN	Regucalcin OS=Homo sapiens OX=9606 GN=RGN PE=1 SV=1
sp|P23142|FBLN1_HUMAN	HUMAN	Fibulin-1 OS=Homo sapiens OX=9606 GN=FBLN1 PE=1 SV=4
sp|Q96QU8|XPO6_HUMAN	HUMAN	Exportin-6 OS=Homo sapiens OX=9606 GN=XPO6 PE=1 SV=1
sp|Q01469|FABP5_HUMAN	HUMAN	Fatty acid-binding protein 5 OS=Homo sapiens OX=9606 GN=FABP5 PE=1 SV=3
sp|P61088|UBE2N_HUMAN	HUMAN	Ubiquitin-conjugating enzyme E2 N OS=Homo sapiens OX=9606 GN=UBE2N PE=1 SV=1
sp|P17096|HMGA1_HUMAN	HUMAN	High mobility group protein HMG-I/HMG-Y OS=Homo sapiens OX=9606 GN=HMGA1 PE=1 SV=3
sp|O76021|RL1D1_HUMAN	HUMAN	Ribosomal L1 domain-containing protein 1 OS=Homo sapiens OX=9606 GN=RSL1D1 PE=1 SV=3
sp|O15126|SCAM1_HUMAN	HUMAN	Secretory carrier-associated membrane protein 1 OS=Homo sapiens OX=9606 GN=SCAMP1 PE=1 SV=2
sp|P49755|TMEDA_HUMAN	HUMAN	Transmembrane emp24 domain-containing protein 10 OS=Homo sapiens OX=9606 GN=TMED10 PE=1 SV=2

sp|P62140|PP1B_HUMAN	HUMAN	Serine/threonine-protein phosphatase PP1-beta catalytic subunit OS=Homo sapiens OX=9606 GN=PPP1CB PE=1 SV=3
sp|P15260|INGR1_HUMAN	HUMAN	Interferon gamma receptor 1 OS=Homo sapiens OX=9606 GN=IFNGR1 PE=1 SV=1
sp|P53990|IST1_HUMAN	HUMAN	IST1 homolog OS=Homo sapiens OX=9606 GN=IST1 PE=1 SV=1
sp|P40121|CAPG_HUMAN	HUMAN	Macrophage-capping protein OS=Homo sapiens OX=9606 GN=CAPG PE=1 SV=2
sp|P24666|PPAC_HUMAN	HUMAN	Low molecular weight phosphotyrosine protein phosphatase OS=Homo sapiens OX=9606 GN=ACP1 PE=1 SV=3
sp|Q9GZV4|IF5A2_HUMAN	HUMAN	Eukaryotic translation initiation factor 5A-2 OS=Homo sapiens OX=9606 GN=EIF5A2 PE=1 SV=3
sp|P35443|TSP4_HUMAN	HUMAN	Thrombospondin-4 OS=Homo sapiens OX=9606 GN=THBS4 PE=1 SV=2
sp|P13693|TCTP_HUMAN	HUMAN	Translationally-controlled tumor protein OS=Homo sapiens OX=9606 GN=TPT1 PE=1 SV=1
sp|Q15555|MARE2_HUMAN	HUMAN	Microtubule-associated protein RP/EB family member 2 OS=Homo sapiens OX=9606 GN=MAPRE2 PE=1 SV=1
sp|Q00577|PURA_HUMAN	HUMAN	Transcriptional activator protein Pur-alpha OS=Homo sapiens OX=9606 GN=PURA PE=1 SV=2
sp|P48059|LIMS1_HUMAN	HUMAN	LIM and senescent cell antigen-like-containing domain protein 1 OS=Homo sapiens OX=9606 GN=LIMS1 PE=1 SV=4
sp|Q06203|PUR1_HUMAN	HUMAN	Amidophosphoribosyltransferase OS=Homo sapiens OX=9606 GN=PPAT PE=1 SV=1
sp|Q9NY35|CLDN1_HUMAN	HUMAN	Claudin domain-containing protein 1 OS=Homo sapiens OX=9606 GN=CLDND1 PE=1 SV=1
sp|Q9NX76|CKLF6_HUMAN	HUMAN	CKLF-like MARVEL transmembrane domain-containing protein 6 OS=Homo sapiens OX=9606 GN=CMTM6 PE=1 SV=1
sp|Q02413|DSG1_HUMAN	HUMAN	Desmoglein-1 OS=Homo sapiens OX=9606 GN=DSG1 PE=1 SV=2
sp|Q13564|ULA1_HUMAN	HUMAN	NEDD8-activating enzyme E1 regulatory subunit OS=Homo sapiens OX=9606 GN=NAE1 PE=1 SV=1
sp|Q92478|CLC2B_HUMAN	HUMAN	C-type lectin domain family 2 member B OS=Homo sapiens OX=9606 GN=CLEC2B PE=1 SV=2
sp|P01008|ANT3_HUMAN	HUMAN	Antithrombin-III OS=Homo sapiens OX=9606 GN=SERPINC1 PE=1 SV=1
sp|O76094|SRP72_HUMAN	HUMAN	Signal recognition particle subunit SRP72 OS=Homo sapiens OX=9606 GN=SRP72 PE=1 SV=3
sp|P17252|KPCA_HUMAN	HUMAN	Protein kinase C alpha type OS=Homo sapiens OX=9606 GN=PRKCA PE=1 SV=4
sp|O60701|UGDH_HUMAN	HUMAN	UDP-glucose 6-dehydrogenase OS=Homo sapiens OX=9606 GN=UGDH PE=1 SV=1
sp|Q9BUD6|SPON2_HUMAN	HUMAN	Spondin-2 OS=Homo sapiens OX=9606 GN=SPON2 PE=1 SV=3
sp|P80303|NUCB2_HUMAN	HUMAN	Nucleobindin-2 OS=Homo sapiens OX=9606 GN=NUCB2 PE=1 SV=3
sp|P30825|SL7A1_HUMAN	HUMAN	High affinity cationic amino acid transporter 1 OS=Homo sapiens OX=9606 GN=SLC7A1 PE=1 SV=1
sp|Q08722|CD47_HUMAN	HUMAN	Leukocyte surface antigen CD47 OS=Homo sapiens OX=9606 GN=CD47 PE=1 SV=1
sp|P16152|CBR1_HUMAN	HUMAN	Carbonyl reductase [NADPH] 1 OS=Homo sapiens OX=9606 GN=CBR1 PE=1 SV=3
sp|Q9NVJ2|ARL8B_HUMAN	HUMAN	ADP-ribosylation factor-like protein 8B OS=Homo sapiens OX=9606 GN=ARL8B PE=1 SV=1
sp|Q8IZP2|ST134_HUMAN	HUMAN	Putative protein FAM10A4 OS=Homo sapiens OX=9606 GN=ST13P4 PE=5 SV=1

sp|P62273|RS29_HUMAN	HUMAN	40S ribosomal protein S29 OS=Homo sapiens OX=9606 GN=RPS29 PE=1 SV=2
sp|P40925|MDHC_HUMAN	HUMAN	Malate dehydrogenase, cytoplasmic OS=Homo sapiens OX=9606 GN=MDH1 PE=1 SV=4
sp|O00743|PPP6_HUMAN	HUMAN	Serine/threonine-protein phosphatase 6 catalytic subunit OS=Homo sapiens OX=9606 GN=PPP6C PE=1 SV=1
sp|P49840|GSK3A_HUMAN	HUMAN	Glycogen synthase kinase-3 alpha OS=Homo sapiens OX=9606 GN=GSK3A PE=1 SV=2
sp|Q08J23|NSUN2_HUMAN	HUMAN	RNA cytosine C(5)-methyltransferase NSUN2 OS=Homo sapiens OX=9606 GN=NSUN2 PE=1 SV=2
sp|Q8IZA0|K319L_HUMAN	HUMAN	Dyslexia-associated protein KIAA0319-like protein OS=Homo sapiens OX=9606 GN=KIAA0319L PE=1 SV=2
sp|P58335|ANTR2_HUMAN	HUMAN	Anthrax toxin receptor 2 OS=Homo sapiens OX=9606 GN=ANTXR2 PE=1 SV=5
sp|P68400|CSK21_HUMAN	HUMAN	Casein kinase II subunit alpha OS=Homo sapiens OX=9606 GN=CSNK2A1 PE=1 SV=1
sp|P29401|TKT_HUMAN	HUMAN	Transketolase OS=Homo sapiens OX=9606 GN=TKT PE=1 SV=3
sp|Q99816|TS101_HUMAN	HUMAN	Tumor susceptibility gene 101 protein OS=Homo sapiens OX=9606 GN=TSG101 PE=1 SV=2
sp|Q99497|PARK7_HUMAN	HUMAN	Protein/nucleic acid deglycase DJ-1 OS=Homo sapiens OX=9606 GN=PARK7 PE=1 SV=2
sp|Q10471|GALT2_HUMAN	HUMAN	Polypeptide N-acetylgalactosaminyltransferase 2 OS=Homo sapiens OX=9606 GN=GALNT2 PE=1 SV=1
sp|Q9NP79|VTA1_HUMAN	HUMAN	Vacuolar protein sorting-associated protein VTA1 homolog OS=Homo sapiens OX=9606 GN=VTA1 PE=1 SV=1
sp|Q9H4B7|TBB1_HUMAN	HUMAN	Tubulin beta-1 chain OS=Homo sapiens OX=9606 GN=TUBB1 PE=1 SV=1
sp|Q96C19|EFHD2_HUMAN	HUMAN	EF-hand domain-containing protein D2 OS=Homo sapiens OX=9606 GN=EFHD2 PE=1 SV=1
sp|Q96H20|SNF8_HUMAN	HUMAN	Vacuolar-sorting protein SNF8 OS=Homo sapiens OX=9606 GN=SNF8 PE=1 SV=1
sp|Q13630|FCL_HUMAN	HUMAN	GDP-L-fucose synthase OS=Homo sapiens OX=9606 GN=TSTA3 PE=1 SV=1
sp|Q8N1N4|K2C78_HUMAN	HUMAN	Keratin, type II cytoskeletal 78 OS=Homo sapiens OX=9606 GN=KRT78 PE=1 SV=2
sp|Q15645|PCH2_HUMAN	HUMAN	Pachytene checkpoint protein 2 homolog OS=Homo sapiens OX=9606 GN=TRIP13 PE=1 SV=2
sp|Q92619|HMHA1_HUMAN	HUMAN	Rho GTPase-activating protein 45 OS=Homo sapiens OX=9606 GN=ARHGAP45 PE=1 SV=2
sp|Q96G01|BICD1_HUMAN	HUMAN	Protein bicaudal D homolog 1 OS=Homo sapiens OX=9606 GN=BICD1 PE=1 SV=3
sp|Q9HAV0|GBB4_HUMAN	HUMAN	Guanine nucleotide-binding protein subunit beta-4 OS=Homo sapiens OX=9606 GN=GNB4 PE=1 SV=3
sp|P07477|TRY1_HUMAN	HUMAN	Trypsin-1 OS=Homo sapiens OX=9606 GN=PRSS1 PE=1 SV=1
sp|O60832|DKC1_HUMAN	HUMAN	H/ACA ribonucleoprotein complex subunit DKC1 OS=Homo sapiens OX=9606 GN=DKC1 PE=1 SV=3
sp|Q15043|S39AE_HUMAN	HUMAN	Zinc transporter ZIP14 OS=Homo sapiens OX=9606 GN=SLC39A14 PE=1 SV=3
sp|Q7Z794|K2C1B_HUMAN	HUMAN	Keratin, type II cytoskeletal 1b OS=Homo sapiens OX=9606 GN=KRT77 PE=2 SV=3
sp|P39748|FEN1_HUMAN	HUMAN	Flap endonuclease 1 OS=Homo sapiens OX=9606 GN=FEN1 PE=1 SV=1
sp|Q9HAV4|XPO5_HUMAN	HUMAN	Exportin-5 OS=Homo sapiens OX=9606 GN=XPO5 PE=1 SV=1
sp|Q9UHD8|SEPT9_HUMAN	HUMAN	Septin-9 OS=Homo sapiens OX=9606 GN=SEPTIN9 PE=1 SV=2
sp|Q96C86|DCPS_HUMAN	HUMAN	m7GpppX diphosphatase OS=Homo sapiens OX=9606 GN=DCPS PE=1 SV=2

sp|P52788|SPSY_HUMAN	HUMAN	Spermine synthase OS=Homo sapiens OX=9606 GN=SMS PE=1 SV=2
sp|P02647|APOA1_HUMAN	HUMAN	Apolipoprotein A-I OS=Homo sapiens OX=9606 GN=APOA1 PE=1 SV=1
sp|O60784|TOM1_HUMAN	HUMAN	Target of Myb protein 1 OS=Homo sapiens OX=9606 GN=TOM1 PE=1 SV=2
sp|P01893|HLAH_HUMAN	HUMAN	Putative HLA class I histocompatibility antigen, alpha chain H OS=Homo sapiens OX=9606 GN=HLA-H PE=5 SV=3
sp|P20339|RAB5A_HUMAN	HUMAN	Ras-related protein Rab-5A OS=Homo sapiens OX=9606 GN=RAB5A PE=1 SV=2
sp|P17844|DDX5_HUMAN	HUMAN	Probable ATP-dependent RNA helicase DDX5 OS=Homo sapiens OX=9606 GN=DDX5 PE=1 SV=1
sp|P13929|ENOB_HUMAN	HUMAN	Beta-enolase OS=Homo sapiens OX=9606 GN=ENO3 PE=1 SV=5
sp|P84243|H33_HUMAN	HUMAN	Histone H3.3 OS=Homo sapiens OX=9606 GN=H3-3A PE=1 SV=2
sp|Q9Y3L5|RAP2C_HUMAN	HUMAN	Ras-related protein Rap-2c OS=Homo sapiens OX=9606 GN=RAP2C PE=1 SV=1
sp|Q08209|PP2BA_HUMAN	HUMAN	Serine/threonine-protein phosphatase 2B catalytic subunit alpha isoform OS=Homo sapiens OX=9606 GN=PPP3CA PE=1 SV=1
sp|Q14019|COTL1_HUMAN	HUMAN	Coactosin-like protein OS=Homo sapiens OX=9606 GN=COTL1 PE=1 SV=3
sp|Q9H2D6|TARA_HUMAN	HUMAN	TRIO and F-actin-binding protein OS=Homo sapiens OX=9606 GN=TRIOBP PE=1 SV=3
sp|Q9NY33|DPP3_HUMAN	HUMAN	Dipeptidyl peptidase 3 OS=Homo sapiens OX=9606 GN=DPP3 PE=1 SV=2
sp|Q9BW27|NUP85_HUMAN	HUMAN	Nuclear pore complex protein Nup85 OS=Homo sapiens OX=9606 GN=NUP85 PE=1 SV=1
sp|Q99519|NEUR1_HUMAN	HUMAN	Sialidase-1 OS=Homo sapiens OX=9606 GN=NEU1 PE=1 SV=1
sp|Q14242|SELPL_HUMAN	HUMAN	P-selectin glycoprotein ligand 1 OS=Homo sapiens OX=9606 GN=SELPLG PE=1 SV=1
sp|P46439|GSTM5_HUMAN	HUMAN	Glutathione S-transferase Mu 5 OS=Homo sapiens OX=9606 GN=GSTM5 PE=1 SV=3
sp|P37108|SRP14_HUMAN	HUMAN	Signal recognition particle 14 kDa protein OS=Homo sapiens OX=9606 GN=SRP14 PE=1 SV=2
sp|P31785|IL2RG_HUMAN	HUMAN	Cytokine receptor common subunit gamma OS=Homo sapiens OX=9606 GN=IL2RG PE=1 SV=1
sp|P20774|MIME_HUMAN	HUMAN	Mimecan OS=Homo sapiens OX=9606 GN=OGN PE=1 SV=1
sp|P12236|ADT3_HUMAN	HUMAN	ADP/ATP translocase 3 OS=Homo sapiens OX=9606 GN=SLC25A6 PE=1 SV=4
sp|P09467|F16P1_HUMAN	HUMAN	Fructose-1,6-bisphosphatase 1 OS=Homo sapiens OX=9606 GN=FBP1 PE=1 SV=5
sp|P06734|FCER2_HUMAN	HUMAN	Low affinity immunoglobulin epsilon Fc receptor OS=Homo sapiens OX=9606 GN=FCER2 PE=1 SV=1
sp|P02774|VTDB_HUMAN	HUMAN	Vitamin D-binding protein OS=Homo sapiens OX=9606 GN=GC PE=1 SV=2
sp|P00488|F13A_HUMAN	HUMAN	Coagulation factor XIII A chain OS=Homo sapiens OX=9606 GN=F13A1 PE=1 SV=4
sp|Q9Y5P6|GMPPB_HUMAN	HUMAN	Mannose-1-phosphate guanyltransferase beta OS=Homo sapiens OX=9606 GN=GMPPB PE=1 SV=2
sp|Q9Y3B4|SF3B6_HUMAN	HUMAN	Splicing factor 3B subunit 6 OS=Homo sapiens OX=9606 GN=SF3B6 PE=1 SV=1
sp|Q9Y266|NUDC_HUMAN	HUMAN	Nuclear migration protein nudC OS=Homo sapiens OX=9606 GN=NUDC PE=1 SV=1
sp|Q9P1F3|ABRAL_HUMAN	HUMAN	Costars family protein ABRACL OS=Homo sapiens OX=9606 GN=ABRACL PE=1 SV=1
sp|Q9NY12|GAR1_HUMAN	HUMAN	H/ACA ribonucleoprotein complex subunit 1 OS=Homo sapiens OX=9606 GN=GAR1 PE=1 SV=1

sp|Q9NVP1|DDX18_HUMAN	HUMAN	ATP-dependent RNA helicase DDX18 OS=Homo sapiens OX=9606 GN=DDX18 PE=1 SV=2
sp|Q9NR31|SAR1A_HUMAN	HUMAN	GTP-binding protein SAR1a OS=Homo sapiens OX=9606 GN=SAR1A PE=1 SV=1
sp|Q9HC07|TM165_HUMAN	HUMAN	Transmembrane protein 165 OS=Homo sapiens OX=9606 GN=TMEM165 PE=1 SV=1
sp|Q9HB07|MYG1_HUMAN	HUMAN	UPF0160 protein MYG1, mitochondrial OS=Homo sapiens OX=9606 GN=C12orf10 PE=1 SV=2
sp|Q9H9E3|COG4_HUMAN	HUMAN	Conserved oligomeric Golgi complex subunit 4 OS=Homo sapiens OX=9606 GN=COG4 PE=1 SV=3
sp|Q9H4G4|GAPR1_HUMAN	HUMAN	Golgi-associated plant pathogenesis-related protein 1 OS=Homo sapiens OX=9606 GN=GLIPR2 PE=1 SV=3
sp|Q9H3K6|BOLA2_HUMAN	HUMAN	BolA-like protein 2 OS=Homo sapiens OX=9606 GN=BOLA2 PE=1 SV=1
sp|Q9GZM5|YIPF3_HUMAN	HUMAN	Protein YIPF3 OS=Homo sapiens OX=9606 GN=YIPF3 PE=1 SV=1
sp|Q9C0H2|TTYH3_HUMAN	HUMAN	Protein tweety homolog 3 OS=Homo sapiens OX=9606 GN=TTYH3 PE=1 SV=3
sp|Q9BPX5|ARP5L_HUMAN	HUMAN	Actin-related protein 2/3 complex subunit 5-like protein OS=Homo sapiens OX=9606 GN=ARPC5L PE=1 SV=1
sp|Q96KP4|CNDP2_HUMAN	HUMAN	Cytosolic non-specific dipeptidase OS=Homo sapiens OX=9606 GN=CNDP2 PE=1 SV=2
sp|Q969X1|LFG3_HUMAN	HUMAN	Protein lifeguard 3 OS=Homo sapiens OX=9606 GN=TMBIM1 PE=1 SV=2
sp|Q92820|GGH_HUMAN	HUMAN	Gamma-glutamyl hydrolase OS=Homo sapiens OX=9606 GN=GGH PE=1 SV=2
sp|Q8TCT8|SPP2A_HUMAN	HUMAN	Signal peptide peptidase-like 2A OS=Homo sapiens OX=9606 GN=SPPL2A PE=1 SV=2
sp|Q8N339|MT1M_HUMAN	HUMAN	Metallothionein-1M OS=Homo sapiens OX=9606 GN=MT1M PE=3 SV=2
sp|Q86VN1|VPS36_HUMAN	HUMAN	Vacuolar protein-sorting-associated protein 36 OS=Homo sapiens OX=9606 GN=VPS36 PE=1 SV=1
sp|Q6L8Q7|PDE12_HUMAN	HUMAN	2',5'-phosphodiesterase 12 OS=Homo sapiens OX=9606 GN=PDE12 PE=1 SV=2
sp|Q6DKI2|LEG9C_HUMAN	HUMAN	Galectin-9C OS=Homo sapiens OX=9606 GN=LGALS9C PE=1 SV=2
sp|Q13907|IDI1_HUMAN	HUMAN	Isopentenyl-diphosphate Delta-isomerase 1 OS=Homo sapiens OX=9606 GN=IDI1 PE=1 SV=2
sp|Q10589|BST2_HUMAN	HUMAN	Bone marrow stromal antigen 2 OS=Homo sapiens OX=9606 GN=BST2 PE=1 SV=1
sp|Q08334|I10R2_HUMAN	HUMAN	Interleukin-10 receptor subunit beta OS=Homo sapiens OX=9606 GN=IL10RB PE=1 SV=2
sp|Q01629|IFM2_HUMAN	HUMAN	Interferon-induced transmembrane protein 2 OS=Homo sapiens OX=9606 GN=IFITM2 PE=1 SV=2
sp|P62841|RS15_HUMAN	HUMAN	40S ribosomal protein S15 OS=Homo sapiens OX=9606 GN=RPS15 PE=1 SV=2
sp|P62266|RS23_HUMAN	HUMAN	40S ribosomal protein S23 OS=Homo sapiens OX=9606 GN=RPS23 PE=1 SV=3
sp|P58546|MTPN_HUMAN	HUMAN	Myotrophin OS=Homo sapiens OX=9606 GN=MTPN PE=1 SV=2
sp|P54578|UBP14_HUMAN	HUMAN	Ubiquitin carboxyl-terminal hydrolase 14 OS=Homo sapiens OX=9606 GN=USP14 PE=1 SV=3
sp|P49795|RGS19_HUMAN	HUMAN	Regulator of G-protein signaling 19 OS=Homo sapiens OX=9606 GN=RGS19 PE=1 SV=1
sp|P35244|RFA3_HUMAN	HUMAN	Replication protein A 14 kDa subunit OS=Homo sapiens OX=9606 GN=RPA3 PE=1 SV=1
sp|P31949|S10AB_HUMAN	HUMAN	Protein S100-A11 OS=Homo sapiens OX=9606 GN=S100A11 PE=1 SV=2
sp|P31350|RIR2_HUMAN	HUMAN	Ribonucleoside-diphosphate reductase subunit M2 OS=Homo sapiens OX=9606 GN=RRM2 PE=1 SV=1

sp|P28676|GRAN_HUMAN	HUMAN	Grancalcin OS=Homo sapiens OX=9606 GN=GCA PE=1 SV=2
sp|P28161|GSTM2_HUMAN	HUMAN	Glutathione S-transferase Mu 2 OS=Homo sapiens OX=9606 GN=GSTM2 PE=1 SV=2
sp|P24071|FCAR_HUMAN	HUMAN	Immunoglobulin alpha Fc receptor OS=Homo sapiens OX=9606 GN=FCAR PE=1 SV=1
sp|P15151|PVR_HUMAN	HUMAN	Poliovirus receptor OS=Homo sapiens OX=9606 GN=PVR PE=1 SV=2
sp|P05546|HEP2_HUMAN	HUMAN	Heparin cofactor 2 OS=Homo sapiens OX=9606 GN=SERPIND1 PE=1 SV=3
sp|P05543|THBG_HUMAN	HUMAN	Thyroxine-binding globulin OS=Homo sapiens OX=9606 GN=SERPINA7 PE=1 SV=2
sp|P05386|RLA1_HUMAN	HUMAN	60S acidic ribosomal protein P1 OS=Homo sapiens OX=9606 GN=RPLP1 PE=1 SV=1
sp|P00441|SODC_HUMAN	HUMAN	Superoxide dismutase [Cu-Zn] OS=Homo sapiens OX=9606 GN=SOD1 PE=1 SV=2
sp|O95456|PSMG1_HUMAN	HUMAN	Proteasome assembly chaperone 1 OS=Homo sapiens OX=9606 GN=PSMG1 PE=1 SV=1
sp|O43914|TYOBP_HUMAN	HUMAN	TYRO protein tyrosine kinase-binding protein OS=Homo sapiens OX=9606 GN=TYROBP PE=1 SV=1
sp|O00391|QSOX1_HUMAN	HUMAN	Sulfhydryl oxidase 1 OS=Homo sapiens OX=9606 GN=QSOX1 PE=1 SV=3
sp|P62993|GRB2_HUMAN	HUMAN	Growth factor receptor-bound protein 2 OS=Homo sapiens OX=9606 GN=GRB2 PE=1 SV=1
sp|Q12904|AIMP1_HUMAN	HUMAN	Aminoacyl tRNA synthase complex-interacting multifunctional protein 1 OS=Homo sapiens OX=9606 GN=AIMP1 PE=1 SV=2
sp|P13473|LAMP2_HUMAN	HUMAN	Lysosome-associated membrane glycoprotein 2 OS=Homo sapiens OX=9606 GN=LAMP2 PE=1 SV=2
sp|Q92979|NEP1_HUMAN	HUMAN	Ribosomal RNA small subunit methyltransferase NEP1 OS=Homo sapiens OX=9606 GN=EMG1 PE=1 SV=4
sp|P23921|RIR1_HUMAN	HUMAN	Ribonucleoside-diphosphate reductase large subunit OS=Homo sapiens OX=9606 GN=RRM1 PE=1 SV=1
sp|Q9UNX3|RL26L_HUMAN	HUMAN	60S ribosomal protein L26-like 1 OS=Homo sapiens OX=9606 GN=RPL26L1 PE=1 SV=1
sp|Q8NBZ7|UXS1_HUMAN	HUMAN	UDP-glucuronic acid decarboxylase 1 OS=Homo sapiens OX=9606 GN=UXS1 PE=1 SV=1
sp|Q3ZCW2|LEGL_HUMAN	HUMAN	Galectin-related protein OS=Homo sapiens OX=9606 GN=LGALSL PE=1 SV=2
sp|P14209|CD99_HUMAN	HUMAN	CD99 antigen OS=Homo sapiens OX=9606 GN=CD99 PE=1 SV=1
sp|Q9HA64|KT3K_HUMAN	HUMAN	Ketosamine-3-kinase OS=Homo sapiens OX=9606 GN=FN3KRP PE=1 SV=2
sp|Q9UNP9|PPIE_HUMAN	HUMAN	Peptidyl-prolyl cis-trans isomerase E OS=Homo sapiens OX=9606 GN=PPIE PE=1 SV=1
sp|P62308|RUXG_HUMAN	HUMAN	Small nuclear ribonucleoprotein G OS=Homo sapiens OX=9606 GN=SNRPG PE=1 SV=1
sp|P40616|ARL1_HUMAN	HUMAN	ADP-ribosylation factor-like protein 1 OS=Homo sapiens OX=9606 GN=ARL1 PE=1 SV=1
sp|P31153|METK2_HUMAN	HUMAN	S-adenosylmethionine synthase isoform type-2 OS=Homo sapiens OX=9606 GN=MAT2A PE=1 SV=1
sp|Q15382|RHEB_HUMAN	HUMAN	GTP-binding protein Rheb OS=Homo sapiens OX=9606 GN=RHEB PE=1 SV=1
sp|P24534|EF1B_HUMAN	HUMAN	Elongation factor 1-beta OS=Homo sapiens OX=9606 GN=EEF1B2 PE=1 SV=3
sp|P39019|RS19_HUMAN	HUMAN	40S ribosomal protein S19 OS=Homo sapiens OX=9606 GN=RPS19 PE=1 SV=2
sp|P62857|RS28_HUMAN	HUMAN	40S ribosomal protein S28 OS=Homo sapiens OX=9606 GN=RPS28 PE=1 SV=1
sp|P02656|APOC3_HUMAN	HUMAN	Apolipoprotein C-III OS=Homo sapiens OX=9606 GN=APOC3 PE=1 SV=1

sp|Q93096|TP4A1_HUMAN	HUMAN	Protein tyrosine phosphatase type IVA 1 OS=Homo sapiens OX=9606 GN=PTP4A1 PE=1 SV=2
sp|O15145|ARPC3_HUMAN	HUMAN	Actin-related protein 2/3 complex subunit 3 OS=Homo sapiens OX=9606 GN=ARPC3 PE=1 SV=3
sp|P30043|BLVRB_HUMAN	HUMAN	Flavin reductase (NADPH) OS=Homo sapiens OX=9606 GN=BLVRB PE=1 SV=3
sp|P42345|MTOR_HUMAN	HUMAN	Serine/threonine-protein kinase mTOR OS=Homo sapiens OX=9606 GN=MTOR PE=1 SV=1
sp|Q13443|ADAM9_HUMAN	HUMAN	Disintegrin and metalloproteinase domain-containing protein 9 OS=Homo sapiens OX=9606 GN=ADAM9 PE=1 SV=1
sp|P61081|UBC12_HUMAN	HUMAN	NEDD8-conjugating enzyme Ubc12 OS=Homo sapiens OX=9606 GN=UBE2M PE=1 SV=1
sp|P48506|GSH1_HUMAN	HUMAN	Glutamate--cysteine ligase catalytic subunit OS=Homo sapiens OX=9606 GN=GCLC PE=1 SV=2
sp|Q14314|FGL2_HUMAN	HUMAN	Fibroleukin OS=Homo sapiens OX=9606 GN=FGL2 PE=1 SV=1
sp|Q13283|G3BP1_HUMAN	HUMAN	Ras GTPase-activating protein-binding protein 1 OS=Homo sapiens OX=9606 GN=G3BP1 PE=1 SV=1
sp|O75131|CPNE3_HUMAN	HUMAN	Copine-3 OS=Homo sapiens OX=9606 GN=CPNE3 PE=1 SV=1
sp|P28065|PSB9_HUMAN	HUMAN	Proteasome subunit beta type-9 OS=Homo sapiens OX=9606 GN=PSMB9 PE=1 SV=2
sp|Q9P289|STK26_HUMAN	HUMAN	Serine/threonine-protein kinase 26 OS=Homo sapiens OX=9606 GN=STK26 PE=1 SV=2
sp|Q14376|GALE_HUMAN	HUMAN	UDP-glucose 4-epimerase OS=Homo sapiens OX=9606 GN=GALE PE=1 SV=2
sp|Q15819|UB2V2_HUMAN	HUMAN	Ubiquitin-conjugating enzyme E2 variant 2 OS=Homo sapiens OX=9606 GN=UBE2V2 PE=1 SV=4
sp|O15400|STX7_HUMAN	HUMAN	Syntaxin-7 OS=Homo sapiens OX=9606 GN=STX7 PE=1 SV=4
sp|P47755|CAZA2_HUMAN	HUMAN	F-actin-capping protein subunit alpha-2 OS=Homo sapiens OX=9606 GN=CAPZA2 PE=1 SV=3
sp|P84103|SRSF3_HUMAN	HUMAN	Serine/arginine-rich splicing factor 3 OS=Homo sapiens OX=9606 GN=SRSF3 PE=1 SV=1
sp|P18031|PTN1_HUMAN	HUMAN	Tyrosine-protein phosphatase non-receptor type 1 OS=Homo sapiens OX=9606 GN=PTPN1 PE=1 SV=1
sp|P67870|CSK2B_HUMAN	HUMAN	Casein kinase II subunit beta OS=Homo sapiens OX=9606 GN=CSNK2B PE=1 SV=1
sp|Q13547|HDAC1_HUMAN	HUMAN	Histone deacetylase 1 OS=Homo sapiens OX=9606 GN=HDAC1 PE=1 SV=1
sp|P60981|DEST_HUMAN	HUMAN	Destrin OS=Homo sapiens OX=9606 GN=DSTN PE=1 SV=3
sp|O15160|RPAC1_HUMAN	HUMAN	DNA-directed RNA polymerases I and III subunit RPAC1 OS=Homo sapiens OX=9606 GN=POLR1C PE=1 SV=1
sp|P60709|ACTB_HUMAN	HUMAN	Actin, cytoplasmic 1 OS=Homo sapiens OX=9606 GN=ACTB PE=1 SV=1
sp|O75718|CRTAP_HUMAN	HUMAN	Cartilage-associated protein OS=Homo sapiens OX=9606 GN=CRTAP PE=1 SV=1
sp|Q99436|PSB7_HUMAN	HUMAN	Proteasome subunit beta type-7 OS=Homo sapiens OX=9606 GN=PSMB7 PE=1 SV=1
sp|P52895|AK1C2_HUMAN	HUMAN	Aldo-keto reductase family 1 member C2 OS=Homo sapiens OX=9606 GN=AKR1C2 PE=1 SV=3
sp|O94979|SC31A_HUMAN	HUMAN	Protein transport protein Sec31A OS=Homo sapiens OX=9606 GN=SEC31A PE=1 SV=3
sp|P25054|APC_HUMAN	HUMAN	Adenomatous polyposis coli protein OS=Homo sapiens OX=9606 GN=APC PE=1 SV=2
sp|Q8IZP0|ABI1_HUMAN	HUMAN	Abl interactor 1 OS=Homo sapiens OX=9606 GN=ABI1 PE=1 SV=4
sp|P11049|CD37_HUMAN	HUMAN	Leukocyte antigen CD37 OS=Homo sapiens OX=9606 GN=CD37 PE=1 SV=2

sp|Q9BUJ2|HNRL1_HUMAN	HUMAN	Heterogeneous nuclear ribonucleoprotein U-like protein 1 OS=Homo sapiens OX=9606 GN=HNRNPUL1 PE=1 SV=2
sp|Q6XQN6|PNCB_HUMAN	HUMAN	Nicotinate phosphoribosyltransferase OS=Homo sapiens OX=9606 GN=NAPRT PE=1 SV=2
sp|P23469|PTPRE_HUMAN	HUMAN	Receptor-type tyrosine-protein phosphatase epsilon OS=Homo sapiens OX=9606 GN=PTPRE PE=1 SV=1
sp|P62829|RL23_HUMAN	HUMAN	60S ribosomal protein L23 OS=Homo sapiens OX=9606 GN=RPL23 PE=1 SV=1
sp|Q01650|LAT1_HUMAN	HUMAN	Large neutral amino acids transporter small subunit 1 OS=Homo sapiens OX=9606 GN=SLC7A5 PE=1 SV=2
sp|P37235|HPCL1_HUMAN	HUMAN	Hippocalcin-like protein 1 OS=Homo sapiens OX=9606 GN=HPCAL1 PE=1 SV=3
sp|Q9BQ67|GRWD1_HUMAN	HUMAN	Glutamate-rich WD repeat-containing protein 1 OS=Homo sapiens OX=9606 GN=GRWD1 PE=1 SV=1
sp|Q99828|CIB1_HUMAN	HUMAN	Calcium and integrin-binding protein 1 OS=Homo sapiens OX=9606 GN=CIB1 PE=1 SV=4
sp|P54852|EMP3_HUMAN	HUMAN	Epithelial membrane protein 3 OS=Homo sapiens OX=9606 GN=EMP3 PE=1 SV=1
sp|Q93077|H2A1C_HUMAN	HUMAN	Histone H2A type 1-C OS=Homo sapiens OX=9606 GN=HIST1H2AC PE=1 SV=3
sp|P02751|FINC_HUMAN	HUMAN	Fibronectin OS=Homo sapiens OX=9606 GN=FN1 PE=1 SV=5
sp|Q8N3D4|EH1L1_HUMAN	HUMAN	EH domain-binding protein 1-like protein 1 OS=Homo sapiens OX=9606 GN=EHBP1L1 PE=1 SV=2
sp|P62750|RL23A_HUMAN	HUMAN	60S ribosomal protein L23a OS=Homo sapiens OX=9606 GN=RPL23A PE=1 SV=1
sp|P02675|FIBB_HUMAN	HUMAN	Fibrinogen beta chain OS=Homo sapiens OX=9606 GN=FGB PE=1 SV=2
sp|O75347|TBCA_HUMAN	HUMAN	Tubulin-specific chaperone A OS=Homo sapiens OX=9606 GN=TBCA PE=1 SV=3
sp|Q92900|RENT1_HUMAN	HUMAN	Regulator of nonsense transcripts 1 OS=Homo sapiens OX=9606 GN=UPF1 PE=1 SV=2
sp|P27695|APEX1_HUMAN	HUMAN	DNA-(apurinic or apyrimidinic site) lyase OS=Homo sapiens OX=9606 GN=APEX1 PE=1 SV=2
sp|P81605|DCD_HUMAN	HUMAN	Dermcidin OS=Homo sapiens OX=9606 GN=DCD PE=1 SV=2
sp|P26447|S10A4_HUMAN	HUMAN	Protein S100-A4 OS=Homo sapiens OX=9606 GN=S100A4 PE=1 SV=1
sp|P04080|CYTB_HUMAN	HUMAN	Cystatin-B OS=Homo sapiens OX=9606 GN=CSTB PE=1 SV=2
sp|Q9BXJ4|C1QT3_HUMAN	HUMAN	Complement C1q tumor necrosis factor-related protein 3 OS=Homo sapiens OX=9606 GN=C1QTNF3 PE=1 SV=1
sp|P02765|FETUA_HUMAN	HUMAN	Alpha-2-HS-glycoprotein OS=Homo sapiens OX=9606 GN=AHSG PE=1 SV=2
sp|P09497|CLCB_HUMAN	HUMAN	Clathrin light chain B OS=Homo sapiens OX=9606 GN=CLTB PE=1 SV=1
sp|Q9H832|UBE2Z_HUMAN	HUMAN	Ubiquitin-conjugating enzyme E2 Z OS=Homo sapiens OX=9606 GN=UBE2Z PE=1 SV=2
sp|Q96EC8|YIPF6_HUMAN	HUMAN	Protein YIPF6 OS=Homo sapiens OX=9606 GN=YIPF6 PE=1 SV=2
sp|Q99571|P2RX4_HUMAN	HUMAN	P2X purinoceptor 4 OS=Homo sapiens OX=9606 GN=P2RX4 PE=1 SV=2
sp|Q13033|STRN3_HUMAN	HUMAN	Striatin-3 OS=Homo sapiens OX=9606 GN=STRN3 PE=1 SV=3
sp|P62837|UB2D2_HUMAN	HUMAN	Ubiquitin-conjugating enzyme E2 D2 OS=Homo sapiens OX=9606 GN=UBE2D2 PE=1 SV=1
sp|O43660|PLRG1_HUMAN	HUMAN	Pleiotropic regulator 1 OS=Homo sapiens OX=9606 GN=PLRG1 PE=1 SV=1
sp|P31946|1433B_HUMAN	HUMAN	14-3-3 protein beta/alpha OS=Homo sapiens OX=9606 GN=YWHAB PE=1 SV=3

sp|Q9Y6W3|CAN7_HUMAN	HUMAN	Calpain-7 OS=Homo sapiens OX=9606 GN=CAPN7 PE=1 SV=1
sp|Q9Y4Z0|LSM4_HUMAN	HUMAN	U6 snRNA-associated Sm-like protein LSm4 OS=Homo sapiens OX=9606 GN=LSM4 PE=1 SV=1
sp|O43252|PAPS1_HUMAN	HUMAN	Bifunctional 3'-phosphoadenosine 5'-phosphosulfate synthase 1 OS=Homo sapiens OX=9606 GN=PAPSS1 PE=1 SV=2
sp|Q14141|SEPT6_HUMAN	HUMAN	Septin-6 OS=Homo sapiens OX=9606 GN=SEPTIN6 PE=1 SV=4
sp|P10644|KAP0_HUMAN	HUMAN	cAMP-dependent protein kinase type I-alpha regulatory subunit OS=Homo sapiens OX=9606 GN=PRKAR1A PE=1 SV=1
sp|Q92621|NU205_HUMAN	HUMAN	Nuclear pore complex protein Nup205 OS=Homo sapiens OX=9606 GN=NUP205 PE=1 SV=3
sp|Q9NR19|ACSA_HUMAN	HUMAN	Acetyl-coenzyme A synthetase, cytoplasmic OS=Homo sapiens OX=9606 GN=ACSS2 PE=1 SV=1

B) Proteomics Analysis of U937 CDNs

Accession #	Species	Name
sp|P49327|FAS_HUMAN	HUMAN	Fatty acid synthase OS=Homo sapiens OX=9606 GN=FASN PE=1 SV=3
sp|P78527|PRKDC_HUMAN	HUMAN	DNA-dependent protein kinase catalytic subunit OS=Homo sapiens OX=9606 GN=PRKDC PE=1 SV=3
sp|P08238|HS90B_HUMAN	HUMAN	Heat shock protein HSP 90-beta OS=Homo sapiens OX=9606 GN=HSP90AB1 PE=1 SV=4
sp|P21333|FLNA_HUMAN	HUMAN	Filamin-A OS=Homo sapiens OX=9606 GN=FLNA PE=1 SV=4
sp|Q14204|DYHC1_HUMAN	HUMAN	Cytoplasmic dynein 1 heavy chain 1 OS=Homo sapiens OX=9606 GN=DYNC1H1 PE=1 SV=5
sp|Q9Y490|TLN1_HUMAN	HUMAN	Talin-1 OS=Homo sapiens OX=9606 GN=TLN1 PE=1 SV=3
sp|P35579|MYH9_HUMAN	HUMAN	Myosin-9 OS=Homo sapiens OX=9606 GN=MYH9 PE=1 SV=4
sp|P07814|SYEP_HUMAN	HUMAN	Bifunctional glutamate/proline--tRNA ligase OS=Homo sapiens OX=9606 GN=EPRS1 PE=1 SV=5
sp|P06733|ENOA_HUMAN	HUMAN	Alpha-enolase OS=Homo sapiens OX=9606 GN=ENO1 PE=1 SV=2
sp|P13639|EF2_HUMAN	HUMAN	Elongation factor 2 OS=Homo sapiens OX=9606 GN=EEF2 PE=1 SV=4
sp|P14618|KPYM_HUMAN	HUMAN	Pyruvate kinase PKM OS=Homo sapiens OX=9606 GN=PKM PE=1 SV=4
sp|P08133|ANXA6_HUMAN	HUMAN	Annexin A6 OS=Homo sapiens OX=9606 GN=ANXA6 PE=1 SV=3
sp|Q00610|CLH1_HUMAN	HUMAN	Clathrin heavy chain 1 OS=Homo sapiens OX=9606 GN=CLTC PE=1 SV=5
sp|P26038|MOES_HUMAN	HUMAN	Moesin OS=Homo sapiens OX=9606 GN=MSN PE=1 SV=3
sp|P00558|PGK1_HUMAN	HUMAN	Phosphoglycerate kinase 1 OS=Homo sapiens OX=9606 GN=PGK1 PE=1 SV=3
sp|P53396|ACLY_HUMAN	HUMAN	ATP-citrate synthase OS=Homo sapiens OX=9606 GN=ACLY PE=1 SV=3
sp|P55072|TERA_HUMAN	HUMAN	Transitional endoplasmic reticulum ATPase OS=Homo sapiens OX=9606 GN=VCP PE=1 SV=4
sp|P11142|HSP7C_HUMAN	HUMAN	Heat shock cognate 71 kDa protein OS=Homo sapiens OX=9606 GN=HSPA8 PE=1 SV=1
sp|O43707|ACTN4_HUMAN	HUMAN	Alpha-actinin-4 OS=Homo sapiens OX=9606 GN=ACTN4 PE=1 SV=2
sp|P46940|IQGA1_HUMAN	HUMAN	Ras GTPase-activating-like protein IQGAP1 OS=Homo sapiens OX=9606 GN=IQGAP1 PE=1 SV=1
sp|P22314|UBA1_HUMAN	HUMAN	Ubiquitin-like modifier-activating enzyme 1 OS=Homo sapiens OX=9606 GN=UBA1 PE=1 SV=3
sp|P07237|PDIA1_HUMAN	HUMAN	Protein disulfide-isomerase OS=Homo sapiens OX=9606 GN=P4HB PE=1 SV=3
sp|P13796|PLSL_HUMAN	HUMAN	Plastin-2 OS=Homo sapiens OX=9606 GN=LCP1 PE=1 SV=6
sp|P04075|ALDOA_HUMAN	HUMAN	Fructose-bisphosphate aldolase A OS=Homo sapiens OX=9606 GN=ALDOA PE=1 SV=2
sp|P49588|SYAC_HUMAN	HUMAN	Alanine--tRNA ligase, cytoplasmic OS=Homo sapiens OX=9606 GN=AARS1 PE=1 SV=2
sp|P78371|TCPB_HUMAN	HUMAN	T-complex protein 1 subunit beta OS=Homo sapiens OX=9606 GN=CCT2 PE=1 SV=4
sp|P04406|G3P_HUMAN	HUMAN	Glyceraldehyde-3-phosphate dehydrogenase OS=Homo sapiens OX=9606 GN=GAPDH PE=1 SV=3
sp|P48643|TCPE_HUMAN	HUMAN	T-complex protein 1 subunit epsilon OS=Homo sapiens OX=9606 GN=CCT5 PE=1 SV=1

sp|P31939|PUR9_HUMAN	HUMAN	Bifunctional purine biosynthesis protein PURH OS=Homo sapiens OX=9606 GN=ATIC PE=1 SV=3
sp|P07437|TBB5_HUMAN	HUMAN	Tubulin beta chain OS=Homo sapiens OX=9606 GN=TUBB PE=1 SV=2
sp|P26640|SYVC_HUMAN	HUMAN	Valine--tRNA ligase OS=Homo sapiens OX=9606 GN=VARS1 PE=1 SV=4
sp|P10809|CH60_HUMAN	HUMAN	60 kDa heat shock protein, mitochondrial OS=Homo sapiens OX=9606 GN=HSPD1 PE=1 SV=2
sp|P50395|GDIB_HUMAN	HUMAN	Rab GDP dissociation inhibitor beta OS=Homo sapiens OX=9606 GN=GDI2 PE=1 SV=2
sp|P50990|TCPQ_HUMAN	HUMAN	T-complex protein 1 subunit theta OS=Homo sapiens OX=9606 GN=CCT8 PE=1 SV=4
sp|P63261|ACTG_HUMAN	HUMAN	Actin, cytoplasmic 2 OS=Homo sapiens OX=9606 GN=ACTG1 PE=1 SV=1
sp|P49368|TCPG_HUMAN	HUMAN	T-complex protein 1 subunit gamma OS=Homo sapiens OX=9606 GN=CCT3 PE=1 SV=4
sp|Q99832|TCPH_HUMAN	HUMAN	T-complex protein 1 subunit eta OS=Homo sapiens OX=9606 GN=CCT7 PE=1 SV=2
sp|P54577|SYYC_HUMAN	HUMAN	Tyrosine--tRNA ligase, cytoplasmic OS=Homo sapiens OX=9606 GN=YARS1 PE=1 SV=4
sp|Q86VP6|CAND1_HUMAN	HUMAN	Cullin-associated NEDD8-dissociated protein 1 OS=Homo sapiens OX=9606 GN=CAND1 PE=1 SV=2
sp|Q01518|CAP1_HUMAN	HUMAN	Adenylyl cyclase-associated protein 1 OS=Homo sapiens OX=9606 GN=CAP1 PE=1 SV=5
sp|P07900|HS90A_HUMAN	HUMAN	Heat shock protein HSP 90-alpha OS=Homo sapiens OX=9606 GN=HSP90AA1 PE=1 SV=5
sp|P06737|PYGL_HUMAN	HUMAN	Glycogen phosphorylase, liver form OS=Homo sapiens OX=9606 GN=PYGL PE=1 SV=4
sp|P29144|TPP2_HUMAN	HUMAN	Tripeptidyl-peptidase 2 OS=Homo sapiens OX=9606 GN=TPP2 PE=1 SV=4
sp|Q13451|FKBP5_HUMAN	HUMAN	Peptidyl-prolyl cis-trans isomerase FKBP5 OS=Homo sapiens OX=9606 GN=FKBP5 PE=1 SV=2
sp|P11021|BIP_HUMAN	HUMAN	Endoplasmic reticulum chaperone BiP OS=Homo sapiens OX=9606 GN=HSPA5 PE=1 SV=2
sp|P19338|NUCL_HUMAN	HUMAN	Nucleolin OS=Homo sapiens OX=9606 GN=NCL PE=1 SV=3
sp|P68104|EF1A1_HUMAN	HUMAN	Elongation factor 1-alpha 1 OS=Homo sapiens OX=9606 GN=EEF1A1 PE=1 SV=1
sp|O00410|IPO5_HUMAN	HUMAN	Importin-5 OS=Homo sapiens OX=9606 GN=IPO5 PE=1 SV=4
sp|P11586|C1TC_HUMAN	HUMAN	C-1-tetrahydrofolate synthase, cytoplasmic OS=Homo sapiens OX=9606 GN=MTHFD1 PE=1 SV=3
sp|P31948|STIP1_HUMAN	HUMAN	Stress-induced-phosphoprotein 1 OS=Homo sapiens OX=9606 GN=STIP1 PE=1 SV=1
sp|P68363|TBA1B_HUMAN	HUMAN	Tubulin alpha-1B chain OS=Homo sapiens OX=9606 GN=TUBA1B PE=1 SV=1
sp|P13010|XRCC5_HUMAN	HUMAN	X-ray repair cross-complementing protein 5 OS=Homo sapiens OX=9606 GN=XRCC5 PE=1 SV=3
sp|P25205|MCM3_HUMAN	HUMAN	DNA replication licensing factor MCM3 OS=Homo sapiens OX=9606 GN=MCM3 PE=1 SV=3
sp|P06744|G6PI_HUMAN	HUMAN	Glucose-6-phosphate isomerase OS=Homo sapiens OX=9606 GN=GPI PE=1 SV=4
sp|P22102|PUR2_HUMAN	HUMAN	Trifunctional purine biosynthetic protein adenosine-3 OS=Homo sapiens OX=9606 GN=GART PE=1 SV=1
sp|P55786|PSA_HUMAN	HUMAN	Puromycin-sensitive aminopeptidase OS=Homo sapiens OX=9606 GN=NPEPPS PE=1 SV=2
sp|P00338|LDHA_HUMAN	HUMAN	L-lactate dehydrogenase A chain OS=Homo sapiens OX=9606 GN=LDHA PE=1 SV=2
sp|P27708|PYR1_HUMAN	HUMAN	CAD protein OS=Homo sapiens OX=9606 GN=CAD PE=1 SV=3
sp|P29401|TKT_HUMAN	HUMAN	Transketolase OS=Homo sapiens OX=9606 GN=TKT PE=1 SV=3

sp|Q92598|HS105_HUMAN	HUMAN	Heat shock protein 105 kDa OS=Homo sapiens OX=9606 GN=HSPH1 PE=1 SV=1
sp|Q9P2J5|SYLC_HUMAN	HUMAN	Leucine--tRNA ligase, cytoplasmic OS=Homo sapiens OX=9606 GN=LARS1 PE=1 SV=2
sp|P07355|ANXA2_HUMAN	HUMAN	Annexin A2 OS=Homo sapiens OX=9606 GN=ANXA2 PE=1 SV=2
sp|P63104|1433Z_HUMAN	HUMAN	14-3-3 protein zeta/delta OS=Homo sapiens OX=9606 GN=YWHAZ PE=1 SV=1
sp|P33991|MCM4_HUMAN	HUMAN	DNA replication licensing factor MCM4 OS=Homo sapiens OX=9606 GN=MCM4 PE=1 SV=5
sp|P49736|MCM2_HUMAN	HUMAN	DNA replication licensing factor MCM2 OS=Homo sapiens OX=9606 GN=MCM2 PE=1 SV=4
sp|O15067|PUR4_HUMAN	HUMAN	Phosphoribosylformylglycinamidine synthase OS=Homo sapiens OX=9606 GN=PFAS PE=1 SV=4
sp|O75369|FLNB_HUMAN	HUMAN	Filamin-B OS=Homo sapiens OX=9606 GN=FLNB PE=1 SV=2
sp|Q14152|EIF3A_HUMAN	HUMAN	Eukaryotic translation initiation factor 3 subunit A OS=Homo sapiens OX=9606 GN=EIF3A PE=1 SV=1
sp|P31146|COR1A_HUMAN	HUMAN	Coronin-1A OS=Homo sapiens OX=9606 GN=CORO1A PE=1 SV=4
sp|Q7Z6Z7|HUWE1_HUMAN	HUMAN	E3 ubiquitin-protein ligase HUWE1 OS=Homo sapiens OX=9606 GN=HUWE1 PE=1 SV=3
sp|P52209|6PGD_HUMAN	HUMAN	6-phosphogluconate dehydrogenase, decarboxylating OS=Homo sapiens OX=9606 GN=PGD PE=1 SV=3
sp|P18669|PGAM1_HUMAN	HUMAN	Phosphoglycerate mutase 1 OS=Homo sapiens OX=9606 GN=PGAM1 PE=1 SV=2
sp|P26639|SYTC_HUMAN	HUMAN	Threonine--tRNA ligase 1, cytoplasmic OS=Homo sapiens OX=9606 GN=TARS1 PE=1 SV=3
sp|P12956|XRCC6_HUMAN	HUMAN	X-ray repair cross-complementing protein 6 OS=Homo sapiens OX=9606 GN=XRCC6 PE=1 SV=2
sp|P50991|TCPD_HUMAN	HUMAN	T-complex protein 1 subunit delta OS=Homo sapiens OX=9606 GN=CCT4 PE=1 SV=4
sp|P41252|SYIC_HUMAN	HUMAN	Isoleucine--tRNA ligase, cytoplasmic OS=Homo sapiens OX=9606 GN=IARS1 PE=1 SV=2
sp|P33993|MCM7_HUMAN	HUMAN	DNA replication licensing factor MCM7 OS=Homo sapiens OX=9606 GN=MCM7 PE=1 SV=4
sp|Q02790|FKBP4_HUMAN	HUMAN	Peptidyl-prolyl cis-trans isomerase FKBP4 OS=Homo sapiens OX=9606 GN=FKBP4 PE=1 SV=3
sp|Q16555|DPYL2_HUMAN	HUMAN	Dihydropyrimidinase-related protein 2 OS=Homo sapiens OX=9606 GN=DPYSL2 PE=1 SV=1
sp|P41250|GARS_HUMAN	HUMAN	Glycine--tRNA ligase OS=Homo sapiens OX=9606 GN=GARS1 PE=1 SV=3
sp|P04264|K2C1_HUMAN	HUMAN	Keratin, type II cytoskeletal 1 OS=Homo sapiens OX=9606 GN=KRT1 PE=1 SV=6
sp|P40227|TCPZ_HUMAN	HUMAN	T-complex protein 1 subunit zeta OS=Homo sapiens OX=9606 GN=CCT6A PE=1 SV=3
sp|P17987|TCPA_HUMAN	HUMAN	T-complex protein 1 subunit alpha OS=Homo sapiens OX=9606 GN=TCP1 PE=1 SV=1
sp|P04040|CATA_HUMAN	HUMAN	Catalase OS=Homo sapiens OX=9606 GN=CAT PE=1 SV=3
sp|P23396|RS3_HUMAN	HUMAN	40S ribosomal protein S3 OS=Homo sapiens OX=9606 GN=RPS3 PE=1 SV=2
sp|O60506|HNRPQ_HUMAN	HUMAN	Heterogeneous nuclear ribonucleoprotein Q OS=Homo sapiens OX=9606 GN=SYNCRIP PE=1 SV=2
sp|P0DMV9|HS71B_HUMAN	HUMAN	Heat shock 70 kDa protein 1B OS=Homo sapiens OX=9606 GN=HSPA1B PE=1 SV=1
sp|Q8WUM4|PDC6I_HUMAN	HUMAN	Programmed cell death 6-interacting protein OS=Homo sapiens OX=9606 GN=PDCD6IP PE=1 SV=1
sp|P30101|PDIA3_HUMAN	HUMAN	Protein disulfide-isomerase A3 OS=Homo sapiens OX=9606 GN=PDIA3 PE=1 SV=4
sp|P47897|SYQ_HUMAN	HUMAN	Glutamine--tRNA ligase OS=Homo sapiens OX=9606 GN=QARS1 PE=1 SV=1

sp|P12814|ACTN1_HUMAN	HUMAN	Alpha-actinin-1 OS=Homo sapiens OX=9606 GN=ACTN1 PE=1 SV=2
sp|P30153|2AAA_HUMAN	HUMAN	Serine/threonine-protein phosphatase 2A 65 kDa regulatory subunit A alpha isoform OS=Homo sapiens OX=9606 GN=PPP2R1A PE=1 SV=4
sp|P23381|SYWC_HUMAN	HUMAN	Tryptophan--tRNA ligase, cytoplasmic OS=Homo sapiens OX=9606 GN=WARS1 PE=1 SV=2
sp|P07195|LDHB_HUMAN	HUMAN	L-lactate dehydrogenase B chain OS=Homo sapiens OX=9606 GN=LDHB PE=1 SV=2
sp|P12268|IMDH2_HUMAN	HUMAN	Inosine-5'-monophosphate dehydrogenase 2 OS=Homo sapiens OX=9606 GN=IMPDH2 PE=1 SV=2
sp|Q9H4A4|AMPB_HUMAN	HUMAN	Aminopeptidase B OS=Homo sapiens OX=9606 GN=RNPEP PE=1 SV=2
sp|P23526|SAHH_HUMAN	HUMAN	Adenosylhomocysteinase OS=Homo sapiens OX=9606 GN=AHCY PE=1 SV=4
sp|P33992|MCM5_HUMAN	HUMAN	DNA replication licensing factor MCM5 OS=Homo sapiens OX=9606 GN=MCM5 PE=1 SV=5
sp|P60174|TPIS_HUMAN	HUMAN	Triosephosphate isomerase OS=Homo sapiens OX=9606 GN=TPI1 PE=1 SV=3
sp|Q13263|TIF1B_HUMAN	HUMAN	Transcription intermediary factor 1-beta OS=Homo sapiens OX=9606 GN=TRIM28 PE=1 SV=5
sp|P05455|LA_HUMAN	HUMAN	Lupus La protein OS=Homo sapiens OX=9606 GN=SSB PE=1 SV=2
sp|Q92616|GCN1_HUMAN	HUMAN	eIF-2-alpha kinase activator GCN1 OS=Homo sapiens OX=9606 GN=GCN1 PE=1 SV=6
sp|Q99460|PSMD1_HUMAN	HUMAN	26S proteasome non-ATPase regulatory subunit 1 OS=Homo sapiens OX=9606 GN=PSMD1 PE=1 SV=2
sp|Q9UQ80|PA2G4_HUMAN	HUMAN	Proliferation-associated protein 2G4 OS=Homo sapiens OX=9606 GN=PA2G4 PE=1 SV=3
sp|P18206|VINC_HUMAN	HUMAN	Vinculin OS=Homo sapiens OX=9606 GN=VCL PE=1 SV=4
sp|P34932|HSP74_HUMAN	HUMAN	Heat shock 70 kDa protein 4 OS=Homo sapiens OX=9606 GN=HSPA4 PE=1 SV=4
sp|O43776|SYNC_HUMAN	HUMAN	Asparagine--tRNA ligase, cytoplasmic OS=Homo sapiens OX=9606 GN=NARS1 PE=1 SV=1
sp|Q14697|GANAB_HUMAN	HUMAN	Neutral alpha-glucosidase AB OS=Homo sapiens OX=9606 GN=GANAB PE=1 SV=3
sp|Q14974|IMB1_HUMAN	HUMAN	Importin subunit beta-1 OS=Homo sapiens OX=9606 GN=KPNB1 PE=1 SV=2
sp|Q14566|MCM6_HUMAN	HUMAN	DNA replication licensing factor MCM6 OS=Homo sapiens OX=9606 GN=MCM6 PE=1 SV=1
sp|O75874|IDHC_HUMAN	HUMAN	Isocitrate dehydrogenase [NADP] cytoplasmic OS=Homo sapiens OX=9606 GN=IDH1 PE=1 SV=2
sp|Q7KZF4|SND1_HUMAN	HUMAN	Staphylococcal nuclease domain-containing protein 1 OS=Homo sapiens OX=9606 GN=SND1 PE=1 SV=1
sp|P61158|ARP3_HUMAN	HUMAN	Actin-related protein 3 OS=Homo sapiens OX=9606 GN=ACTR3 PE=1 SV=3
sp|P06753|TPM3_HUMAN	HUMAN	Tropomyosin alpha-3 chain OS=Homo sapiens OX=9606 GN=TPM3 PE=1 SV=2
sp|O43175|SERA_HUMAN	HUMAN	D-3-phosphoglycerate dehydrogenase OS=Homo sapiens OX=9606 GN=PHGDH PE=1 SV=4
sp|P36871|PGM1_HUMAN	HUMAN	Phosphoglucomutase-1 OS=Homo sapiens OX=9606 GN=PGM1 PE=1 SV=3
sp|P14625|ENPL_HUMAN	HUMAN	Endoplasmin OS=Homo sapiens OX=9606 GN=HSP90B1 PE=1 SV=1
sp|P09874|PARP1_HUMAN	HUMAN	Poly [ADP-ribose] polymerase 1 OS=Homo sapiens OX=9606 GN=PARP1 PE=1 SV=4
sp|P09960|LKHA4_HUMAN	HUMAN	Leukotriene A-4 hydrolase OS=Homo sapiens OX=9606 GN=LTA4H PE=1 SV=2
sp|P28838|AMPL_HUMAN	HUMAN	Cytosol aminopeptidase OS=Homo sapiens OX=9606 GN=LAP3 PE=1 SV=3

sp|P60842|IF4A1_HUMAN	HUMAN	Eukaryotic initiation factor 4A-I OS=Homo sapiens OX=9606 GN=EIF4A1 PE=1 SV=1
sp|P04083|ANXA1_HUMAN	HUMAN	Annexin A1 OS=Homo sapiens OX=9606 GN=ANXA1 PE=1 SV=2
sp|Q06830|PRDX1_HUMAN	HUMAN	Peroxiredoxin-1 OS=Homo sapiens OX=9606 GN=PRDX1 PE=1 SV=1
sp|Q9Y617|SERC_HUMAN	HUMAN	Phosphoserine aminotransferase OS=Homo sapiens OX=9606 GN=PSAT1 PE=1 SV=2
sp|Q01813|PFKAP_HUMAN	HUMAN	ATP-dependent 6-phosphofructokinase, platelet type OS=Homo sapiens OX=9606 GN=PFKP PE=1 SV=2
sp|P48147|PPCE_HUMAN	HUMAN	Prolyl endopeptidase OS=Homo sapiens OX=9606 GN=PREP PE=1 SV=2
sp|P09429|HMGB1_HUMAN	HUMAN	High mobility group protein B1 OS=Homo sapiens OX=9606 GN=HMGB1 PE=1 SV=3
sp|P53621|COPA_HUMAN	HUMAN	Coatomer subunit alpha OS=Homo sapiens OX=9606 GN=COPA PE=1 SV=2
sp|P05120|PAI2_HUMAN	HUMAN	Plasminogen activator inhibitor 2 OS=Homo sapiens OX=9606 GN=SERPINB2 PE=1 SV=2
sp|P22234|PUR6_HUMAN	HUMAN	Multifunctional protein ADE2 OS=Homo sapiens OX=9606 GN=PAICS PE=1 SV=3
sp|P14868|SYDC_HUMAN	HUMAN	Aspartate--tRNA ligase, cytoplasmic OS=Homo sapiens OX=9606 GN=DARS1 PE=1 SV=2
sp|P00491|PNPH_HUMAN	HUMAN	Purine nucleoside phosphorylase OS=Homo sapiens OX=9606 GN=PNP PE=1 SV=2
sp|Q08211|DHX9_HUMAN	HUMAN	ATP-dependent RNA helicase A OS=Homo sapiens OX=9606 GN=DHX9 PE=1 SV=4
sp|O00299|CLIC1_HUMAN	HUMAN	Chloride intracellular channel protein 1 OS=Homo sapiens OX=9606 GN=CLIC1 PE=1 SV=4
sp|Q6XQN6|PNCB_HUMAN	HUMAN	Nicotinate phosphoribosyltransferase OS=Homo sapiens OX=9606 GN=NAPRT PE=1 SV=2
sp|P36578|RL4_HUMAN	HUMAN	60S ribosomal protein L4 OS=Homo sapiens OX=9606 GN=RPL4 PE=1 SV=5
sp|P40926|MDHM_HUMAN	HUMAN	Malate dehydrogenase, mitochondrial OS=Homo sapiens OX=9606 GN=MDH2 PE=1 SV=3
sp|P08758|ANXA5_HUMAN	HUMAN	Annexin A5 OS=Homo sapiens OX=9606 GN=ANXA5 PE=1 SV=2
sp|P62258|1433E_HUMAN	HUMAN	14-3-3 protein epsilon OS=Homo sapiens OX=9606 GN=YWHAE PE=1 SV=1
sp|P54136|SYRC_HUMAN	HUMAN	Arginine--tRNA ligase, cytoplasmic OS=Homo sapiens OX=9606 GN=RARS1 PE=1 SV=2
sp|P46777|RL5_HUMAN	HUMAN	60S ribosomal protein L5 OS=Homo sapiens OX=9606 GN=RPL5 PE=1 SV=3
sp|O14980|XPO1_HUMAN	HUMAN	Exportin-1 OS=Homo sapiens OX=9606 GN=XPO1 PE=1 SV=1
sp|Q09666|AHNK_HUMAN	HUMAN	Neuroblast differentiation-associated protein AHNAK OS=Homo sapiens OX=9606 GN=AHNAK PE=1 SV=2
sp|P13489|RINI_HUMAN	HUMAN	Ribonuclease inhibitor OS=Homo sapiens OX=9606 GN=RNH1 PE=1 SV=2
sp|P62937|PPIA_HUMAN	HUMAN	Peptidyl-prolyl cis-trans isomerase A OS=Homo sapiens OX=9606 GN=PPIA PE=1 SV=2
sp|P49915|GUAA_HUMAN	HUMAN	GMP synthase [glutamine-hydrolyzing] OS=Homo sapiens OX=9606 GN=GMPS PE=1 SV=1
sp|P23528|COF1_HUMAN	HUMAN	Cofilin-1 OS=Homo sapiens OX=9606 GN=CFL1 PE=1 SV=3
sp|P07737|PROF1_HUMAN	HUMAN	Profilin-1 OS=Homo sapiens OX=9606 GN=PFN1 PE=1 SV=2
sp|P29350|PTN6_HUMAN	HUMAN	Tyrosine-protein phosphatase non-receptor type 6 OS=Homo sapiens OX=9606 GN=PTPN6 PE=1 SV=1
sp|Q15046|SYK_HUMAN	HUMAN	Lysine--tRNA ligase OS=Homo sapiens OX=9606 GN=KARS1 PE=1 SV=3
sp|Q16543|CDC37_HUMAN	HUMAN	Hsp90 co-chaperone Cdc37 OS=Homo sapiens OX=9606 GN=CDC37 PE=1 SV=1

sp|P52566|GDIR2_HUMAN	HUMAN	Rho GDP-dissociation inhibitor 2 OS=Homo sapiens OX=9606 GN=ARHGDIB PE=1 SV=3
sp|Q86UX7|URP2_HUMAN	HUMAN	Fermitin family homolog 3 OS=Homo sapiens OX=9606 GN=FERMT3 PE=1 SV=1
sp|P21399|ACOC_HUMAN	HUMAN	Cytoplasmic aconitate hydratase OS=Homo sapiens OX=9606 GN=ACO1 PE=1 SV=3
sp|P40925|MDHC_HUMAN	HUMAN	Malate dehydrogenase, cytoplasmic OS=Homo sapiens OX=9606 GN=MDH1 PE=1 SV=4
sp|Q96KP4|CNDP2_HUMAN	HUMAN	Cytosolic non-specific dipeptidase OS=Homo sapiens OX=9606 GN=CNDP2 PE=1 SV=2
sp|P39687|AN32A_HUMAN	HUMAN	Acidic leucine-rich nuclear phosphoprotein 32 family member A OS=Homo sapiens OX=9606 GN=ANP32A PE=1 SV=1
sp|Q99497|PARK7_HUMAN	HUMAN	Protein/nucleic acid deglycase DJ-1 OS=Homo sapiens OX=9606 GN=PARK7 PE=1 SV=2
sp|P38646|GRP75_HUMAN	HUMAN	Stress-70 protein, mitochondrial OS=Homo sapiens OX=9606 GN=HSPA9 PE=1 SV=2
sp|P11940|PABP1_HUMAN	HUMAN	Polyadenylate-binding protein 1 OS=Homo sapiens OX=9606 GN=PABPC1 PE=1 SV=2
sp|Q16531|DDB1_HUMAN	HUMAN	DNA damage-binding protein 1 OS=Homo sapiens OX=9606 GN=DDB1 PE=1 SV=1
sp|O75083|WDR1_HUMAN	HUMAN	WD repeat-containing protein 1 OS=Homo sapiens OX=9606 GN=WDR1 PE=1 SV=4
sp|P27348|1433T_HUMAN	HUMAN	14-3-3 protein theta OS=Homo sapiens OX=9606 GN=YWHAQ PE=1 SV=1
sp|O43143|DHX15_HUMAN	HUMAN	Pre-mRNA-splicing factor ATP-dependent RNA helicase DHX15 OS=Homo sapiens OX=9606 GN=DHX15 PE=1 SV=2
sp|P55884|EIF3B_HUMAN	HUMAN	Eukaryotic translation initiation factor 3 subunit B OS=Homo sapiens OX=9606 GN=EIF3B PE=1 SV=3
sp|P08865|RSSA_HUMAN	HUMAN	40S ribosomal protein SA OS=Homo sapiens OX=9606 GN=RPSA PE=1 SV=4
sp|Q9UBT2|SAE2_HUMAN	HUMAN	SUMO-activating enzyme subunit 2 OS=Homo sapiens OX=9606 GN=UBA2 PE=1 SV=2
sp|P62826|RAN_HUMAN	HUMAN	GTP-binding nuclear protein Ran OS=Homo sapiens OX=9606 GN=RAN PE=1 SV=3
sp|Q9Y230|RUVB2_HUMAN	HUMAN	RuvB-like 2 OS=Homo sapiens OX=9606 GN=RUVBL2 PE=1 SV=3
sp|P62195|PRS8_HUMAN	HUMAN	26S proteasome regulatory subunit 8 OS=Homo sapiens OX=9606 GN=PSMC5 PE=1 SV=1
sp|P55060|XPO2_HUMAN	HUMAN	Exportin-2 OS=Homo sapiens OX=9606 GN=CSE1L PE=1 SV=3
sp|Q15029|U5S1_HUMAN	HUMAN	116 kDa U5 small nuclear ribonucleoprotein component OS=Homo sapiens OX=9606 GN=EFTUD2 PE=1 SV=1
sp|Q15181|IPYR_HUMAN	HUMAN	Inorganic pyrophosphatase OS=Homo sapiens OX=9606 GN=PPA1 PE=1 SV=2
sp|P38606|VATA_HUMAN	HUMAN	V-type proton ATPase catalytic subunit A OS=Homo sapiens OX=9606 GN=ATP6V1A PE=1 SV=2
sp|P61160|ARP2_HUMAN	HUMAN	Actin-related protein 2 OS=Homo sapiens OX=9606 GN=ACTR2 PE=1 SV=1
sp|P16930|FAAA_HUMAN	HUMAN	Fumarylacetoacetase OS=Homo sapiens OX=9606 GN=FAH PE=1 SV=2
sp|P30041|PRDX6_HUMAN	HUMAN	Peroxiredoxin-6 OS=Homo sapiens OX=9606 GN=PRDX6 PE=1 SV=3
sp|Q08J23|NSUN2_HUMAN	HUMAN	RNA cytosine C(5)-methyltransferase NSUN2 OS=Homo sapiens OX=9606 GN=NSUN2 PE=1 SV=2
sp|O60664|PLIN3_HUMAN	HUMAN	Perilipin-3 OS=Homo sapiens OX=9606 GN=PLIN3 PE=1 SV=3
sp|Q9NQW7|XPP1_HUMAN	HUMAN	Xaa-Pro aminopeptidase 1 OS=Homo sapiens OX=9606 GN=XPNPEP1 PE=1 SV=3

sp|P25786|PSA1_HUMAN	HUMAN	Proteasome subunit alpha type-1 OS=Homo sapiens OX=9606 GN=PSMA1 PE=1 SV=1
sp|Q9NTJ3|SMC4_HUMAN	HUMAN	Structural maintenance of chromosomes protein 4 OS=Homo sapiens OX=9606 GN=SMC4 PE=1 SV=2
sp|P42224|STAT1_HUMAN	HUMAN	Signal transducer and activator of transcription 1-alpha/beta OS=Homo sapiens OX=9606 GN=STAT1 PE=1 SV=2
sp|Q92945|FUBP2_HUMAN	HUMAN	Far upstream element-binding protein 2 OS=Homo sapiens OX=9606 GN=KHSRP PE=1 SV=4
sp|P12081|HARS1_HUMAN	HUMAN	Histidine--tRNA ligase, cytoplasmic OS=Homo sapiens OX=9606 GN=HARS1 PE=1 SV=2
sp|P06576|ATPB_HUMAN	HUMAN	ATP synthase subunit beta, mitochondrial OS=Homo sapiens OX=9606 GN=ATP5F1B PE=1 SV=3
sp|Q9P258|RCC2_HUMAN	HUMAN	Protein RCC2 OS=Homo sapiens OX=9606 GN=RCC2 PE=1 SV=2
sp|Q9HB71|CYBP_HUMAN	HUMAN	Calcyclin-binding protein OS=Homo sapiens OX=9606 GN=CACYBP PE=1 SV=2
sp|P26641|EF1G_HUMAN	HUMAN	Elongation factor 1-gamma OS=Homo sapiens OX=9606 GN=EEF1G PE=1 SV=3
sp|P29692|EF1D_HUMAN	HUMAN	Elongation factor 1-delta OS=Homo sapiens OX=9606 GN=EEF1D PE=1 SV=5
sp|P49591|SYSC_HUMAN	HUMAN	Serine--tRNA ligase, cytoplasmic OS=Homo sapiens OX=9606 GN=SARS1 PE=1 SV=3
sp|P56192|SYMC_HUMAN	HUMAN	Methionine--tRNA ligase, cytoplasmic OS=Homo sapiens OX=9606 GN=MARS1 PE=1 SV=2
sp|O95347|SMC2_HUMAN	HUMAN	Structural maintenance of chromosomes protein 2 OS=Homo sapiens OX=9606 GN=SMC2 PE=1 SV=2
sp|O60841|IF2P_HUMAN	HUMAN	Eukaryotic translation initiation factor 5B OS=Homo sapiens OX=9606 GN=EIF5B PE=1 SV=4
sp|P05388|RLA0_HUMAN	HUMAN	60S acidic ribosomal protein P0 OS=Homo sapiens OX=9606 GN=RPLP0 PE=1 SV=1
sp|P61978|HNRPK_HUMAN	HUMAN	Heterogeneous nuclear ribonucleoprotein K OS=Homo sapiens OX=9606 GN=HNRNPK PE=1 SV=1
sp|P60900|PSA6_HUMAN	HUMAN	Proteasome subunit alpha type-6 OS=Homo sapiens OX=9606 GN=PSMA6 PE=1 SV=1
sp|Q99613|EIF3C_HUMAN	HUMAN	Eukaryotic translation initiation factor 3 subunit C OS=Homo sapiens OX=9606 GN=EIF3C PE=1 SV=1
sp|P27797|CALR_HUMAN	HUMAN	Calreticulin OS=Homo sapiens OX=9606 GN=CALR PE=1 SV=1
sp|P54920|SNAA_HUMAN	HUMAN	Alpha-soluble NSF attachment protein OS=Homo sapiens OX=9606 GN=NAPA PE=1 SV=3
sp|O00429|DNM1L_HUMAN	HUMAN	Dynamin-1-like protein OS=Homo sapiens OX=9606 GN=DNM1L PE=1 SV=2
sp|P23141|EST1_HUMAN	HUMAN	Liver carboxylesterase 1 OS=Homo sapiens OX=9606 GN=CES1 PE=1 SV=2
sp|P30740|ILEU_HUMAN	HUMAN	Leukocyte elastase inhibitor OS=Homo sapiens OX=9606 GN=SERPINB1 PE=1 SV=1
sp|P35527|K1C9_HUMAN	HUMAN	Keratin, type I cytoskeletal 9 OS=Homo sapiens OX=9606 GN=KRT9 PE=1 SV=3
sp|Q9Y265|RUVB1_HUMAN	HUMAN	RuvB-like 1 OS=Homo sapiens OX=9606 GN=RUVBL1 PE=1 SV=1
sp|Q9NTK5|OLA1_HUMAN	HUMAN	Obg-like ATPase 1 OS=Homo sapiens OX=9606 GN=OLA1 PE=1 SV=2
sp|Q01469|FABP5_HUMAN	HUMAN	Fatty acid-binding protein 5 OS=Homo sapiens OX=9606 GN=FABP5 PE=1 SV=3
sp|P11908|PRPS2_HUMAN	HUMAN	Ribose-phosphate pyrophosphokinase 2 OS=Homo sapiens OX=9606 GN=PRPS2 PE=1 SV=2
sp|P37802|TAGL2_HUMAN	HUMAN	Transgelin-2 OS=Homo sapiens OX=9606 GN=TAGLN2 PE=1 SV=3
sp|P49589|SYCC_HUMAN	HUMAN	Cysteine--tRNA ligase, cytoplasmic OS=Homo sapiens OX=9606 GN=CARS1 PE=1 SV=3

contam_sp|ALBU_BOVIN|		contam_sp|ALBU_BOVIN|
sp|P15311|EZRI_HUMAN	HUMAN	Ezrin OS=Homo sapiens OX=9606 GN=EZR PE=1 SV=4
sp|Q00796|DHSO_HUMAN	HUMAN	Sorbitol dehydrogenase OS=Homo sapiens OX=9606 GN=SORD PE=1 SV=4
sp|Q13303|KCAB2_HUMAN	HUMAN	Voltage-gated potassium channel subunit beta-2 OS=Homo sapiens OX=9606 GN=KCNAB2 PE=1 SV=2
sp|P45974|UBP5_HUMAN	HUMAN	Ubiquitin carboxyl-terminal hydrolase 5 OS=Homo sapiens OX=9606 GN=USP5 PE=1 SV=2
sp|Q9Y4L1|HYOU1_HUMAN	HUMAN	Hypoxia up-regulated protein 1 OS=Homo sapiens OX=9606 GN=HYOU1 PE=1 SV=1
sp|P61981|1433G_HUMAN	HUMAN	14-3-3 protein gamma OS=Homo sapiens OX=9606 GN=YWHAG PE=1 SV=2
sp|P63241|IF5A1_HUMAN	HUMAN	Eukaryotic translation initiation factor 5A-1 OS=Homo sapiens OX=9606 GN=EIF5A PE=1 SV=2
sp|Q16881|TRXR1_HUMAN	HUMAN	Thioredoxin reductase 1, cytoplasmic OS=Homo sapiens OX=9606 GN=TXNRD1 PE=1 SV=3
sp|P49321|NASP_HUMAN	HUMAN	Nuclear autoantigenic sperm protein OS=Homo sapiens OX=9606 GN=NASP PE=1 SV=2
sp|O00232|PSD12_HUMAN	HUMAN	26S proteasome non-ATPase regulatory subunit 12 OS=Homo sapiens OX=9606 GN=PSMD12 PE=1 SV=3
sp|P13667|PDIA4_HUMAN	HUMAN	Protein disulfide-isomerase A4 OS=Homo sapiens OX=9606 GN=PDIA4 PE=1 SV=2
sp|P35998|PRS7_HUMAN	HUMAN	26S proteasome regulatory subunit 7 OS=Homo sapiens OX=9606 GN=PSMC2 PE=1 SV=3
sp|P62701|RS4X_HUMAN	HUMAN	40S ribosomal protein S4, X isoform OS=Homo sapiens OX=9606 GN=RPS4X PE=1 SV=2
sp|Q9BXJ9|NAA15_HUMAN	HUMAN	N-alpha-acetyltransferase 15, NatA auxiliary subunit OS=Homo sapiens OX=9606 GN=NAA15 PE=1 SV=1
sp|P09211|GSTP1_HUMAN	HUMAN	Glutathione S-transferase P OS=Homo sapiens OX=9606 GN=GSTP1 PE=1 SV=2
sp|P49189|AL9A1_HUMAN	HUMAN	4-trimethylaminobutyraldehyde dehydrogenase OS=Homo sapiens OX=9606 GN=ALDH9A1 PE=1 SV=3
sp|Q9UNM6|PSD13_HUMAN	HUMAN	26S proteasome non-ATPase regulatory subunit 13 OS=Homo sapiens OX=9606 GN=PSMD13 PE=1 SV=2
sp|P30566|PUR8_HUMAN	HUMAN	Adenylosuccinate lyase OS=Homo sapiens OX=9606 GN=ADSL PE=1 SV=2
sp|P47756|CAPZB_HUMAN	HUMAN	F-actin-capping protein subunit beta OS=Homo sapiens OX=9606 GN=CAPZB PE=1 SV=4
sp|Q9NY33|DPP3_HUMAN	HUMAN	Dipeptidyl peptidase 3 OS=Homo sapiens OX=9606 GN=DPP3 PE=1 SV=2
sp|O00571|DDX3X_HUMAN	HUMAN	ATP-dependent RNA helicase DDX3X OS=Homo sapiens OX=9606 GN=DDX3X PE=1 SV=3
sp|P62140|PP1B_HUMAN	HUMAN	Serine/threonine-protein phosphatase PP1-beta catalytic subunit OS=Homo sapiens OX=9606 GN=PPP1CB PE=1 SV=3
sp|P00367|DHE3_HUMAN	HUMAN	Glutamate dehydrogenase 1, mitochondrial OS=Homo sapiens OX=9606 GN=GLUD1 PE=1 SV=2
sp|P11413|G6PD_HUMAN	HUMAN	Glucose-6-phosphate 1-dehydrogenase OS=Homo sapiens OX=9606 GN=G6PD PE=1 SV=4
sp|P06132|DCUP_HUMAN	HUMAN	Uroporphyrinogen decarboxylase OS=Homo sapiens OX=9606 GN=UROD PE=1 SV=2
sp|Q9Y262|EIF3L_HUMAN	HUMAN	Eukaryotic translation initiation factor 3 subunit L OS=Homo sapiens OX=9606 GN=EIF3L PE=1 SV=1
sp|P41091|IF2G_HUMAN	HUMAN	Eukaryotic translation initiation factor 2 subunit 3 OS=Homo sapiens OX=9606 GN=EIF2S3 PE=1 SV=3
sp|P52907|CAZA1_HUMAN	HUMAN	F-actin-capping protein subunit alpha-1 OS=Homo sapiens OX=9606 GN=CAPZA1 PE=1 SV=3
sp|Q13045|FLII_HUMAN	HUMAN	Protein flightless-1 homolog OS=Homo sapiens OX=9606 GN=FLII PE=1 SV=2

sp|O14745|NHRF1_HUMAN	HUMAN	Na(+)/H(+) exchange regulatory cofactor NHE-RF1 OS=Homo sapiens OX=9606 GN=SLC9A3R1 PE=1 SV=4
sp|O75643|U520_HUMAN	HUMAN	U5 small nuclear ribonucleoprotein 200 kDa helicase OS=Homo sapiens OX=9606 GN=SNRNP200 PE=1 SV=2
sp|P22392|NDKB_HUMAN	HUMAN	Nucleoside diphosphate kinase B OS=Homo sapiens OX=9606 GN=NME2 PE=1 SV=1
sp|P14550|AK1A1_HUMAN	HUMAN	Aldo-keto reductase family 1 member A1 OS=Homo sapiens OX=9606 GN=AKR1A1 PE=1 SV=3
sp|Q14166|TTL12_HUMAN	HUMAN	Tubulin--tyrosine ligase-like protein 12 OS=Homo sapiens OX=9606 GN=TTLL12 PE=1 SV=2
sp|P37837|TALDO_HUMAN	HUMAN	Transaldolase OS=Homo sapiens OX=9606 GN=TALDO1 PE=1 SV=2
sp|Q15021|CND1_HUMAN	HUMAN	Condensin complex subunit 1 OS=Homo sapiens OX=9606 GN=NCAPD2 PE=1 SV=3
sp|P04792|HSPB1_HUMAN	HUMAN	Heat shock protein beta-1 OS=Homo sapiens OX=9606 GN=HSPB1 PE=1 SV=2
sp|P43686|PRS6B_HUMAN	HUMAN	26S proteasome regulatory subunit 6B OS=Homo sapiens OX=9606 GN=PSMC4 PE=1 SV=2
sp|P50995|ANX11_HUMAN	HUMAN	Annexin A11 OS=Homo sapiens OX=9606 GN=ANXA11 PE=1 SV=1
sp|P63244|RACK1_HUMAN	HUMAN	Receptor of activated protein C kinase 1 OS=Homo sapiens OX=9606 GN=RACK1 PE=1 SV=3
sp|Q92499|DDX1_HUMAN	HUMAN	ATP-dependent RNA helicase DDX1 OS=Homo sapiens OX=9606 GN=DDX1 PE=1 SV=2
sp|Q9UNZ2|NSF1C_HUMAN	HUMAN	NSFL1 cofactor p47 OS=Homo sapiens OX=9606 GN=NSFL1C PE=1 SV=2
sp|P21281|VATB2_HUMAN	HUMAN	V-type proton ATPase subunit B, brain isoform OS=Homo sapiens OX=9606 GN=ATP6V1B2 PE=1 SV=3
sp|O00231|PSD11_HUMAN	HUMAN	26S proteasome non-ATPase regulatory subunit 11 OS=Homo sapiens OX=9606 GN=PSMD11 PE=1 SV=3
sp|P19367|HXK1_HUMAN	HUMAN	Hexokinase-1 OS=Homo sapiens OX=9606 GN=HK1 PE=1 SV=3
sp|O14818|PSA7_HUMAN	HUMAN	Proteasome subunit alpha type-7 OS=Homo sapiens OX=9606 GN=PSMA7 PE=1 SV=1
sp|P23193|TCEA1_HUMAN	HUMAN	Transcription elongation factor A protein 1 OS=Homo sapiens OX=9606 GN=TCEA1 PE=1 SV=2
sp|O43242|PSMD3_HUMAN	HUMAN	26S proteasome non-ATPase regulatory subunit 3 OS=Homo sapiens OX=9606 GN=PSMD3 PE=1 SV=2
sp|Q9BWD1|THIC_HUMAN	HUMAN	Acetyl-CoA acetyltransferase, cytosolic OS=Homo sapiens OX=9606 GN=ACAT2 PE=1 SV=2
sp|P08567|PLEK_HUMAN	HUMAN	Pleckstrin OS=Homo sapiens OX=9606 GN=PLEK PE=1 SV=3
sp|P17858|PFKAL_HUMAN	HUMAN	ATP-dependent 6-phosphofructokinase, liver type OS=Homo sapiens OX=9606 GN=PFKL PE=1 SV=6
sp|Q04446|GLGB_HUMAN	HUMAN	1,4-alpha-glucan-branching enzyme OS=Homo sapiens OX=9606 GN=GBE1 PE=1 SV=3
sp|Q06203|PUR1_HUMAN	HUMAN	Amidophosphoribosyltransferase OS=Homo sapiens OX=9606 GN=PPAT PE=1 SV=1
sp|P49411|EFTU_HUMAN	HUMAN	Elongation factor Tu, mitochondrial OS=Homo sapiens OX=9606 GN=TUFM PE=1 SV=2
sp|Q9Y266|NUDC_HUMAN	HUMAN	Nuclear migration protein nudC OS=Homo sapiens OX=9606 GN=NUDC PE=1 SV=1
sp|Q16576|RBBP7_HUMAN	HUMAN	Histone-binding protein RBBP7 OS=Homo sapiens OX=9606 GN=RBBP7 PE=1 SV=1
sp|Q06210|GFPT1_HUMAN	HUMAN	Glutamine--fructose-6-phosphate aminotransferase [isomerizing] 1 OS=Homo sapiens OX=9606 GN=GFPT1 PE=1 SV=3
sp|Q13200|PSMD2_HUMAN	HUMAN	26S proteasome non-ATPase regulatory subunit 2 OS=Homo sapiens OX=9606 GN=PSMD2 PE=1 SV=3
sp|Q04760|LGUL_HUMAN	HUMAN	Lactoylglutathione lyase OS=Homo sapiens OX=9606 GN=GLO1 PE=1 SV=4

sp|P02545|LMNA_HUMAN	HUMAN	Prelamin-A/C OS=Homo sapiens OX=9606 GN=LMNA PE=1 SV=1
sp|Q9NSD9|SYFB_HUMAN	HUMAN	Phenylalanine--tRNA ligase beta subunit OS=Homo sapiens OX=9606 GN=FARSB PE=1 SV=3
sp|O15143|ARC1B_HUMAN	HUMAN	Actin-related protein 2/3 complex subunit 1B OS=Homo sapiens OX=9606 GN=ARPC1B PE=1 SV=3
sp|Q12904|AIMP1_HUMAN	HUMAN	Aminoacyl tRNA synthase complex-interacting multifunctional protein 1 OS=Homo sapiens OX=9606 GN=AIMP1 PE=1 SV=2
sp|P13798|ACPH_HUMAN	HUMAN	Acylamino-acid-releasing enzyme OS=Homo sapiens OX=9606 GN=APEH PE=1 SV=4
sp|Q14683|SMC1A_HUMAN	HUMAN	Structural maintenance of chromosomes protein 1A OS=Homo sapiens OX=9606 GN=SMC1A PE=1 SV=2
sp|P30086|PEBP1_HUMAN	HUMAN	Phosphatidylethanolamine-binding protein 1 OS=Homo sapiens OX=9606 GN=PEBP1 PE=1 SV=3
sp|P62081|RS7_HUMAN	HUMAN	40S ribosomal protein S7 OS=Homo sapiens OX=9606 GN=RPS7 PE=1 SV=1
sp|P42704|LPPRC_HUMAN	HUMAN	Leucine-rich PPR motif-containing protein, mitochondrial OS=Homo sapiens OX=9606 GN=LRPPRC PE=1 SV=3
sp|P20618|PSB1_HUMAN	HUMAN	Proteasome subunit beta type-1 OS=Homo sapiens OX=9606 GN=PSMB1 PE=1 SV=2
sp|Q9Y295|DRG1_HUMAN	HUMAN	Developmentally-regulated GTP-binding protein 1 OS=Homo sapiens OX=9606 GN=DRG1 PE=1 SV=1
sp|P51991|ROA3_HUMAN	HUMAN	Heterogeneous nuclear ribonucleoprotein A3 OS=Homo sapiens OX=9606 GN=HNRNPA3 PE=1 SV=2
sp|P33316|DUT_HUMAN	HUMAN	Deoxyuridine 5'-triphosphate nucleotidohydrolase, mitochondrial OS=Homo sapiens OX=9606 GN=DUT PE=1 SV=4
sp|P39023|RL3_HUMAN	HUMAN	60S ribosomal protein L3 OS=Homo sapiens OX=9606 GN=RPL3 PE=1 SV=2
sp|P62249|RS16_HUMAN	HUMAN	40S ribosomal protein S16 OS=Homo sapiens OX=9606 GN=RPS16 PE=1 SV=2
sp|P14317|HCLS1_HUMAN	HUMAN	Hematopoietic lineage cell-specific protein OS=Homo sapiens OX=9606 GN=HCLS1 PE=1 SV=3
sp|Q13838|DX39B_HUMAN	HUMAN	Spliceosome RNA helicase DDX39B OS=Homo sapiens OX=9606 GN=DDX39B PE=1 SV=1
sp|P62333|PRS10_HUMAN	HUMAN	26S proteasome regulatory subunit 10B OS=Homo sapiens OX=9606 GN=PSMC6 PE=1 SV=1
sp|P55209|NP1L1_HUMAN	HUMAN	Nucleosome assembly protein 1-like 1 OS=Homo sapiens OX=9606 GN=NAP1L1 PE=1 SV=1
sp|O75533|SF3B1_HUMAN	HUMAN	Splicing factor 3B subunit 1 OS=Homo sapiens OX=9606 GN=SF3B1 PE=1 SV=3
sp|P17980|PRS6A_HUMAN	HUMAN	26S proteasome regulatory subunit 6A OS=Homo sapiens OX=9606 GN=PSMC3 PE=1 SV=3
sp|P15880|RS2_HUMAN	HUMAN	40S ribosomal protein S2 OS=Homo sapiens OX=9606 GN=RPS2 PE=1 SV=2
sp|P00390|GSHR_HUMAN	HUMAN	Glutathione reductase, mitochondrial OS=Homo sapiens OX=9606 GN=GSR PE=1 SV=2
sp|Q12906|ILF3_HUMAN	HUMAN	Interleukin enhancer-binding factor 3 OS=Homo sapiens OX=9606 GN=ILF3 PE=1 SV=3
sp|P12955|PEPD_HUMAN	HUMAN	Xaa-Pro dipeptidase OS=Homo sapiens OX=9606 GN=PEPD PE=1 SV=3
sp|P43487|RANG_HUMAN	HUMAN	Ran-specific GTPase-activating protein OS=Homo sapiens OX=9606 GN=RANBP1 PE=1 SV=1
sp|P27816|MAP4_HUMAN	HUMAN	Microtubule-associated protein 4 OS=Homo sapiens OX=9606 GN=MAP4 PE=1 SV=3
sp|O15355|PPM1G_HUMAN	HUMAN	Protein phosphatase 1G OS=Homo sapiens OX=9606 GN=PPM1G PE=1 SV=1
sp|Q96AE4|FUBP1_HUMAN	HUMAN	Far upstream element-binding protein 1 OS=Homo sapiens OX=9606 GN=FUBP1 PE=1 SV=3

sp|Q01105|SET_HUMAN	HUMAN	Protein SET OS=Homo sapiens OX=9606 GN=SET PE=1 SV=3
sp|Q16539|MK14_HUMAN	HUMAN	Mitogen-activated protein kinase 14 OS=Homo sapiens OX=9606 GN=MAPK14 PE=1 SV=3
sp|Q8IZ83|A16A1_HUMAN	HUMAN	Aldehyde dehydrogenase family 16 member A1 OS=Homo sapiens OX=9606 GN=ALDH16A1 PE=1 SV=2
sp|Q00839|HNRPU_HUMAN	HUMAN	Heterogeneous nuclear ribonucleoprotein U OS=Homo sapiens OX=9606 GN=HNRNPU PE=1 SV=6
sp|P33176|KINH_HUMAN	HUMAN	Kinesin-1 heavy chain OS=Homo sapiens OX=9606 GN=KIF5B PE=1 SV=1
sp|P17174|AATC_HUMAN	HUMAN	Aspartate aminotransferase, cytoplasmic OS=Homo sapiens OX=9606 GN=GOT1 PE=1 SV=3
sp|P43490|NAMPT_HUMAN	HUMAN	Nicotinamide phosphoribosyltransferase OS=Homo sapiens OX=9606 GN=NAMPT PE=1 SV=1
sp|P61586|RHOA_HUMAN	HUMAN	Transforming protein RhoA OS=Homo sapiens OX=9606 GN=RHOA PE=1 SV=1
sp|Q06323|PSME1_HUMAN	HUMAN	Proteasome activator complex subunit 1 OS=Homo sapiens OX=9606 GN=PSME1 PE=1 SV=1
sp|Q6IBS0|TWF2_HUMAN	HUMAN	Twinfilin-2 OS=Homo sapiens OX=9606 GN=TWF2 PE=1 SV=2
sp|Q02878|RL6_HUMAN	HUMAN	60S ribosomal protein L6 OS=Homo sapiens OX=9606 GN=RPL6 PE=1 SV=3
sp|P61247|RS3A_HUMAN	HUMAN	40S ribosomal protein S3a OS=Homo sapiens OX=9606 GN=RPS3A PE=1 SV=2
sp|P50502|F10A1_HUMAN	HUMAN	Hsc70-interacting protein OS=Homo sapiens OX=9606 GN=ST13 PE=1 SV=2
sp|Q13126|MTAP_HUMAN	HUMAN	S-methyl-5'-thioadenosine phosphorylase OS=Homo sapiens OX=9606 GN=MTAP PE=1 SV=2
sp|Q13561|DCTN2_HUMAN	HUMAN	Dynactin subunit 2 OS=Homo sapiens OX=9606 GN=DCTN2 PE=1 SV=4
sp|Q96G03|PGM2_HUMAN	HUMAN	Phosphoglucomutase-2 OS=Homo sapiens OX=9606 GN=PGM2 PE=1 SV=4
sp|P05198|IF2A_HUMAN	HUMAN	Eukaryotic translation initiation factor 2 subunit 1 OS=Homo sapiens OX=9606 GN=EIF2S1 PE=1 SV=3
sp|P34897|GLYM_HUMAN	HUMAN	Serine hydroxymethyltransferase, mitochondrial OS=Homo sapiens OX=9606 GN=SHMT2 PE=1 SV=3
sp|Q5VYK3|ECM29_HUMAN	HUMAN	Proteasome adapter and scaffold protein ECM29 OS=Homo sapiens OX=9606 GN=ECPAS PE=1 SV=2
sp|P52788|SPSY_HUMAN	HUMAN	Spermine synthase OS=Homo sapiens OX=9606 GN=SMS PE=1 SV=2
sp|Q96QK1|VPS35_HUMAN	HUMAN	Vacuolar protein sorting-associated protein 35 OS=Homo sapiens OX=9606 GN=VPS35 PE=1 SV=2
sp|P07741|APT_HUMAN	HUMAN	Adenine phosphoribosyltransferase OS=Homo sapiens OX=9606 GN=APRT PE=1 SV=2
sp|P25398|RS12_HUMAN	HUMAN	40S ribosomal protein S12 OS=Homo sapiens OX=9606 GN=RPS12 PE=1 SV=3
sp|Q15020|SART3_HUMAN	HUMAN	Squamous cell carcinoma antigen recognized by T-cells 3 OS=Homo sapiens OX=9606 GN=SART3 PE=1 SV=1
sp|P25098|ARBK1_HUMAN	HUMAN	Beta-adrenergic receptor kinase 1 OS=Homo sapiens OX=9606 GN=GRK2 PE=1 SV=2
sp|Q15084|PDIA6_HUMAN	HUMAN	Protein disulfide-isomerase A6 OS=Homo sapiens OX=9606 GN=PDIA6 PE=1 SV=1
sp|O75822|EIF3J_HUMAN	HUMAN	Eukaryotic translation initiation factor 3 subunit J OS=Homo sapiens OX=9606 GN=EIF3J PE=1 SV=2
sp|P23284|PPIB_HUMAN	HUMAN	Peptidyl-prolyl cis-trans isomerase B OS=Homo sapiens OX=9606 GN=PPIB PE=1 SV=2
sp|O15144|ARPC2_HUMAN	HUMAN	Actin-related protein 2/3 complex subunit 2 OS=Homo sapiens OX=9606 GN=ARPC2 PE=1 SV=1
sp|O15371|EIF3D_HUMAN	HUMAN	Eukaryotic translation initiation factor 3 subunit D OS=Homo sapiens OX=9606 GN=EIF3D PE=1 SV=1
sp|O00154|BACH_HUMAN	HUMAN	Cytosolic acyl coenzyme A thioester hydrolase OS=Homo sapiens OX=9606 GN=ACOT7 PE=1 SV=3

sp|P16152|CBR1_HUMAN	HUMAN	Carbonyl reductase [NADPH] 1 OS=Homo sapiens OX=9606 GN=CBR1 PE=1 SV=3
sp|Q14103|HNRPD_HUMAN	HUMAN	Heterogeneous nuclear ribonucleoprotein D0 OS=Homo sapiens OX=9606 GN=HNRNPD PE=1 SV=1
sp|P25788|PSA3_HUMAN	HUMAN	Proteasome subunit alpha type-3 OS=Homo sapiens OX=9606 GN=PSMA3 PE=1 SV=2
sp|P62241|RS8_HUMAN	HUMAN	40S ribosomal protein S8 OS=Homo sapiens OX=9606 GN=RPS8 PE=1 SV=2
sp|P40121|CAPG_HUMAN	HUMAN	Macrophage-capping protein OS=Homo sapiens OX=9606 GN=CAPG PE=1 SV=2
sp|P62805|H4_HUMAN	HUMAN	Histone H4 OS=Homo sapiens OX=9606 GN=H4C1 PE=1 SV=2
sp|P53041|PPP5_HUMAN	HUMAN	Serine/threonine-protein phosphatase 5 OS=Homo sapiens OX=9606 GN=PPP5C PE=1 SV=1
sp|P48444|COPD_HUMAN	HUMAN	Coatomer subunit delta OS=Homo sapiens OX=9606 GN=ARCN1 PE=1 SV=1
sp|P30048|PRDX3_HUMAN	HUMAN	Thioredoxin-dependent peroxide reductase, mitochondrial OS=Homo sapiens OX=9606 GN=PRDX3 PE=1 SV=3
sp|P27695|APEX1_HUMAN	HUMAN	DNA-(apurinic or apyrimidinic site) lyase OS=Homo sapiens OX=9606 GN=APEX1 PE=1 SV=2
sp|O75347|TBCA_HUMAN	HUMAN	Tubulin-specific chaperone A OS=Homo sapiens OX=9606 GN=TBCA PE=1 SV=3
sp|O00303|EIF3F_HUMAN	HUMAN	Eukaryotic translation initiation factor 3 subunit F OS=Homo sapiens OX=9606 GN=EIF3F PE=1 SV=1
sp|P22626|ROA2_HUMAN	HUMAN	Heterogeneous nuclear ribonucleoproteins A2/B1 OS=Homo sapiens OX=9606 GN=HNRNPA2B1 PE=1 SV=2
sp|P46781|RS9_HUMAN	HUMAN	40S ribosomal protein S9 OS=Homo sapiens OX=9606 GN=RPS9 PE=1 SV=3
sp|P19623|SPEE_HUMAN	HUMAN	Spermidine synthase OS=Homo sapiens OX=9606 GN=SRM PE=1 SV=1
sp|P02792|FRIL_HUMAN	HUMAN	Ferritin light chain OS=Homo sapiens OX=9606 GN=FTL PE=1 SV=2
sp|Q9Y696|CLIC4_HUMAN	HUMAN	Chloride intracellular channel protein 4 OS=Homo sapiens OX=9606 GN=CLIC4 PE=1 SV=4
sp|Q99873|ANM1_HUMAN	HUMAN	Protein arginine N-methyltransferase 1 OS=Homo sapiens OX=9606 GN=PRMT1 PE=1 SV=3
sp|P00492|HPRT_HUMAN	HUMAN	Hypoxanthine-guanine phosphoribosyltransferase OS=Homo sapiens OX=9606 GN=HPRT1 PE=1 SV=2
sp|Q13564|ULA1_HUMAN	HUMAN	NEDD8-activating enzyme E1 regulatory subunit OS=Homo sapiens OX=9606 GN=NAE1 PE=1 SV=1
sp|P18124|RL7_HUMAN	HUMAN	60S ribosomal protein L7 OS=Homo sapiens OX=9606 GN=RPL7 PE=1 SV=1
sp|P04899|GNAI2_HUMAN	HUMAN	Guanine nucleotide-binding protein G(i) subunit alpha-2 OS=Homo sapiens OX=9606 GN=GNAI2 PE=1 SV=3
sp|Q9BUJ2|HNRL1_HUMAN	HUMAN	Heterogeneous nuclear ribonucleoprotein U-like protein 1 OS=Homo sapiens OX=9606 GN=HNRNPUL1 PE=1 SV=2
sp|P78417|GSTO1_HUMAN	HUMAN	Glutathione S-transferase omega-1 OS=Homo sapiens OX=9606 GN=GSTO1 PE=1 SV=2
sp|P06748|NPM_HUMAN	HUMAN	Nucleophosmin OS=Homo sapiens OX=9606 GN=NPM1 PE=1 SV=2
sp|P51570|GALK1_HUMAN	HUMAN	Galactokinase OS=Homo sapiens OX=9606 GN=GALK1 PE=1 SV=1
sp|Q99536|VAT1_HUMAN	HUMAN	Synaptic vesicle membrane protein VAT-1 homolog OS=Homo sapiens OX=9606 GN=VAT1 PE=1 SV=2
sp|Q8N1G4|LRC47_HUMAN	HUMAN	Leucine-rich repeat-containing protein 47 OS=Homo sapiens OX=9606 GN=LRRC47 PE=1 SV=1
sp|Q9NUQ9|FA49B_HUMAN	HUMAN	Protein FAM49B OS=Homo sapiens OX=9606 GN=FAM49B PE=1 SV=1
sp|Q9HC38|GLOD4_HUMAN	HUMAN	Glyoxalase domain-containing protein 4 OS=Homo sapiens OX=9606 GN=GLOD4 PE=1 SV=1

sp|Q96C19|EFHD2_HUMAN	HUMAN	EF-hand domain-containing protein D2 OS=Homo sapiens OX=9606 GN=EFHD2 PE=1 SV=1
sp|P26599|PTBP1_HUMAN	HUMAN	Polypyrimidine tract-binding protein 1 OS=Homo sapiens OX=9606 GN=PTBP1 PE=1 SV=1
sp|P00505|AATM_HUMAN	HUMAN	Aspartate aminotransferase, mitochondrial OS=Homo sapiens OX=9606 GN=GOT2 PE=1 SV=3
sp|Q00341|VIGLN_HUMAN	HUMAN	Vigilin OS=Homo sapiens OX=9606 GN=HDLBP PE=1 SV=2
sp|O75153|CLU_HUMAN	HUMAN	Clustered mitochondria protein homolog OS=Homo sapiens OX=9606 GN=CLUH PE=1 SV=2
sp|P68400|CSK21_HUMAN	HUMAN	Casein kinase II subunit alpha OS=Homo sapiens OX=9606 GN=CSNK2A1 PE=1 SV=1
sp|P62906|RL10A_HUMAN	HUMAN	60S ribosomal protein L10a OS=Homo sapiens OX=9606 GN=RPL10A PE=1 SV=2
sp|Q99798|ACON_HUMAN	HUMAN	Aconitate hydratase, mitochondrial OS=Homo sapiens OX=9606 GN=ACO2 PE=1 SV=2
sp|Q14558|KPRA_HUMAN	HUMAN	Phosphoribosyl pyrophosphate synthase-associated protein 1 OS=Homo sapiens OX=9606 GN=PRPSAP1 PE=1 SV=2
sp|Q9UBE0|SAE1_HUMAN	HUMAN	SUMO-activating enzyme subunit 1 OS=Homo sapiens OX=9606 GN=SAE1 PE=1 SV=1
sp|P00441|SODC_HUMAN	HUMAN	Superoxide dismutase [Cu-Zn] OS=Homo sapiens OX=9606 GN=SOD1 PE=1 SV=2
sp|P31943|HNRH1_HUMAN	HUMAN	Heterogeneous nuclear ribonucleoprotein H OS=Homo sapiens OX=9606 GN=HNRNPH1 PE=1 SV=4
sp|Q15393|SF3B3_HUMAN	HUMAN	Splicing factor 3B subunit 3 OS=Homo sapiens OX=9606 GN=SF3B3 PE=1 SV=4
sp|Q9UJ70|NAGK_HUMAN	HUMAN	N-acetyl-D-glucosamine kinase OS=Homo sapiens OX=9606 GN=NAGK PE=1 SV=4
sp|P52292|IMA1_HUMAN	HUMAN	Importin subunit alpha-1 OS=Homo sapiens OX=9606 GN=KPNA2 PE=1 SV=1
sp|P17812|PYRG1_HUMAN	HUMAN	CTP synthase 1 OS=Homo sapiens OX=9606 GN=CTPS1 PE=1 SV=2
sp|P20042|IF2B_HUMAN	HUMAN	Eukaryotic translation initiation factor 2 subunit 2 OS=Homo sapiens OX=9606 GN=EIF2S2 PE=1 SV=2
sp|P48735|IDHP_HUMAN	HUMAN	Isocitrate dehydrogenase [NADP], mitochondrial OS=Homo sapiens OX=9606 GN=IDH2 PE=1 SV=2
sp|Q12905|ILF2_HUMAN	HUMAN	Interleukin enhancer-binding factor 2 OS=Homo sapiens OX=9606 GN=ILF2 PE=1 SV=2
sp|Q9NZL9|MAT2B_HUMAN	HUMAN	Methionine adenosyltransferase 2 subunit beta OS=Homo sapiens OX=9606 GN=MAT2B PE=1 SV=1
sp|P30520|PURA2_HUMAN	HUMAN	Adenylosuccinate synthetase isozyme 2 OS=Homo sapiens OX=9606 GN=ADSS2 PE=1 SV=3
sp|P54819|KAD2_HUMAN	HUMAN	Adenylate kinase 2, mitochondrial OS=Homo sapiens OX=9606 GN=AK2 PE=1 SV=2
sp|P62424|RL7A_HUMAN	HUMAN	60S ribosomal protein L7a OS=Homo sapiens OX=9606 GN=RPL7A PE=1 SV=2
sp|P12004|PCNA_HUMAN	HUMAN	Proliferating cell nuclear antigen OS=Homo sapiens OX=9606 GN=PCNA PE=1 SV=1
sp|P14324|FPPS_HUMAN	HUMAN	Farnesyl pyrophosphate synthase OS=Homo sapiens OX=9606 GN=FDPS PE=1 SV=4
sp|P67775|PP2AA_HUMAN	HUMAN	Serine/threonine-protein phosphatase 2A catalytic subunit alpha isoform OS=Homo sapiens OX=9606 GN=PPP2CA PE=1 SV=1
sp|P24752|THIL_HUMAN	HUMAN	Acetyl-CoA acetyltransferase, mitochondrial OS=Homo sapiens OX=9606 GN=ACAT1 PE=1 SV=1
sp|Q13435|SF3B2_HUMAN	HUMAN	Splicing factor 3B subunit 2 OS=Homo sapiens OX=9606 GN=SF3B2 PE=1 SV=2
sp|Q15365|PCBP1_HUMAN	HUMAN	Poly(rC)-binding protein 1 OS=Homo sapiens OX=9606 GN=PCBP1 PE=1 SV=2
sp|P62244|RS15A_HUMAN	HUMAN	40S ribosomal protein S15a OS=Homo sapiens OX=9606 GN=RPS15A PE=1 SV=2

sp|P05387|RLA2_HUMAN	HUMAN	60S acidic ribosomal protein P2 OS=Homo sapiens OX=9606 GN=RPLP2 PE=1 SV=1
sp|Q9UIA9|XPO7_HUMAN	HUMAN	Exportin-7 OS=Homo sapiens OX=9606 GN=XPO7 PE=1 SV=3
sp|P13693|TCTP_HUMAN	HUMAN	Translationally-controlled tumor protein OS=Homo sapiens OX=9606 GN=TPT1 PE=1 SV=1
sp|P20839|IMDH1_HUMAN	HUMAN	Inosine-5'-monophosphate dehydrogenase 1 OS=Homo sapiens OX=9606 GN=IMPDH1 PE=1 SV=2
sp|O95336|6PGL_HUMAN	HUMAN	6-phosphogluconolactonase OS=Homo sapiens OX=9606 GN=PGLS PE=1 SV=2
sp|P23246|SFPQ_HUMAN	HUMAN	Splicing factor, proline- and glutamine-rich OS=Homo sapiens OX=9606 GN=SFPQ PE=1 SV=2
sp|P51858|HDGF_HUMAN	HUMAN	Hepatoma-derived growth factor OS=Homo sapiens OX=9606 GN=HDGF PE=1 SV=1
sp|P07339|CATD_HUMAN	HUMAN	Cathepsin D OS=Homo sapiens OX=9606 GN=CTSD PE=1 SV=1
contam_sp|TRYP_PIG|		contam_sp|TRYP_PIG|
sp|P46976|GLYG_HUMAN	HUMAN	Glycogenin-1 OS=Homo sapiens OX=9606 GN=GYG1 PE=1 SV=4
sp|P32969|RL9_HUMAN	HUMAN	60S ribosomal protein L9 OS=Homo sapiens OX=9606 GN=RPL9 PE=1 SV=1
sp|Q53EL6|PDCD4_HUMAN	HUMAN	Programmed cell death protein 4 OS=Homo sapiens OX=9606 GN=PDCD4 PE=1 SV=2
sp|P55036|PSMD4_HUMAN	HUMAN	26S proteasome non-ATPase regulatory subunit 4 OS=Homo sapiens OX=9606 GN=PSMD4 PE=1 SV=1
sp|P24534|EF1B_HUMAN	HUMAN	Elongation factor 1-beta OS=Homo sapiens OX=9606 GN=EEF1B2 PE=1 SV=3
sp|Q9BT78|CSN4_HUMAN	HUMAN	COP9 signalosome complex subunit 4 OS=Homo sapiens OX=9606 GN=COPS4 PE=1 SV=1
sp|P53004|BIEA_HUMAN	HUMAN	Biliverdin reductase A OS=Homo sapiens OX=9606 GN=BLVRA PE=1 SV=2
sp|Q16851|UGPA_HUMAN	HUMAN	UTP--glucose-1-phosphate uridylyltransferase OS=Homo sapiens OX=9606 GN=UGP2 PE=1 SV=5
sp|P30044|PRDX5_HUMAN	HUMAN	Peroxiredoxin-5, mitochondrial OS=Homo sapiens OX=9606 GN=PRDX5 PE=1 SV=4
sp|P61221|ABCE1_HUMAN	HUMAN	ATP-binding cassette sub-family E member 1 OS=Homo sapiens OX=9606 GN=ABCE1 PE=1 SV=1
sp|P16278|BGAL_HUMAN	HUMAN	Beta-galactosidase OS=Homo sapiens OX=9606 GN=GLB1 PE=1 SV=2
sp|Q15631|TSN_HUMAN	HUMAN	Translin OS=Homo sapiens OX=9606 GN=TSN PE=1 SV=1
sp|Q9Y3F4|STRAP_HUMAN	HUMAN	Serine-threonine kinase receptor-associated protein OS=Homo sapiens OX=9606 GN=STRAP PE=1 SV=1
sp|Q53T59|H1BP3_HUMAN	HUMAN	HCLS1-binding protein 3 OS=Homo sapiens OX=9606 GN=HS1BP3 PE=1 SV=1
sp|Q92608|DOCK2_HUMAN	HUMAN	Dedicator of cytokinesis protein 2 OS=Homo sapiens OX=9606 GN=DOCK2 PE=1 SV=2
sp|O76003|GLRX3_HUMAN	HUMAN	Glutaredoxin-3 OS=Homo sapiens OX=9606 GN=GLRX3 PE=1 SV=2
sp|P13645|K1C10_HUMAN	HUMAN	Keratin, type I cytoskeletal 10 OS=Homo sapiens OX=9606 GN=KRT10 PE=1 SV=6
sp|Q13177|PAK2_HUMAN	HUMAN	Serine/threonine-protein kinase PAK 2 OS=Homo sapiens OX=9606 GN=PAK2 PE=1 SV=3
sp|Q99829|CPNE1_HUMAN	HUMAN	Copine-1 OS=Homo sapiens OX=9606 GN=CPNE1 PE=1 SV=1
sp|O43396|TXNL1_HUMAN	HUMAN	Thioredoxin-like protein 1 OS=Homo sapiens OX=9606 GN=TXNL1 PE=1 SV=3
sp|P25787|PSA2_HUMAN	HUMAN	Proteasome subunit alpha type-2 OS=Homo sapiens OX=9606 GN=PSMA2 PE=1 SV=2
sp|Q10567|AP1B1_HUMAN	HUMAN	AP-1 complex subunit beta-1 OS=Homo sapiens OX=9606 GN=AP1B1 PE=1 SV=2

sp|Q9Y5B9|SP16H_HUMAN	HUMAN	FACT complex subunit SPT16 OS=Homo sapiens OX=9606 GN=SUPT16H PE=1 SV=1
sp|Q9UMS4|PRP19_HUMAN	HUMAN	Pre-mRNA-processing factor 19 OS=Homo sapiens OX=9606 GN=PRPF19 PE=1 SV=1
sp|O75937|DNJC8_HUMAN	HUMAN	DnaJ homolog subfamily C member 8 OS=Homo sapiens OX=9606 GN=DNAJC8 PE=1 SV=2
sp|Q93009|UBP7_HUMAN	HUMAN	Ubiquitin carboxyl-terminal hydrolase 7 OS=Homo sapiens OX=9606 GN=USP7 PE=1 SV=2
sp|Q9UHD1|CHRD1_HUMAN	HUMAN	Cysteine and histidine-rich domain-containing protein 1 OS=Homo sapiens OX=9606 GN=CHORDC1 PE=1 SV=2
sp|P57737|CORO7_HUMAN	HUMAN	Coronin-7 OS=Homo sapiens OX=9606 GN=CORO7 PE=1 SV=2
sp|P25789|PSA4_HUMAN	HUMAN	Proteasome subunit alpha type-4 OS=Homo sapiens OX=9606 GN=PSMA4 PE=1 SV=1
sp|P63151|2ABA_HUMAN	HUMAN	Serine/threonine-protein phosphatase 2A 55 kDa regulatory subunit B alpha isoform OS=Homo sapiens OX=9606 GN=PPP2R2A PE=1 SV=1
sp|Q6P2Q9|PRP8_HUMAN	HUMAN	Pre-mRNA-processing-splicing factor 8 OS=Homo sapiens OX=9606 GN=PRPF8 PE=1 SV=2
sp|P54727|RD23B_HUMAN	HUMAN	UV excision repair protein RAD23 homolog B OS=Homo sapiens OX=9606 GN=RAD23B PE=1 SV=1
sp|P16949|STMN1_HUMAN	HUMAN	Stathmin OS=Homo sapiens OX=9606 GN=STMN1 PE=1 SV=3
sp|P61088|UBE2N_HUMAN	HUMAN	Ubiquitin-conjugating enzyme E2 N OS=Homo sapiens OX=9606 GN=UBE2N PE=1 SV=1
sp|P28062|PSB8_HUMAN	HUMAN	Proteasome subunit beta type-8 OS=Homo sapiens OX=9606 GN=PSMB8 PE=1 SV=3
sp|P39019|RS19_HUMAN	HUMAN	40S ribosomal protein S19 OS=Homo sapiens OX=9606 GN=RPS19 PE=1 SV=2
sp|Q14203|DCTN1_HUMAN	HUMAN	Dynactin subunit 1 OS=Homo sapiens OX=9606 GN=DCTN1 PE=1 SV=3
sp|Q08945|SSRP1_HUMAN	HUMAN	FACT complex subunit SSRP1 OS=Homo sapiens OX=9606 GN=SSRP1 PE=1 SV=1
sp|P54578|UBP14_HUMAN	HUMAN	Ubiquitin carboxyl-terminal hydrolase 14 OS=Homo sapiens OX=9606 GN=USP14 PE=1 SV=3
sp|Q9UNF0|PACN2_HUMAN	HUMAN	Protein kinase C and casein kinase substrate in neurons protein 2 OS=Homo sapiens OX=9606 GN=PACSIN2 PE=1 SV=2
sp|P29218|IMPA1_HUMAN	HUMAN	Inositol monophosphatase 1 OS=Homo sapiens OX=9606 GN=IMPA1 PE=1 SV=1
sp|Q13011|ECH1_HUMAN	HUMAN	Delta(3,5)-Delta(2,4)-dienoyl-CoA isomerase, mitochondrial OS=Homo sapiens OX=9606 GN=ECH1 PE=1 SV=2
sp|P80303|NUCB2_HUMAN	HUMAN	Nucleobindin-2 OS=Homo sapiens OX=9606 GN=NUCB2 PE=1 SV=3
sp|P61604|CH10_HUMAN	HUMAN	10 kDa heat shock protein, mitochondrial OS=Homo sapiens OX=9606 GN=HSPE1 PE=1 SV=2
sp|Q9UJU6|DBNL_HUMAN	HUMAN	Drebrin-like protein OS=Homo sapiens OX=9606 GN=DBNL PE=1 SV=1
sp|Q9BUF5|TBB6_HUMAN	HUMAN	Tubulin beta-6 chain OS=Homo sapiens OX=9606 GN=TUBB6 PE=1 SV=1
sp|P11216|PYGB_HUMAN	HUMAN	Glycogen phosphorylase, brain form OS=Homo sapiens OX=9606 GN=PYGB PE=1 SV=5
sp|Q15008|PSMD6_HUMAN	HUMAN	26S proteasome non-ATPase regulatory subunit 6 OS=Homo sapiens OX=9606 GN=PSMD6 PE=1 SV=1
sp|P30050|RL12_HUMAN	HUMAN	60S ribosomal protein L12 OS=Homo sapiens OX=9606 GN=RPL12 PE=1 SV=1
sp|P13861|KAP2_HUMAN	HUMAN	cAMP-dependent protein kinase type II-alpha regulatory subunit OS=Homo sapiens OX=9606 GN=PRKAR2A PE=1 SV=2

sp|P35268|RL22_HUMAN	HUMAN	60S ribosomal protein L22 OS=Homo sapiens OX=9606 GN=RPL22 PE=1 SV=2
sp|P35237|SPB6_HUMAN	HUMAN	Serpin B6 OS=Homo sapiens OX=9606 GN=SERPINB6 PE=1 SV=3
sp|Q9BTT0|AN32E_HUMAN	HUMAN	Acidic leucine-rich nuclear phosphoprotein 32 family member E OS=Homo sapiens OX=9606 GN=ANP32E PE=1 SV=1
sp|Q15437|SC23B_HUMAN	HUMAN	Protein transport protein Sec23B OS=Homo sapiens OX=9606 GN=SEC23B PE=1 SV=2
sp|P52565|GDIR1_HUMAN	HUMAN	Rho GDP-dissociation inhibitor 1 OS=Homo sapiens OX=9606 GN=ARHGDIA PE=1 SV=3
sp|P53999|TCP4_HUMAN	HUMAN	Activated RNA polymerase II transcriptional coactivator p15 OS=Homo sapiens OX=9606 GN=SUB1 PE=1 SV=3
sp|P46783|RS10_HUMAN	HUMAN	40S ribosomal protein S10 OS=Homo sapiens OX=9606 GN=RPS10 PE=1 SV=1
sp|O75663|TIPRL_HUMAN	HUMAN	TIP41-like protein OS=Homo sapiens OX=9606 GN=TIPRL PE=1 SV=2
sp|Q9UL46|PSME2_HUMAN	HUMAN	Proteasome activator complex subunit 2 OS=Homo sapiens OX=9606 GN=PSME2 PE=1 SV=4
sp|Q9NTZ6|RBM12_HUMAN	HUMAN	RNA-binding protein 12 OS=Homo sapiens OX=9606 GN=RBM12 PE=1 SV=1
sp|Q8NC51|PAIRB_HUMAN	HUMAN	Plasminogen activator inhibitor 1 RNA-binding protein OS=Homo sapiens OX=9606 GN=SERBP1 PE=1 SV=2
sp|P00813|ADA_HUMAN	HUMAN	Adenosine deaminase OS=Homo sapiens OX=9606 GN=ADA PE=1 SV=3
sp|O95757|HS74L_HUMAN	HUMAN	Heat shock 70 kDa protein 4L OS=Homo sapiens OX=9606 GN=HSPA4L PE=1 SV=3
sp|P48637|GSHB_HUMAN	HUMAN	Glutathione synthetase OS=Homo sapiens OX=9606 GN=GSS PE=1 SV=1
sp|P14735|IDE_HUMAN	HUMAN	Insulin-degrading enzyme OS=Homo sapiens OX=9606 GN=IDE PE=1 SV=4
sp|O75131|CPNE3_HUMAN	HUMAN	Copine-3 OS=Homo sapiens OX=9606 GN=CPNE3 PE=1 SV=1
sp|Q9H0D6|XRN2_HUMAN	HUMAN	5'-3' exoribonuclease 2 OS=Homo sapiens OX=9606 GN=XRN2 PE=1 SV=1
sp|P10768|ESTD_HUMAN	HUMAN	S-formylglutathione hydrolase OS=Homo sapiens OX=9606 GN=ESD PE=1 SV=2
sp|P49247|RPIA_HUMAN	HUMAN	Ribose-5-phosphate isomerase OS=Homo sapiens OX=9606 GN=RPIA PE=1 SV=3
sp|Q96T76|MMS19_HUMAN	HUMAN	MMS19 nucleotide excision repair protein homolog OS=Homo sapiens OX=9606 GN=MMS19 PE=1 SV=2
sp|P60228|EIF3E_HUMAN	HUMAN	Eukaryotic translation initiation factor 3 subunit E OS=Homo sapiens OX=9606 GN=EIF3E PE=1 SV=1
sp|O14929|HAT1_HUMAN	HUMAN	Histone acetyltransferase type B catalytic subunit OS=Homo sapiens OX=9606 GN=HAT1 PE=1 SV=1
sp|O75688|PPM1B_HUMAN	HUMAN	Protein phosphatase 1B OS=Homo sapiens OX=9606 GN=PPM1B PE=1 SV=1
sp|P51149|RAB7A_HUMAN	HUMAN	Ras-related protein Rab-7a OS=Homo sapiens OX=9606 GN=RAB7A PE=1 SV=1
sp|P59998|ARPC4_HUMAN	HUMAN	Actin-related protein 2/3 complex subunit 4 OS=Homo sapiens OX=9606 GN=ARPC4 PE=1 SV=3
sp|Q9HB07|MYG1_HUMAN	HUMAN	UPF0160 protein MYG1, mitochondrial OS=Homo sapiens OX=9606 GN=C12orf10 PE=1 SV=2
sp|P10155|RO60_HUMAN	HUMAN	60 kDa SS-A/Ro ribonucleoprotein OS=Homo sapiens OX=9606 GN=RO60 PE=1 SV=2
sp|P30043|BLVRB_HUMAN	HUMAN	Flavin reductase (NADPH) OS=Homo sapiens OX=9606 GN=BLVRB PE=1 SV=3
sp|Q15233|NONO_HUMAN	HUMAN	Non-POU domain-containing octamer-binding protein OS=Homo sapiens OX=9606 GN=NONO PE=1 SV=4
sp|Q8N163|CCAR2_HUMAN	HUMAN	Cell cycle and apoptosis regulator protein 2 OS=Homo sapiens OX=9606 GN=CCAR2 PE=1 SV=2

sp|P49721|PSB2_HUMAN	HUMAN	Proteasome subunit beta type-2 OS=Homo sapiens OX=9606 GN=PSMB2 PE=1 SV=1
sp|Q9H773|DCTP1_HUMAN	HUMAN	dCTP pyrophosphatase 1 OS=Homo sapiens OX=9606 GN=DCTPP1 PE=1 SV=1
sp|O15511|ARPC5_HUMAN	HUMAN	Actin-related protein 2/3 complex subunit 5 OS=Homo sapiens OX=9606 GN=ARPC5 PE=1 SV=3
sp|P62277|RS13_HUMAN	HUMAN	40S ribosomal protein S13 OS=Homo sapiens OX=9606 GN=RPS13 PE=1 SV=2
sp|Q7L576|CYFP1_HUMAN	HUMAN	Cytoplasmic FMR1-interacting protein 1 OS=Homo sapiens OX=9606 GN=CYFIP1 PE=1 SV=1
sp|Q9Y4E8|UBP15_HUMAN	HUMAN	Ubiquitin carboxyl-terminal hydrolase 15 OS=Homo sapiens OX=9606 GN=USP15 PE=1 SV=3
sp|P62993|GRB2_HUMAN	HUMAN	Growth factor receptor-bound protein 2 OS=Homo sapiens OX=9606 GN=GRB2 PE=1 SV=1
sp|P49720|PSB3_HUMAN	HUMAN	Proteasome subunit beta type-3 OS=Homo sapiens OX=9606 GN=PSMB3 PE=1 SV=2
sp|Q16222|UAP1_HUMAN	HUMAN	UDP-N-acetylhexosamine pyrophosphorylase OS=Homo sapiens OX=9606 GN=UAP1 PE=1 SV=3
sp|P62191|PRS4_HUMAN	HUMAN	26S proteasome regulatory subunit 4 OS=Homo sapiens OX=9606 GN=PSMC1 PE=1 SV=1
sp|Q9NQR4|NIT2_HUMAN	HUMAN	Omega-amidase NIT2 OS=Homo sapiens OX=9606 GN=NIT2 PE=1 SV=1
sp|P16403|H12_HUMAN	HUMAN	Histone H1.2 OS=Homo sapiens OX=9606 GN=H1-2 PE=1 SV=2
sp|P62328|TYB4_HUMAN	HUMAN	Thymosin beta-4 OS=Homo sapiens OX=9606 GN=TMSB4X PE=1 SV=2
sp|O75368|SH3L1_HUMAN	HUMAN	SH3 domain-binding glutamic acid-rich-like protein OS=Homo sapiens OX=9606 GN=SH3BGRL PE=1 SV=1
sp|Q9Y5K5|UCHL5_HUMAN	HUMAN	Ubiquitin carboxyl-terminal hydrolase isozyme L5 OS=Homo sapiens OX=9606 GN=UCHL5 PE=1 SV=3
sp|P17844|DDX5_HUMAN	HUMAN	Probable ATP-dependent RNA helicase DDX5 OS=Homo sapiens OX=9606 GN=DDX5 PE=1 SV=1
sp|P28070|PSB4_HUMAN	HUMAN	Proteasome subunit beta type-4 OS=Homo sapiens OX=9606 GN=PSMB4 PE=1 SV=4
sp|Q8TEX9|IPO4_HUMAN	HUMAN	Importin-4 OS=Homo sapiens OX=9606 GN=IPO4 PE=1 SV=2
sp|Q9Y224|RTRAF_HUMAN	HUMAN	RNA transcription, translation and transport factor protein OS=Homo sapiens OX=9606 GN=RTRAF PE=1 SV=1
sp|P63000|RAC1_HUMAN	HUMAN	Ras-related C3 botulinum toxin substrate 1 OS=Homo sapiens OX=9606 GN=RAC1 PE=1 SV=1
sp|Q15691|MARE1_HUMAN	HUMAN	Microtubule-associated protein RP/EB family member 1 OS=Homo sapiens OX=9606 GN=MAPRE1 PE=1 SV=3
sp|P43034|LIS1_HUMAN	HUMAN	Platelet-activating factor acetylhydrolase IB subunit alpha OS=Homo sapiens OX=9606 GN=PAFAH1B1 PE=1 SV=2
sp|Q9ULV4|COR1C_HUMAN	HUMAN	Coronin-1C OS=Homo sapiens OX=9606 GN=CORO1C PE=1 SV=1
sp|Q9UQE7|SMC3_HUMAN	HUMAN	Structural maintenance of chromosomes protein 3 OS=Homo sapiens OX=9606 GN=SMC3 PE=1 SV=2
sp|Q15942|ZYX_HUMAN	HUMAN	Zyxin OS=Homo sapiens OX=9606 GN=ZYX PE=1 SV=1
sp|P34896|GLYC_HUMAN	HUMAN	Serine hydroxymethyltransferase, cytosolic OS=Homo sapiens OX=9606 GN=SHMT1 PE=1 SV=1
sp|P15104|GLNA_HUMAN	HUMAN	Glutamine synthetase OS=Homo sapiens OX=9606 GN=GLUL PE=1 SV=4
sp|P62495|ERF1_HUMAN	HUMAN	Eukaryotic peptide chain release factor subunit 1 OS=Homo sapiens OX=9606 GN=ETF1 PE=1 SV=3
sp|P55010|IF5_HUMAN	HUMAN	Eukaryotic translation initiation factor 5 OS=Homo sapiens OX=9606 GN=EIF5 PE=1 SV=2

sp|O43847|NRDC_HUMAN	HUMAN	Nardilysin OS=Homo sapiens OX=9606 GN=NRDC PE=1 SV=3
sp|P50225|ST1A1_HUMAN	HUMAN	Sulfotransferase 1A1 OS=Homo sapiens OX=9606 GN=SULT1A1 PE=1 SV=3
sp|O96019|ACL6A_HUMAN	HUMAN	Actin-like protein 6A OS=Homo sapiens OX=9606 GN=ACTL6A PE=1 SV=1
sp|Q96SB4|SRPK1_HUMAN	HUMAN	SRSF protein kinase 1 OS=Homo sapiens OX=9606 GN=SRPK1 PE=1 SV=2
sp|P46060|RAGP1_HUMAN	HUMAN	Ran GTPase-activating protein 1 OS=Homo sapiens OX=9606 GN=RANGAP1 PE=1 SV=1
sp|Q8NBS9|TXND5_HUMAN	HUMAN	Thioredoxin domain-containing protein 5 OS=Homo sapiens OX=9606 GN=TXNDC5 PE=1 SV=2
sp|P31350|RIR2_HUMAN	HUMAN	Ribonucleoside-diphosphate reductase subunit M2 OS=Homo sapiens OX=9606 GN=RRM2 PE=1 SV=1
sp|P52701|MSH6_HUMAN	HUMAN	DNA mismatch repair protein Msh6 OS=Homo sapiens OX=9606 GN=MSH6 PE=1 SV=2
sp|Q99598|TSNAX_HUMAN	HUMAN	Translin-associated protein X OS=Homo sapiens OX=9606 GN=TSNAX PE=1 SV=1
sp|Q8TBC4|UBA3_HUMAN	HUMAN	NEDD8-activating enzyme E1 catalytic subunit OS=Homo sapiens OX=9606 GN=UBA3 PE=1 SV=2
sp|P38919|IF4A3_HUMAN	HUMAN	Eukaryotic initiation factor 4A-III OS=Homo sapiens OX=9606 GN=EIF4A3 PE=1 SV=4
sp|Q9UKK9|NUDT5_HUMAN	HUMAN	ADP-sugar pyrophosphatase OS=Homo sapiens OX=9606 GN=NUDT5 PE=1 SV=1
sp|Q12874|SF3A3_HUMAN	HUMAN	Splicing factor 3A subunit 3 OS=Homo sapiens OX=9606 GN=SF3A3 PE=1 SV=1
sp|P67809|YBOX1_HUMAN	HUMAN	Y-box-binding protein 1 OS=Homo sapiens OX=9606 GN=YBX1 PE=1 SV=3
sp|P30533|AMRP_HUMAN	HUMAN	Alpha-2-macroglobulin receptor-associated protein OS=Homo sapiens OX=9606 GN=LRPAP1 PE=1 SV=1
sp|Q92688|AN32B_HUMAN	HUMAN	Acidic leucine-rich nuclear phosphoprotein 32 family member B OS=Homo sapiens OX=9606 GN=ANP32B PE=1 SV=1
sp|Q15366|PCBP2_HUMAN	HUMAN	Poly(rC)-binding protein 2 OS=Homo sapiens OX=9606 GN=PCBP2 PE=1 SV=1
sp|P61086|UBE2K_HUMAN	HUMAN	Ubiquitin-conjugating enzyme E2 K OS=Homo sapiens OX=9606 GN=UBE2K PE=1 SV=3
sp|P09661|RU2A_HUMAN	HUMAN	U2 small nuclear ribonucleoprotein A' OS=Homo sapiens OX=9606 GN=SNRPA1 PE=1 SV=2
sp|Q9NR50|EI2BG_HUMAN	HUMAN	Translation initiation factor eIF-2B subunit gamma OS=Homo sapiens OX=9606 GN=EIF2B3 PE=1 SV=1
sp|P62753|RS6_HUMAN	HUMAN	40S ribosomal protein S6 OS=Homo sapiens OX=9606 GN=RPS6 PE=1 SV=1
sp|O94903|PLPHP_HUMAN	HUMAN	Pyridoxal phosphate homeostasis protein OS=Homo sapiens OX=9606 GN=PLPBP PE=1 SV=1
sp|Q9NQP4|PFD4_HUMAN	HUMAN	Prefoldin subunit 4 OS=Homo sapiens OX=9606 GN=PFDN4 PE=1 SV=1
sp|P31946|1433B_HUMAN	HUMAN	14-3-3 protein beta/alpha OS=Homo sapiens OX=9606 GN=YWHAB PE=1 SV=3
sp|P06454|PTMA_HUMAN	HUMAN	Prothymosin alpha OS=Homo sapiens OX=9606 GN=PTMA PE=1 SV=2
sp|P52272|HNRPM_HUMAN	HUMAN	Heterogeneous nuclear ribonucleoprotein M OS=Homo sapiens OX=9606 GN=HNRNPM PE=1 SV=3
sp|O60256|KPRB_HUMAN	HUMAN	Phosphoribosyl pyrophosphate synthase-associated protein 2 OS=Homo sapiens OX=9606 GN=PRPSAP2 PE=1 SV=1
sp|Q9BTE6|AASD1_HUMAN	HUMAN	Alanyl-tRNA editing protein Aarsd1 OS=Homo sapiens OX=9606 GN=AARSD1 PE=1 SV=2
sp|O14744|ANM5_HUMAN	HUMAN	Protein arginine N-methyltransferase 5 OS=Homo sapiens OX=9606 GN=PRMT5 PE=1 SV=4
sp|P08670|VIME_HUMAN	HUMAN	Vimentin OS=Homo sapiens OX=9606 GN=VIM PE=1 SV=4

sp|P09382|LEG1_HUMAN	HUMAN	Galectin-1 OS=Homo sapiens OX=9606 GN=LGALS1 PE=1 SV=2
sp|P61289|PSME3_HUMAN	HUMAN	Proteasome activator complex subunit 3 OS=Homo sapiens OX=9606 GN=PSME3 PE=1 SV=1
sp|P46459|NSF_HUMAN	HUMAN	Vesicle-fusing ATPase OS=Homo sapiens OX=9606 GN=NSF PE=1 SV=3
sp|P62888|RL30_HUMAN	HUMAN	60S ribosomal protein L30 OS=Homo sapiens OX=9606 GN=RPL30 PE=1 SV=2
sp|P61201|CSN2_HUMAN	HUMAN	COP9 signalosome complex subunit 2 OS=Homo sapiens OX=9606 GN=COPS2 PE=1 SV=1
sp|P30040|ERP29_HUMAN	HUMAN	Endoplasmic reticulum resident protein 29 OS=Homo sapiens OX=9606 GN=ERP29 PE=1 SV=4
sp|P35606|COPB2_HUMAN	HUMAN	Coatomer subunit beta' OS=Homo sapiens OX=9606 GN=COPB2 PE=1 SV=2
sp|O95352|ATG7_HUMAN	HUMAN	Ubiquitin-like modifier-activating enzyme ATG7 OS=Homo sapiens OX=9606 GN=ATG7 PE=1 SV=1
sp|P13804|ETFA_HUMAN	HUMAN	Electron transfer flavoprotein subunit alpha, mitochondrial OS=Homo sapiens OX=9606 GN=ETFA PE=1 SV=1
sp|P07910|HNRPC_HUMAN	HUMAN	Heterogeneous nuclear ribonucleoproteins C1/C2 OS=Homo sapiens OX=9606 GN=HNRNPC PE=1 SV=4
sp|Q8IYD1|ERF3B_HUMAN	HUMAN	Eukaryotic peptide chain release factor GTP-binding subunit ERF3B OS=Homo sapiens OX=9606 GN=GSPT2 PE=1 SV=2
sp|P37108|SRP14_HUMAN	HUMAN	Signal recognition particle 14 kDa protein OS=Homo sapiens OX=9606 GN=SRP14 PE=1 SV=2
sp|P53618|COPB_HUMAN	HUMAN	Coatomer subunit beta OS=Homo sapiens OX=9606 GN=COPB1 PE=1 SV=3
sp|O75312|ZPR1_HUMAN	HUMAN	Zinc finger protein ZPR1 OS=Homo sapiens OX=9606 GN=ZPR1 PE=1 SV=1
sp|P14866|HNRPL_HUMAN	HUMAN	Heterogeneous nuclear ribonucleoprotein L OS=Homo sapiens OX=9606 GN=HNRNPL PE=1 SV=2
sp|Q9NT62|ATG3_HUMAN	HUMAN	Ubiquitin-like-conjugating enzyme ATG3 OS=Homo sapiens OX=9606 GN=ATG3 PE=1 SV=1
sp|P07954|FUMH_HUMAN	HUMAN	Fumarate hydratase, mitochondrial OS=Homo sapiens OX=9606 GN=FH PE=1 SV=3
sp|Q8NE71|ABCF1_HUMAN	HUMAN	ATP-binding cassette sub-family F member 1 OS=Homo sapiens OX=9606 GN=ABCF1 PE=1 SV=2
sp|O95433|AHSA1_HUMAN	HUMAN	Activator of 90 kDa heat shock protein ATPase homolog 1 OS=Homo sapiens OX=9606 GN=AHSA1 PE=1 SV=1
sp|Q9UBQ7|GRHPR_HUMAN	HUMAN	Glyoxylate reductase/hydroxypyruvate reductase OS=Homo sapiens OX=9606 GN=GRHPR PE=1 SV=1
sp|Q13347|EIF3I_HUMAN	HUMAN	Eukaryotic translation initiation factor 3 subunit I OS=Homo sapiens OX=9606 GN=EIF3I PE=1 SV=1
sp|Q2NL82|TSR1_HUMAN	HUMAN	Pre-rRNA-processing protein TSR1 homolog OS=Homo sapiens OX=9606 GN=TSR1 PE=1 SV=1
sp|P41240|CSK_HUMAN	HUMAN	Tyrosine-protein kinase CSK OS=Homo sapiens OX=9606 GN=CSK PE=1 SV=1
sp|Q9UI10|EI2BD_HUMAN	HUMAN	Translation initiation factor eIF-2B subunit delta OS=Homo sapiens OX=9606 GN=EIF2B4 PE=1 SV=2
sp|Q7Z4W1|DCXR_HUMAN	HUMAN	L-xylulose reductase OS=Homo sapiens OX=9606 GN=DCXR PE=1 SV=2
sp|P61353|RL27_HUMAN	HUMAN	60S ribosomal protein L27 OS=Homo sapiens OX=9606 GN=RPL27 PE=1 SV=2
sp|P47755|CAZA2_HUMAN	HUMAN	F-actin-capping protein subunit alpha-2 OS=Homo sapiens OX=9606 GN=CAPZA2 PE=1 SV=3
sp|P09972|ALDOC_HUMAN	HUMAN	Fructose-bisphosphate aldolase C OS=Homo sapiens OX=9606 GN=ALDOC PE=1 SV=2
sp|O75390|CISY_HUMAN	HUMAN	Citrate synthase, mitochondrial OS=Homo sapiens OX=9606 GN=CS PE=1 SV=2

sp|P20290|BTF3_HUMAN	HUMAN	Transcription factor BTF3 OS=Homo sapiens OX=9606 GN=BTF3 PE=1 SV=1
sp|Q9Y3I0|RTCB_HUMAN	HUMAN	RNA-splicing ligase RtcB homolog OS=Homo sapiens OX=9606 GN=RTCB PE=1 SV=1
sp|Q99714|HCD2_HUMAN	HUMAN	3-hydroxyacyl-CoA dehydrogenase type-2 OS=Homo sapiens OX=9606 GN=HSD17B10 PE=1 SV=3
sp|Q15717|ELAV1_HUMAN	HUMAN	ELAV-like protein 1 OS=Homo sapiens OX=9606 GN=ELAVL1 PE=1 SV=2
sp|Q92900|RENT1_HUMAN	HUMAN	Regulator of nonsense transcripts 1 OS=Homo sapiens OX=9606 GN=UPF1 PE=1 SV=2
sp|Q9Y383|LC7L2_HUMAN	HUMAN	Putative RNA-binding protein Luc7-like 2 OS=Homo sapiens OX=9606 GN=LUC7L2 PE=1 SV=2
sp|P00966|ASSY_HUMAN	HUMAN	Argininosuccinate synthase OS=Homo sapiens OX=9606 GN=ASS1 PE=1 SV=2
sp|Q99880|H2B1L_HUMAN	HUMAN	Histone H2B type 1-L OS=Homo sapiens OX=9606 GN=H2BC13 PE=1 SV=3
sp|O43399|TPD54_HUMAN	HUMAN	Tumor protein D54 OS=Homo sapiens OX=9606 GN=TPD52L2 PE=1 SV=2
sp|Q9GZP4|PITH1_HUMAN	HUMAN	PITH domain-containing protein 1 OS=Homo sapiens OX=9606 GN=PITHD1 PE=1 SV=1
sp|Q00688|FKBP3_HUMAN	HUMAN	Peptidyl-prolyl cis-trans isomerase FKBP3 OS=Homo sapiens OX=9606 GN=FKBP3 PE=1 SV=1
sp|P07686|HEXB_HUMAN	HUMAN	Beta-hexosaminidase subunit beta OS=Homo sapiens OX=9606 GN=HEXB PE=1 SV=3
sp|Q9P289|STK26_HUMAN	HUMAN	Serine/threonine-protein kinase 26 OS=Homo sapiens OX=9606 GN=STK26 PE=1 SV=2
sp|P31153|METK2_HUMAN	HUMAN	S-adenosylmethionine synthase isoform type-2 OS=Homo sapiens OX=9606 GN=MAT2A PE=1 SV=1
sp|P30046|DOPD_HUMAN	HUMAN	D-dopachrome decarboxylase OS=Homo sapiens OX=9606 GN=DDT PE=1 SV=3
sp|Q9Y678|COPG1_HUMAN	HUMAN	Coatomer subunit gamma-1 OS=Homo sapiens OX=9606 GN=COPG1 PE=1 SV=1
sp|P15531|NDKA_HUMAN	HUMAN	Nucleoside diphosphate kinase A OS=Homo sapiens OX=9606 GN=NME1 PE=1 SV=1
sp|P06400|RB_HUMAN	HUMAN	Retinoblastoma-associated protein OS=Homo sapiens OX=9606 GN=RB1 PE=1 SV=2
sp|Q14232|EI2BA_HUMAN	HUMAN	Translation initiation factor eIF-2B subunit alpha OS=Homo sapiens OX=9606 GN=EIF2B1 PE=1 SV=1
sp|P62857|RS28_HUMAN	HUMAN	40S ribosomal protein S28 OS=Homo sapiens OX=9606 GN=RPS28 PE=1 SV=1
sp|Q14258|TRI25_HUMAN	HUMAN	E3 ubiquitin/ISG15 ligase TRIM25 OS=Homo sapiens OX=9606 GN=TRIM25 PE=1 SV=2
sp|Q9ULC4|MCTS1_HUMAN	HUMAN	Malignant T-cell-amplified sequence 1 OS=Homo sapiens OX=9606 GN=MCTS1 PE=1 SV=1
sp|O00273|DFFA_HUMAN	HUMAN	DNA fragmentation factor subunit alpha OS=Homo sapiens OX=9606 GN=DFFA PE=1 SV=1
sp|O75534|CSDE1_HUMAN	HUMAN	Cold shock domain-containing protein E1 OS=Homo sapiens OX=9606 GN=CSDE1 PE=1 SV=2
sp|Q9BTE3|MCMBP_HUMAN	HUMAN	Mini-chromosome maintenance complex-binding protein OS=Homo sapiens OX=9606 GN=MCMBP PE=1 SV=2
sp|Q92973|TNPO1_HUMAN	HUMAN	Transportin-1 OS=Homo sapiens OX=9606 GN=TNPO1 PE=1 SV=2
sp|Q13098|CSN1_HUMAN	HUMAN	COP9 signalosome complex subunit 1 OS=Homo sapiens OX=9606 GN=GPS1 PE=1 SV=4
sp|P26358|DNMT1_HUMAN	HUMAN	DNA (cytosine-5)-methyltransferase 1 OS=Homo sapiens OX=9606 GN=DNMT1 PE=1 SV=2
sp|O95782|AP2A1_HUMAN	HUMAN	AP-2 complex subunit alpha-1 OS=Homo sapiens OX=9606 GN=AP2A1 PE=1 SV=3
sp|Q7L014|DDX46_HUMAN	HUMAN	Probable ATP-dependent RNA helicase DDX46 OS=Homo sapiens OX=9606 GN=DDX46 PE=1 SV=2

sp|Q04917|1433F_HUMAN	HUMAN	14-3-3 protein eta OS=Homo sapiens OX=9606 GN=YWHAH PE=1 SV=4
sp|Q92556|ELMO1_HUMAN	HUMAN	Engulfment and cell motility protein 1 OS=Homo sapiens OX=9606 GN=ELMO1 PE=1 SV=2
sp|P49903|SPS1_HUMAN	HUMAN	Selenide, water dikinase 1 OS=Homo sapiens OX=9606 GN=SEPHS1 PE=1 SV=2
sp|Q15003|CND2_HUMAN	HUMAN	Condensin complex subunit 2 OS=Homo sapiens OX=9606 GN=NCAPH PE=1 SV=3
sp|Q8TAQ2|SMRC2_HUMAN	HUMAN	SWI/SNF complex subunit SMARCC2 OS=Homo sapiens OX=9606 GN=SMARCC2 PE=1 SV=1
sp|P10599|THIO_HUMAN	HUMAN	Thioredoxin OS=Homo sapiens OX=9606 GN=TXN PE=1 SV=3
sp|Q9NR45|SIAS_HUMAN	HUMAN	Sialic acid synthase OS=Homo sapiens OX=9606 GN=NANS PE=1 SV=2
sp|Q15459|SF3A1_HUMAN	HUMAN	Splicing factor 3A subunit 1 OS=Homo sapiens OX=9606 GN=SF3A1 PE=1 SV=1
sp|O00487|PSDE_HUMAN	HUMAN	26S proteasome non-ATPase regulatory subunit 14 OS=Homo sapiens OX=9606 GN=PSMD14 PE=1 SV=1
sp|Q02543|RL18A_HUMAN	HUMAN	60S ribosomal protein L18a OS=Homo sapiens OX=9606 GN=RPL18A PE=1 SV=2
sp|Q7Z4H3|HDDC2_HUMAN	HUMAN	HD domain-containing protein 2 OS=Homo sapiens OX=9606 GN=HDDC2 PE=1 SV=1
sp|P04424|ARLY_HUMAN	HUMAN	Argininosuccinate lyase OS=Homo sapiens OX=9606 GN=ASL PE=1 SV=4
sp|P78347|GTF2I_HUMAN	HUMAN	General transcription factor II-I OS=Homo sapiens OX=9606 GN=GTF2I PE=1 SV=2
sp|Q32MZ4|LRRF1_HUMAN	HUMAN	Leucine-rich repeat flightless-interacting protein 1 OS=Homo sapiens OX=9606 GN=LRRFIP1 PE=1 SV=2
sp|O43592|XPOT_HUMAN	HUMAN	Exportin-T OS=Homo sapiens OX=9606 GN=XPOT PE=1 SV=2
sp|P63220|RS21_HUMAN	HUMAN	40S ribosomal protein S21 OS=Homo sapiens OX=9606 GN=RPS21 PE=1 SV=1
sp|Q01581|HMCS1_HUMAN	HUMAN	Hydroxymethylglutaryl-CoA synthase, cytoplasmic OS=Homo sapiens OX=9606 GN=HMGCS1 PE=1 SV=2
sp|P27707|DCK_HUMAN	HUMAN	Deoxycytidine kinase OS=Homo sapiens OX=9606 GN=DCK PE=1 SV=1
sp|P48506|GSH1_HUMAN	HUMAN	Glutamate--cysteine ligase catalytic subunit OS=Homo sapiens OX=9606 GN=GCLC PE=1 SV=2
sp|Q13630|FCL_HUMAN	HUMAN	GDP-L-fucose synthase OS=Homo sapiens OX=9606 GN=TSTA3 PE=1 SV=1
sp|P09488|GSTM1_HUMAN	HUMAN	Glutathione S-transferase Mu 1 OS=Homo sapiens OX=9606 GN=GSTM1 PE=1 SV=3
sp|P60953|CDC42_HUMAN	HUMAN	Cell division control protein 42 homolog OS=Homo sapiens OX=9606 GN=CDC42 PE=1 SV=2
sp|P27694|RFA1_HUMAN	HUMAN	Replication protein A 70 kDa DNA-binding subunit OS=Homo sapiens OX=9606 GN=RPA1 PE=1 SV=2
sp|P17655|CAN2_HUMAN	HUMAN	Calpain-2 catalytic subunit OS=Homo sapiens OX=9606 GN=CAPN2 PE=1 SV=6
sp|Q9NZL4|HPBP1_HUMAN	HUMAN	Hsp70-binding protein 1 OS=Homo sapiens OX=9606 GN=HSPBP1 PE=1 SV=2
sp|Q99439|CNN2_HUMAN	HUMAN	Calponin-2 OS=Homo sapiens OX=9606 GN=CNN2 PE=1 SV=4
sp|Q9Y6E2|BZW2_HUMAN	HUMAN	Basic leucine zipper and W2 domain-containing protein 2 OS=Homo sapiens OX=9606 GN=BZW2 PE=1 SV=1
sp|Q16401|PSMD5_HUMAN	HUMAN	26S proteasome non-ATPase regulatory subunit 5 OS=Homo sapiens OX=9606 GN=PSMD5 PE=1 SV=3
sp|Q13155|AIMP2_HUMAN	HUMAN	Aminoacyl tRNA synthase complex-interacting multifunctional protein 2 OS=Homo sapiens OX=9606 GN=AIMP2 PE=1 SV=2
sp|P61758|PFD3_HUMAN	HUMAN	Prefoldin subunit 3 OS=Homo sapiens OX=9606 GN=VBP1 PE=1 SV=4

sp|Q9UMX0|UBQL1_HUMAN	HUMAN	Ubiquilin-1 OS=Homo sapiens OX=9606 GN=UBQLN1 PE=1 SV=2
sp|Q9H2J4|PDCL3_HUMAN	HUMAN	Phosducin-like protein 3 OS=Homo sapiens OX=9606 GN=PDCL3 PE=1 SV=1
sp|P60660|MYL6_HUMAN	HUMAN	Myosin light polypeptide 6 OS=Homo sapiens OX=9606 GN=MYL6 PE=1 SV=2
sp|P16885|PLCG2_HUMAN	HUMAN	1-phosphatidylinositol 4,5-bisphosphate phosphodiesterase gamma-2 OS=Homo sapiens OX=9606 GN=PLCG2 PE=1 SV=4
sp|P52888|THOP1_HUMAN	HUMAN	Thimet oligopeptidase OS=Homo sapiens OX=9606 GN=THOP1 PE=1 SV=2
sp|P48426|PI42A_HUMAN	HUMAN	Phosphatidylinositol 5-phosphate 4-kinase type-2 alpha OS=Homo sapiens OX=9606 GN=PIP4K2A PE=1 SV=2
sp|Q16181|SEPT7_HUMAN	HUMAN	Septin-7 OS=Homo sapiens OX=9606 GN=SEPTIN7 PE=1 SV=2
sp|P62913|RL11_HUMAN	HUMAN	60S ribosomal protein L11 OS=Homo sapiens OX=9606 GN=RPL11 PE=1 SV=2
sp|O94819|KBTBB_HUMAN	HUMAN	Kelch repeat and BTB domain-containing protein 11 OS=Homo sapiens OX=9606 GN=KBTBD11 PE=1 SV=1
sp|O43447|PPIH_HUMAN	HUMAN	Peptidyl-prolyl cis-trans isomerase H OS=Homo sapiens OX=9606 GN=PPIH PE=1 SV=1
sp|O43488|ARK72_HUMAN	HUMAN	Aflatoxin B1 aldehyde reductase member 2 OS=Homo sapiens OX=9606 GN=AKR7A2 PE=1 SV=3
sp|Q00534|CDK6_HUMAN	HUMAN	Cyclin-dependent kinase 6 OS=Homo sapiens OX=9606 GN=CDK6 PE=1 SV=1
sp|O43390|HNRPR_HUMAN	HUMAN	Heterogeneous nuclear ribonucleoprotein R OS=Homo sapiens OX=9606 GN=HNRNPR PE=1 SV=1
sp|P07108|ACBP_HUMAN	HUMAN	Acyl-CoA-binding protein OS=Homo sapiens OX=9606 GN=DBI PE=1 SV=2
sp|P62263|RS14_HUMAN	HUMAN	40S ribosomal protein S14 OS=Homo sapiens OX=9606 GN=RPS14 PE=1 SV=3
sp|P11766|ADHX_HUMAN	HUMAN	Alcohol dehydrogenase class-3 OS=Homo sapiens OX=9606 GN=ADH5 PE=1 SV=4
sp|Q01433|AMPD2_HUMAN	HUMAN	AMP deaminase 2 OS=Homo sapiens OX=9606 GN=AMPD2 PE=1 SV=2
sp|P15374|UCHL3_HUMAN	HUMAN	Ubiquitin carboxyl-terminal hydrolase isozyme L3 OS=Homo sapiens OX=9606 GN=UCHL3 PE=1 SV=1
sp|Q04637|IF4G1_HUMAN	HUMAN	Eukaryotic translation initiation factor 4 gamma 1 OS=Homo sapiens OX=9606 GN=EIF4G1 PE=1 SV=4
sp|Q9NYL9|TMOD3_HUMAN	HUMAN	Tropomodulin-3 OS=Homo sapiens OX=9606 GN=TMOD3 PE=1 SV=1
sp|P39748|FEN1_HUMAN	HUMAN	Flap endonuclease 1 OS=Homo sapiens OX=9606 GN=FEN1 PE=1 SV=1
sp|Q9BTM1|H2AJ_HUMAN	HUMAN	Histone H2A.J OS=Homo sapiens OX=9606 GN=H2AFJ PE=1 SV=1
sp|P10644|KAP0_HUMAN	HUMAN	cAMP-dependent protein kinase type I-alpha regulatory subunit OS=Homo sapiens OX=9606 GN=PRKAR1A PE=1 SV=1
sp|P51812|KS6A3_HUMAN	HUMAN	Ribosomal protein S6 kinase alpha-3 OS=Homo sapiens OX=9606 GN=RPS6KA3 PE=1 SV=1
sp|O15212|PFD6_HUMAN	HUMAN	Prefoldin subunit 6 OS=Homo sapiens OX=9606 GN=PFDN6 PE=1 SV=1
sp|P61163|ACTZ_HUMAN	HUMAN	Alpha-centractin OS=Homo sapiens OX=9606 GN=ACTR1A PE=1 SV=1
sp|Q16836|HCDH_HUMAN	HUMAN	Hydroxyacyl-coenzyme A dehydrogenase, mitochondrial OS=Homo sapiens OX=9606 GN=HADH PE=1 SV=3
sp|Q14847|LASP1_HUMAN	HUMAN	LIM and SH3 domain protein 1 OS=Homo sapiens OX=9606 GN=LASP1 PE=1 SV=2

sp|P22061|PIMT_HUMAN	HUMAN	Protein-L-isoaspartate(D-aspartate) O-methyltransferase OS=Homo sapiens OX=9606 GN=PCMT1 PE=1 SV=4
sp|O00442|RTCA_HUMAN	HUMAN	RNA 3'-terminal phosphate cyclase OS=Homo sapiens OX=9606 GN=RTCA PE=1 SV=1
sp|Q15435|PP1R7_HUMAN	HUMAN	Protein phosphatase 1 regulatory subunit 7 OS=Homo sapiens OX=9606 GN=PPP1R7 PE=1 SV=1
sp|Q16630|CPSF6_HUMAN	HUMAN	Cleavage and polyadenylation specificity factor subunit 6 OS=Homo sapiens OX=9606 GN=CPSF6 PE=1 SV=2
sp|P13716|HEM2_HUMAN	HUMAN	Delta-aminolevulinic acid dehydratase OS=Homo sapiens OX=9606 GN=ALAD PE=1 SV=1
sp|P62750|RL23A_HUMAN	HUMAN	60S ribosomal protein L23a OS=Homo sapiens OX=9606 GN=RPL23A PE=1 SV=1
sp|Q14839|CHD4_HUMAN	HUMAN	Chromodomain-helicase-DNA-binding protein 4 OS=Homo sapiens OX=9606 GN=CHD4 PE=1 SV=2
sp|Q8NCW5|NNRE_HUMAN	HUMAN	NAD(P)H-hydrate epimerase OS=Homo sapiens OX=9606 GN=NAXE PE=1 SV=2
sp|P14314|GLU2B_HUMAN	HUMAN	Glucosidase 2 subunit beta OS=Homo sapiens OX=9606 GN=PRKCSH PE=1 SV=2
sp|P68036|UB2L3_HUMAN	HUMAN	Ubiquitin-conjugating enzyme E2 L3 OS=Homo sapiens OX=9606 GN=UBE2L3 PE=1 SV=1
sp|Q53H82|LACB2_HUMAN	HUMAN	Endoribonuclease LACTB2 OS=Homo sapiens OX=9606 GN=LACTB2 PE=1 SV=2
sp|P15586|GNS_HUMAN	HUMAN	N-acetylglucosamine-6-sulfatase OS=Homo sapiens OX=9606 GN=GNS PE=1 SV=3
sp|Q9Y376|CAB39_HUMAN	HUMAN	Calcium-binding protein 39 OS=Homo sapiens OX=9606 GN=CAB39 PE=1 SV=1
sp|Q13283|G3BP1_HUMAN	HUMAN	Ras GTPase-activating protein-binding protein 1 OS=Homo sapiens OX=9606 GN=G3BP1 PE=1 SV=1
sp|Q9ULZ3|ASC_HUMAN	HUMAN	Apoptosis-associated speck-like protein containing a CARD OS=Homo sapiens OX=9606 GN=PYCARD PE=1 SV=2
sp|P62820|RAB1A_HUMAN	HUMAN	Ras-related protein Rab-1A OS=Homo sapiens OX=9606 GN=RAB1A PE=1 SV=3
sp|O95861|BPNT1_HUMAN	HUMAN	3'(2'),5'-bisphosphate nucleotidase 1 OS=Homo sapiens OX=9606 GN=BPNT1 PE=1 SV=1
sp|P18754|RCC1_HUMAN	HUMAN	Regulator of chromosome condensation OS=Homo sapiens OX=9606 GN=RCC1 PE=1 SV=1
sp|Q92890|UFD1_HUMAN	HUMAN	Ubiquitin recognition factor in ER-associated degradation protein 1 OS=Homo sapiens OX=9606 GN=UFD1 PE=1 SV=3
sp|O00425|IF2B3_HUMAN	HUMAN	Insulin-like growth factor 2 mRNA-binding protein 3 OS=Homo sapiens OX=9606 GN=IGF2BP3 PE=1 SV=2
sp|P67936|TPM4_HUMAN	HUMAN	Tropomyosin alpha-4 chain OS=Homo sapiens OX=9606 GN=TPM4 PE=1 SV=3
sp|Q9Y5X3|SNX5_HUMAN	HUMAN	Sorting nexin-5 OS=Homo sapiens OX=9606 GN=SNX5 PE=1 SV=1
sp|Q9BPX3|CND3_HUMAN	HUMAN	Condensin complex subunit 3 OS=Homo sapiens OX=9606 GN=NCAPG PE=1 SV=1
sp|Q15637|SF01_HUMAN	HUMAN	Splicing factor 1 OS=Homo sapiens OX=9606 GN=SF1 PE=1 SV=4
sp|O15160|RPAC1_HUMAN	HUMAN	DNA-directed RNA polymerases I and III subunit RPAC1 OS=Homo sapiens OX=9606 GN=POLR1C PE=1 SV=1
sp|Q96C86|DCPS_HUMAN	HUMAN	m7GpppX diphosphatase OS=Homo sapiens OX=9606 GN=DCPS PE=1 SV=2
sp|P51665|PSMD7_HUMAN	HUMAN	26S proteasome non-ATPase regulatory subunit 7 OS=Homo sapiens OX=9606 GN=PSMD7 PE=1 SV=2
sp|P62987|RL40_HUMAN	HUMAN	Ubiquitin-60S ribosomal protein L40 OS=Homo sapiens OX=9606 GN=UBA52 PE=1 SV=2
sp|Q15185|TEBP_HUMAN	HUMAN	Prostaglandin E synthase 3 OS=Homo sapiens OX=9606 GN=PTGES3 PE=1 SV=1

sp|P23368|MAOM_HUMAN	HUMAN	NAD-dependent malic enzyme, mitochondrial OS=Homo sapiens OX=9606 GN=ME2 PE=1 SV=1
sp|Q92990|GLMN_HUMAN	HUMAN	Glomulin OS=Homo sapiens OX=9606 GN=GLMN PE=1 SV=2
sp|Q92619|HMHA1_HUMAN	HUMAN	Rho GTPase-activating protein 45 OS=Homo sapiens OX=9606 GN=ARHGAP45 PE=1 SV=2
sp|P68371|TBB4B_HUMAN	HUMAN	Tubulin beta-4B chain OS=Homo sapiens OX=9606 GN=TUBB4B PE=1 SV=1
sp|Q9H9T3|ELP3_HUMAN	HUMAN	Elongator complex protein 3 OS=Homo sapiens OX=9606 GN=ELP3 PE=1 SV=2
sp|P26196|DDX6_HUMAN	HUMAN	Probable ATP-dependent RNA helicase DDX6 OS=Homo sapiens OX=9606 GN=DDX6 PE=1 SV=2
sp|P41227|NAA10_HUMAN	HUMAN	N-alpha-acetyltransferase 10 OS=Homo sapiens OX=9606 GN=NAA10 PE=1 SV=1
sp|Q9NXG2|THUM1_HUMAN	HUMAN	THUMP domain-containing protein 1 OS=Homo sapiens OX=9606 GN=THUMPD1 PE=1 SV=2
sp|Q8TAT6|NPL4_HUMAN	HUMAN	Nuclear protein localization protein 4 homolog OS=Homo sapiens OX=9606 GN=NPLOC4 PE=1 SV=3
sp|O43765|SGTA_HUMAN	HUMAN	Small glutamine-rich tetratricopeptide repeat-containing protein alpha OS=Homo sapiens OX=9606 GN=SGTA PE=1 SV=1
sp|P61254|RL26_HUMAN	HUMAN	60S ribosomal protein L26 OS=Homo sapiens OX=9606 GN=RPL26 PE=1 SV=1
sp|P62899|RL31_HUMAN	HUMAN	60S ribosomal protein L31 OS=Homo sapiens OX=9606 GN=RPL31 PE=1 SV=1
sp|P62847|RS24_HUMAN	HUMAN	40S ribosomal protein S24 OS=Homo sapiens OX=9606 GN=RPS24 PE=1 SV=1
sp|Q9UJA5|TRM6_HUMAN	HUMAN	tRNA (adenine(58)-N(1))-methyltransferase non-catalytic subunit TRM6 OS=Homo sapiens OX=9606 GN=TRMT6 PE=1 SV=1
sp|P18077|RL35A_HUMAN	HUMAN	60S ribosomal protein L35a OS=Homo sapiens OX=9606 GN=RPL35A PE=1 SV=2
sp|O75348|VATG1_HUMAN	HUMAN	V-type proton ATPase subunit G 1 OS=Homo sapiens OX=9606 GN=ATP6V1G1 PE=1 SV=3
sp|Q9BUL8|PDC10_HUMAN	HUMAN	Programmed cell death protein 10 OS=Homo sapiens OX=9606 GN=PDCD10 PE=1 SV=1
sp|Q9UNH7|SNX6_HUMAN	HUMAN	Sorting nexin-6 OS=Homo sapiens OX=9606 GN=SNX6 PE=1 SV=1
sp|P30085|KCY_HUMAN	HUMAN	UMP-CMP kinase OS=Homo sapiens OX=9606 GN=CMPK1 PE=1 SV=3
sp|P08311|CATG_HUMAN	HUMAN	Cathepsin G OS=Homo sapiens OX=9606 GN=CTSG PE=1 SV=2
sp|Q13813|SPTN1_HUMAN	HUMAN	Spectrin alpha chain, non-erythrocytic 1 OS=Homo sapiens OX=9606 GN=SPTAN1 PE=1 SV=3
sp|P23588|IF4B_HUMAN	HUMAN	Eukaryotic translation initiation factor 4B OS=Homo sapiens OX=9606 GN=EIF4B PE=1 SV=2
sp|Q99733|NP1L4_HUMAN	HUMAN	Nucleosome assembly protein 1-like 4 OS=Homo sapiens OX=9606 GN=NAP1L4 PE=1 SV=1
sp|Q06187|BTK_HUMAN	HUMAN	Tyrosine-protein kinase BTK OS=Homo sapiens OX=9606 GN=BTK PE=1 SV=3
sp|Q9Y285|SYFA_HUMAN	HUMAN	Phenylalanine--tRNA ligase alpha subunit OS=Homo sapiens OX=9606 GN=FARSA PE=1 SV=3
sp|P49593|PPM1F_HUMAN	HUMAN	Protein phosphatase 1F OS=Homo sapiens OX=9606 GN=PPM1F PE=1 SV=3
sp|Q13617|CUL2_HUMAN	HUMAN	Cullin-2 OS=Homo sapiens OX=9606 GN=CUL2 PE=1 SV=2
sp|O60610|DIAP1_HUMAN	HUMAN	Protein diaphanous homolog 1 OS=Homo sapiens OX=9606 GN=DIAPH1 PE=1 SV=2
sp|P26447|S10A4_HUMAN	HUMAN	Protein S100-A4 OS=Homo sapiens OX=9606 GN=S100A4 PE=1 SV=1
sp|Q07020|RL18_HUMAN	HUMAN	60S ribosomal protein L18 OS=Homo sapiens OX=9606 GN=RPL18 PE=1 SV=2

sp|Q9Y606|TRUA_HUMAN	HUMAN	tRNA pseudouridine synthase A OS=Homo sapiens OX=9606 GN=PUS1 PE=1 SV=3
sp|Q13765|NACA_HUMAN	HUMAN	Nascent polypeptide-associated complex subunit alpha OS=Homo sapiens OX=9606 GN=NACA PE=1 SV=1
sp|O00754|MA2B1_HUMAN	HUMAN	Lysosomal alpha-mannosidase OS=Homo sapiens OX=9606 GN=MAN2B1 PE=1 SV=3
sp|Q92597|NDRG1_HUMAN	HUMAN	Protein NDRG1 OS=Homo sapiens OX=9606 GN=NDRG1 PE=1 SV=1
sp|Q9UKF6|CPSF3_HUMAN	HUMAN	Cleavage and polyadenylation specificity factor subunit 3 OS=Homo sapiens OX=9606 GN=CPSF3 PE=1 SV=1
sp|P60866|RS20_HUMAN	HUMAN	40S ribosomal protein S20 OS=Homo sapiens OX=9606 GN=RPS20 PE=1 SV=1
sp|Q07955|SRSF1_HUMAN	HUMAN	Serine/arginine-rich splicing factor 1 OS=Homo sapiens OX=9606 GN=SRSF1 PE=1 SV=2
sp|O43252|PAPS1_HUMAN	HUMAN	Bifunctional 3'-phosphoadenosine 5'-phosphosulfate synthase 1 OS=Homo sapiens OX=9606 GN=PAPSS1 PE=1 SV=2
sp|Q13442|HAP28_HUMAN	HUMAN	28 kDa heat- and acid-stable phosphoprotein OS=Homo sapiens OX=9606 GN=PDAP1 PE=1 SV=1
sp|P28074|PSB5_HUMAN	HUMAN	Proteasome subunit beta type-5 OS=Homo sapiens OX=9606 GN=PSMB5 PE=1 SV=3
sp|P49902|5NTC_HUMAN	HUMAN	Cytosolic purine 5'-nucleotidase OS=Homo sapiens OX=9606 GN=NT5C2 PE=1 SV=1
sp|Q9Y6A5|TACC3_HUMAN	HUMAN	Transforming acidic coiled-coil-containing protein 3 OS=Homo sapiens OX=9606 GN=TACC3 PE=1 SV=1
sp|P20073|ANXA7_HUMAN	HUMAN	Annexin A7 OS=Homo sapiens OX=9606 GN=ANXA7 PE=1 SV=3
sp|P54687|BCAT1_HUMAN	HUMAN	Branched-chain-amino-acid aminotransferase, cytosolic OS=Homo sapiens OX=9606 GN=BCAT1 PE=1 SV=3
sp|Q96Q11|TRNT1_HUMAN	HUMAN	CCA tRNA nucleotidyltransferase 1, mitochondrial OS=Homo sapiens OX=9606 GN=TRNT1 PE=1 SV=2
sp|Q15019|SEPT2_HUMAN	HUMAN	Septin-2 OS=Homo sapiens OX=9606 GN=SEPTIN2 PE=1 SV=1
sp|Q13185|CBX3_HUMAN	HUMAN	Chromobox protein homolog 3 OS=Homo sapiens OX=9606 GN=CBX3 PE=1 SV=4
sp|Q16666|IF16_HUMAN	HUMAN	Gamma-interferon-inducible protein 16 OS=Homo sapiens OX=9606 GN=IFI16 PE=1 SV=3
sp|Q9NQ48|LZTL1_HUMAN	HUMAN	Leucine zipper transcription factor-like protein 1 OS=Homo sapiens OX=9606 GN=LZTFL1 PE=1 SV=1
sp|P52306|GDS1_HUMAN	HUMAN	Rap1 GTPase-GDP dissociation stimulator 1 OS=Homo sapiens OX=9606 GN=RAP1GDS1 PE=1 SV=3
sp|Q9UKD2|MRT4_HUMAN	HUMAN	mRNA turnover protein 4 homolog OS=Homo sapiens OX=9606 GN=MRTO4 PE=1 SV=2
sp|Q8IYS1|P20D2_HUMAN	HUMAN	Peptidase M20 domain-containing protein 2 OS=Homo sapiens OX=9606 GN=PM20D2 PE=1 SV=2
sp|Q9Y2L1|RRP44_HUMAN	HUMAN	Exosome complex exonuclease RRP44 OS=Homo sapiens OX=9606 GN=DIS3 PE=1 SV=2
sp|O14737|PDCD5_HUMAN	HUMAN	Programmed cell death protein 5 OS=Homo sapiens OX=9606 GN=PDCD5 PE=1 SV=3
sp|Q5TFE4|NT5D1_HUMAN	HUMAN	5'-nucleotidase domain-containing protein 1 OS=Homo sapiens OX=9606 GN=NT5DC1 PE=1 SV=1
sp|P35908|K22E_HUMAN	HUMAN	Keratin, type II cytoskeletal 2 epidermal OS=Homo sapiens OX=9606 GN=KRT2 PE=1 SV=2
sp|Q9UHX1|PUF60_HUMAN	HUMAN	Poly(U)-binding-splicing factor PUF60 OS=Homo sapiens OX=9606 GN=PUF60 PE=1 SV=1
sp|Q06124|PTN11_HUMAN	HUMAN	Tyrosine-protein phosphatase non-receptor type 11 OS=Homo sapiens OX=9606 GN=PTPN11 PE=1 SV=3
sp|Q9UKY7|CDV3_HUMAN	HUMAN	Protein CDV3 homolog OS=Homo sapiens OX=9606 GN=CDV3 PE=1 SV=1
sp|Q8NEZ5|FBX22_HUMAN	HUMAN	F-box only protein 22 OS=Homo sapiens OX=9606 GN=FBXO22 PE=1 SV=1

sp|Q8WXX5|DNJC9_HUMAN	HUMAN	DnaJ homolog subfamily C member 9 OS=Homo sapiens OX=9606 GN=DNAJC9 PE=1 SV=1
sp|Q13418|ILK_HUMAN	HUMAN	Integrin-linked protein kinase OS=Homo sapiens OX=9606 GN=ILK PE=1 SV=2
sp|P68366|TBA4A_HUMAN	HUMAN	Tubulin alpha-4A chain OS=Homo sapiens OX=9606 GN=TUBA4A PE=1 SV=1
sp|O95373|IPO7_HUMAN	HUMAN	Importin-7 OS=Homo sapiens OX=9606 GN=IPO7 PE=1 SV=1
sp|P27635|RL10_HUMAN	HUMAN	60S ribosomal protein L10 OS=Homo sapiens OX=9606 GN=RPL10 PE=1 SV=4
sp|Q9UHY7|ENOPH_HUMAN	HUMAN	Enolase-phosphatase E1 OS=Homo sapiens OX=9606 GN=ENOPH1 PE=1 SV=1
sp|Q92769|HDAC2_HUMAN	HUMAN	Histone deacetylase 2 OS=Homo sapiens OX=9606 GN=HDAC2 PE=1 SV=2
sp|Q9GZT8|NIF3L_HUMAN	HUMAN	NIF3-like protein 1 OS=Homo sapiens OX=9606 GN=NIF3L1 PE=1 SV=2
sp|Q01130|SRSF2_HUMAN	HUMAN	Serine/arginine-rich splicing factor 2 OS=Homo sapiens OX=9606 GN=SRSF2 PE=1 SV=4
sp|Q96I15|SCLY_HUMAN	HUMAN	Selenocysteine lyase OS=Homo sapiens OX=9606 GN=SCLY PE=1 SV=4
sp|Q14CX7|NAA25_HUMAN	HUMAN	N-alpha-acetyltransferase 25, NatB auxiliary subunit OS=Homo sapiens OX=9606 GN=NAA25 PE=1 SV=1
sp|P84103|SRSF3_HUMAN	HUMAN	Serine/arginine-rich splicing factor 3 OS=Homo sapiens OX=9606 GN=SRSF3 PE=1 SV=1
sp|P62942|FKB1A_HUMAN	HUMAN	Peptidyl-prolyl cis-trans isomerase FKBP1A OS=Homo sapiens OX=9606 GN=FKBP1A PE=1 SV=2
sp|P50914|RL14_HUMAN	HUMAN	60S ribosomal protein L14 OS=Homo sapiens OX=9606 GN=RPL14 PE=1 SV=4
sp|P46782|RS5_HUMAN	HUMAN	40S ribosomal protein S5 OS=Homo sapiens OX=9606 GN=RPS5 PE=1 SV=4
sp|P38159|RBMX_HUMAN	HUMAN	RNA-binding motif protein, X chromosome OS=Homo sapiens OX=9606 GN=RBMX PE=1 SV=3
sp|P24666|PPAC_HUMAN	HUMAN	Low molecular weight phosphotyrosine protein phosphatase OS=Homo sapiens OX=9606 GN=ACP1 PE=1 SV=3
sp|P08243|ASNS_HUMAN	HUMAN	Asparagine synthetase [glutamine-hydrolyzing] OS=Homo sapiens OX=9606 GN=ASNS PE=1 SV=4
sp|Q9UNS2|CSN3_HUMAN	HUMAN	COP9 signalosome complex subunit 3 OS=Homo sapiens OX=9606 GN=COPS3 PE=1 SV=3
sp|Q9Y6G9|DC1L1_HUMAN	HUMAN	Cytoplasmic dynein 1 light intermediate chain 1 OS=Homo sapiens OX=9606 GN=DYNC1LI1 PE=1 SV=3
sp|P63279|UBC9_HUMAN	HUMAN	SUMO-conjugating enzyme UBC9 OS=Homo sapiens OX=9606 GN=UBE2I PE=1 SV=1
sp|P78330|SERB_HUMAN	HUMAN	Phosphoserine phosphatase OS=Homo sapiens OX=9606 GN=PSPH PE=1 SV=2
sp|P84098|RL19_HUMAN	HUMAN	60S ribosomal protein L19 OS=Homo sapiens OX=9606 GN=RPL19 PE=1 SV=1
sp|P28482|MK01_HUMAN	HUMAN	Mitogen-activated protein kinase 1 OS=Homo sapiens OX=9606 GN=MAPK1 PE=1 SV=3
sp|P35637|FUS_HUMAN	HUMAN	RNA-binding protein FUS OS=Homo sapiens OX=9606 GN=FUS PE=1 SV=1
sp|P07384|CAN1_HUMAN	HUMAN	Calpain-1 catalytic subunit OS=Homo sapiens OX=9606 GN=CAPN1 PE=1 SV=1
sp|Q16644|MAPK3_HUMAN	HUMAN	MAP kinase-activated protein kinase 3 OS=Homo sapiens OX=9606 GN=MAPKAPK3 PE=1 SV=1
sp|O60701|UGDH_HUMAN	HUMAN	UDP-glucose 6-dehydrogenase OS=Homo sapiens OX=9606 GN=UGDH PE=1 SV=1
sp|P42285|MTREX_HUMAN	HUMAN	Exosome RNA helicase MTR4 OS=Homo sapiens OX=9606 GN=MTREX PE=1 SV=3
sp|O43324|MCA3_HUMAN	HUMAN	Eukaryotic translation elongation factor 1 epsilon-1 OS=Homo sapiens OX=9606 GN=EEF1E1 PE=1 SV=1

sp|P43246|MSH2_HUMAN	HUMAN	DNA mismatch repair protein Msh2 OS=Homo sapiens OX=9606 GN=MSH2 PE=1 SV=1
sp|Q8TDP1|RNH2C_HUMAN	HUMAN	Ribonuclease H2 subunit C OS=Homo sapiens OX=9606 GN=RNASEH2C PE=1 SV=1
sp|P28066|PSA5_HUMAN	HUMAN	Proteasome subunit alpha type-5 OS=Homo sapiens OX=9606 GN=PSMA5 PE=1 SV=3
sp|P09651|ROA1_HUMAN	HUMAN	Heterogeneous nuclear ribonucleoprotein A1 OS=Homo sapiens OX=9606 GN=HNRNPA1 PE=1 SV=5
sp|O15145|ARPC3_HUMAN	HUMAN	Actin-related protein 2/3 complex subunit 3 OS=Homo sapiens OX=9606 GN=ARPC3 PE=1 SV=3
sp|Q6FI81|CPIN1_HUMAN	HUMAN	Anamorsin OS=Homo sapiens OX=9606 GN=CIAPIN1 PE=1 SV=2
sp|P62314|SMD1_HUMAN	HUMAN	Small nuclear ribonucleoprotein Sm D1 OS=Homo sapiens OX=9606 GN=SNRPD1 PE=1 SV=1
sp|P08708|RS17_HUMAN	HUMAN	40S ribosomal protein S17 OS=Homo sapiens OX=9606 GN=RPS17 PE=1 SV=2
sp|P09525|ANXA4_HUMAN	HUMAN	Annexin A4 OS=Homo sapiens OX=9606 GN=ANXA4 PE=1 SV=4
sp|Q13057|COASY_HUMAN	HUMAN	Bifunctional coenzyme A synthase OS=Homo sapiens OX=9606 GN=COASY PE=1 SV=4
sp|P52597|HNRPF_HUMAN	HUMAN	Heterogeneous nuclear ribonucleoprotein F OS=Homo sapiens OX=9606 GN=HNRNPF PE=1 SV=3
sp|P46063|RECQ1_HUMAN	HUMAN	ATP-dependent DNA helicase Q1 OS=Homo sapiens OX=9606 GN=RECQL PE=1 SV=3
sp|Q99471|PFD5_HUMAN	HUMAN	Prefoldin subunit 5 OS=Homo sapiens OX=9606 GN=PFDN5 PE=1 SV=2
sp|Q16186|ADRM1_HUMAN	HUMAN	Proteasomal ubiquitin receptor ADRM1 OS=Homo sapiens OX=9606 GN=ADRM1 PE=1 SV=2
sp|P04080|CYTB_HUMAN	HUMAN	Cystatin-B OS=Homo sapiens OX=9606 GN=CSTB PE=1 SV=2
sp|Q9NTM9|CUTC_HUMAN	HUMAN	Copper homeostasis protein cutC homolog OS=Homo sapiens OX=9606 GN=CUTC PE=1 SV=1
sp|P04632|CPNS1_HUMAN	HUMAN	Calpain small subunit 1 OS=Homo sapiens OX=9606 GN=CAPNS1 PE=1 SV=1
sp|O94804|STK10_HUMAN	HUMAN	Serine/threonine-protein kinase 10 OS=Homo sapiens OX=9606 GN=STK10 PE=1 SV=1
sp|P26583|HMGB2_HUMAN	HUMAN	High mobility group protein B2 OS=Homo sapiens OX=9606 GN=HMGB2 PE=1 SV=2
sp|Q9UBQ5|EIF3K_HUMAN	HUMAN	Eukaryotic translation initiation factor 3 subunit K OS=Homo sapiens OX=9606 GN=EIF3K PE=1 SV=1
sp|Q13162|PRDX4_HUMAN	HUMAN	Peroxiredoxin-4 OS=Homo sapiens OX=9606 GN=PRDX4 PE=1 SV=1
sp|Q9Y3U8|RL36_HUMAN	HUMAN	60S ribosomal protein L36 OS=Homo sapiens OX=9606 GN=RPL36 PE=1 SV=3
sp|Q96B97|SH3K1_HUMAN	HUMAN	SH3 domain-containing kinase-binding protein 1 OS=Homo sapiens OX=9606 GN=SH3KBP1 PE=1 SV=2
sp|Q13043|STK4_HUMAN	HUMAN	Serine/threonine-protein kinase 4 OS=Homo sapiens OX=9606 GN=STK4 PE=1 SV=2
sp|Q8NBJ7|SUMF2_HUMAN	HUMAN	Inactive C-alpha-formylglycine-generating enzyme 2 OS=Homo sapiens OX=9606 GN=SUMF2 PE=1 SV=2
sp|P08559|ODPA_HUMAN	HUMAN	Pyruvate dehydrogenase E1 component subunit alpha, somatic form, mitochondrial OS=Homo sapiens OX=9606 GN=PDHA1 PE=1 SV=3
sp|P61081|UBC12_HUMAN	HUMAN	NEDD8-conjugating enzyme Ubc12 OS=Homo sapiens OX=9606 GN=UBE2M PE=1 SV=1
sp|P50452|SPB8_HUMAN	HUMAN	Serpin B8 OS=Homo sapiens OX=9606 GN=SERPINB8 PE=1 SV=2
sp|P62633|CNBP_HUMAN	HUMAN	Cellular nucleic acid-binding protein OS=Homo sapiens OX=9606 GN=CNBP PE=1 SV=1
sp|Q07021|C1QBP_HUMAN	HUMAN	Complement component 1 Q subcomponent-binding protein, mitochondrial OS=Homo sapiens OX=9606 GN=C1QBP PE=1 SV=1

sp|Q15257|PTPA_HUMAN	HUMAN	Serine/threonine-protein phosphatase 2A activator OS=Homo sapiens OX=9606 GN=PTPA PE=1 SV=3
sp|Q6P2E9|EDC4_HUMAN	HUMAN	Enhancer of mRNA-decapping protein 4 OS=Homo sapiens OX=9606 GN=EDC4 PE=1 SV=1
sp|O15372|EIF3H_HUMAN	HUMAN	Eukaryotic translation initiation factor 3 subunit H OS=Homo sapiens OX=9606 GN=EIF3H PE=1 SV=1
sp|O00584|RNT2_HUMAN	HUMAN	Ribonuclease T2 OS=Homo sapiens OX=9606 GN=RNASET2 PE=1 SV=2
sp|P55263|ADK_HUMAN	HUMAN	Adenosine kinase OS=Homo sapiens OX=9606 GN=ADK PE=1 SV=2
sp|P49773|HINT1_HUMAN	HUMAN	Histidine triad nucleotide-binding protein 1 OS=Homo sapiens OX=9606 GN=HINT1 PE=1 SV=2
sp|P06396|GELS_HUMAN	HUMAN	Gelsolin OS=Homo sapiens OX=9606 GN=GSN PE=1 SV=1
sp|Q16822|PCKGM_HUMAN	HUMAN	Phosphoenolpyruvate carboxykinase [GTP], mitochondrial OS=Homo sapiens OX=9606 GN=PCK2 PE=1 SV=4
sp|Q14019|COTL1_HUMAN	HUMAN	Coactosin-like protein OS=Homo sapiens OX=9606 GN=COTL1 PE=1 SV=3
sp|Q9C0C9|UBE2O_HUMAN	HUMAN	(E3-independent) E2 ubiquitin-conjugating enzyme OS=Homo sapiens OX=9606 GN=UBE2O PE=1 SV=3
sp|O00629|IMA3_HUMAN	HUMAN	Importin subunit alpha-3 OS=Homo sapiens OX=9606 GN=KPNA4 PE=1 SV=1
sp|Q6PKG0|LARP1_HUMAN	HUMAN	La-related protein 1 OS=Homo sapiens OX=9606 GN=LARP1 PE=1 SV=2
sp|Q7Z7A4|PXK_HUMAN	HUMAN	PX domain-containing protein kinase-like protein OS=Homo sapiens OX=9606 GN=PXK PE=1 SV=1
sp|Q15121|PEA15_HUMAN	HUMAN	Astrocytic phosphoprotein PEA-15 OS=Homo sapiens OX=9606 GN=PEA15 PE=1 SV=2
sp|O00160|MYO1F_HUMAN	HUMAN	Unconventional myosin-If OS=Homo sapiens OX=9606 GN=MYO1F PE=1 SV=3
sp|P13807|GYS1_HUMAN	HUMAN	Glycogen [starch] synthase, muscle OS=Homo sapiens OX=9606 GN=GYS1 PE=1 SV=2
sp|Q9UBQ0|VPS29_HUMAN	HUMAN	Vacuolar protein sorting-associated protein 29 OS=Homo sapiens OX=9606 GN=VPS29 PE=1 SV=1
sp|O75821|EIF3G_HUMAN	HUMAN	Eukaryotic translation initiation factor 3 subunit G OS=Homo sapiens OX=9606 GN=EIF3G PE=1 SV=2
sp|Q9Y2Z0|SGT1_HUMAN	HUMAN	Protein SGT1 homolog OS=Homo sapiens OX=9606 GN=SUGT1 PE=1 SV=3
sp|Q7L2H7|EIF3M_HUMAN	HUMAN	Eukaryotic translation initiation factor 3 subunit M OS=Homo sapiens OX=9606 GN=EIF3M PE=1 SV=1
sp|Q9BZQ8|NIBA1_HUMAN	HUMAN	Protein Niban 1 OS=Homo sapiens OX=9606 GN=NIBAN1 PE=1 SV=1
sp|P40429|RL13A_HUMAN	HUMAN	60S ribosomal protein L13a OS=Homo sapiens OX=9606 GN=RPL13A PE=1 SV=2
sp|Q9ULA0|DNPEP_HUMAN	HUMAN	Aspartyl aminopeptidase OS=Homo sapiens OX=9606 GN=DNPEP PE=1 SV=1
sp|P62280|RS11_HUMAN	HUMAN	40S ribosomal protein S11 OS=Homo sapiens OX=9606 GN=RPS11 PE=1 SV=3
sp|O60271|JIP4_HUMAN	HUMAN	C-Jun-amino-terminal kinase-interacting protein 4 OS=Homo sapiens OX=9606 GN=SPAG9 PE=1 SV=4
sp|O00178|GTPB1_HUMAN	HUMAN	GTP-binding protein 1 OS=Homo sapiens OX=9606 GN=GTPBP1 PE=1 SV=3
sp|Q9H3U1|UN45A_HUMAN	HUMAN	Protein unc-45 homolog A OS=Homo sapiens OX=9606 GN=UNC45A PE=1 SV=1
sp|O75436|VP26A_HUMAN	HUMAN	Vacuolar protein sorting-associated protein 26A OS=Homo sapiens OX=9606 GN=VPS26A PE=1 SV=2
sp|Q9BQ52|RNZ2_HUMAN	HUMAN	Zinc phosphodiesterase ELAC protein 2 OS=Homo sapiens OX=9606 GN=ELAC2 PE=1 SV=2
sp|P27986|P85A_HUMAN	HUMAN	Phosphatidylinositol 3-kinase regulatory subunit alpha OS=Homo sapiens OX=9606 GN=PIK3R1 PE=1 SV=2
sp|Q15907|RB11B_HUMAN	HUMAN	Ras-related protein Rab-11B OS=Homo sapiens OX=9606 GN=RAB11B PE=1 SV=4

sp|Q09028|RBBP4_HUMAN	HUMAN	Histone-binding protein RBBP4 OS=Homo sapiens OX=9606 GN=RBBP4 PE=1 SV=3
sp|Q92841|DDX17_HUMAN	HUMAN	Probable ATP-dependent RNA helicase DDX17 OS=Homo sapiens OX=9606 GN=DDX17 PE=1 SV=2
sp|Q96RU3|FNBP1_HUMAN	HUMAN	Formin-binding protein 1 OS=Homo sapiens OX=9606 GN=FNBP1 PE=1 SV=2
sp|Q9BQE3|TBA1C_HUMAN	HUMAN	Tubulin alpha-1C chain OS=Homo sapiens OX=9606 GN=TUBA1C PE=1 SV=1
sp|P38117|ETFB_HUMAN	HUMAN	Electron transfer flavoprotein subunit beta OS=Homo sapiens OX=9606 GN=ETFB PE=1 SV=3
sp|Q13685|AAMP_HUMAN	HUMAN	Angio-associated migratory cell protein OS=Homo sapiens OX=9606 GN=AAMP PE=1 SV=2
sp|P36551|HEM6_HUMAN	HUMAN	Oxygen-dependent coproporphyrinogen-III oxidase, mitochondrial OS=Homo sapiens OX=9606 GN=CPOX PE=1 SV=3
sp|P42166|LAP2A_HUMAN	HUMAN	Lamina-associated polypeptide 2, isoform alpha OS=Homo sapiens OX=9606 GN=TMPO PE=1 SV=2
sp|Q13907|IDI1_HUMAN	HUMAN	Isopentenyl-diphosphate Delta-isomerase 1 OS=Homo sapiens OX=9606 GN=IDI1 PE=1 SV=2
sp|O60711|LPXN_HUMAN	HUMAN	Leupaxin OS=Homo sapiens OX=9606 GN=LPXN PE=1 SV=1
sp|Q07866|KLC1_HUMAN	HUMAN	Kinesin light chain 1 OS=Homo sapiens OX=9606 GN=KLC1 PE=1 SV=2
sp|Q5JVF3|PCID2_HUMAN	HUMAN	PCI domain-containing protein 2 OS=Homo sapiens OX=9606 GN=PCID2 PE=1 SV=2
sp|O43598|DNPH1_HUMAN	HUMAN	2'-deoxynucleoside 5'-phosphate N-hydrolase 1 OS=Homo sapiens OX=9606 GN=DNPH1 PE=1 SV=1
sp|P21291|CSRP1_HUMAN	HUMAN	Cysteine and glycine-rich protein 1 OS=Homo sapiens OX=9606 GN=CSRP1 PE=1 SV=3
sp|Q9NYU2|UGGG1_HUMAN	HUMAN	UDP-glucose:glycoprotein glucosyltransferase 1 OS=Homo sapiens OX=9606 GN=UGGT1 PE=1 SV=3
sp|P67870|CSK2B_HUMAN	HUMAN	Casein kinase II subunit beta OS=Homo sapiens OX=9606 GN=CSNK2B PE=1 SV=1
sp|Q14738|2A5D_HUMAN	HUMAN	Serine/threonine-protein phosphatase 2A 56 kDa regulatory subunit delta isoform OS=Homo sapiens OX=9606 GN=PPP2R5D PE=1 SV=1
sp|P47813|IF1AX_HUMAN	HUMAN	Eukaryotic translation initiation factor 1A, X-chromosomal OS=Homo sapiens OX=9606 GN=EIF1AX PE=1 SV=2
sp|P51452|DUS3_HUMAN	HUMAN	Dual specificity protein phosphatase 3 OS=Homo sapiens OX=9606 GN=DUSP3 PE=1 SV=1
sp|Q14444|CAPR1_HUMAN	HUMAN	Caprin-1 OS=Homo sapiens OX=9606 GN=CAPRIN1 PE=1 SV=2
sp|Q96CW1|AP2M1_HUMAN	HUMAN	AP-2 complex subunit mu OS=Homo sapiens OX=9606 GN=AP2M1 PE=1 SV=2
sp|P46926|GNPI1_HUMAN	HUMAN	Glucosamine-6-phosphate isomerase 1 OS=Homo sapiens OX=9606 GN=GNPDA1 PE=1 SV=1
sp|P18858|DNLI1_HUMAN	HUMAN	DNA ligase 1 OS=Homo sapiens OX=9606 GN=LIG1 PE=1 SV=1
sp|P40939|ECHA_HUMAN	HUMAN	Trifunctional enzyme subunit alpha, mitochondrial OS=Homo sapiens OX=9606 GN=HADHA PE=1 SV=2
sp|Q08752|PPID_HUMAN	HUMAN	Peptidyl-prolyl cis-trans isomerase D OS=Homo sapiens OX=9606 GN=PPID PE=1 SV=3
sp|Q99729|ROAA_HUMAN	HUMAN	Heterogeneous nuclear ribonucleoprotein A/B OS=Homo sapiens OX=9606 GN=HNRNPAB PE=1 SV=2
sp|P84077|ARF1_HUMAN	HUMAN	ADP-ribosylation factor 1 OS=Homo sapiens OX=9606 GN=ARF1 PE=1 SV=2
sp|P30419|NMT1_HUMAN	HUMAN	Glycylpeptide N-tetradecanoyltransferase 1 OS=Homo sapiens OX=9606 GN=NMT1 PE=1 SV=2
sp|P62917|RL8_HUMAN	HUMAN	60S ribosomal protein L8 OS=Homo sapiens OX=9606 GN=RPL8 PE=1 SV=2

sp|P26368|U2AF2_HUMAN	HUMAN	Splicing factor U2AF 65 kDa subunit OS=Homo sapiens OX=9606 GN=U2AF2 PE=1 SV=4
sp|O43684|BUB3_HUMAN	HUMAN	Mitotic checkpoint protein BUB3 OS=Homo sapiens OX=9606 GN=BUB3 PE=1 SV=1
sp|Q9GZS3|WDR61_HUMAN	HUMAN	WD repeat-containing protein 61 OS=Homo sapiens OX=9606 GN=WDR61 PE=1 SV=1
sp|Q96M27|PRRC1_HUMAN	HUMAN	Protein PRRC1 OS=Homo sapiens OX=9606 GN=PRRC1 PE=1 SV=1
sp|Q9HAV4|XPO5_HUMAN	HUMAN	Exportin-5 OS=Homo sapiens OX=9606 GN=XPO5 PE=1 SV=1
sp|Q5TDH0|DDI2_HUMAN	HUMAN	Protein DDI1 homolog 2 OS=Homo sapiens OX=9606 GN=DDI2 PE=1 SV=1
sp|O75340|PDCD6_HUMAN	HUMAN	Programmed cell death protein 6 OS=Homo sapiens OX=9606 GN=PDCD6 PE=1 SV=1
sp|Q7L5N1|CSN6_HUMAN	HUMAN	COP9 signalosome complex subunit 6 OS=Homo sapiens OX=9606 GN=COPS6 PE=1 SV=1
sp|O60502|OGA_HUMAN	HUMAN	Protein O-GlcNAcase OS=Homo sapiens OX=9606 GN=OGA PE=1 SV=2
sp|P62841|RS15_HUMAN	HUMAN	40S ribosomal protein S15 OS=Homo sapiens OX=9606 GN=RPS15 PE=1 SV=2
sp|P08621|RU17_HUMAN	HUMAN	U1 small nuclear ribonucleoprotein 70 kDa OS=Homo sapiens OX=9606 GN=SNRNP70 PE=1 SV=2
sp|P09622|DLDH_HUMAN	HUMAN	Dihydrolipoyl dehydrogenase, mitochondrial OS=Homo sapiens OX=9606 GN=DLD PE=1 SV=2
sp|Q9BZZ5|API5_HUMAN	HUMAN	Apoptosis inhibitor 5 OS=Homo sapiens OX=9606 GN=API5 PE=1 SV=3
sp|P04183|KITH_HUMAN	HUMAN	Thymidine kinase, cytosolic OS=Homo sapiens OX=9606 GN=TK1 PE=1 SV=2
sp|Q9P2T1|GMPR2_HUMAN	HUMAN	GMP reductase 2 OS=Homo sapiens OX=9606 GN=GMPR2 PE=1 SV=1
sp|Q96I24|FUBP3_HUMAN	HUMAN	Far upstream element-binding protein 3 OS=Homo sapiens OX=9606 GN=FUBP3 PE=1 SV=2
sp|Q07960|RHG01_HUMAN	HUMAN	Rho GTPase-activating protein 1 OS=Homo sapiens OX=9606 GN=ARHGAP1 PE=1 SV=1
sp|Q9P287|BCCIP_HUMAN	HUMAN	BRCA2 and CDKN1A-interacting protein OS=Homo sapiens OX=9606 GN=BCCIP PE=1 SV=1
sp|Q92882|OSTF1_HUMAN	HUMAN	Osteoclast-stimulating factor 1 OS=Homo sapiens OX=9606 GN=OSTF1 PE=1 SV=2
sp|Q9UBB4|ATX10_HUMAN	HUMAN	Ataxin-10 OS=Homo sapiens OX=9606 GN=ATXN10 PE=1 SV=1
sp|Q8IVD9|NUDC3_HUMAN	HUMAN	NudC domain-containing protein 3 OS=Homo sapiens OX=9606 GN=NUDCD3 PE=1 SV=3
sp|Q96P70|IPO9_HUMAN	HUMAN	Importin-9 OS=Homo sapiens OX=9606 GN=IPO9 PE=1 SV=3
sp|O75995|SASH3_HUMAN	HUMAN	SAM and SH3 domain-containing protein 3 OS=Homo sapiens OX=9606 GN=SASH3 PE=1 SV=2
sp|P28065|PSB9_HUMAN	HUMAN	Proteasome subunit beta type-9 OS=Homo sapiens OX=9606 GN=PSMB9 PE=1 SV=2
sp|P23921|RIR1_HUMAN	HUMAN	Ribonucleoside-diphosphate reductase large subunit OS=Homo sapiens OX=9606 GN=RRM1 PE=1 SV=1
sp|P19784|CSK22_HUMAN	HUMAN	Casein kinase II subunit alpha' OS=Homo sapiens OX=9606 GN=CSNK2A2 PE=1 SV=1
sp|O75531|BAF_HUMAN	HUMAN	Barrier-to-autointegration factor OS=Homo sapiens OX=9606 GN=BANF1 PE=1 SV=1
sp|Q9BYT8|NEUL_HUMAN	HUMAN	Neurolysin, mitochondrial OS=Homo sapiens OX=9606 GN=NLN PE=1 SV=1
sp|Q13619|CUL4A_HUMAN	HUMAN	Cullin-4A OS=Homo sapiens OX=9606 GN=CUL4A PE=1 SV=3
sp|Q15056|IF4H_HUMAN	HUMAN	Eukaryotic translation initiation factor 4H OS=Homo sapiens OX=9606 GN=EIF4H PE=1 SV=5
sp|Q9NXR7|BABA2_HUMAN	HUMAN	BRISC and BRCA1-A complex member 2 OS=Homo sapiens OX=9606 GN=BABAM2 PE=1 SV=2

sp|Q9BRG1|VPS25_HUMAN	HUMAN	Vacuolar protein-sorting-associated protein 25 OS=Homo sapiens OX=9606 GN=VPS25 PE=1 SV=1
sp|Q99447|PCY2_HUMAN	HUMAN	Ethanolamine-phosphate cytidylyltransferase OS=Homo sapiens OX=9606 GN=PCYT2 PE=1 SV=1
sp|Q96FW1|OTUB1_HUMAN	HUMAN	Ubiquitin thioesterase OTUB1 OS=Homo sapiens OX=9606 GN=OTUB1 PE=1 SV=2
sp|Q15018|ABRX2_HUMAN	HUMAN	BRISC complex subunit Abraxas 2 OS=Homo sapiens OX=9606 GN=ABRAXAS2 PE=1 SV=2
sp|P62316|SMD2_HUMAN	HUMAN	Small nuclear ribonucleoprotein Sm D2 OS=Homo sapiens OX=9606 GN=SNRPD2 PE=1 SV=1
sp|P55084|ECHB_HUMAN	HUMAN	Trifunctional enzyme subunit beta, mitochondrial OS=Homo sapiens OX=9606 GN=HADHB PE=1 SV=3
sp|P04179|SODM_HUMAN	HUMAN	Superoxide dismutase [Mn], mitochondrial OS=Homo sapiens OX=9606 GN=SOD2 PE=1 SV=3
sp|Q14240|IF4A2_HUMAN	HUMAN	Eukaryotic initiation factor 4A-II OS=Homo sapiens OX=9606 GN=EIF4A2 PE=1 SV=2
sp|P15153|RAC2_HUMAN	HUMAN	Ras-related C3 botulinum toxin substrate 2 OS=Homo sapiens OX=9606 GN=RAC2 PE=1 SV=1
sp|O94979|SC31A_HUMAN	HUMAN	Protein transport protein Sec31A OS=Homo sapiens OX=9606 GN=SEC31A PE=1 SV=3
sp|Q96EP5|DAZP1_HUMAN	HUMAN	DAZ-associated protein 1 OS=Homo sapiens OX=9606 GN=DAZAP1 PE=1 SV=1
sp|Q8WU90|ZC3HF_HUMAN	HUMAN	Zinc finger CCCH domain-containing protein 15 OS=Homo sapiens OX=9606 GN=ZC3H15 PE=1 SV=1
sp|P14678|RSMB_HUMAN	HUMAN	Small nuclear ribonucleoprotein-associated proteins B and B' OS=Homo sapiens OX=9606 GN=SNRPB PE=1 SV=2
sp|Q9UKX7|NUP50_HUMAN	HUMAN	Nuclear pore complex protein Nup50 OS=Homo sapiens OX=9606 GN=NUP50 PE=1 SV=2
sp|Q9NZT2|OGFR_HUMAN	HUMAN	Opioid growth factor receptor OS=Homo sapiens OX=9606 GN=OGFR PE=1 SV=3
sp|Q86V81|THOC4_HUMAN	HUMAN	THO complex subunit 4 OS=Homo sapiens OX=9606 GN=ALYREF PE=1 SV=3
sp|Q9UK76|JUPI1_HUMAN	HUMAN	Jupiter microtubule associated homolog 1 OS=Homo sapiens OX=9606 GN=JPT1 PE=1 SV=3
sp|Q9NX46|ARHL2_HUMAN	HUMAN	ADP-ribose glycohydrolase ARH3 OS=Homo sapiens OX=9606 GN=ADPRHL2 PE=1 SV=1
sp|Q9H0R4|HDHD2_HUMAN	HUMAN	Haloacid dehalogenase-like hydrolase domain-containing protein 2 OS=Homo sapiens OX=9606 GN=HDHD2 PE=1 SV=1
sp|Q9C0B1|FTO_HUMAN	HUMAN	Alpha-ketoglutarate-dependent dioxygenase FTO OS=Homo sapiens OX=9606 GN=FTO PE=1 SV=3
sp|Q96GK7|FAH2A_HUMAN	HUMAN	Fumarylacetoacetate hydrolase domain-containing protein 2A OS=Homo sapiens OX=9606 GN=FAHD2A PE=1 SV=1
sp|Q96FJ2|DYL2_HUMAN	HUMAN	Dynein light chain 2, cytoplasmic OS=Homo sapiens OX=9606 GN=DYNLL2 PE=1 SV=1
sp|Q13526|PIN1_HUMAN	HUMAN	Peptidyl-prolyl cis-trans isomerase NIMA-interacting 1 OS=Homo sapiens OX=9606 GN=PIN1 PE=1 SV=1
sp|P62318|SMD3_HUMAN	HUMAN	Small nuclear ribonucleoprotein Sm D3 OS=Homo sapiens OX=9606 GN=SNRPD3 PE=1 SV=1
sp|P62269|RS18_HUMAN	HUMAN	40S ribosomal protein S18 OS=Homo sapiens OX=9606 GN=RPS18 PE=1 SV=3
sp|P55957|BID_HUMAN	HUMAN	BH3-interacting domain death agonist OS=Homo sapiens OX=9606 GN=BID PE=1 SV=1
sp|P36543|VATE1_HUMAN	HUMAN	V-type proton ATPase subunit E 1 OS=Homo sapiens OX=9606 GN=ATP6V1E1 PE=1 SV=1
sp|P28072|PSB6_HUMAN	HUMAN	Proteasome subunit beta type-6 OS=Homo sapiens OX=9606 GN=PSMB6 PE=1 SV=4
sp|P08579|RU2B_HUMAN	HUMAN	U2 small nuclear ribonucleoprotein B'' OS=Homo sapiens OX=9606 GN=SNRPB2 PE=1 SV=1

sp|O75223|GGCT_HUMAN	HUMAN	Gamma-glutamylcyclotransferase OS=Homo sapiens OX=9606 GN=GGCT PE=1 SV=1
sp|O15382|BCAT2_HUMAN	HUMAN	Branched-chain-amino-acid aminotransferase, mitochondrial OS=Homo sapiens OX=9606 GN=BCAT2 PE=1 SV=2
sp|Q969T9|WBP2_HUMAN	HUMAN	WW domain-binding protein 2 OS=Homo sapiens OX=9606 GN=WBP2 PE=1 SV=1
sp|Q9H0C8|ILKAP_HUMAN	HUMAN	Integrin-linked kinase-associated serine/threonine phosphatase 2C OS=Homo sapiens OX=9606 GN=ILKAP PE=1 SV=1
sp|P09496|CLCA_HUMAN	HUMAN	Clathrin light chain A OS=Homo sapiens OX=9606 GN=CLTA PE=1 SV=1
sp|P51692|STA5B_HUMAN	HUMAN	Signal transducer and activator of transcription 5B OS=Homo sapiens OX=9606 GN=STAT5B PE=1 SV=2
sp|Q9NP79|VTA1_HUMAN	HUMAN	Vacuolar protein sorting-associated protein VTA1 homolog OS=Homo sapiens OX=9606 GN=VTA1 PE=1 SV=1
sp|P19105|ML12A_HUMAN	HUMAN	Myosin regulatory light chain 12A OS=Homo sapiens OX=9606 GN=MYL12A PE=1 SV=2
sp|Q7L1Q6|BZW1_HUMAN	HUMAN	Basic leucine zipper and W2 domain-containing protein 1 OS=Homo sapiens OX=9606 GN=BZW1 PE=1 SV=1
sp|Q9H2U2|IPYR2_HUMAN	HUMAN	Inorganic pyrophosphatase 2, mitochondrial OS=Homo sapiens OX=9606 GN=PPA2 PE=1 SV=2
sp|P11310|ACADM_HUMAN	HUMAN	Medium-chain specific acyl-CoA dehydrogenase, mitochondrial OS=Homo sapiens OX=9606 GN=ACADM PE=1 SV=1
sp|Q99627|CSN8_HUMAN	HUMAN	COP9 signalosome complex subunit 8 OS=Homo sapiens OX=9606 GN=COPS8 PE=1 SV=1
sp|P62910|RL32_HUMAN	HUMAN	60S ribosomal protein L32 OS=Homo sapiens OX=9606 GN=RPL32 PE=1 SV=2
sp|P02533|K1C14_HUMAN	HUMAN	Keratin, type I cytoskeletal 14 OS=Homo sapiens OX=9606 GN=KRT14 PE=1 SV=4
sp|P23919|KTHY_HUMAN	HUMAN	Thymidylate kinase OS=Homo sapiens OX=9606 GN=DTYMK PE=1 SV=4
sp|P06865|HEXA_HUMAN	HUMAN	Beta-hexosaminidase subunit alpha OS=Homo sapiens OX=9606 GN=HEXA PE=1 SV=2
sp|P46109|CRKL_HUMAN	HUMAN	Crk-like protein OS=Homo sapiens OX=9606 GN=CRKL PE=1 SV=1
sp|Q5TBB1|RNH2B_HUMAN	HUMAN	Ribonuclease H2 subunit B OS=Homo sapiens OX=9606 GN=RNASEH2B PE=1 SV=1
sp|P52789|HXK2_HUMAN	HUMAN	Hexokinase-2 OS=Homo sapiens OX=9606 GN=HK2 PE=1 SV=2
sp|Q9UN86|G3BP2_HUMAN	HUMAN	Ras GTPase-activating protein-binding protein 2 OS=Homo sapiens OX=9606 GN=G3BP2 PE=1 SV=2
sp|Q9H299|SH3L3_HUMAN	HUMAN	SH3 domain-binding glutamic acid-rich-like protein 3 OS=Homo sapiens OX=9606 GN=SH3BGRL3 PE=1 SV=1
sp|O60234|GMFG_HUMAN	HUMAN	Glia maturation factor gamma OS=Homo sapiens OX=9606 GN=GMFG PE=1 SV=1
sp|Q13310|PABP4_HUMAN	HUMAN	Polyadenylate-binding protein 4 OS=Homo sapiens OX=9606 GN=PABPC4 PE=1 SV=1
sp|Q03154|ACY1_HUMAN	HUMAN	Aminoacylase-1 OS=Homo sapiens OX=9606 GN=ACY1 PE=1 SV=1
sp|Q96HE7|ERO1A_HUMAN	HUMAN	ERO1-like protein alpha OS=Homo sapiens OX=9606 GN=ERO1A PE=1 SV=2
sp|O60925|PFD1_HUMAN	HUMAN	Prefoldin subunit 1 OS=Homo sapiens OX=9606 GN=PFDN1 PE=1 SV=2
sp|P62854|RS26_HUMAN	HUMAN	40S ribosomal protein S26 OS=Homo sapiens OX=9606 GN=RPS26 PE=1 SV=3
sp|Q15819|UB2V2_HUMAN	HUMAN	Ubiquitin-conjugating enzyme E2 variant 2 OS=Homo sapiens OX=9606 GN=UBE2V2 PE=1 SV=4

sp|P42768|WASP_HUMAN	HUMAN	Wiskott-Aldrich syndrome protein OS=Homo sapiens OX=9606 GN=WAS PE=1 SV=4
sp|Q14157|UBP2L_HUMAN	HUMAN	Ubiquitin-associated protein 2-like OS=Homo sapiens OX=9606 GN=UBAP2L PE=1 SV=2
sp|P42126|ECI1_HUMAN	HUMAN	Enoyl-CoA delta isomerase 1, mitochondrial OS=Homo sapiens OX=9606 GN=ECI1 PE=1 SV=1
sp|Q9UHV9|PFD2_HUMAN	HUMAN	Prefoldin subunit 2 OS=Homo sapiens OX=9606 GN=PFDN2 PE=1 SV=1
sp|P99999|CYC_HUMAN	HUMAN	Cytochrome c OS=Homo sapiens OX=9606 GN=CYCS PE=1 SV=2
sp|O60684|IMA7_HUMAN	HUMAN	Importin subunit alpha-7 OS=Homo sapiens OX=9606 GN=KPNA6 PE=1 SV=1
sp|Q12931|TRAP1_HUMAN	HUMAN	Heat shock protein 75 kDa, mitochondrial OS=Homo sapiens OX=9606 GN=TRAP1 PE=1 SV=3
sp|Q6NYC1|JMJD6_HUMAN	HUMAN	Bifunctional arginine demethylase and lysyl-hydroxylase JMJD6 OS=Homo sapiens OX=9606 GN=JMJD6 PE=1 SV=1
sp|P61513|RL37A_HUMAN	HUMAN	60S ribosomal protein L37a OS=Homo sapiens OX=9606 GN=RPL37A PE=1 SV=2
sp|P51148|RAB5C_HUMAN	HUMAN	Ras-related protein Rab-5C OS=Homo sapiens OX=9606 GN=RAB5C PE=1 SV=2
sp|Q16629|SRSF7_HUMAN	HUMAN	Serine/arginine-rich splicing factor 7 OS=Homo sapiens OX=9606 GN=SRSF7 PE=1 SV=1
sp|O00233|PSMD9_HUMAN	HUMAN	26S proteasome non-ATPase regulatory subunit 9 OS=Homo sapiens OX=9606 GN=PSMD9 PE=1 SV=3
sp|Q15080|NCF4_HUMAN	HUMAN	Neutrophil cytosol factor 4 OS=Homo sapiens OX=9606 GN=NCF4 PE=1 SV=2
sp|P35659|DEK_HUMAN	HUMAN	Protein DEK OS=Homo sapiens OX=9606 GN=DEK PE=1 SV=1
sp|Q13510|ASAH1_HUMAN	HUMAN	Acid ceramidase OS=Homo sapiens OX=9606 GN=ASAH1 PE=1 SV=5
sp|Q0VDF9|HSP7E_HUMAN	HUMAN	Heat shock 70 kDa protein 14 OS=Homo sapiens OX=9606 GN=HSPA14 PE=1 SV=1
sp|Q969H8|MYDGF_HUMAN	HUMAN	Myeloid-derived growth factor OS=Homo sapiens OX=9606 GN=MYDGF PE=1 SV=1
sp|Q16204|CCDC6_HUMAN	HUMAN	Coiled-coil domain-containing protein 6 OS=Homo sapiens OX=9606 GN=CCDC6 PE=1 SV=2
sp|O95671|ASML_HUMAN	HUMAN	Probable bifunctional dTTP/UTP pyrophosphatase/methyltransferase protein OS=Homo sapiens OX=9606 GN=ASMTL PE=1 SV=3
sp|Q14C86|GAPD1_HUMAN	HUMAN	GTPase-activating protein and VPS9 domain-containing protein 1 OS=Homo sapiens OX=9606 GN=GAPVD1 PE=1 SV=2
sp|Q13409|DC1I2_HUMAN	HUMAN	Cytoplasmic dynein 1 intermediate chain 2 OS=Homo sapiens OX=9606 GN=DYNC1I2 PE=1 SV=3
sp|P30084|ECHM_HUMAN	HUMAN	Enoyl-CoA hydratase, mitochondrial OS=Homo sapiens OX=9606 GN=ECHS1 PE=1 SV=4
sp|Q86VN1|VPS36_HUMAN	HUMAN	Vacuolar protein-sorting-associated protein 36 OS=Homo sapiens OX=9606 GN=VPS36 PE=1 SV=1
sp|P43243|MATR3_HUMAN	HUMAN	Matrin-3 OS=Homo sapiens OX=9606 GN=MATR3 PE=1 SV=2
sp|Q13616|CUL1_HUMAN	HUMAN	Cullin-1 OS=Homo sapiens OX=9606 GN=CUL1 PE=1 SV=2
sp|P46776|RL27A_HUMAN	HUMAN	60S ribosomal protein L27a OS=Homo sapiens OX=9606 GN=RPL27A PE=1 SV=2
sp|P34949|MPI_HUMAN	HUMAN	Mannose-6-phosphate isomerase OS=Homo sapiens OX=9606 GN=MPI PE=1 SV=2
sp|O43768|ENSA_HUMAN	HUMAN	Alpha-endosulfine OS=Homo sapiens OX=9606 GN=ENSA PE=1 SV=1
sp|P09417|DHPR_HUMAN	HUMAN	Dihydropteridine reductase OS=Homo sapiens OX=9606 GN=QDPR PE=1 SV=2

sp|Q96IU4|ABHEB_HUMAN	HUMAN	Protein ABHD14B OS=Homo sapiens OX=9606 GN=ABHD14B PE=1 SV=1
sp|Q29RF7|PDS5A_HUMAN	HUMAN	Sister chromatid cohesion protein PDS5 homolog A OS=Homo sapiens OX=9606 GN=PDS5A PE=1 SV=1
sp|O00170|AIP_HUMAN	HUMAN	AH receptor-interacting protein OS=Homo sapiens OX=9606 GN=AIP PE=1 SV=2
sp|O75569|PRKRA_HUMAN	HUMAN	Interferon-inducible double-stranded RNA-dependent protein kinase activator A OS=Homo sapiens OX=9606 GN=PRKRA PE=1 SV=1
sp|Q9Y2B0|CNPY2_HUMAN	HUMAN	Protein canopy homolog 2 OS=Homo sapiens OX=9606 GN=CNPY2 PE=1 SV=1
sp|P62310|LSM3_HUMAN	HUMAN	U6 snRNA-associated Sm-like protein LSm3 OS=Homo sapiens OX=9606 GN=LSM3 PE=1 SV=2
sp|O14579|COPE_HUMAN	HUMAN	Coatomer subunit epsilon OS=Homo sapiens OX=9606 GN=COPE PE=1 SV=3
sp|P51659|DHB4_HUMAN	HUMAN	Peroxisomal multifunctional enzyme type 2 OS=Homo sapiens OX=9606 GN=HSD17B4 PE=1 SV=3
sp|P49642|PRI1_HUMAN	HUMAN	DNA primase small subunit OS=Homo sapiens OX=9606 GN=PRIM1 PE=1 SV=1
sp|Q96A72|MGN2_HUMAN	HUMAN	Protein mago nashi homolog 2 OS=Homo sapiens OX=9606 GN=MAGOHB PE=1 SV=1
sp|Q15404|RSU1_HUMAN	HUMAN	Ras suppressor protein 1 OS=Homo sapiens OX=9606 GN=RSU1 PE=1 SV=3
sp|Q8WWY3|PRP31_HUMAN	HUMAN	U4/U6 small nuclear ribonucleoprotein Prp31 OS=Homo sapiens OX=9606 GN=PRPF31 PE=1 SV=2
sp|Q9H3K6|BOLA2_HUMAN	HUMAN	BolA-like protein 2 OS=Homo sapiens OX=9606 GN=BOLA2 PE=1 SV=1
sp|P62873|GBB1_HUMAN	HUMAN	Guanine nucleotide-binding protein G(I)/G(S)/G(T) subunit beta-1 OS=Homo sapiens OX=9606 GN=GNB1 PE=1 SV=3
sp|Q52LJ0|FA98B_HUMAN	HUMAN	Protein FAM98B OS=Homo sapiens OX=9606 GN=FAM98B PE=1 SV=2
sp|Q9UHD8|SEPT9_HUMAN	HUMAN	Septin-9 OS=Homo sapiens OX=9606 GN=SEPTIN9 PE=1 SV=2
sp|O75351|VPS4B_HUMAN	HUMAN	Vacuolar protein sorting-associated protein 4B OS=Homo sapiens OX=9606 GN=VPS4B PE=1 SV=2
sp|P48556|PSMD8_HUMAN	HUMAN	26S proteasome non-ATPase regulatory subunit 8 OS=Homo sapiens OX=9606 GN=PSMD8 PE=1 SV=2
sp|P25325|THTM_HUMAN	HUMAN	3-mercaptopyruvate sulfurtransferase OS=Homo sapiens OX=9606 GN=MPST PE=1 SV=3
sp|P22059|OSBP1_HUMAN	HUMAN	Oxysterol-binding protein 1 OS=Homo sapiens OX=9606 GN=OSBP PE=1 SV=1
sp|P11387|TOP1_HUMAN	HUMAN	DNA topoisomerase 1 OS=Homo sapiens OX=9606 GN=TOP1 PE=1 SV=2
sp|Q6JBY9|CPZIP_HUMAN	HUMAN	CapZ-interacting protein OS=Homo sapiens OX=9606 GN=RCSD1 PE=1 SV=1
sp|Q08209|PP2BA_HUMAN	HUMAN	Serine/threonine-protein phosphatase 2B catalytic subunit alpha isoform OS=Homo sapiens OX=9606 GN=PPP3CA PE=1 SV=1
sp|Q13618|CUL3_HUMAN	HUMAN	Cullin-3 OS=Homo sapiens OX=9606 GN=CUL3 PE=1 SV=2
sp|Q9NZZ3|CHMP5_HUMAN	HUMAN	Charged multivesicular body protein 5 OS=Homo sapiens OX=9606 GN=CHMP5 PE=1 SV=1
sp|P05771|KPCB_HUMAN	HUMAN	Protein kinase C beta type OS=Homo sapiens OX=9606 GN=PRKCB PE=1 SV=4
sp|Q15418|KS6A1_HUMAN	HUMAN	Ribosomal protein S6 kinase alpha-1 OS=Homo sapiens OX=9606 GN=RPS6KA1 PE=1 SV=2
sp|Q9H074|PAIP1_HUMAN	HUMAN	Polyadenylate-binding protein-interacting protein 1 OS=Homo sapiens OX=9606 GN=PAIP1 PE=1 SV=1
sp|P06730|IF4E_HUMAN	HUMAN	Eukaryotic translation initiation factor 4E OS=Homo sapiens OX=9606 GN=EIF4E PE=1 SV=2

sp|O14974|MYPT1_HUMAN	HUMAN	Protein phosphatase 1 regulatory subunit 12A OS=Homo sapiens OX=9606 GN=PPP1R12A PE=1 SV=1
sp|O60749|SNX2_HUMAN	HUMAN	Sorting nexin-2 OS=Homo sapiens OX=9606 GN=SNX2 PE=1 SV=2
sp|Q9P1F3|ABRAL_HUMAN	HUMAN	Costars family protein ABRACL OS=Homo sapiens OX=9606 GN=ABRACL PE=1 SV=1
sp|Q01415|GALK2_HUMAN	HUMAN	N-acetylgalactosamine kinase OS=Homo sapiens OX=9606 GN=GALK2 PE=1 SV=1
sp|Q96CN7|ISOC1_HUMAN	HUMAN	Isochorismatase domain-containing protein 1 OS=Homo sapiens OX=9606 GN=ISOC1 PE=1 SV=3
sp|P46779|RL28_HUMAN	HUMAN	60S ribosomal protein L28 OS=Homo sapiens OX=9606 GN=RPL28 PE=1 SV=3
sp|Q14155|ARHG7_HUMAN	HUMAN	Rho guanine nucleotide exchange factor 7 OS=Homo sapiens OX=9606 GN=ARHGEF7 PE=1 SV=2
sp|Q9UI30|TR112_HUMAN	HUMAN	Multifunctional methyltransferase subunit TRM112-like protein OS=Homo sapiens OX=9606 GN=TRMT112 PE=1 SV=1
sp|Q9H4E7|DEFI6_HUMAN	HUMAN	Differentially expressed in FDCP 6 homolog OS=Homo sapiens OX=9606 GN=DEF6 PE=1 SV=1
sp|P12270|TPR_HUMAN	HUMAN	Nucleoprotein TPR OS=Homo sapiens OX=9606 GN=TPR PE=1 SV=3
sp|Q9NX55|HYPK_HUMAN	HUMAN	Huntingtin-interacting protein K OS=Homo sapiens OX=9606 GN=HYPK PE=1 SV=2
sp|Q9BY43|CHM4A_HUMAN	HUMAN	Charged multivesicular body protein 4a OS=Homo sapiens OX=9606 GN=CHMP4A PE=1 SV=3
sp|Q8N335|GPD1L_HUMAN	HUMAN	Glycerol-3-phosphate dehydrogenase 1-like protein OS=Homo sapiens OX=9606 GN=GPD1L PE=1 SV=1
sp|O95834|EMAL2_HUMAN	HUMAN	Echinoderm microtubule-associated protein-like 2 OS=Homo sapiens OX=9606 GN=EML2 PE=1 SV=1
sp|P55160|NCKPL_HUMAN	HUMAN	Nck-associated protein 1-like OS=Homo sapiens OX=9606 GN=NCKAP1L PE=1 SV=3
sp|P51610|HCFC1_HUMAN	HUMAN	Host cell factor 1 OS=Homo sapiens OX=9606 GN=HCFC1 PE=1 SV=2
sp|P11388|TOP2A_HUMAN	HUMAN	DNA topoisomerase 2-alpha OS=Homo sapiens OX=9606 GN=TOP2A PE=1 SV=3
sp|Q86U42|PABP2_HUMAN	HUMAN	Polyadenylate-binding protein 2 OS=Homo sapiens OX=9606 GN=PABPN1 PE=1 SV=3
sp|P41567|EIF1_HUMAN	HUMAN	Eukaryotic translation initiation factor 1 OS=Homo sapiens OX=9606 GN=EIF1 PE=1 SV=1
sp|A6NDG6|PGP_HUMAN	HUMAN	Glycerol-3-phosphate phosphatase OS=Homo sapiens OX=9606 GN=PGP PE=1 SV=1
sp|P02794|FRIH_HUMAN	HUMAN	Ferritin heavy chain OS=Homo sapiens OX=9606 GN=FTH1 PE=1 SV=2
sp|Q92734|TFG_HUMAN	HUMAN	Protein TFG OS=Homo sapiens OX=9606 GN=TFG PE=1 SV=2
sp|Q96BS2|CHP3_HUMAN	HUMAN	Calcineurin B homologous protein 3 OS=Homo sapiens OX=9606 GN=TESC PE=1 SV=3
sp|Q9BRT8|CBWD1_HUMAN	HUMAN	COBW domain-containing protein 1 OS=Homo sapiens OX=9606 GN=CBWD1 PE=2 SV=1
sp|P50897|PPT1_HUMAN	HUMAN	Palmitoyl-protein thioesterase 1 OS=Homo sapiens OX=9606 GN=PPT1 PE=1 SV=1
sp|P48739|PIPNB_HUMAN	HUMAN	Phosphatidylinositol transfer protein beta isoform OS=Homo sapiens OX=9606 GN=PITPNB PE=1 SV=2
sp|Q9NRN7|ADPPT_HUMAN	HUMAN	L-aminoadipate-semialdehyde dehydrogenase-phosphopantetheinyl transferase OS=Homo sapiens OX=9606 GN=AASDHPPT PE=1 SV=2
sp|P27824|CALX_HUMAN	HUMAN	Calnexin OS=Homo sapiens OX=9606 GN=CANX PE=1 SV=2
sp|Q15645|PCH2_HUMAN	HUMAN	Pachytene checkpoint protein 2 homolog OS=Homo sapiens OX=9606 GN=TRIP13 PE=1 SV=2
sp|Q9BVG4|PBDC1_HUMAN	HUMAN	Protein PBDC1 OS=Homo sapiens OX=9606 GN=PBDC1 PE=1 SV=1

sp|O60341|KDM1A_HUMAN	HUMAN	Lysine-specific histone demethylase 1A OS=Homo sapiens OX=9606 GN=KDM1A PE=1 SV=2
sp|Q99661|KIF2C_HUMAN	HUMAN	Kinesin-like protein KIF2C OS=Homo sapiens OX=9606 GN=KIF2C PE=1 SV=2
sp|P04818|TYSY_HUMAN	HUMAN	Thymidylate synthase OS=Homo sapiens OX=9606 GN=TYMS PE=1 SV=3
sp|Q96RS6|NUDC1_HUMAN	HUMAN	NudC domain-containing protein 1 OS=Homo sapiens OX=9606 GN=NUDCD1 PE=1 SV=2
sp|Q9HCN4|GPN1_HUMAN	HUMAN	GPN-loop GTPase 1 OS=Homo sapiens OX=9606 GN=GPN1 PE=1 SV=1
sp|Q92538|GBF1_HUMAN	HUMAN	Golgi-specific brefeldin A-resistance guanine nucleotide exchange factor 1 OS=Homo sapiens OX=9606 GN=GBF1 PE=1 SV=2
sp|P26373|RL13_HUMAN	HUMAN	60S ribosomal protein L13 OS=Homo sapiens OX=9606 GN=RPL13 PE=1 SV=4
sp|Q8WXF1|PSPC1_HUMAN	HUMAN	Paraspeckle component 1 OS=Homo sapiens OX=9606 GN=PSPC1 PE=1 SV=1
sp|Q9HAV7|GRPE1_HUMAN	HUMAN	GrpE protein homolog 1, mitochondrial OS=Homo sapiens OX=9606 GN=GRPEL1 PE=1 SV=2
sp|P11172|UMPS_HUMAN	HUMAN	Uridine 5'-monophosphate synthase OS=Homo sapiens OX=9606 GN=UMPS PE=1 SV=1
sp|Q93008|USP9X_HUMAN	HUMAN	Probable ubiquitin carboxyl-terminal hydrolase FAF-X OS=Homo sapiens OX=9606 GN=USP9X PE=1 SV=3
sp|Q96GG9|DCNL1_HUMAN	HUMAN	DCN1-like protein 1 OS=Homo sapiens OX=9606 GN=DCUN1D1 PE=1 SV=1
sp|Q9UHB9|SRP68_HUMAN	HUMAN	Signal recognition particle subunit SRP68 OS=Homo sapiens OX=9606 GN=SRP68 PE=1 SV=2
sp|Q12972|PP1R8_HUMAN	HUMAN	Nuclear inhibitor of protein phosphatase 1 OS=Homo sapiens OX=9606 GN=PPP1R8 PE=1 SV=2
sp|Q96PZ0|PUS7_HUMAN	HUMAN	Pseudouridylate synthase 7 homolog OS=Homo sapiens OX=9606 GN=PUS7 PE=1 SV=2
sp|O00625|PIR_HUMAN	HUMAN	Pirin OS=Homo sapiens OX=9606 GN=PIR PE=1 SV=1
sp|P30837|AL1B1_HUMAN	HUMAN	Aldehyde dehydrogenase X, mitochondrial OS=Homo sapiens OX=9606 GN=ALDH1B1 PE=1 SV=3
sp|O94776|MTA2_HUMAN	HUMAN	Metastasis-associated protein MTA2 OS=Homo sapiens OX=9606 GN=MTA2 PE=1 SV=1
sp|P25705|ATPA_HUMAN	HUMAN	ATP synthase subunit alpha, mitochondrial OS=Homo sapiens OX=9606 GN=ATP5F1A PE=1 SV=1
sp|O95163|ELP1_HUMAN	HUMAN	Elongator complex protein 1 OS=Homo sapiens OX=9606 GN=ELP1 PE=1 SV=3
sp|P78318|IGBP1_HUMAN	HUMAN	Immunoglobulin-binding protein 1 OS=Homo sapiens OX=9606 GN=IGBP1 PE=1 SV=1
sp|P61956|SUMO2_HUMAN	HUMAN	Small ubiquitin-related modifier 2 OS=Homo sapiens OX=9606 GN=SUMO2 PE=1 SV=3
sp|Q13247|SRSF6_HUMAN	HUMAN	Serine/arginine-rich splicing factor 6 OS=Homo sapiens OX=9606 GN=SRSF6 PE=1 SV=2
sp|P17931|LEG3_HUMAN	HUMAN	Galectin-3 OS=Homo sapiens OX=9606 GN=LGALS3 PE=1 SV=5
sp|P60891|PRPS1_HUMAN	HUMAN	Ribose-phosphate pyrophosphokinase 1 OS=Homo sapiens OX=9606 GN=PRPS1 PE=1 SV=2
sp|Q9BQ67|GRWD1_HUMAN	HUMAN	Glutamate-rich WD repeat-containing protein 1 OS=Homo sapiens OX=9606 GN=GRWD1 PE=1 SV=1
sp|Q9BY44|EIF2A_HUMAN	HUMAN	Eukaryotic translation initiation factor 2A OS=Homo sapiens OX=9606 GN=EIF2A PE=1 SV=3
sp|Q9H6Z4|RANB3_HUMAN	HUMAN	Ran-binding protein 3 OS=Homo sapiens OX=9606 GN=RANBP3 PE=1 SV=1
sp|P51532|SMCA4_HUMAN	HUMAN	Transcription activator BRG1 OS=Homo sapiens OX=9606 GN=SMARCA4 PE=1 SV=2
sp|Q86X55|CARM1_HUMAN	HUMAN	Histone-arginine methyltransferase CARM1 OS=Homo sapiens OX=9606 GN=CARM1 PE=1 SV=3

sp|Q3KQV9|UAP1L_HUMAN	HUMAN	UDP-N-acetylhexosamine pyrophosphorylase-like protein 1 OS=Homo sapiens OX=9606 GN=UAP1L1 PE=1 SV=2
sp|Q9Y5P6|GMPPB_HUMAN	HUMAN	Mannose-1-phosphate guanyltransferase beta OS=Homo sapiens OX=9606 GN=GMPPB PE=1 SV=2
sp|Q9Y4K1|CRBG1_HUMAN	HUMAN	Beta/gamma crystallin domain-containing protein 1 OS=Homo sapiens OX=9606 GN=CRYBG1 PE=1 SV=3
sp|Q9Y315|DEOC_HUMAN	HUMAN	Deoxyribose-phosphate aldolase OS=Homo sapiens OX=9606 GN=DERA PE=1 SV=2
sp|P31942|HNRH3_HUMAN	HUMAN	Heterogeneous nuclear ribonucleoprotein H3 OS=Homo sapiens OX=9606 GN=HNRNPH3 PE=1 SV=2
sp|Q9NVE7|PANK4_HUMAN	HUMAN	4'-phosphopantetheine phosphatase OS=Homo sapiens OX=9606 GN=PANK4 PE=1 SV=1
sp|Q8TEQ6|GEMI5_HUMAN	HUMAN	Gem-associated protein 5 OS=Homo sapiens OX=9606 GN=GEMIN5 PE=1 SV=3
sp|P36405|ARL3_HUMAN	HUMAN	ADP-ribosylation factor-like protein 3 OS=Homo sapiens OX=9606 GN=ARL3 PE=1 SV=2
sp|Q9Y263|PLAP_HUMAN	HUMAN	Phospholipase A-2-activating protein OS=Homo sapiens OX=9606 GN=PLAA PE=1 SV=2
sp|Q9NVS9|PNPO_HUMAN	HUMAN	Pyridoxine-5'-phosphate oxidase OS=Homo sapiens OX=9606 GN=PNPO PE=1 SV=1
sp|Q71DI3|H32_HUMAN	HUMAN	Histone H3.2 OS=Homo sapiens OX=9606 GN=HIST2H3A PE=1 SV=3
sp|Q9UBP6|TRMB_HUMAN	HUMAN	tRNA (guanine-N(7)-)-methyltransferase OS=Homo sapiens OX=9606 GN=METTL1 PE=1 SV=1
sp|Q9BRA2|TXD17_HUMAN	HUMAN	Thioredoxin domain-containing protein 17 OS=Homo sapiens OX=9606 GN=TXNDC17 PE=1 SV=1
sp|Q4J6C6|PPCEL_HUMAN	HUMAN	Prolyl endopeptidase-like OS=Homo sapiens OX=9606 GN=PREPL PE=1 SV=1
sp|Q04323|UBXN1_HUMAN	HUMAN	UBX domain-containing protein 1 OS=Homo sapiens OX=9606 GN=UBXN1 PE=1 SV=2
sp|P61970|NTF2_HUMAN	HUMAN	Nuclear transport factor 2 OS=Homo sapiens OX=9606 GN=NUTF2 PE=1 SV=1
sp|O60869|EDF1_HUMAN	HUMAN	Endothelial differentiation-related factor 1 OS=Homo sapiens OX=9606 GN=EDF1 PE=1 SV=1
sp|P62136|PP1A_HUMAN	HUMAN	Serine/threonine-protein phosphatase PP1-alpha catalytic subunit OS=Homo sapiens OX=9606 GN=PPP1CA PE=1 SV=1
sp|O75044|SRGP2_HUMAN	HUMAN	SLIT-ROBO Rho GTPase-activating protein 2 OS=Homo sapiens OX=9606 GN=SRGAP2 PE=1 SV=3
sp|Q13573|SNW1_HUMAN	HUMAN	SNW domain-containing protein 1 OS=Homo sapiens OX=9606 GN=SNW1 PE=1 SV=1
sp|O95394|AGM1_HUMAN	HUMAN	Phosphoacetylglucosamine mutase OS=Homo sapiens OX=9606 GN=PGM3 PE=1 SV=1
sp|Q9Y5Z4|HEBP2_HUMAN	HUMAN	Heme-binding protein 2 OS=Homo sapiens OX=9606 GN=HEBP2 PE=1 SV=1
sp|Q9UFN0|NPS3A_HUMAN	HUMAN	Protein NipSnap homolog 3A OS=Homo sapiens OX=9606 GN=NIPSNAP3A PE=1 SV=2
sp|Q96CT7|CC124_HUMAN	HUMAN	Coiled-coil domain-containing protein 124 OS=Homo sapiens OX=9606 GN=CCDC124 PE=1 SV=1
sp|Q8WVJ2|NUDC2_HUMAN	HUMAN	NudC domain-containing protein 2 OS=Homo sapiens OX=9606 GN=NUDCD2 PE=1 SV=1
sp|Q15067|ACOX1_HUMAN	HUMAN	Peroxisomal acyl-coenzyme A oxidase 1 OS=Homo sapiens OX=9606 GN=ACOX1 PE=1 SV=3
sp|Q13568|IRF5_HUMAN	HUMAN	Interferon regulatory factor 5 OS=Homo sapiens OX=9606 GN=IRF5 PE=1 SV=2
sp|P42226|STAT6_HUMAN	HUMAN	Signal transducer and activator of transcription 6 OS=Homo sapiens OX=9606 GN=STAT6 PE=1 SV=1
sp|O43670|ZN207_HUMAN	HUMAN	BUB3-interacting and GLEBS motif-containing protein ZNF207 OS=Homo sapiens OX=9606 GN=ZNF207 PE=1 SV=1

sp|Q16778|H2B2E_HUMAN	HUMAN	Histone H2B type 2-E OS=Homo sapiens OX=9606 GN=HIST2H2BE PE=1 SV=3
sp|Q13547|HDAC1_HUMAN	HUMAN	Histone deacetylase 1 OS=Homo sapiens OX=9606 GN=HDAC1 PE=1 SV=1
sp|P48668|K2C6C_HUMAN	HUMAN	Keratin, type II cytoskeletal 6C OS=Homo sapiens OX=9606 GN=KRT6C PE=1 SV=3
sp|Q9BT73|PSMG3_HUMAN	HUMAN	Proteasome assembly chaperone 3 OS=Homo sapiens OX=9606 GN=PSMG3 PE=1 SV=1
sp|P14174|MIF_HUMAN	HUMAN	Macrophage migration inhibitory factor OS=Homo sapiens OX=9606 GN=MIF PE=1 SV=4
sp|Q9Y508|RN114_HUMAN	HUMAN	E3 ubiquitin-protein ligase RNF114 OS=Homo sapiens OX=9606 GN=RNF114 PE=1 SV=1
sp|Q9NUU7|DD19A_HUMAN	HUMAN	ATP-dependent RNA helicase DDX19A OS=Homo sapiens OX=9606 GN=DDX19A PE=1 SV=1
sp|Q9NVM9|INT13_HUMAN	HUMAN	Integrator complex subunit 13 OS=Homo sapiens OX=9606 GN=INTS13 PE=1 SV=2
sp|Q9NPF4|OSGEP_HUMAN	HUMAN	Probable tRNA N6-adenosine threonylcarbamoyltransferase OS=Homo sapiens OX=9606 GN=OSGEP PE=1 SV=1
sp|Q9GZU8|PIP30_HUMAN	HUMAN	PSME3-interacting protein OS=Homo sapiens OX=9606 GN=PSME3IP1 PE=1 SV=1
sp|Q9BZE9|ASPC1_HUMAN	HUMAN	Tether containing UBX domain for GLUT4 OS=Homo sapiens OX=9606 GN=ASPSCR1 PE=1 SV=1
sp|Q9BXP5|SRRT_HUMAN	HUMAN	Serrate RNA effector molecule homolog OS=Homo sapiens OX=9606 GN=SRRT PE=1 SV=1
sp|Q86UV5|UBP48_HUMAN	HUMAN	Ubiquitin carboxyl-terminal hydrolase 48 OS=Homo sapiens OX=9606 GN=USP48 PE=1 SV=1
sp|Q15276|RABE1_HUMAN	HUMAN	Rab GTPase-binding effector protein 1 OS=Homo sapiens OX=9606 GN=RABEP1 PE=1 SV=2
sp|Q15102|PA1B3_HUMAN	HUMAN	Platelet-activating factor acetylhydrolase IB subunit gamma OS=Homo sapiens OX=9606 GN=PAFAH1B3 PE=1 SV=1
sp|Q14320|FA50A_HUMAN	HUMAN	Protein FAM50A OS=Homo sapiens OX=9606 GN=FAM50A PE=1 SV=2
sp|P68402|PA1B2_HUMAN	HUMAN	Platelet-activating factor acetylhydrolase IB subunit beta OS=Homo sapiens OX=9606 GN=PAFAH1B2 PE=1 SV=1
sp|P63208|SKP1_HUMAN	HUMAN	S-phase kinase-associated protein 1 OS=Homo sapiens OX=9606 GN=SKP1 PE=1 SV=2
sp|P35244|RFA3_HUMAN	HUMAN	Replication protein A 14 kDa subunit OS=Homo sapiens OX=9606 GN=RPA3 PE=1 SV=1
sp|P15927|RFA2_HUMAN	HUMAN	Replication protein A 32 kDa subunit OS=Homo sapiens OX=9606 GN=RPA2 PE=1 SV=1
sp|O95232|LC7L3_HUMAN	HUMAN	Luc7-like protein 3 OS=Homo sapiens OX=9606 GN=LUC7L3 PE=1 SV=2
sp|O60216|RAD21_HUMAN	HUMAN	Double-strand-break repair protein rad21 homolog OS=Homo sapiens OX=9606 GN=RAD21 PE=1 SV=2
sp|O15347|HMGB3_HUMAN	HUMAN	High mobility group protein B3 OS=Homo sapiens OX=9606 GN=HMGB3 PE=1 SV=4
sp|Q9UBV8|PEF1_HUMAN	HUMAN	Peflin OS=Homo sapiens OX=9606 GN=PEF1 PE=1 SV=1
sp|Q9NQG5|RPR1B_HUMAN	HUMAN	Regulation of nuclear pre-mRNA domain-containing protein 1B OS=Homo sapiens OX=9606 GN=RPRD1B PE=1 SV=1
sp|Q9H993|ARMT1_HUMAN	HUMAN	Damage-control phosphatase ARMT1 OS=Homo sapiens OX=9606 GN=ARMT1 PE=1 SV=1
sp|Q9H098|F107B_HUMAN	HUMAN	Protein FAM107B OS=Homo sapiens OX=9606 GN=FAM107B PE=1 SV=1
sp|Q9GZT3|SLIRP_HUMAN	HUMAN	SRA stem-loop-interacting RNA-binding protein, mitochondrial OS=Homo sapiens OX=9606 GN=SLIRP PE=1 SV=1

sp|Q9BZK7|TBL1R_HUMAN	HUMAN	F-box-like/WD repeat-containing protein TBL1XR1 OS=Homo sapiens OX=9606 GN=TBL1XR1 PE=1 SV=1
sp|Q9BTD8|RBM42_HUMAN	HUMAN	RNA-binding protein 42 OS=Homo sapiens OX=9606 GN=RBM42 PE=1 SV=1
sp|Q9BQA1|MEP50_HUMAN	HUMAN	Methylosome protein 50 OS=Homo sapiens OX=9606 GN=WDR77 PE=1 SV=1
sp|Q99417|MYCBP_HUMAN	HUMAN	c-Myc-binding protein OS=Homo sapiens OX=9606 GN=MYCBP PE=1 SV=3
sp|Q96J01|THOC3_HUMAN	HUMAN	THO complex subunit 3 OS=Homo sapiens OX=9606 GN=THOC3 PE=1 SV=1
sp|Q96G28|CFA36_HUMAN	HUMAN	Cilia- and flagella-associated protein 36 OS=Homo sapiens OX=9606 GN=CFAP36 PE=1 SV=2
sp|Q92522|H1X_HUMAN	HUMAN	Histone H1x OS=Homo sapiens OX=9606 GN=H1FX PE=1 SV=1
sp|Q8WZA0|LZIC_HUMAN	HUMAN	Protein LZIC OS=Homo sapiens OX=9606 GN=LZIC PE=1 SV=1
sp|Q8WU79|SMAP2_HUMAN	HUMAN	Stromal membrane-associated protein 2 OS=Homo sapiens OX=9606 GN=SMAP2 PE=1 SV=1
sp|Q8NFH3|NUP43_HUMAN	HUMAN	Nucleoporin Nup43 OS=Homo sapiens OX=9606 GN=NUP43 PE=1 SV=1
sp|Q8IU85|KCC1D_HUMAN	HUMAN	Calcium/calmodulin-dependent protein kinase type 1D OS=Homo sapiens OX=9606 GN=CAMK1D PE=1 SV=1
sp|Q4VC31|CCD58_HUMAN	HUMAN	Coiled-coil domain-containing protein 58 OS=Homo sapiens OX=9606 GN=CCDC58 PE=1 SV=1
sp|Q13501|SQSTM_HUMAN	HUMAN	Sequestosome-1 OS=Homo sapiens OX=9606 GN=SQSTM1 PE=1 SV=1
sp|Q07812|BAX_HUMAN	HUMAN	Apoptosis regulator BAX OS=Homo sapiens OX=9606 GN=BAX PE=1 SV=1
sp|Q01844|EWS_HUMAN	HUMAN	RNA-binding protein EWS OS=Homo sapiens OX=9606 GN=EWSR1 PE=1 SV=1
sp|Q01085|TIAR_HUMAN	HUMAN	Nucleolysin TIAR OS=Homo sapiens OX=9606 GN=TIAL1 PE=1 SV=1
sp|P62837|UB2D2_HUMAN	HUMAN	Ubiquitin-conjugating enzyme E2 D2 OS=Homo sapiens OX=9606 GN=UBE2D2 PE=1 SV=1
sp|P49458|SRP09_HUMAN	HUMAN	Signal recognition particle 9 kDa protein OS=Homo sapiens OX=9606 GN=SRP9 PE=1 SV=2
sp|P48507|GSH0_HUMAN	HUMAN	Glutamate--cysteine ligase regulatory subunit OS=Homo sapiens OX=9606 GN=GCLM PE=1 SV=1
sp|P46379|BAG6_HUMAN	HUMAN	Large proline-rich protein BAG6 OS=Homo sapiens OX=9606 GN=BAG6 PE=1 SV=2
sp|P36639|8ODP_HUMAN	HUMAN	7,8-dihydro-8-oxoguanine triphosphatase OS=Homo sapiens OX=9606 GN=NUDT1 PE=1 SV=3
sp|P31949|S10AB_HUMAN	HUMAN	Protein S100-A11 OS=Homo sapiens OX=9606 GN=S100A11 PE=1 SV=2
sp|P31937|3HIDH_HUMAN	HUMAN	3-hydroxyisobutyrate dehydrogenase, mitochondrial OS=Homo sapiens OX=9606 GN=HIBADH PE=1 SV=2
sp|P17096|HMGA1_HUMAN	HUMAN	High mobility group protein HMG-I/HMG-Y OS=Homo sapiens OX=9606 GN=HMGA1 PE=1 SV=3
sp|O75607|NPM3_HUMAN	HUMAN	Nucleoplasmin-3 OS=Homo sapiens OX=9606 GN=NPM3 PE=1 SV=3
sp|O43681|ASNA_HUMAN	HUMAN	ATPase ASNA1 OS=Homo sapiens OX=9606 GN=ASNA1 PE=1 SV=2
sp|O00267|SPT5H_HUMAN	HUMAN	Transcription elongation factor SPT5 OS=Homo sapiens OX=9606 GN=SUPT5H PE=1 SV=1
sp|Q96D71|REPS1_HUMAN	HUMAN	RalBP1-associated Eps domain-containing protein 1 OS=Homo sapiens OX=9606 GN=REPS1 PE=1 SV=3
sp|Q9UG63|ABCF2_HUMAN	HUMAN	ATP-binding cassette sub-family F member 2 OS=Homo sapiens OX=9606 GN=ABCF2 PE=1 SV=2
sp|Q15813|TBCE_HUMAN	HUMAN	Tubulin-specific chaperone E OS=Homo sapiens OX=9606 GN=TBCE PE=1 SV=1

sp|Q9BXR0|TGT_HUMAN	HUMAN	Queuine tRNA-ribosyltransferase catalytic subunit 1 OS=Homo sapiens OX=9606 GN=QTRT1 PE=1 SV=3
sp|Q13595|TRA2A_HUMAN	HUMAN	Transformer-2 protein homolog alpha OS=Homo sapiens OX=9606 GN=TRA2A PE=1 SV=1
sp|P50579|MAP2_HUMAN	HUMAN	Methionine aminopeptidase 2 OS=Homo sapiens OX=9606 GN=METAP2 PE=1 SV=1
sp|Q96KB5|TOPK_HUMAN	HUMAN	Lymphokine-activated killer T-cell-originated protein kinase OS=Homo sapiens OX=9606 GN=PBK PE=1 SV=3
sp|Q92530|PSMF1_HUMAN	HUMAN	Proteasome inhibitor PI31 subunit OS=Homo sapiens OX=9606 GN=PSMF1 PE=1 SV=2
sp|Q9H6T3|RPAP3_HUMAN	HUMAN	RNA polymerase II-associated protein 3 OS=Homo sapiens OX=9606 GN=RPAP3 PE=1 SV=2
sp|P22307|NLTP_HUMAN	HUMAN	Non-specific lipid-transfer protein OS=Homo sapiens OX=9606 GN=SCP2 PE=1 SV=2
sp|Q15785|TOM34_HUMAN	HUMAN	Mitochondrial import receptor subunit TOM34 OS=Homo sapiens OX=9606 GN=TOMM34 PE=1 SV=2
sp|Q15369|ELOC_HUMAN	HUMAN	Elongin-C OS=Homo sapiens OX=9606 GN=ELOC PE=1 SV=1
sp|O43516|WIPF1_HUMAN	HUMAN	WAS/WASL-interacting protein family member 1 OS=Homo sapiens OX=9606 GN=WIPF1 PE=1 SV=3
sp|P15498|VAV_HUMAN	HUMAN	Proto-oncogene vav OS=Homo sapiens OX=9606 GN=VAV1 PE=1 SV=4
sp|Q99436|PSB7_HUMAN	HUMAN	Proteasome subunit beta type-7 OS=Homo sapiens OX=9606 GN=PSMB7 PE=1 SV=1
sp|Q9NZJ9|NUDT4_HUMAN	HUMAN	Diphosphoinositol polyphosphate phosphohydrolase 2 OS=Homo sapiens OX=9606 GN=NUDT4 PE=1 SV=2
sp|P37198|NUP62_HUMAN	HUMAN	Nuclear pore glycoprotein p62 OS=Homo sapiens OX=9606 GN=NUP62 PE=1 SV=3
sp|P36957|ODO2_HUMAN	HUMAN	Dihydrolipoyllysine-residue succinyltransferase component of 2-oxoglutarate dehydrogenase complex, mitochondrial OS=Homo sapiens OX=9606 GN=DLST PE=1 SV=4
sp|P23469|PTPRE_HUMAN	HUMAN	Receptor-type tyrosine-protein phosphatase epsilon OS=Homo sapiens OX=9606 GN=PTPRE PE=1 SV=1
sp|Q9NP77|SSU72_HUMAN	HUMAN	RNA polymerase II subunit A C-terminal domain phosphatase SSU72 OS=Homo sapiens OX=9606 GN=SSU72 PE=1 SV=1
sp|O43665|RGS10_HUMAN	HUMAN	Regulator of G-protein signaling 10 OS=Homo sapiens OX=9606 GN=RGS10 PE=1 SV=3
sp|Q9UI12|VATH_HUMAN	HUMAN	V-type proton ATPase subunit H OS=Homo sapiens OX=9606 GN=ATP6V1H PE=1 SV=1
sp|Q86UA1|PRP39_HUMAN	HUMAN	Pre-mRNA-processing factor 39 OS=Homo sapiens OX=9606 GN=PRPF39 PE=1 SV=3
sp|Q96AG4|LRC59_HUMAN	HUMAN	Leucine-rich repeat-containing protein 59 OS=Homo sapiens OX=9606 GN=LRRC59 PE=1 SV=1
sp|Q9UBW8|CSN7A_HUMAN	HUMAN	COP9 signalosome complex subunit 7a OS=Homo sapiens OX=9606 GN=COPS7A PE=1 SV=1
sp|Q9Y4Z0|LSM4_HUMAN	HUMAN	U6 snRNA-associated Sm-like protein LSm4 OS=Homo sapiens OX=9606 GN=LSM4 PE=1 SV=1
sp|Q92804|RBP56_HUMAN	HUMAN	TATA-binding protein-associated factor 2N OS=Homo sapiens OX=9606 GN=TAF15 PE=1 SV=1
sp|Q8NBF2|NHLC2_HUMAN	HUMAN	NHL repeat-containing protein 2 OS=Homo sapiens OX=9606 GN=NHLRC2 PE=1 SV=1
sp|Q6IA69|NADE_HUMAN	HUMAN	Glutamine-dependent NAD(+) synthetase OS=Homo sapiens OX=9606 GN=NADSYN1 PE=1 SV=3
sp|O95571|ETHE1_HUMAN	HUMAN	Persulfide dioxygenase ETHE1, mitochondrial OS=Homo sapiens OX=9606 GN=ETHE1 PE=1 SV=2
sp|Q9Y316|MEMO1_HUMAN	HUMAN	Protein MEMO1 OS=Homo sapiens OX=9606 GN=MEMO1 PE=1 SV=1
sp|P42766|RL35_HUMAN	HUMAN	60S ribosomal protein L35 OS=Homo sapiens OX=9606 GN=RPL35 PE=1 SV=2

sp|Q14978|NOLC1_HUMAN	HUMAN	Nucleolar and coiled-body phosphoprotein 1 OS=Homo sapiens OX=9606 GN=NOLC1 PE=1 SV=2
sp|P28340|DPOD1_HUMAN	HUMAN	DNA polymerase delta catalytic subunit OS=Homo sapiens OX=9606 GN=POLD1 PE=1 SV=2
sp|Q14790|CASP8_HUMAN	HUMAN	Caspase-8 OS=Homo sapiens OX=9606 GN=CASP8 PE=1 SV=1
sp|Q9NSE4|SYIM_HUMAN	HUMAN	Isoleucine--tRNA ligase, mitochondrial OS=Homo sapiens OX=9606 GN=IARS2 PE=1 SV=2
sp|P32320|CDD_HUMAN	HUMAN	Cytidine deaminase OS=Homo sapiens OX=9606 GN=CDA PE=1 SV=2
sp|Q9Y6M1|IF2B2_HUMAN	HUMAN	Insulin-like growth factor 2 mRNA-binding protein 2 OS=Homo sapiens OX=9606 GN=IGF2BP2 PE=1 SV=2
sp|Q14116|IL18_HUMAN	HUMAN	Interleukin-18 OS=Homo sapiens OX=9606 GN=IL18 PE=1 SV=1
sp|Q04864|REL_HUMAN	HUMAN	Proto-oncogene c-Rel OS=Homo sapiens OX=9606 GN=REL PE=1 SV=1
sp|O60763|USO1_HUMAN	HUMAN	General vesicular transport factor p115 OS=Homo sapiens OX=9606 GN=USO1 PE=1 SV=2
sp|P11177|ODPB_HUMAN	HUMAN	Pyruvate dehydrogenase E1 component subunit beta, mitochondrial OS=Homo sapiens OX=9606 GN=PDHB PE=1 SV=3
sp|P04843|RPN1_HUMAN	HUMAN	Dolichyl-diphosphooligosaccharide--protein glycosyltransferase subunit 1 OS=Homo sapiens OX=9606 GN=RPN1 PE=1 SV=1
sp|Q05655|KPCD_HUMAN	HUMAN	Protein kinase C delta type OS=Homo sapiens OX=9606 GN=PRKCD PE=1 SV=2
sp|Q9NRF9|DPOE3_HUMAN	HUMAN	DNA polymerase epsilon subunit 3 OS=Homo sapiens OX=9606 GN=POLE3 PE=1 SV=1
sp|O43617|TPPC3_HUMAN	HUMAN	Trafficking protein particle complex subunit 3 OS=Homo sapiens OX=9606 GN=TRAPPC3 PE=1 SV=1
sp|Q8IYB5|SMAP1_HUMAN	HUMAN	Stromal membrane-associated protein 1 OS=Homo sapiens OX=9606 GN=SMAP1 PE=1 SV=2
sp|P24158|PRTN3_HUMAN	HUMAN	Myeloblastin OS=Homo sapiens OX=9606 GN=PRTN3 PE=1 SV=3
sp|P25685|DNJB1_HUMAN	HUMAN	DnaJ homolog subfamily B member 1 OS=Homo sapiens OX=9606 GN=DNAJB1 PE=1 SV=4
sp|Q09161|NCBP1_HUMAN	HUMAN	Nuclear cap-binding protein subunit 1 OS=Homo sapiens OX=9606 GN=NCBP1 PE=1 SV=1
sp|Q14696|MESD_HUMAN	HUMAN	LRP chaperone MESD OS=Homo sapiens OX=9606 GN=MESD PE=1 SV=2
sp|P82979|SARNP_HUMAN	HUMAN	SAP domain-containing ribonucleoprotein OS=Homo sapiens OX=9606 GN=SARNP PE=1 SV=3
sp|Q8WWM7|ATX2L_HUMAN	HUMAN	Ataxin-2-like protein OS=Homo sapiens OX=9606 GN=ATXN2L PE=1 SV=2
sp|Q15436|SC23A_HUMAN	HUMAN	Protein transport protein Sec23A OS=Homo sapiens OX=9606 GN=SEC23A PE=1 SV=2
sp|P31150|GDIA_HUMAN	HUMAN	Rab GDP dissociation inhibitor alpha OS=Homo sapiens OX=9606 GN=GDI1 PE=1 SV=2
sp|Q8NBJ5|GT251_HUMAN	HUMAN	Procollagen galactosyltransferase 1 OS=Homo sapiens OX=9606 GN=COLGALT1 PE=1 SV=1
sp|P61313|RL15_HUMAN	HUMAN	60S ribosomal protein L15 OS=Homo sapiens OX=9606 GN=RPL15 PE=1 SV=2
sp|P63167|DYL1_HUMAN	HUMAN	Dynein light chain 1, cytoplasmic OS=Homo sapiens OX=9606 GN=DYNLL1 PE=1 SV=1
sp|Q9UBS4|DJB11_HUMAN	HUMAN	DnaJ homolog subfamily B member 11 OS=Homo sapiens OX=9606 GN=DNAJB11 PE=1 SV=1
sp|Q9NRX4|PHP14_HUMAN	HUMAN	14 kDa phosphohistidine phosphatase OS=Homo sapiens OX=9606 GN=PHPT1 PE=1 SV=1
sp|Q15428|SF3A2_HUMAN	HUMAN	Splicing factor 3A subunit 2 OS=Homo sapiens OX=9606 GN=SF3A2 PE=1 SV=2
sp|O75792|RNH2A_HUMAN	HUMAN	Ribonuclease H2 subunit A OS=Homo sapiens OX=9606 GN=RNASEH2A PE=1 SV=2

sp|Q9UNE7|CHIP_HUMAN	HUMAN	E3 ubiquitin-protein ligase CHIP OS=Homo sapiens OX=9606 GN=STUB1 PE=1 SV=2
sp|P07858|CATB_HUMAN	HUMAN	Cathepsin B OS=Homo sapiens OX=9606 GN=CTSB PE=1 SV=3
sp|O60784|TOM1_HUMAN	HUMAN	Target of Myb protein 1 OS=Homo sapiens OX=9606 GN=TOM1 PE=1 SV=2
sp|P62834|RAP1A_HUMAN	HUMAN	Ras-related protein Rap-1A OS=Homo sapiens OX=9606 GN=RAP1A PE=1 SV=1
sp|P53634|CATC_HUMAN	HUMAN	Dipeptidyl peptidase 1 OS=Homo sapiens OX=9606 GN=CTSC PE=1 SV=2
sp|Q8N3C0|ASCC3_HUMAN	HUMAN	Activating signal cointegrator 1 complex subunit 3 OS=Homo sapiens OX=9606 GN=ASCC3 PE=1 SV=3
sp|Q9UNN5|FAF1_HUMAN	HUMAN	FAS-associated factor 1 OS=Homo sapiens OX=9606 GN=FAF1 PE=1 SV=2
sp|P54886|P5CS_HUMAN	HUMAN	Delta-1-pyrroline-5-carboxylate synthase OS=Homo sapiens OX=9606 GN=ALDH18A1 PE=1 SV=2
sp|Q13363|CTBP1_HUMAN	HUMAN	C-terminal-binding protein 1 OS=Homo sapiens OX=9606 GN=CTBP1 PE=1 SV=2
sp|Q9NXH9|TRM1_HUMAN	HUMAN	tRNA (guanine(26)-N(2))-dimethyltransferase OS=Homo sapiens OX=9606 GN=TRMT1 PE=1 SV=1
sp|P40818|UBP8_HUMAN	HUMAN	Ubiquitin carboxyl-terminal hydrolase 8 OS=Homo sapiens OX=9606 GN=USP8 PE=1 SV=1
sp|Q9UNP9|PPIE_HUMAN	HUMAN	Peptidyl-prolyl cis-trans isomerase E OS=Homo sapiens OX=9606 GN=PPIE PE=1 SV=1
sp|O15027|SC16A_HUMAN	HUMAN	Protein transport protein Sec16A OS=Homo sapiens OX=9606 GN=SEC16A PE=1 SV=4
sp|Q99615|DNJC7_HUMAN	HUMAN	DnaJ homolog subfamily C member 7 OS=Homo sapiens OX=9606 GN=DNAJC7 PE=1 SV=2
sp|Q96GX9|MTNB_HUMAN	HUMAN	Methylthioribulose-1-phosphate dehydratase OS=Homo sapiens OX=9606 GN=APIP PE=1 SV=1
sp|O43809|CPSF5_HUMAN	HUMAN	Cleavage and polyadenylation specificity factor subunit 5 OS=Homo sapiens OX=9606 GN=NUDT21 PE=1 SV=1
sp|O00764|PDXK_HUMAN	HUMAN	Pyridoxal kinase OS=Homo sapiens OX=9606 GN=PDXK PE=1 SV=1
sp|Q8IYB3|SRRM1_HUMAN	HUMAN	Serine/arginine repetitive matrix protein 1 OS=Homo sapiens OX=9606 GN=SRRM1 PE=1 SV=2
sp|O43865|SAHH2_HUMAN	HUMAN	S-adenosylhomocysteine hydrolase-like protein 1 OS=Homo sapiens OX=9606 GN=AHCYL1 PE=1 SV=2
sp|P09012|SNRPA_HUMAN	HUMAN	U1 small nuclear ribonucleoprotein A OS=Homo sapiens OX=9606 GN=SNRPA PE=1 SV=3
sp|Q02750|MP2K1_HUMAN	HUMAN	Dual specificity mitogen-activated protein kinase kinase 1 OS=Homo sapiens OX=9606 GN=MAP2K1 PE=1 SV=2
sp|Q53GS9|SNUT2_HUMAN	HUMAN	U4/U6.U5 tri-snRNP-associated protein 2 OS=Homo sapiens OX=9606 GN=USP39 PE=1 SV=2
sp|O94855|SC24D_HUMAN	HUMAN	Protein transport protein Sec24D OS=Homo sapiens OX=9606 GN=SEC24D PE=1 SV=2
sp|B0I1T2|MYO1G_HUMAN	HUMAN	Unconventional myosin-Ig OS=Homo sapiens OX=9606 GN=MYO1G PE=1 SV=2
sp|P50213|IDH3A_HUMAN	HUMAN	Isocitrate dehydrogenase [NAD] subunit alpha, mitochondrial OS=Homo sapiens OX=9606 GN=IDH3A PE=1 SV=1
sp|Q969U7|PSMG2_HUMAN	HUMAN	Proteasome assembly chaperone 2 OS=Homo sapiens OX=9606 GN=PSMG2 PE=1 SV=1
sp|P10253|LYAG_HUMAN	HUMAN	Lysosomal alpha-glucosidase OS=Homo sapiens OX=9606 GN=GAA PE=1 SV=4
sp|P60510|PP4C_HUMAN	HUMAN	Serine/threonine-protein phosphatase 4 catalytic subunit OS=Homo sapiens OX=9606 GN=PPP4C PE=1 SV=1

sp|P55735|SEC13_HUMAN	HUMAN	Protein SEC13 homolog OS=Homo sapiens OX=9606 GN=SEC13 PE=1 SV=3
sp|P19838|NFKB1_HUMAN	HUMAN	Nuclear factor NF-kappa-B p105 subunit OS=Homo sapiens OX=9606 GN=NFKB1 PE=1 SV=2
sp|Q7Z5L9|I2BP2_HUMAN	HUMAN	Interferon regulatory factor 2-binding protein 2 OS=Homo sapiens OX=9606 GN=IRF2BP2 PE=1 SV=2
sp|Q9BXS5|AP1M1_HUMAN	HUMAN	AP-1 complex subunit mu-1 OS=Homo sapiens OX=9606 GN=AP1M1 PE=1 SV=3
sp|P53992|SC24C_HUMAN	HUMAN	Protein transport protein Sec24C OS=Homo sapiens OX=9606 GN=SEC24C PE=1 SV=3
sp|Q9NUJ1|ABHDA_HUMAN	HUMAN	Mycophenolic acid acyl-glucuronide esterase, mitochondrial OS=Homo sapiens OX=9606 GN=ABHD10 PE=1 SV=1
sp|Q9BUQ8|DDX23_HUMAN	HUMAN	Probable ATP-dependent RNA helicase DDX23 OS=Homo sapiens OX=9606 GN=DDX23 PE=1 SV=3
sp|Q13144|EI2BE_HUMAN	HUMAN	Translation initiation factor eIF-2B subunit epsilon OS=Homo sapiens OX=9606 GN=EIF2B5 PE=1 SV=3
sp|P61106|RAB14_HUMAN	HUMAN	Ras-related protein Rab-14 OS=Homo sapiens OX=9606 GN=RAB14 PE=1 SV=4
sp|P83731|RL24_HUMAN	HUMAN	60S ribosomal protein L24 OS=Homo sapiens OX=9606 GN=RPL24 PE=1 SV=1
sp|Q9Y333|LSM2_HUMAN	HUMAN	U6 snRNA-associated Sm-like protein LSm2 OS=Homo sapiens OX=9606 GN=LSM2 PE=1 SV=1
sp|Q9Y3C4|TPRKB_HUMAN	HUMAN	EKC/KEOPS complex subunit TPRKB OS=Homo sapiens OX=9606 GN=TPRKB PE=1 SV=1
sp|Q15126|PMVK_HUMAN	HUMAN	Phosphomevalonate kinase OS=Homo sapiens OX=9606 GN=PMVK PE=1 SV=3
sp|Q06787|FMR1_HUMAN	HUMAN	Synaptic functional regulator FMR1 OS=Homo sapiens OX=9606 GN=FMR1 PE=1 SV=1
sp|A0AVT1|UBA6_HUMAN	HUMAN	Ubiquitin-like modifier-activating enzyme 6 OS=Homo sapiens OX=9606 GN=UBA6 PE=1 SV=1
sp|Q13464|ROCK1_HUMAN	HUMAN	Rho-associated protein kinase 1 OS=Homo sapiens OX=9606 GN=ROCK1 PE=1 SV=1
sp|Q9P2E9|RRBP1_HUMAN	HUMAN	Ribosome-binding protein 1 OS=Homo sapiens OX=9606 GN=RRBP1 PE=1 SV=5
sp|Q8WUA2|PPIL4_HUMAN	HUMAN	Peptidyl-prolyl cis-trans isomerase-like 4 OS=Homo sapiens OX=9606 GN=PPIL4 PE=1 SV=1
sp|O95239|KIF4A_HUMAN	HUMAN	Chromosome-associated kinesin KIF4A OS=Homo sapiens OX=9606 GN=KIF4A PE=1 SV=3
sp|Q92974|ARHG2_HUMAN	HUMAN	Rho guanine nucleotide exchange factor 2 OS=Homo sapiens OX=9606 GN=ARHGEF2 PE=1 SV=4
sp|Q6L8Q7|PDE12_HUMAN	HUMAN	2',5'-phosphodiesterase 12 OS=Homo sapiens OX=9606 GN=PDE12 PE=1 SV=2
sp|Q15370|ELOB_HUMAN	HUMAN	Elongin-B OS=Homo sapiens OX=9606 GN=ELOB PE=1 SV=1
sp|P62851|RS25_HUMAN	HUMAN	40S ribosomal protein S25 OS=Homo sapiens OX=9606 GN=RPS25 PE=1 SV=1
sp|O60493|SNX3_HUMAN	HUMAN	Sorting nexin-3 OS=Homo sapiens OX=9606 GN=SNX3 PE=1 SV=3
sp|Q9BVK6|TMED9_HUMAN	HUMAN	Transmembrane emp24 domain-containing protein 9 OS=Homo sapiens OX=9606 GN=TMED9 PE=1 SV=2
sp|Q71UM5|RS27L_HUMAN	HUMAN	40S ribosomal protein S27-like OS=Homo sapiens OX=9606 GN=RPS27L PE=1 SV=3
sp|P40763|STAT3_HUMAN	HUMAN	Signal transducer and activator of transcription 3 OS=Homo sapiens OX=9606 GN=STAT3 PE=1 SV=2
sp|Q9UJY4|GGA2_HUMAN	HUMAN	ADP-ribosylation factor-binding protein GGA2 OS=Homo sapiens OX=9606 GN=GGA2 PE=1 SV=3
sp|P55265|DSRAD_HUMAN	HUMAN	Double-stranded RNA-specific adenosine deaminase OS=Homo sapiens OX=9606 GN=ADAR PE=1 SV=4
sp|Q9H307|PININ_HUMAN	HUMAN	Pinin OS=Homo sapiens OX=9606 GN=PNN PE=1 SV=5

sp|P49366|DHYS_HUMAN	HUMAN	Deoxyhypusine synthase OS=Homo sapiens OX=9606 GN=DHPS PE=1 SV=1
sp|Q92922|SMRC1_HUMAN	HUMAN	SWI/SNF complex subunit SMARCC1 OS=Homo sapiens OX=9606 GN=SMARCC1 PE=1 SV=3
sp|Q9UPT8|ZC3H4_HUMAN	HUMAN	Zinc finger CCCH domain-containing protein 4 OS=Homo sapiens OX=9606 GN=ZC3H4 PE=1 SV=3
sp|Q9H8W4|PKHF2_HUMAN	HUMAN	Pleckstrin homology domain-containing family F member 2 OS=Homo sapiens OX=9606 GN=PLEKHF2 PE=1 SV=1
sp|P61962|DCAF7_HUMAN	HUMAN	DDB1- and CUL4-associated factor 7 OS=Homo sapiens OX=9606 GN=DCAF7 PE=1 SV=1
sp|P0DP25|CALM3_HUMAN	HUMAN	Calmodulin-3 OS=Homo sapiens OX=9606 GN=CALM3 PE=1 SV=1
sp|P46778|RL21_HUMAN	HUMAN	60S ribosomal protein L21 OS=Homo sapiens OX=9606 GN=RPL21 PE=1 SV=2
sp|Q8NBT2|SPC24_HUMAN	HUMAN	Kinetochore protein Spc24 OS=Homo sapiens OX=9606 GN=SPC24 PE=1 SV=2
sp|Q9BS26|ERP44_HUMAN	HUMAN	Endoplasmic reticulum resident protein 44 OS=Homo sapiens OX=9606 GN=ERP44 PE=1 SV=1
sp|Q8N6M0|OTU6B_HUMAN	HUMAN	Deubiquitinase OTUD6B OS=Homo sapiens OX=9606 GN=OTUD6B PE=1 SV=1
sp|Q86VP1|TAXB1_HUMAN	HUMAN	Tax1-binding protein 1 OS=Homo sapiens OX=9606 GN=TAX1BP1 PE=1 SV=2
sp|Q8IZP0|ABI1_HUMAN	HUMAN	Abl interactor 1 OS=Homo sapiens OX=9606 GN=ABI1 PE=1 SV=4
sp|O14979|HNRDL_HUMAN	HUMAN	Heterogeneous nuclear ribonucleoprotein D-like OS=Homo sapiens OX=9606 GN=HNRNPDL PE=1 SV=3
sp|O95154|ARK73_HUMAN	HUMAN	Aflatoxin B1 aldehyde reductase member 3 OS=Homo sapiens OX=9606 GN=AKR7A3 PE=1 SV=2
sp|P49207|RL34_HUMAN	HUMAN	60S ribosomal protein L34 OS=Homo sapiens OX=9606 GN=RPL34 PE=1 SV=3
sp|P49407|ARRB1_HUMAN	HUMAN	Beta-arrestin-1 OS=Homo sapiens OX=9606 GN=ARRB1 PE=1 SV=2
sp|Q6UX04|CWC27_HUMAN	HUMAN	Spliceosome-associated protein CWC27 homolog OS=Homo sapiens OX=9606 GN=CWC27 PE=1 SV=1
sp|O60678|ANM3_HUMAN	HUMAN	Protein arginine N-methyltransferase 3 OS=Homo sapiens OX=9606 GN=PRMT3 PE=1 SV=4
sp|Q1KMD3|HNRL2_HUMAN	HUMAN	Heterogeneous nuclear ribonucleoprotein U-like protein 2 OS=Homo sapiens OX=9606 GN=HNRNPUL2 PE=1 SV=1
sp|Q9Y3D0|CIA2B_HUMAN	HUMAN	Cytosolic iron-sulfur assembly component 2B OS=Homo sapiens OX=9606 GN=CIAO2B PE=1 SV=1
sp|Q86UP2|KTN1_HUMAN	HUMAN	Kinectin OS=Homo sapiens OX=9606 GN=KTN1 PE=1 SV=1
sp|O75592|MYCB2_HUMAN	HUMAN	E3 ubiquitin-protein ligase MYCBP2 OS=Homo sapiens OX=9606 GN=MYCBP2 PE=1 SV=4
sp|O15397|IPO8_HUMAN	HUMAN	Importin-8 OS=Homo sapiens OX=9606 GN=IPO8 PE=1 SV=2
sp|P35613|BASI_HUMAN	HUMAN	Basigin OS=Homo sapiens OX=9606 GN=BSG PE=1 SV=2
sp|P30876|RPB2_HUMAN	HUMAN	DNA-directed RNA polymerase II subunit RPB2 OS=Homo sapiens OX=9606 GN=POLR2B PE=1 SV=1
sp|P29466|CASP1_HUMAN	HUMAN	Caspase-1 OS=Homo sapiens OX=9606 GN=CASP1 PE=1 SV=1
sp|Q9NY12|GAR1_HUMAN	HUMAN	H/ACA ribonucleoprotein complex subunit 1 OS=Homo sapiens OX=9606 GN=GAR1 PE=1 SV=1
sp|P52434|RPAB3_HUMAN	HUMAN	DNA-directed RNA polymerases I, II, and III subunit RPABC3 OS=Homo sapiens OX=9606 GN=POLR2H PE=1 SV=4

sp|Q9P0L0|VAPA_HUMAN	HUMAN	Vesicle-associated membrane protein-associated protein A OS=Homo sapiens OX=9606 GN=VAPA PE=1 SV=3
sp|Q8IUD2|RB6I2_HUMAN	HUMAN	ELKS/Rab6-interacting/CAST family member 1 OS=Homo sapiens OX=9606 GN=ERC1 PE=1 SV=1
sp|P41218|MNDA_HUMAN	HUMAN	Myeloid cell nuclear differentiation antigen OS=Homo sapiens OX=9606 GN=MNDA PE=1 SV=1
sp|Q5T4S7|UBR4_HUMAN	HUMAN	E3 ubiquitin-protein ligase UBR4 OS=Homo sapiens OX=9606 GN=UBR4 PE=1 SV=1
sp|P19174|PLCG1_HUMAN	HUMAN	1-phosphatidylinositol 4,5-bisphosphate phosphodiesterase gamma-1 OS=Homo sapiens OX=9606 GN=PLCG1 PE=1 SV=1
sp|P46108|CRK_HUMAN	HUMAN	Adapter molecule crk OS=Homo sapiens OX=9606 GN=CRK PE=1 SV=2
sp|Q15149|PLEC_HUMAN	HUMAN	Plectin OS=Homo sapiens OX=9606 GN=PLEC PE=1 SV=3
sp|Q9UJ68|MSRA_HUMAN	HUMAN	Mitochondrial peptide methionine sulfoxide reductase OS=Homo sapiens OX=9606 GN=MSRA PE=1 SV=1
sp|Q9Y5K6|CD2AP_HUMAN	HUMAN	CD2-associated protein OS=Homo sapiens OX=9606 GN=CD2AP PE=1 SV=1
sp|O75191|XYLB_HUMAN	HUMAN	Xylulose kinase OS=Homo sapiens OX=9606 GN=XYLB PE=1 SV=3
sp|Q08378|GOGA3_HUMAN	HUMAN	Golgin subfamily A member 3 OS=Homo sapiens OX=9606 GN=GOLGA3 PE=1 SV=2
sp|Q2M389|WASC4_HUMAN	HUMAN	WASH complex subunit 4 OS=Homo sapiens OX=9606 GN=WASHC4 PE=1 SV=2
sp|O43314|VIP2_HUMAN	HUMAN	Inositol hexakisphosphate and diphosphoinositol-pentakisphosphate kinase 2 OS=Homo sapiens OX=9606 GN=PPIP5K2 PE=1 SV=3
sp|P30626|SORCN_HUMAN	HUMAN	Sorcin OS=Homo sapiens OX=9606 GN=SRI PE=1 SV=1
sp|P56537|IF6_HUMAN	HUMAN	Eukaryotic translation initiation factor 6 OS=Homo sapiens OX=9606 GN=EIF6 PE=1 SV=1
sp|P68133|ACTS_HUMAN	HUMAN	Actin, alpha skeletal muscle OS=Homo sapiens OX=9606 GN=ACTA1 PE=1 SV=1
sp|Q15274|NADC_HUMAN	HUMAN	Nicotinate-nucleotide pyrophosphorylase [carboxylating] OS=Homo sapiens OX=9606 GN=QPRT PE=1 SV=3
sp|P50552|VASP_HUMAN	HUMAN	Vasodilator-stimulated phosphoprotein OS=Homo sapiens OX=9606 GN=VASP PE=1 SV=3
sp|P49005|DPOD2_HUMAN	HUMAN	DNA polymerase delta subunit 2 OS=Homo sapiens OX=9606 GN=POLD2 PE=1 SV=1
sp|Q9UK45|LSM7_HUMAN	HUMAN	U6 snRNA-associated Sm-like protein LSm7 OS=Homo sapiens OX=9606 GN=LSM7 PE=1 SV=1
sp|P85037|FOXK1_HUMAN	HUMAN	Forkhead box protein K1 OS=Homo sapiens OX=9606 GN=FOXK1 PE=1 SV=1
sp|P53597|SUCA_HUMAN	HUMAN	Succinate--CoA ligase [ADP/GDP-forming] subunit alpha, mitochondrial OS=Homo sapiens OX=9606 GN=SUCLG1 PE=1 SV=4
sp|Q92614|MY18A_HUMAN	HUMAN	Unconventional myosin-XVIIIa OS=Homo sapiens OX=9606 GN=MYO18A PE=1 SV=3
sp|Q8TF42|UBS3B_HUMAN	HUMAN	Ubiquitin-associated and SH3 domain-containing protein B OS=Homo sapiens OX=9606 GN=UBASH3B PE=1 SV=2
sp|P60981|DEST_HUMAN	HUMAN	Destrin OS=Homo sapiens OX=9606 GN=DSTN PE=1 SV=3
sp|P10619|PPGB_HUMAN	HUMAN	Lysosomal protective protein OS=Homo sapiens OX=9606 GN=CTSA PE=1 SV=2
sp|O60888|CUTA_HUMAN	HUMAN	Protein CutA OS=Homo sapiens OX=9606 GN=CUTA PE=1 SV=2
sp|Q9NRH3|TBG2_HUMAN	HUMAN	Tubulin gamma-2 chain OS=Homo sapiens OX=9606 GN=TUBG2 PE=2 SV=1

sp|Q7Z2Z2|EFL1_HUMAN	HUMAN	Elongation factor-like GTPase 1 OS=Homo sapiens OX=9606 GN=EFL1 PE=1 SV=2
sp|Q8N806|UBR7_HUMAN	HUMAN	Putative E3 ubiquitin-protein ligase UBR7 OS=Homo sapiens OX=9606 GN=UBR7 PE=1 SV=2
sp|Q92835|SHIP1_HUMAN	HUMAN	Phosphatidylinositol 3,4,5-trisphosphate 5-phosphatase 1 OS=Homo sapiens OX=9606 GN=INPP5D PE=1 SV=2
sp|Q9Y6I3|EPN1_HUMAN	HUMAN	Epsin-1 OS=Homo sapiens OX=9606 GN=EPN1 PE=1 SV=2
sp|Q92905|CSN5_HUMAN	HUMAN	COP9 signalosome complex subunit 5 OS=Homo sapiens OX=9606 GN=COPS5 PE=1 SV=4
sp|P19388|RPAB1_HUMAN	HUMAN	DNA-directed RNA polymerases I, II, and III subunit RPABC1 OS=Homo sapiens OX=9606 GN=POLR2E PE=1 SV=4
sp|Q92696|PGTA_HUMAN	HUMAN	Geranylgeranyl transferase type-2 subunit alpha OS=Homo sapiens OX=9606 GN=RABGGTA PE=1 SV=2
sp|Q9Y5A9|YTHD2_HUMAN	HUMAN	YTH domain-containing family protein 2 OS=Homo sapiens OX=9606 GN=YTHDF2 PE=1 SV=2
sp|Q9H7E9|CH033_HUMAN	HUMAN	UPF0488 protein C8orf33 OS=Homo sapiens OX=9606 GN=C8orf33 PE=1 SV=1
sp|P49643|PRI2_HUMAN	HUMAN	DNA primase large subunit OS=Homo sapiens OX=9606 GN=PRIM2 PE=1 SV=2
sp|P22694|KAPCB_HUMAN	HUMAN	cAMP-dependent protein kinase catalytic subunit beta OS=Homo sapiens OX=9606 GN=PRKACB PE=1 SV=2
sp|Q14651|PLSI_HUMAN	HUMAN	Plastin-1 OS=Homo sapiens OX=9606 GN=PLS1 PE=1 SV=2
sp|Q9Y3A5|SBDS_HUMAN	HUMAN	Ribosome maturation protein SBDS OS=Homo sapiens OX=9606 GN=SBDS PE=1 SV=4
sp|P63010|AP2B1_HUMAN	HUMAN	AP-2 complex subunit beta OS=Homo sapiens OX=9606 GN=AP2B1 PE=1 SV=1
sp|Q9NQC3|RTN4_HUMAN	HUMAN	Reticulon-4 OS=Homo sapiens OX=9606 GN=RTN4 PE=1 SV=2
sp|P78406|RAE1L_HUMAN	HUMAN	mRNA export factor OS=Homo sapiens OX=9606 GN=RAE1 PE=1 SV=1
sp|P24941|CDK2_HUMAN	HUMAN	Cyclin-dependent kinase 2 OS=Homo sapiens OX=9606 GN=CDK2 PE=1 SV=2
sp|Q96IV0|NGLY1_HUMAN	HUMAN	Peptide-N(4)-(N-acetyl-beta-glucosaminyl)asparagine amidase OS=Homo sapiens OX=9606 GN=NGLY1 PE=1 SV=1
sp|Q96BW5|PTER_HUMAN	HUMAN	Phosphotriesterase-related protein OS=Homo sapiens OX=9606 GN=PTER PE=1 SV=1
sp|Q9UQ35|SRRM2_HUMAN	HUMAN	Serine/arginine repetitive matrix protein 2 OS=Homo sapiens OX=9606 GN=SRRM2 PE=1 SV=2
sp|Q5VTR2|BRE1A_HUMAN	HUMAN	E3 ubiquitin-protein ligase BRE1A OS=Homo sapiens OX=9606 GN=RNF20 PE=1 SV=2
sp|O60826|CCD22_HUMAN	HUMAN	Coiled-coil domain-containing protein 22 OS=Homo sapiens OX=9606 GN=CCDC22 PE=1 SV=1
sp|O14773|TPP1_HUMAN	HUMAN	Tripeptidyl-peptidase 1 OS=Homo sapiens OX=9606 GN=TPP1 PE=1 SV=2
sp|Q04837|SSBP_HUMAN	HUMAN	Single-stranded DNA-binding protein, mitochondrial OS=Homo sapiens OX=9606 GN=SSBP1 PE=1 SV=1
sp|Q16763|UBE2S_HUMAN	HUMAN	Ubiquitin-conjugating enzyme E2 S OS=Homo sapiens OX=9606 GN=UBE2S PE=1 SV=2
sp|Q13555|KCC2G_HUMAN	HUMAN	Calcium/calmodulin-dependent protein kinase type II subunit gamma OS=Homo sapiens OX=9606 GN=CAMK2G PE=1 SV=4
sp|Q9H2K8|TAOK3_HUMAN	HUMAN	Serine/threonine-protein kinase TAO3 OS=Homo sapiens OX=9606 GN=TAOK3 PE=1 SV=2
sp|Q07666|KHDR1_HUMAN	HUMAN	KH domain-containing, RNA-binding, signal transduction-associated protein 1 OS=Homo sapiens OX=9606 GN=KHDRBS1 PE=1 SV=1

sp|P36776|LONM_HUMAN	HUMAN	Lon protease homolog, mitochondrial OS=Homo sapiens OX=9606 GN=LONP1 PE=1 SV=2
sp|P05556|ITB1_HUMAN	HUMAN	Integrin beta-1 OS=Homo sapiens OX=9606 GN=ITGB1 PE=1 SV=2
sp|O43813|LANC1_HUMAN	HUMAN	Glutathione S-transferase LANCL1 OS=Homo sapiens OX=9606 GN=LANCL1 PE=1 SV=1
sp|Q9BV57|MTND_HUMAN	HUMAN	1,2-dihydroxy-3-keto-5-methylthiopentene dioxygenase OS=Homo sapiens OX=9606 GN=ADI1 PE=1 SV=1
sp|Q13576|IQGA2_HUMAN	HUMAN	Ras GTPase-activating-like protein IQGAP2 OS=Homo sapiens OX=9606 GN=IQGAP2 PE=1 SV=4
sp|Q01082|SPTB2_HUMAN	HUMAN	Spectrin beta chain, non-erythrocytic 1 OS=Homo sapiens OX=9606 GN=SPTBN1 PE=1 SV=2
sp|P58546|MTPN_HUMAN	HUMAN	Myotrophin OS=Homo sapiens OX=9606 GN=MTPN PE=1 SV=2
sp|P35251|RFC1_HUMAN	HUMAN	Replication factor C subunit 1 OS=Homo sapiens OX=9606 GN=RFC1 PE=1 SV=4
sp|Q8N9N8|EIF1A_HUMAN	HUMAN	Probable RNA-binding protein EIF1AD OS=Homo sapiens OX=9606 GN=EIF1AD PE=1 SV=1
sp|P11717|MPRI_HUMAN	HUMAN	Cation-independent mannose-6-phosphate receptor OS=Homo sapiens OX=9606 GN=IGF2R PE=1 SV=3
sp|O75608|LYPA1_HUMAN	HUMAN	Acyl-protein thioesterase 1 OS=Homo sapiens OX=9606 GN=LYPLA1 PE=1 SV=1
sp|P13929|ENOB_HUMAN	HUMAN	Beta-enolase OS=Homo sapiens OX=9606 GN=ENO3 PE=1 SV=5
sp|P61026|RAB10_HUMAN	HUMAN	Ras-related protein Rab-10 OS=Homo sapiens OX=9606 GN=RAB10 PE=1 SV=1
sp|Q9BTW9|TBCD_HUMAN	HUMAN	Tubulin-specific chaperone D OS=Homo sapiens OX=9606 GN=TBCD PE=1 SV=2
sp|O95071|UBR5_HUMAN	HUMAN	E3 ubiquitin-protein ligase UBR5 OS=Homo sapiens OX=9606 GN=UBR5 PE=1 SV=2
sp|Q9H974|QTRT2_HUMAN	HUMAN	Queuine tRNA-ribosyltransferase accessory subunit 2 OS=Homo sapiens OX=9606 GN=QTRT2 PE=1 SV=1
sp|P11279|LAMP1_HUMAN	HUMAN	Lysosome-associated membrane glycoprotein 1 OS=Homo sapiens OX=9606 GN=LAMP1 PE=1 SV=3
sp|P46013|KI67_HUMAN	HUMAN	Proliferation marker protein Ki-67 OS=Homo sapiens OX=9606 GN=MKI67 PE=1 SV=2
sp|Q86WR0|CCD25_HUMAN	HUMAN	Coiled-coil domain-containing protein 25 OS=Homo sapiens OX=9606 GN=CCDC25 PE=1 SV=2
sp|P78344|IF4G2_HUMAN	HUMAN	Eukaryotic translation initiation factor 4 gamma 2 OS=Homo sapiens OX=9606 GN=EIF4G2 PE=1 SV=1
sp|Q9Y570|PPME1_HUMAN	HUMAN	Protein phosphatase methylesterase 1 OS=Homo sapiens OX=9606 GN=PPME1 PE=1 SV=3
sp|Q9NZB2|F120A_HUMAN	HUMAN	Constitutive coactivator of PPAR-gamma-like protein 1 OS=Homo sapiens OX=9606 GN=FAM120A PE=1 SV=2
sp|Q9BRT9|SLD5_HUMAN	HUMAN	DNA replication complex GINS protein SLD5 OS=Homo sapiens OX=9606 GN=GINS4 PE=1 SV=1
sp|Q99700|ATX2_HUMAN	HUMAN	Ataxin-2 OS=Homo sapiens OX=9606 GN=ATXN2 PE=1 SV=2
sp|Q96G46|DUS3L_HUMAN	HUMAN	tRNA-dihydrouridine(47) synthase [NAD(P)(+)]-like OS=Homo sapiens OX=9606 GN=DUS3L PE=1 SV=2
sp|Q8N8A2|ANR44_HUMAN	HUMAN	Serine/threonine-protein phosphatase 6 regulatory ankyrin repeat subunit B OS=Homo sapiens OX=9606 GN=ANKRD44 PE=1 SV=3
sp|Q7L591|DOK3_HUMAN	HUMAN	Docking protein 3 OS=Homo sapiens OX=9606 GN=DOK3 PE=1 SV=2
sp|Q6YP21|KAT3_HUMAN	HUMAN	Kynurenine--oxoglutarate transaminase 3 OS=Homo sapiens OX=9606 GN=KYAT3 PE=1 SV=1
sp|Q5VW32|BROX_HUMAN	HUMAN	BRO1 domain-containing protein BROX OS=Homo sapiens OX=9606 GN=BROX PE=1 SV=1
sp|P62304|RUXE_HUMAN	HUMAN	Small nuclear ribonucleoprotein E OS=Homo sapiens OX=9606 GN=SNRPE PE=1 SV=1

sp|P55212|CASP6_HUMAN	HUMAN	Caspase-6 OS=Homo sapiens OX=9606 GN=CASP6 PE=1 SV=2
sp|P53582|MAP11_HUMAN	HUMAN	Methionine aminopeptidase 1 OS=Homo sapiens OX=9606 GN=METAP1 PE=1 SV=2
sp|P60709|ACTB_HUMAN	HUMAN	Actin, cytoplasmic 1 OS=Homo sapiens OX=9606 GN=ACTB PE=1 SV=1
sp|Q71U36|TBA1A_HUMAN	HUMAN	Tubulin alpha-1A chain OS=Homo sapiens OX=9606 GN=TUBA1A PE=1 SV=1
sp|Q13509|TBB3_HUMAN	HUMAN	Tubulin beta-3 chain OS=Homo sapiens OX=9606 GN=TUBB3 PE=1 SV=2
sp|P36873|PP1G_HUMAN	HUMAN	Serine/threonine-protein phosphatase PP1-gamma catalytic subunit OS=Homo sapiens OX=9606 GN=PPP1CC PE=1 SV=1
sp|P62714|PP2AB_HUMAN	HUMAN	Serine/threonine-protein phosphatase 2A catalytic subunit beta isoform OS=Homo sapiens OX=9606 GN=PPP2CB PE=1 SV=1
sp|P30154|2AAB_HUMAN	HUMAN	Serine/threonine-protein phosphatase 2A 65 kDa regulatory subunit A beta isoform OS=Homo sapiens OX=9606 GN=PPP2R1B PE=1 SV=3
sp|P08134|RHOC_HUMAN	HUMAN	Rho-related GTP-binding protein RhoC OS=Homo sapiens OX=9606 GN=RHOC PE=1 SV=1
sp|O00148|DX39A_HUMAN	HUMAN	ATP-dependent RNA helicase DDX39A OS=Homo sapiens OX=9606 GN=DDX39A PE=1 SV=2
sp|Q71UI9|H2AV_HUMAN	HUMAN	Histone H2A.V OS=Homo sapiens OX=9606 GN=H2AFV PE=1 SV=3
sp|P08754|GNAI3_HUMAN	HUMAN	Guanine nucleotide-binding protein G(i) subunit alpha OS=Homo sapiens OX=9606 GN=GNAI3 PE=1 SV=3
sp|P08779|K1C16_HUMAN	HUMAN	Keratin, type I cytoskeletal 16 OS=Homo sapiens OX=9606 GN=KRT16 PE=1 SV=4
sp|Q14344|GNA13_HUMAN	HUMAN	Guanine nucleotide-binding protein subunit alpha-13 OS=Homo sapiens OX=9606 GN=GNA13 PE=1 SV=2
sp|P54725|RD23A_HUMAN	HUMAN	UV excision repair protein RAD23 homolog A OS=Homo sapiens OX=9606 GN=RAD23A PE=1 SV=1
sp|Q9H2G2|SLK_HUMAN	HUMAN	STE20-like serine/threonine-protein kinase OS=Homo sapiens OX=9606 GN=SLK PE=1 SV=1
sp|P51114|FXR1_HUMAN	HUMAN	Fragile X mental retardation syndrome-related protein 1 OS=Homo sapiens OX=9606 GN=FXR1 PE=1 SV=3
sp|O00505|IMA4_HUMAN	HUMAN	Importin subunit alpha-4 OS=Homo sapiens OX=9606 GN=KPNA3 PE=1 SV=2
sp|O60306|AQR_HUMAN	HUMAN	RNA helicase aquarius OS=Homo sapiens OX=9606 GN=AQR PE=1 SV=4
sp|O60231|DHX16_HUMAN	HUMAN	Pre-mRNA-splicing factor ATP-dependent RNA helicase DHX16 OS=Homo sapiens OX=9606 GN=DHX16 PE=1 SV=2
sp|P06493|CDK1_HUMAN	HUMAN	Cyclin-dependent kinase 1 OS=Homo sapiens OX=9606 GN=CDK1 PE=1 SV=3
sp|P17612|KAPCA_HUMAN	HUMAN	cAMP-dependent protein kinase catalytic subunit alpha OS=Homo sapiens OX=9606 GN=PRKACA PE=1 SV=2
sp|Q12792|TWF1_HUMAN	HUMAN	Twinfilin-1 OS=Homo sapiens OX=9606 GN=TWF1 PE=1 SV=3
sp|Q9UPW6|SATB2_HUMAN	HUMAN	DNA-binding protein SATB2 OS=Homo sapiens OX=9606 GN=SATB2 PE=1 SV=2
sp|P20810|ICAL_HUMAN	HUMAN	Calpastatin OS=Homo sapiens OX=9606 GN=CAST PE=1 SV=4
sp|Q9Y3B8|ORN_HUMAN	HUMAN	Oligoribonuclease, mitochondrial OS=Homo sapiens OX=9606 GN=REXO2 PE=1 SV=3
sp|Q13094|LCP2_HUMAN	HUMAN	Lymphocyte cytosolic protein 2 OS=Homo sapiens OX=9606 GN=LCP2 PE=1 SV=1
sp|Q9BU89|DOHH_HUMAN	HUMAN	Deoxyhypusine hydroxylase OS=Homo sapiens OX=9606 GN=DOHH PE=1 SV=1

sp|Q01658|NC2B_HUMAN	HUMAN	Protein Dr1 OS=Homo sapiens OX=9606 GN=DR1 PE=1 SV=1
sp|Q00653|NFKB2_HUMAN	HUMAN	Nuclear factor NF-kappa-B p100 subunit OS=Homo sapiens OX=9606 GN=NFKB2 PE=1 SV=4
sp|P49023|PAXI_HUMAN	HUMAN	Paxillin OS=Homo sapiens OX=9606 GN=PXN PE=1 SV=3
sp|O75717|WDHD1_HUMAN	HUMAN	WD repeat and HMG-box DNA-binding protein 1 OS=Homo sapiens OX=9606 GN=WDHD1 PE=1 SV=1
sp|Q15042|RB3GP_HUMAN	HUMAN	Rab3 GTPase-activating protein catalytic subunit OS=Homo sapiens OX=9606 GN=RAB3GAP1 PE=1 SV=3
sp|Q9UBB6|NCDN_HUMAN	HUMAN	Neurochondrin OS=Homo sapiens OX=9606 GN=NCDN PE=1 SV=1
sp|Q9NUK0|MBNL3_HUMAN	HUMAN	Muscleblind-like protein 3 OS=Homo sapiens OX=9606 GN=MBNL3 PE=1 SV=2
sp|Q8N1F7|NUP93_HUMAN	HUMAN	Nuclear pore complex protein Nup93 OS=Homo sapiens OX=9606 GN=NUP93 PE=1 SV=2
sp|Q32P28|P3H1_HUMAN	HUMAN	Prolyl 3-hydroxylase 1 OS=Homo sapiens OX=9606 GN=P3H1 PE=1 SV=2
sp|P18621|RL17_HUMAN	HUMAN	60S ribosomal protein L17 OS=Homo sapiens OX=9606 GN=RPL17 PE=1 SV=3
sp|P15090|FABP4_HUMAN	HUMAN	Fatty acid-binding protein, adipocyte OS=Homo sapiens OX=9606 GN=FABP4 PE=1 SV=3
sp|Q9Y6Y8|S23IP_HUMAN	HUMAN	SEC23-interacting protein OS=Homo sapiens OX=9606 GN=SEC23IP PE=1 SV=1
sp|Q9UPN7|PP6R1_HUMAN	HUMAN	Serine/threonine-protein phosphatase 6 regulatory subunit 1 OS=Homo sapiens OX=9606 GN=PPP6R1 PE=1 SV=5
sp|Q9UEE9|CFDP1_HUMAN	HUMAN	Craniofacial development protein 1 OS=Homo sapiens OX=9606 GN=CFDP1 PE=1 SV=1
sp|Q9NWS0|PIHD1_HUMAN	HUMAN	PIH1 domain-containing protein 1 OS=Homo sapiens OX=9606 GN=PIH1D1 PE=1 SV=1
sp|Q9NVT9|ARMC1_HUMAN	HUMAN	Armadillo repeat-containing protein 1 OS=Homo sapiens OX=9606 GN=ARMC1 PE=1 SV=1
sp|Q9H568|ACTL8_HUMAN	HUMAN	Actin-like protein 8 OS=Homo sapiens OX=9606 GN=ACTL8 PE=1 SV=1
sp|Q9H2U1|DHX36_HUMAN	HUMAN	ATP-dependent DNA/RNA helicase DHX36 OS=Homo sapiens OX=9606 GN=DHX36 PE=1 SV=2
sp|Q9H0L4|CSTFT_HUMAN	HUMAN	Cleavage stimulation factor subunit 2 tau variant OS=Homo sapiens OX=9606 GN=CSTF2T PE=1 SV=1
sp|Q9GZZ1|NAA50_HUMAN	HUMAN	N-alpha-acetyltransferase 50 OS=Homo sapiens OX=9606 GN=NAA50 PE=1 SV=1
sp|Q9BWU0|NADAP_HUMAN	HUMAN	Kanadaptin OS=Homo sapiens OX=9606 GN=SLC4A1AP PE=1 SV=1
sp|Q9BPX5|ARP5L_HUMAN	HUMAN	Actin-related protein 2/3 complex subunit 5-like protein OS=Homo sapiens OX=9606 GN=ARPC5L PE=1 SV=1
sp|Q99543|DNJC2_HUMAN	HUMAN	DnaJ homolog subfamily C member 2 OS=Homo sapiens OX=9606 GN=DNAJC2 PE=1 SV=4
sp|Q96Q15|SMG1_HUMAN	HUMAN	Serine/threonine-protein kinase SMG1 OS=Homo sapiens OX=9606 GN=SMG1 PE=1 SV=3
sp|Q96GD0|PLPP_HUMAN	HUMAN	Pyridoxal phosphate phosphatase OS=Homo sapiens OX=9606 GN=PDXP PE=1 SV=2
sp|Q96AB3|ISOC2_HUMAN	HUMAN	Isochorismatase domain-containing protein 2 OS=Homo sapiens OX=9606 GN=ISOC2 PE=1 SV=1
sp|Q92620|PRP16_HUMAN	HUMAN	Pre-mRNA-splicing factor ATP-dependent RNA helicase PRP16 OS=Homo sapiens OX=9606 GN=DHX38 PE=1 SV=2
sp|Q8N1G2|CMTR1_HUMAN	HUMAN	Cap-specific mRNA (nucleoside-2'-O-)-methyltransferase 1 OS=Homo sapiens OX=9606 GN=CMTR1 PE=1 SV=1

sp|Q15208|STK38_HUMAN	HUMAN	Serine/threonine-protein kinase 38 OS=Homo sapiens OX=9606 GN=STK38 PE=1 SV=1
sp|P80297|MT1X_HUMAN	HUMAN	Metallothionein-1X OS=Homo sapiens OX=9606 GN=MT1X PE=1 SV=1
sp|P62266|RS23_HUMAN	HUMAN	40S ribosomal protein S23 OS=Homo sapiens OX=9606 GN=RPS23 PE=1 SV=3
sp|P55039|DRG2_HUMAN	HUMAN	Developmentally-regulated GTP-binding protein 2 OS=Homo sapiens OX=9606 GN=DRG2 PE=1 SV=1
sp|P46736|BRCC3_HUMAN	HUMAN	Lys-63-specific deubiquitinase BRCC36 OS=Homo sapiens OX=9606 GN=BRCC3 PE=1 SV=2
sp|P42898|MTHR_HUMAN	HUMAN	Methylenetetrahydrofolate reductase OS=Homo sapiens OX=9606 GN=MTHFR PE=1 SV=3
sp|P32321|DCTD_HUMAN	HUMAN	Deoxycytidylate deaminase OS=Homo sapiens OX=9606 GN=DCTD PE=1 SV=2
sp|P29083|T2EA_HUMAN	HUMAN	General transcription factor IIE subunit 1 OS=Homo sapiens OX=9606 GN=GTF2E1 PE=1 SV=2
sp|P24928|RPB1_HUMAN	HUMAN	DNA-directed RNA polymerase II subunit RPB1 OS=Homo sapiens OX=9606 GN=POLR2A PE=1 SV=2
sp|P21283|VATC1_HUMAN	HUMAN	V-type proton ATPase subunit C 1 OS=Homo sapiens OX=9606 GN=ATP6V1C1 PE=1 SV=4
sp|O95379|TFIP8_HUMAN	HUMAN	Tumor necrosis factor alpha-induced protein 8 OS=Homo sapiens OX=9606 GN=TNFAIP8 PE=1 SV=1
sp|O94888|UBXN7_HUMAN	HUMAN	UBX domain-containing protein 7 OS=Homo sapiens OX=9606 GN=UBXN7 PE=1 SV=2
sp|O75718|CRTAP_HUMAN	HUMAN	Cartilage-associated protein OS=Homo sapiens OX=9606 GN=CRTAP PE=1 SV=1
sp|O60568|PLOD3_HUMAN	HUMAN	Multifunctional procollagen lysine hydroxylase and glycosyltransferase LH3 OS=Homo sapiens OX=9606 GN=PLOD3 PE=1 SV=1
sp|O60504|VINEX_HUMAN	HUMAN	Vinexin OS=Homo sapiens OX=9606 GN=SORBS3 PE=1 SV=2
sp|O14964|HGS_HUMAN	HUMAN	Hepatocyte growth factor-regulated tyrosine kinase substrate OS=Homo sapiens OX=9606 GN=HGS PE=1 SV=1
sp|O00743|PPP6_HUMAN	HUMAN	Serine/threonine-protein phosphatase 6 catalytic subunit OS=Homo sapiens OX=9606 GN=PPP6C PE=1 SV=1
sp|Q9Y5P4|CERT_HUMAN	HUMAN	Ceramide transfer protein OS=Homo sapiens OX=9606 GN=CERT1 PE=1 SV=1
sp|Q9Y3D8|KAD6_HUMAN	HUMAN	Adenylate kinase isoenzyme 6 OS=Homo sapiens OX=9606 GN=AK6 PE=1 SV=1
sp|Q9Y3B4|SF3B6_HUMAN	HUMAN	Splicing factor 3B subunit 6 OS=Homo sapiens OX=9606 GN=SF3B6 PE=1 SV=1
sp|Q9Y371|SHLB1_HUMAN	HUMAN	Endophilin-B1 OS=Homo sapiens OX=9606 GN=SH3GLB1 PE=1 SV=1
sp|Q9Y2V2|CHSP1_HUMAN	HUMAN	Calcium-regulated heat-stable protein 1 OS=Homo sapiens OX=9606 GN=CARHSP1 PE=1 SV=2
sp|Q9UN37|VPS4A_HUMAN	HUMAN	Vacuolar protein sorting-associated protein 4A OS=Homo sapiens OX=9606 GN=VPS4A PE=1 SV=1
sp|Q9UJX5|APC4_HUMAN	HUMAN	Anaphase-promoting complex subunit 4 OS=Homo sapiens OX=9606 GN=ANAPC4 PE=1 SV=2
sp|Q9UJX4|APC5_HUMAN	HUMAN	Anaphase-promoting complex subunit 5 OS=Homo sapiens OX=9606 GN=ANAPC5 PE=1 SV=2
sp|Q9UHW5|GPN3_HUMAN	HUMAN	GPN-loop GTPase 3 OS=Homo sapiens OX=9606 GN=GPN3 PE=1 SV=2
sp|Q9UET6|TRM7_HUMAN	HUMAN	Putative tRNA (cytidine(32)/guanosine(34)-2'-O)-methyltransferase OS=Homo sapiens OX=9606 GN=FTSJ1 PE=1 SV=2
sp|Q9NZ63|TLS1_HUMAN	HUMAN	Telomere length and silencing protein 1 homolog OS=Homo sapiens OX=9606 GN=C9orf78 PE=1 SV=1

sp|Q9NYB0|TE2IP_HUMAN	HUMAN	Telomeric repeat-binding factor 2-interacting protein 1 OS=Homo sapiens OX=9606 GN=TERF2IP PE=1 SV=1
sp|Q9NWY4|HPF1_HUMAN	HUMAN	Histone PARylation factor 1 OS=Homo sapiens OX=9606 GN=HPF1 PE=1 SV=2
sp|Q9NVM6|DJC17_HUMAN	HUMAN	DnaJ homolog subfamily C member 17 OS=Homo sapiens OX=9606 GN=DNAJC17 PE=1 SV=1
sp|Q9NVM4|ANM7_HUMAN	HUMAN	Protein arginine N-methyltransferase 7 OS=Homo sapiens OX=9606 GN=PRMT7 PE=1 SV=1
sp|Q9NQ88|TIGAR_HUMAN	HUMAN	Fructose-2,6-bisphosphatase TIGAR OS=Homo sapiens OX=9606 GN=TIGAR PE=1 SV=1
sp|Q9H944|MED20_HUMAN	HUMAN	Mediator of RNA polymerase II transcription subunit 20 OS=Homo sapiens OX=9606 GN=MED20 PE=1 SV=1
sp|Q9H8S9|MOB1A_HUMAN	HUMAN	MOB kinase activator 1A OS=Homo sapiens OX=9606 GN=MOB1A PE=1 SV=4
sp|Q9H814|PHAX_HUMAN	HUMAN	Phosphorylated adapter RNA export protein OS=Homo sapiens OX=9606 GN=PHAX PE=1 SV=1
sp|Q9H5X1|CIA2A_HUMAN	HUMAN	Cytosolic iron-sulfur assembly component 2A OS=Homo sapiens OX=9606 GN=CIAO2A PE=1 SV=1
sp|Q9H446|RWDD1_HUMAN	HUMAN	RWD domain-containing protein 1 OS=Homo sapiens OX=9606 GN=RWDD1 PE=1 SV=1
sp|Q9H0W9|CK054_HUMAN	HUMAN	Ester hydrolase C11orf54 OS=Homo sapiens OX=9606 GN=C11orf54 PE=1 SV=1
sp|Q9H008|LHPP_HUMAN	HUMAN	Phospholysine phosphohistidine inorganic pyrophosphate phosphatase OS=Homo sapiens OX=9606 GN=LHPP PE=1 SV=2
sp|Q9BV20|MTNA_HUMAN	HUMAN	Methylthioribose-1-phosphate isomerase OS=Homo sapiens OX=9606 GN=MRI1 PE=1 SV=1
sp|Q9BUH6|PAXX_HUMAN	HUMAN	Protein PAXX OS=Homo sapiens OX=9606 GN=PAXX PE=1 SV=2
sp|Q9BTE7|DCNL5_HUMAN	HUMAN	DCN1-like protein 5 OS=Homo sapiens OX=9606 GN=DCUN1D5 PE=1 SV=1
sp|Q9BSL1|UBAC1_HUMAN	HUMAN	Ubiquitin-associated domain-containing protein 1 OS=Homo sapiens OX=9606 GN=UBAC1 PE=1 SV=1
sp|Q9BRX5|PSF3_HUMAN	HUMAN	DNA replication complex GINS protein PSF3 OS=Homo sapiens OX=9606 GN=GINS3 PE=1 SV=1
sp|Q9BRJ6|CG050_HUMAN	HUMAN	Uncharacterized protein C7orf50 OS=Homo sapiens OX=9606 GN=C7orf50 PE=1 SV=1
sp|Q96S44|PRPK_HUMAN	HUMAN	EKC/KEOPS complex subunit TP53RK OS=Homo sapiens OX=9606 GN=TP53RK PE=1 SV=2
sp|Q96PU8|QKI_HUMAN	HUMAN	Protein quaking OS=Homo sapiens OX=9606 GN=QKI PE=1 SV=1
sp|Q96IJ6|GMPPA_HUMAN	HUMAN	Mannose-1-phosphate guanyltransferase alpha OS=Homo sapiens OX=9606 GN=GMPPA PE=1 SV=1
sp|Q96GA7|SDSL_HUMAN	HUMAN	Serine dehydratase-like OS=Homo sapiens OX=9606 GN=SDSL PE=1 SV=1
sp|Q96DH6|MSI2H_HUMAN	HUMAN	RNA-binding protein Musashi homolog 2 OS=Homo sapiens OX=9606 GN=MSI2 PE=1 SV=1
sp|Q96B36|AKTS1_HUMAN	HUMAN	Proline-rich AKT1 substrate 1 OS=Homo sapiens OX=9606 GN=AKT1S1 PE=1 SV=1
sp|Q96AT9|RPE_HUMAN	HUMAN	Ribulose-phosphate 3-epimerase OS=Homo sapiens OX=9606 GN=RPE PE=1 SV=1
sp|Q969E8|TSR2_HUMAN	HUMAN	Pre-rRNA-processing protein TSR2 homolog OS=Homo sapiens OX=9606 GN=TSR2 PE=1 SV=1
sp|Q92917|GPKOW_HUMAN	HUMAN	G-patch domain and KOW motifs-containing protein OS=Homo sapiens OX=9606 GN=GPKOW PE=1 SV=2
sp|Q92466|DDB2_HUMAN	HUMAN	DNA damage-binding protein 2 OS=Homo sapiens OX=9606 GN=DDB2 PE=1 SV=1
sp|Q8WZ82|OVCA2_HUMAN	HUMAN	Esterase OVCA2 OS=Homo sapiens OX=9606 GN=OVCA2 PE=1 SV=1
sp|Q8WX92|NELFB_HUMAN	HUMAN	Negative elongation factor B OS=Homo sapiens OX=9606 GN=NELFB PE=1 SV=1

sp|Q8TEA8|DTD1_HUMAN	HUMAN	D-aminoacyl-tRNA deacylase 1 OS=Homo sapiens OX=9606 GN=DTD1 PE=1 SV=2
sp|Q8TDN4|CABL1_HUMAN	HUMAN	CDK5 and ABL1 enzyme substrate 1 OS=Homo sapiens OX=9606 GN=CABLES1 PE=1 SV=2
sp|Q8TCD5|NT5C_HUMAN	HUMAN	5'(3')-deoxyribonucleotidase, cytosolic type OS=Homo sapiens OX=9606 GN=NT5C PE=1 SV=2
sp|Q8IZ73|RUSD2_HUMAN	HUMAN	RNA pseudouridylate synthase domain-containing protein 2 OS=Homo sapiens OX=9606 GN=RPUSD2 PE=1 SV=2
sp|Q8IXM2|BAP18_HUMAN	HUMAN	Chromatin complexes subunit BAP18 OS=Homo sapiens OX=9606 GN=BAP18 PE=1 SV=1
sp|Q8IWT0|ARCH_HUMAN	HUMAN	Protein archease OS=Homo sapiens OX=9606 GN=ZBTB8OS PE=1 SV=2
sp|Q86V21|AACS_HUMAN	HUMAN	Acetoacetyl-CoA synthetase OS=Homo sapiens OX=9606 GN=AACS PE=1 SV=1
sp|Q86SG5|S1A7A_HUMAN	HUMAN	Protein S100-A7A OS=Homo sapiens OX=9606 GN=S100A7A PE=1 SV=3
sp|Q7Z4H8|PLGT3_HUMAN	HUMAN	Protein O-glucosyltransferase 3 OS=Homo sapiens OX=9606 GN=POGLUT3 PE=1 SV=2
sp|Q7Z4G1|COMD6_HUMAN	HUMAN	COMM domain-containing protein 6 OS=Homo sapiens OX=9606 GN=COMMD6 PE=1 SV=1
sp|Q7Z2W4|ZCCHV_HUMAN	HUMAN	Zinc finger CCCH-type antiviral protein 1 OS=Homo sapiens OX=9606 GN=ZC3HAV1 PE=1 SV=3
sp|Q6P3X3|TTC27_HUMAN	HUMAN	Tetratricopeptide repeat protein 27 OS=Homo sapiens OX=9606 GN=TTC27 PE=1 SV=1
sp|Q68EM7|RHG17_HUMAN	HUMAN	Rho GTPase-activating protein 17 OS=Homo sapiens OX=9606 GN=ARHGAP17 PE=1 SV=1
sp|Q5T447|HECD3_HUMAN	HUMAN	E3 ubiquitin-protein ligase HECTD3 OS=Homo sapiens OX=9606 GN=HECTD3 PE=1 SV=1
sp|Q4V328|GRAP1_HUMAN	HUMAN	GRIP1-associated protein 1 OS=Homo sapiens OX=9606 GN=GRIPAP1 PE=1 SV=2
sp|Q16698|DECR_HUMAN	HUMAN	2,4-dienoyl-CoA reductase, mitochondrial OS=Homo sapiens OX=9606 GN=DECR1 PE=1 SV=1
sp|Q15427|SF3B4_HUMAN	HUMAN	Splicing factor 3B subunit 4 OS=Homo sapiens OX=9606 GN=SF3B4 PE=1 SV=1
sp|Q14691|PSF1_HUMAN	HUMAN	DNA replication complex GINS protein PSF1 OS=Homo sapiens OX=9606 GN=GINS1 PE=1 SV=1
sp|Q14498|RBM39_HUMAN	HUMAN	RNA-binding protein 39 OS=Homo sapiens OX=9606 GN=RBM39 PE=1 SV=2
sp|Q14331|FRG1_HUMAN	HUMAN	Protein FRG1 OS=Homo sapiens OX=9606 GN=FRG1 PE=1 SV=1
sp|Q13112|CAF1B_HUMAN	HUMAN	Chromatin assembly factor 1 subunit B OS=Homo sapiens OX=9606 GN=CHAF1B PE=1 SV=1
sp|Q12933|TRAF2_HUMAN	HUMAN	TNF receptor-associated factor 2 OS=Homo sapiens OX=9606 GN=TRAF2 PE=1 SV=2
sp|Q01081|U2AF1_HUMAN	HUMAN	Splicing factor U2AF 35 kDa subunit OS=Homo sapiens OX=9606 GN=U2AF1 PE=1 SV=3
sp|Q00169|PIPNA_HUMAN	HUMAN	Phosphatidylinositol transfer protein alpha isoform OS=Homo sapiens OX=9606 GN=PITPNA PE=1 SV=2
sp|P98179|RBM3_HUMAN	HUMAN	RNA-binding protein 3 OS=Homo sapiens OX=9606 GN=RBM3 PE=1 SV=1
sp|P63272|SPT4H_HUMAN	HUMAN	Transcription elongation factor SPT4 OS=Homo sapiens OX=9606 GN=SUPT4H1 PE=1 SV=1
sp|P62308|RUXG_HUMAN	HUMAN	Small nuclear ribonucleoprotein G OS=Homo sapiens OX=9606 GN=SNRPG PE=1 SV=1
sp|P61960|UFM1_HUMAN	HUMAN	Ubiquitin-fold modifier 1 OS=Homo sapiens OX=9606 GN=UFM1 PE=1 SV=1
sp|P61626|LYSC_HUMAN	HUMAN	Lysozyme C OS=Homo sapiens OX=9606 GN=LYZ PE=1 SV=1
sp|P61599|NAA20_HUMAN	HUMAN	N-alpha-acetyltransferase 20 OS=Homo sapiens OX=9606 GN=NAA20 PE=1 SV=1

sp|P53611|PGTB2_HUMAN	HUMAN	Geranylgeranyl transferase type-2 subunit beta OS=Homo sapiens OX=9606 GN=RABGGTB PE=1 SV=2
sp|P53602|MVD1_HUMAN	HUMAN	Diphosphomevalonate decarboxylase OS=Homo sapiens OX=9606 GN=MVD PE=1 SV=1
sp|P52758|RIDA_HUMAN	HUMAN	2-iminobutanoate/2-iminopropanoate deaminase OS=Homo sapiens OX=9606 GN=RIDA PE=1 SV=1
sp|P49840|GSK3A_HUMAN	HUMAN	Glycogen synthase kinase-3 alpha OS=Homo sapiens OX=9606 GN=GSK3A PE=1 SV=2
sp|P49755|TMEDA_HUMAN	HUMAN	Transmembrane emp24 domain-containing protein 10 OS=Homo sapiens OX=9606 GN=TMED10 PE=1 SV=2
sp|P49459|UBE2A_HUMAN	HUMAN	Ubiquitin-conjugating enzyme E2 A OS=Homo sapiens OX=9606 GN=UBE2A PE=1 SV=2
sp|P40306|PSB10_HUMAN	HUMAN	Proteasome subunit beta type-10 OS=Homo sapiens OX=9606 GN=PSMB10 PE=1 SV=1
sp|P40222|TXLNA_HUMAN	HUMAN	Alpha-taxilin OS=Homo sapiens OX=9606 GN=TXLNA PE=1 SV=3
sp|P35270|SPRE_HUMAN	HUMAN	Sepiapterin reductase OS=Homo sapiens OX=9606 GN=SPR PE=1 SV=1
sp|P32929|CGL_HUMAN	HUMAN	Cystathionine gamma-lyase OS=Homo sapiens OX=9606 GN=CTH PE=1 SV=3
sp|P31040|SDHA_HUMAN	HUMAN	Succinate dehydrogenase [ubiquinone] flavoprotein subunit, mitochondrial OS=Homo sapiens OX=9606 GN=SDHA PE=1 SV=2
sp|P21912|SDHB_HUMAN	HUMAN	Succinate dehydrogenase [ubiquinone] iron-sulfur subunit, mitochondrial OS=Homo sapiens OX=9606 GN=SDHB PE=1 SV=3
sp|P20962|PTMS_HUMAN	HUMAN	Parathymosin OS=Homo sapiens OX=9606 GN=PTMS PE=1 SV=2
sp|P19387|RPB3_HUMAN	HUMAN	DNA-directed RNA polymerase II subunit RPB3 OS=Homo sapiens OX=9606 GN=POLR2C PE=1 SV=2
sp|P16333|NCK1_HUMAN	HUMAN	Cytoplasmic protein NCK1 OS=Homo sapiens OX=9606 GN=NCK1 PE=1 SV=1
sp|P11234|RALB_HUMAN	HUMAN	Ras-related protein Ral-B OS=Homo sapiens OX=9606 GN=RALB PE=1 SV=1
sp|P10153|RNAS2_HUMAN	HUMAN	Non-secretory ribonuclease OS=Homo sapiens OX=9606 GN=RNASE2 PE=1 SV=2
sp|P0DPB6|RPAC2_HUMAN	HUMAN	DNA-directed RNA polymerases I and III subunit RPAC2 OS=Homo sapiens OX=9606 GN=POLR1D PE=1 SV=1
sp|P08397|HEM3_HUMAN	HUMAN	Porphobilinogen deaminase OS=Homo sapiens OX=9606 GN=HMBS PE=1 SV=2
sp|P05386|RLA1_HUMAN	HUMAN	60S acidic ribosomal protein P1 OS=Homo sapiens OX=9606 GN=RPLP1 PE=1 SV=1
sp|P01040|CYTA_HUMAN	HUMAN	Cystatin-A OS=Homo sapiens OX=9606 GN=CSTA PE=1 SV=1
sp|O95777|LSM8_HUMAN	HUMAN	U6 snRNA-associated Sm-like protein LSm8 OS=Homo sapiens OX=9606 GN=LSM8 PE=1 SV=3
sp|O95372|LYPA2_HUMAN	HUMAN	Acyl-protein thioesterase 2 OS=Homo sapiens OX=9606 GN=LYPLA2 PE=1 SV=1
sp|O95319|CELF2_HUMAN	HUMAN	CUGBP Elav-like family member 2 OS=Homo sapiens OX=9606 GN=CELF2 PE=1 SV=1
sp|O75886|STAM2_HUMAN	HUMAN	Signal transducing adapter molecule 2 OS=Homo sapiens OX=9606 GN=STAM2 PE=1 SV=1
sp|O75396|SC22B_HUMAN	HUMAN	Vesicle-trafficking protein SEC22b OS=Homo sapiens OX=9606 GN=SEC22B PE=1 SV=4
sp|O75379|VAMP4_HUMAN	HUMAN	Vesicle-associated membrane protein 4 OS=Homo sapiens OX=9606 GN=VAMP4 PE=1 SV=2
sp|O60508|PRP17_HUMAN	HUMAN	Pre-mRNA-processing factor 17 OS=Homo sapiens OX=9606 GN=CDC40 PE=1 SV=1
sp|O43852|CALU_HUMAN	HUMAN	Calumenin OS=Homo sapiens OX=9606 GN=CALU PE=1 SV=2

sp|O43837|IDH3B_HUMAN	HUMAN	Isocitrate dehydrogenase [NAD] subunit beta, mitochondrial OS=Homo sapiens OX=9606 GN=IDH3B PE=1 SV=2
sp|O15260|SURF4_HUMAN	HUMAN	Surfeit locus protein 4 OS=Homo sapiens OX=9606 GN=SURF4 PE=1 SV=3
sp|O14828|SCAM3_HUMAN	HUMAN	Secretory carrier-associated membrane protein 3 OS=Homo sapiens OX=9606 GN=SCAMP3 PE=1 SV=3
sp|O14757|CHK1_HUMAN	HUMAN	Serine/threonine-protein kinase Chk1 OS=Homo sapiens OX=9606 GN=CHEK1 PE=1 SV=2
sp|O00193|SMAP_HUMAN	HUMAN	Small acidic protein OS=Homo sapiens OX=9606 GN=SMAP PE=1 SV=1
sp|A6NHR9|SMHD1_HUMAN	HUMAN	Structural maintenance of chromosomes flexible hinge domain-containing protein 1 OS=Homo sapiens OX=9606 GN=SMCHD1 PE=1 SV=2
sp|P22570|ADRO_HUMAN	HUMAN	NADPH:adrenodoxin oxidoreductase, mitochondrial OS=Homo sapiens OX=9606 GN=FDXR PE=1 SV=3
sp|P09668|CATH_HUMAN	HUMAN	Pro-cathepsin H OS=Homo sapiens OX=9606 GN=CTSH PE=1 SV=4
sp|P20339|RAB5A_HUMAN	HUMAN	Ras-related protein Rab-5A OS=Homo sapiens OX=9606 GN=RAB5A PE=1 SV=2
sp|O95721|SNP29_HUMAN	HUMAN	Synaptosomal-associated protein 29 OS=Homo sapiens OX=9606 GN=SNAP29 PE=1 SV=1
sp|G2XKQ0|SUMO5_HUMAN	HUMAN	Small ubiquitin-related modifier 5 OS=Homo sapiens OX=9606 GN=SUMO1P1 PE=1 SV=2
sp|Q86TU7|SETD3_HUMAN	HUMAN	Actin-histidine N-methyltransferase OS=Homo sapiens OX=9606 GN=SETD3 PE=1 SV=1
sp|O00418|EF2K_HUMAN	HUMAN	Eukaryotic elongation factor 2 kinase OS=Homo sapiens OX=9606 GN=EEF2K PE=1 SV=2
sp|P62312|LSM6_HUMAN	HUMAN	U6 snRNA-associated Sm-like protein LSm6 OS=Homo sapiens OX=9606 GN=LSM6 PE=1 SV=1
sp|Q9UH65|SWP70_HUMAN	HUMAN	Switch-associated protein 70 OS=Homo sapiens OX=9606 GN=SWAP70 PE=1 SV=1
sp|O94763|RMP_HUMAN	HUMAN	Unconventional prefoldin RPB5 interactor 1 OS=Homo sapiens OX=9606 GN=URI1 PE=1 SV=3
sp|P15559|NQO1_HUMAN	HUMAN	NAD(P)H dehydrogenase [quinone] 1 OS=Homo sapiens OX=9606 GN=NQO1 PE=1 SV=1
sp|Q9Y6E0|STK24_HUMAN	HUMAN	Serine/threonine-protein kinase 24 OS=Homo sapiens OX=9606 GN=STK24 PE=1 SV=1
sp|Q9BWF3|RBM4_HUMAN	HUMAN	RNA-binding protein 4 OS=Homo sapiens OX=9606 GN=RBM4 PE=1 SV=1
sp|Q69YN2|C19L1_HUMAN	HUMAN	CWF19-like protein 1 OS=Homo sapiens OX=9606 GN=CWF19L1 PE=1 SV=2
sp|Q7Z2T5|TRM1L_HUMAN	HUMAN	TRMT1-like protein OS=Homo sapiens OX=9606 GN=TRMT1L PE=1 SV=2
sp|Q13541|4EBP1_HUMAN	HUMAN	Eukaryotic translation initiation factor 4E-binding protein 1 OS=Homo sapiens OX=9606 GN=EIF4EBP1 PE=1 SV=3
sp|O60264|SMCA5_HUMAN	HUMAN	SWI/SNF-related matrix-associated actin-dependent regulator of chromatin subfamily A member 5 OS=Homo sapiens OX=9606 GN=SMARCA5 PE=1 SV=1
sp|Q9NUD5|ZCHC3_HUMAN	HUMAN	Zinc finger CCHC domain-containing protein 3 OS=Homo sapiens OX=9606 GN=ZCCHC3 PE=1 SV=2
sp|P49137|MAPK2_HUMAN	HUMAN	MAP kinase-activated protein kinase 2 OS=Homo sapiens OX=9606 GN=MAPKAPK2 PE=1 SV=1
sp|P13473|LAMP2_HUMAN	HUMAN	Lysosome-associated membrane glycoprotein 2 OS=Homo sapiens OX=9606 GN=LAMP2 PE=1 SV=2
sp|O00329|PK3CD_HUMAN	HUMAN	Phosphatidylinositol 4,5-bisphosphate 3-kinase catalytic subunit delta isoform OS=Homo sapiens OX=9606 GN=PIK3CD PE=1 SV=2
sp|Q9H9Q2|CSN7B_HUMAN	HUMAN	COP9 signalosome complex subunit 7b OS=Homo sapiens OX=9606 GN=COPS7B PE=1 SV=1

sp|P30047|GFRP_HUMAN	HUMAN	GTP cyclohydrolase 1 feedback regulatory protein OS=Homo sapiens OX=9606 GN=GCHFR PE=1 SV=3
sp|Q14919|NC2A_HUMAN	HUMAN	Dr1-associated corepressor OS=Homo sapiens OX=9606 GN=DRAP1 PE=1 SV=3
sp|Q9UHR5|S30BP_HUMAN	HUMAN	SAP30-binding protein OS=Homo sapiens OX=9606 GN=SAP30BP PE=1 SV=1
sp|Q9NY27|PP4R2_HUMAN	HUMAN	Serine/threonine-protein phosphatase 4 regulatory subunit 2 OS=Homo sapiens OX=9606 GN=PPP4R2 PE=1 SV=3
sp|Q16650|TBR1_HUMAN	HUMAN	T-box brain protein 1 OS=Homo sapiens OX=9606 GN=TBR1 PE=1 SV=1
sp|P61764|STXB1_HUMAN	HUMAN	Syntaxin-binding protein 1 OS=Homo sapiens OX=9606 GN=STXBP1 PE=1 SV=1
sp|Q9UHJ6|SHPK_HUMAN	HUMAN	Sedoheptulokinase OS=Homo sapiens OX=9606 GN=SHPK PE=1 SV=3
sp|P61923|COPZ1_HUMAN	HUMAN	Coatomer subunit zeta-1 OS=Homo sapiens OX=9606 GN=COPZ1 PE=1 SV=1
sp|Q13242|SRSF9_HUMAN	HUMAN	Serine/arginine-rich splicing factor 9 OS=Homo sapiens OX=9606 GN=SRSF9 PE=1 SV=1
sp|P02786|TFR1_HUMAN	HUMAN	Transferrin receptor protein 1 OS=Homo sapiens OX=9606 GN=TFRC PE=1 SV=2
sp|Q9Y3L3|3BP1_HUMAN	HUMAN	SH3 domain-binding protein 1 OS=Homo sapiens OX=9606 GN=SH3BP1 PE=1 SV=3
sp|P16401|H15_HUMAN	HUMAN	Histone H1.5 OS=Homo sapiens OX=9606 GN=H1-5 PE=1 SV=3
sp|Q9UHD2|TBK1_HUMAN	HUMAN	Serine/threonine-protein kinase TBK1 OS=Homo sapiens OX=9606 GN=TBK1 PE=1 SV=1
sp|Q2TAY7|SMU1_HUMAN	HUMAN	WD40 repeat-containing protein SMU1 OS=Homo sapiens OX=9606 GN=SMU1 PE=1 SV=2
sp|O95376|ARI2_HUMAN	HUMAN	E3 ubiquitin-protein ligase ARIH2 OS=Homo sapiens OX=9606 GN=ARIH2 PE=1 SV=1
sp|Q00577|PURA_HUMAN	HUMAN	Transcriptional activator protein Pur-alpha OS=Homo sapiens OX=9606 GN=PURA PE=1 SV=2
sp|O75832|PSD10_HUMAN	HUMAN	26S proteasome non-ATPase regulatory subunit 10 OS=Homo sapiens OX=9606 GN=PSMD10 PE=1 SV=1
sp|P61326|MGN_HUMAN	HUMAN	Protein mago nashi homolog OS=Homo sapiens OX=9606 GN=MAGOH PE=1 SV=1
sp|O95989|NUDT3_HUMAN	HUMAN	Diphosphoinositol polyphosphate phosphohydrolase 1 OS=Homo sapiens OX=9606 GN=NUDT3 PE=1 SV=1
sp|Q7Z4Q2|HEAT3_HUMAN	HUMAN	HEAT repeat-containing protein 3 OS=Homo sapiens OX=9606 GN=HEATR3 PE=1 SV=2
sp|O95747|OXSR1_HUMAN	HUMAN	Serine/threonine-protein kinase OSR1 OS=Homo sapiens OX=9606 GN=OXSR1 PE=1 SV=1
sp|Q9Y2D5|AKAP2_HUMAN	HUMAN	A-kinase anchor protein 2 OS=Homo sapiens OX=9606 GN=AKAP2 PE=1 SV=3
sp|Q9BWT3|PAPOG_HUMAN	HUMAN	Poly(A) polymerase gamma OS=Homo sapiens OX=9606 GN=PAPOLG PE=1 SV=2
sp|Q8TBX8|PI42C_HUMAN	HUMAN	Phosphatidylinositol 5-phosphate 4-kinase type-2 gamma OS=Homo sapiens OX=9606 GN=PIP4K2C PE=1 SV=3
sp|P35573|GDE_HUMAN	HUMAN	Glycogen debranching enzyme OS=Homo sapiens OX=9606 GN=AGL PE=1 SV=3
sp|Q08AM6|VAC14_HUMAN	HUMAN	Protein VAC14 homolog OS=Homo sapiens OX=9606 GN=VAC14 PE=1 SV=1
sp|P08240|SRPRA_HUMAN	HUMAN	Signal recognition particle receptor subunit alpha OS=Homo sapiens OX=9606 GN=SRPRA PE=1 SV=2
sp|Q9Y237|PIN4_HUMAN	HUMAN	Peptidyl-prolyl cis-trans isomerase NIMA-interacting 4 OS=Homo sapiens OX=9606 GN=PIN4 PE=1 SV=1
sp|Q05519|SRS11_HUMAN	HUMAN	Serine/arginine-rich splicing factor 11 OS=Homo sapiens OX=9606 GN=SRSF11 PE=1 SV=1
sp|P52732|KIF11_HUMAN	HUMAN	Kinesin-like protein KIF11 OS=Homo sapiens OX=9606 GN=KIF11 PE=1 SV=2

sp|Q96PK6|RBM14_HUMAN	HUMAN	RNA-binding protein 14 OS=Homo sapiens OX=9606 GN=RBM14 PE=1 SV=2
sp|Q15154|PCM1_HUMAN	HUMAN	Pericentriolar material 1 protein OS=Homo sapiens OX=9606 GN=PCM1 PE=1 SV=5
sp|Q7Z4I7|LIMS2_HUMAN	HUMAN	LIM and senescent cell antigen-like-containing domain protein 2 OS=Homo sapiens OX=9606 GN=LIMS2 PE=1 SV=1
sp|Q8TB72|PUM2_HUMAN	HUMAN	Pumilio homolog 2 OS=Homo sapiens OX=9606 GN=PUM2 PE=1 SV=2
sp|Q9HAB8|PPCS_HUMAN	HUMAN	Phosphopantothenate--cysteine ligase OS=Homo sapiens OX=9606 GN=PPCS PE=1 SV=2
sp|P54252|ATX3_HUMAN	HUMAN	Ataxin-3 OS=Homo sapiens OX=9606 GN=ATXN3 PE=1 SV=5
sp|Q8IZ69|TRM2A_HUMAN	HUMAN	tRNA (uracil-5-)-methyltransferase homolog A OS=Homo sapiens OX=9606 GN=TRMT2A PE=1 SV=2
sp|Q86XP3|DDX42_HUMAN	HUMAN	ATP-dependent RNA helicase DDX42 OS=Homo sapiens OX=9606 GN=DDX42 PE=1 SV=1
sp|P08195|4F2_HUMAN	HUMAN	4F2 cell-surface antigen heavy chain OS=Homo sapiens OX=9606 GN=SLC3A2 PE=1 SV=3
sp|P49916|DNLI3_HUMAN	HUMAN	DNA ligase 3 OS=Homo sapiens OX=9606 GN=LIG3 PE=1 SV=2
sp|Q96EV8|DTBP1_HUMAN	HUMAN	Dysbindin OS=Homo sapiens OX=9606 GN=DTNBP1 PE=1 SV=1
sp|P50993|AT1A2_HUMAN	HUMAN	Sodium/potassium-transporting ATPase subunit alpha-2 OS=Homo sapiens OX=9606 GN=ATP1A2 PE=1 SV=1
sp|Q13131|AAPK1_HUMAN	HUMAN	5'-AMP-activated protein kinase catalytic subunit alpha-1 OS=Homo sapiens OX=9606 GN=PRKAA1 PE=1 SV=4
sp|Q04828|AK1C1_HUMAN	HUMAN	Aldo-keto reductase family 1 member C1 OS=Homo sapiens OX=9606 GN=AKR1C1 PE=1 SV=1
sp|O43747|AP1G1_HUMAN	HUMAN	AP-1 complex subunit gamma-1 OS=Homo sapiens OX=9606 GN=AP1G1 PE=1 SV=5
sp|O00139|KIF2A_HUMAN	HUMAN	Kinesin-like protein KIF2A OS=Homo sapiens OX=9606 GN=KIF2A PE=1 SV=3
sp|Q5JTZ9|SYAM_HUMAN	HUMAN	Alanine--tRNA ligase, mitochondrial OS=Homo sapiens OX=9606 GN=AARS2 PE=1 SV=1
sp|P35236|PTN7_HUMAN	HUMAN	Tyrosine-protein phosphatase non-receptor type 7 OS=Homo sapiens OX=9606 GN=PTPN7 PE=1 SV=3

Supplementary Figure S1: Lipidomic Analysis of U937 EVs and U937 CDNs. Lipids are quantified as a percentage of the total lipids. Data presented as mean ± SEM (n = 3). * indicates p < 0.001.**

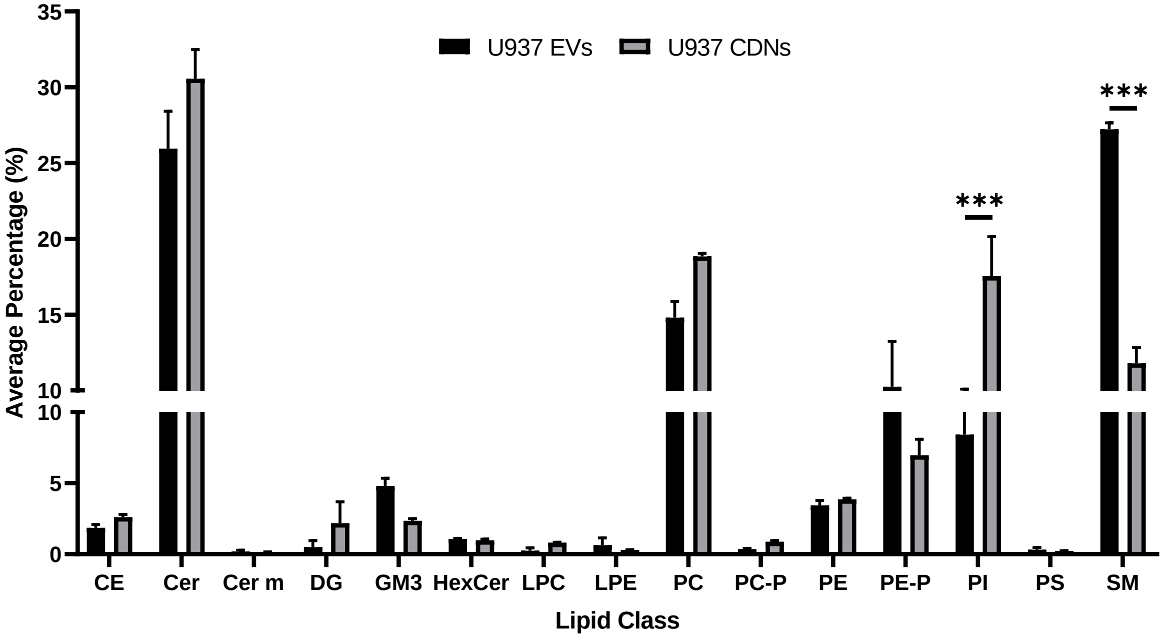

Supplementary Figure S2: Comparison of mean Ct values between U937 cells (indicated as CP on the x-axis) and U937 CDNs (indicated as CDNs on the y-axis). n = 2. Correlation coefficient (r^2) = 0.837. Figure prepared by NUSMed noncoding RNA Core Facility, National University of Singapore.

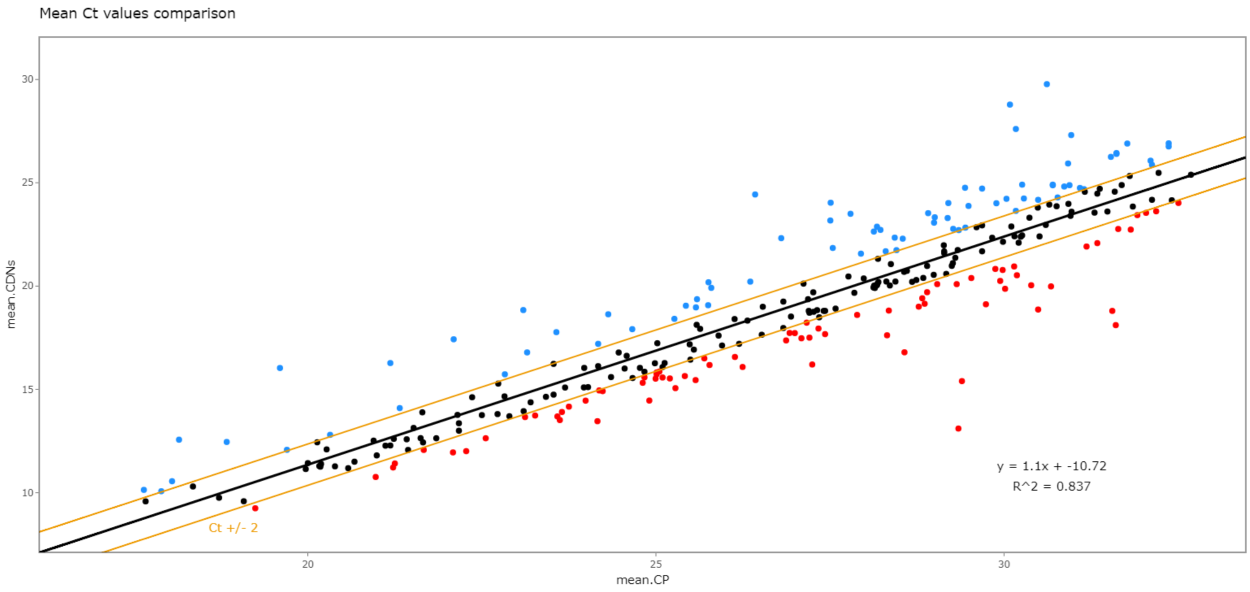

Supplement: Supplementary file 1 [file pharmaceutics-15-01290-s001.zip › pharmaceutics-2270456-supplementary.pdf]
